# Penguins reduced olfactory receptor genes common to other waterbirds

**DOI:** 10.1038/srep31671

**Published:** 2016-08-16

**Authors:** Qin Lu, Kai Wang, Fumin Lei, Dan Yu, Huabin Zhao

**Affiliations:** 1 Department of Ecology, College of Life Sciences, Wuhan University, Wuhan 430072, China; 2 Key Laboratory of Zoological Systematics and Evolution, Institute of Zoology, Chinese Academy of Sciences, Beijing 100101, China

## Abstract

The sense of smell, or olfaction, is fundamental in the life of animals. However, penguins (Aves: Sphenisciformes) possess relatively small olfactory bulbs compared with most other waterbirds such as Procellariiformes and Gaviiformes. To test whether penguins have a reduced reliance on olfaction, we analyzed the draft genome sequences of the two penguins, which diverged at the origin of the order Sphenisciformes; we also examined six closely related species with available genomes, and identified 29 one-to-one orthologous olfactory receptor genes (i.e. *OR*s) that are putatively functionally conserved and important across the eight birds. To survey the 29 one-to-one orthologous *OR*s in penguins and their relatives, we newly generated 34 sequences that are missing from the draft genomes. Through the analysis of totaling 378 *OR* sequences, we found that, of these functionally important *OR*s common to other waterbirds, penguins have a significantly greater percentage of *OR* pseudogenes than other waterbirds, suggesting a reduction of olfactory capability. The penguin-specific reduction of olfactory capability arose in the common ancestor of penguins between 23 and 60 Ma, which may have resulted from the aquatic specializations for underwater vision. Our study provides genetic evidence for a possible reduction of reliance on olfaction in penguins.

Traditionally, animals are believed to have five basic senses: sight, hearing, taste, touch, and smell^1^. All the five sensory modalities are able to perceive stimuli from the external environment and are thus of fundamental importance for animals’ survival. However, one or more of the five senses could be absent in some species of animals^2^,^3^. Even when animals possess same sensory modalities, their reliance on each sense may vary significantly among species. For example, mice have an increased reliance on olfaction compared to humans^4^, while vampire bats showed decreased dependence on taste relative to other bats^5^,^6^.

Of the five basic senses, the sense of smell, or olfaction, is fundamentally important in the life of animals, underpinning many essential behaviors such as food location, mate recognition, and predator avoidance^7^. Most mammals and reptiles typically possess two distinct olfactory systems: the main olfactory system (MOS) and the vomeronasal system (VNS), which were generally assumed to perceive environmental odorants and intraspecific pheromones, respectively^8^. The MOS was observed to primarily express olfactory receptors (ORs), which are encoded by olfactory receptor genes (*OR*s). The binding of odorants to ORs triggers the transduction of olfactory signals to the olfactory bulb in the brain, which results in olfactory perception^9^. The *OR*s make up one of the largest gene families in most vertebrate genomes, but the total number of *OR*s in each species varies dramatically, ranging from 125 in the pufferfish to 2129 in the cow^10^. The striking variation in the number of *OR*s may result from ecological adaptation. For instance, the loss of *OR*s occurred independently in the multiple lineages of aquatic mammals such as cetaceans and sirenians, which coincided with their habitat transition from land to water^11^,^12^. Furthermore, differences in the number of *OR*s may also reflect a tradeoff between olfaction and other senses. For instance, the reduction of *OR*s coincided with the independent acquisition of trichromatic color vision in multiple lineages of primates^13^. As such, investigating the evolutionary changes of *OR*s would provide a valuable route to understanding how genomes have been shaped by habitat transitions, sensory tradeoffs and other ecological adaptations.

Birds are the most species-rich group among tetrapod vertebrates, with diverse and distinct olfactory abilities. In general, they are assumed to have a poor olfactory system, as they seem to rely more on vision and vocalizations^14^. Indeed, birds were observed to have a much smaller *OR* gene repertoire than their closely related cousin, the reptiles^15^,^16^, which suggested a reduced reliance on olfaction. By contrast, numerous studies have argued that birds definitely use olfactory cues in many crucial behaviors, such as foraging, navigation and individual recognition^17^,^18^,^19^,^20^, which was also evidenced by recent genetic data^21^. The contrasting arguments with respect to avian olfaction call for further investigations.

As the only extant group of birds that occupy a secondarily aquatic niche with flightless wing-propelled diving, penguins (order Sphenisciformes) have undergone remarkable adaptations, such as streamlined bodies, flipper-like wings, dense bones and scale-like feathers^22^,^23^,^24^. In terms of sensory ecology, penguins are considered visual specialists, with a flat cornea and a spherical lens for underwater adaptation^25^,^26^. Consistent with the morphological adaptations, genetic studies have observed positive selection on phototransduction genes and accelerated evolution of visual opsin genes in penguins, which were also linked to the aquatic lifestyle^27^,^28^. By contrast, penguins have long been believed to lack a sense of smell, as they primarily rely on vision for underwater foraging^22^. In fact, multiple lines of evidence have demonstrated that penguins possess a functional sense of smell. For example, they smell the dimethyl sulphide (DMS) for prey location^29^,^30^,^31^ and perceive odors for kin recognition^32^. At the molecular level, penguins, along with other waterbirds (or aquatic birds), were identified to carry a significantly greater number of olfactory receptor genes, as compared with vocal-learning birds in a recent genomic analysis^15^, which also suggested that penguins rely heavily on olfaction. The recent genomic analysis classified the 48 birds into four categories: birds of prey, waterbirds, land birds and vocal learners and attempted to link the differences in *OR* gene numbers to ecological variations in the four categoires of birds^15^. However, the differences in *OR* gene numbers within one of the four categories were not examined^15^. Specifically, differences in olfaction between penguins and other waterbirds remain largely unknown. Compared with most other waterbirds, such as Procellariiformes, Ciconiiformes and Gaviiformes, penguins possess relatively small olfactory bulbs^33^, which are commonly used as a proxy for olfactory capability^34^. As such, we hypothesize that penguins may have a reduced number of *OR* genes as compared to other waterbirds. To test this hypothesis, we analyzed the draft genome sequences from the two penguins, which diverged at the origin of the order Sphenisciformes and represented the two major clades of the penguin species tree^35^; we also examined six closely related outgroup species with available genomes (Fig. 1), and identified 29 one-to-one orthologous olfactory receptor genes that are putatively functionally conserved and important across the eight birds. With additional sequencing of these orthologous genes, we found that penguins have a significantly higher percentage of pseudogenes than other waterbirds, although they still retain many intact and putatively functional genes.

## Results

### Survey of *OR* genes in the genomes of penguins and other waterbirds

**Figure 1:**
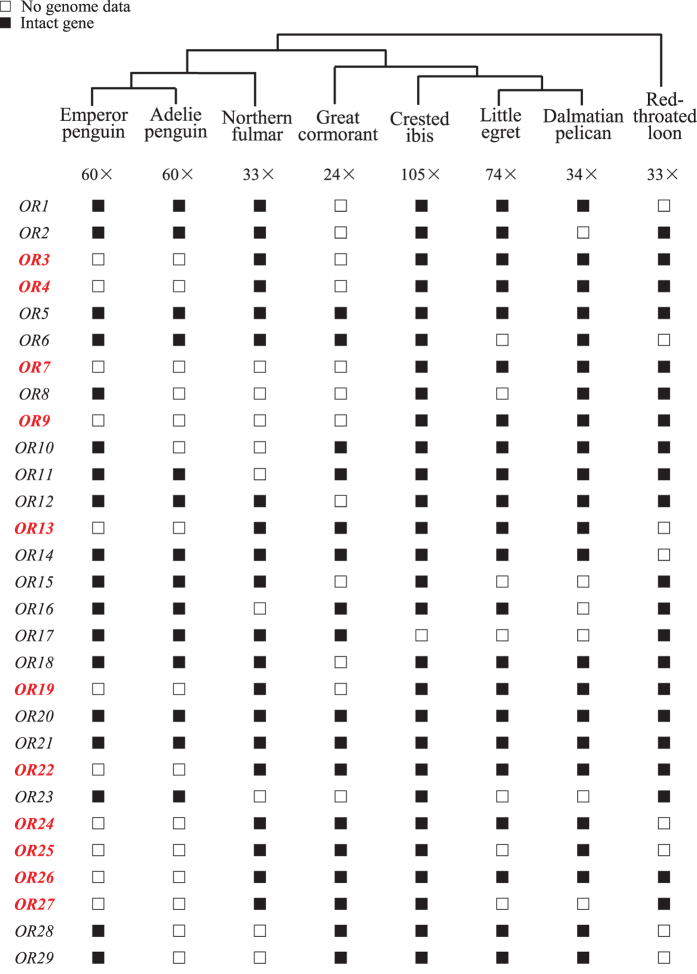
Genomic survey of olfactory receptor genes in the two penguins and six other waterbirds. The eight birds represent four avian orders, their phylogeny follows a recent study^36^. The sequencing coverage of each genome was shown under each common name of birds. A filled square indicates a gene with a full-length coding sequence and an intact open reading frame, whereas an open square refers to either a partial gene or an absence of genomic data. Genes that are potentially lost in penguins were highlighted in red.

Our genomic dataset of birds represented four avian orders, including two species of Sphenisciformes (Emperor penguin, *Aptenodytes forsteri*, 60× coverage; Adelie penguin, *Pygoscelis adeliae*, 60×), one species of Procellariiformes (Northern fulmar, *Fulmarus glacialis*, 33×), four species of Pelecaniformes (Crested ibis, *Nipponia Nippon*, 105×; Little egret, *Egretta garzetta*, 74×; Great cormorant, *Phalacrocorax carbo*, 24×; Dalmatian pelican, *Pelecanus crispus*, 34×), and one species of Gaviiformes (Red-throated loon, *Gavia stellata*, 33×) (Fig. 1; Supplementary Table S1)^36^,^37^. The four orders of birds are closely related, and were referred to as the core waterbirds^36^. Specifically, the penguin order (Sphenisciformes) is the most closely related to the order Procellariiformes; the two orders form a monophyletic group, which is clustered with the order Pelecaniformes; and the fourth order Gaviiformes falls outside of the other three orders (Fig. 1)^36^. In addition, the two penguins (i.e. the emperor penguin and the Adelie penguin) with genome sequences diverged at the origin of the order Sphenisciformes and represented the two major clades of the penguin species tree^35^.

A total of 344 full-length and intact *OR*s (see the identification procedure in Materials and Methods) were identified from the draft genome sequences of the emperor penguin (gene number: 32), Adelie penguin (26), northern fulmar (33), great cormorant (36), crested ibis (47), little egret (106), Dalmatian pelican (20), and red-throated loon (44) (Supplementary Table S1). The numbers of identified intact *OR*s in the present study are similar to those from a recent study with minor differences (Supplementary Table S1), which may result from slightly different bioinformatics approaches between the current work and a previous study^15^. Relative to most mammals, the low number of intact *OR*s in birds suggests a reduced reliance on olfaction, which is consistent with the common view that most birds are primarily visual animals^38^, because the reduction of *OR*s was coincident with the occurrence of better color vision in primates^13^. Notably, the little egret has an extraordinarily large number of *OR*s, indicating an extraordinary expansion as compared to other waterbirds^15^.

Phylogenetic analysis was performed using all intact *OR*s from the eight avian genomes. The resulting phylogenetic tree revealed a major clade comprising mostly the little egret genes, although the clade was not well supported by both phylogenetic methods (Supplementary Fig. S1). This clade consisted of 94 genes in which 75 genes are from the little egret, whereas the remaining clades contain only 31 little egret genes, suggesting an apparent gene expansion in the little egret (Supplementary Fig. S1), which was also observed in a recent analysis^15^. Of note, the monophyly of the major clade remains to be resolved in future. While the basal clades of the phylogenetic trees did not receive high supporting values, well-supported clades were found at the tips of many groupings (Supplementary Fig. S1). We identified 29 potentially one-to-one orthologous genes with the following criteria. First, we selected well-supported clades with a bootstrap value greater than 85%; Second, there is only one single-copy gene from each species; Third, the single-copy gene was detected from at least four avian species in a well-supported clade. The nomenclature of the 29 genes followed the HORDE database (Supplementary Fig. S1)^39^. For convenience, we also named each of the 29 genes numerically in the order of appearance on the phylogenetic tree (Supplementary Fig. S1). These one-to-one orthologous genes are assumed to be functionally conserved in the eight birds, because the same olfactory receptors tend to detect similar odorants^40^. As such, the 29 orthologous genes are expected to be present in most of the eight avian species, and some genes are absent possibly because of incomplete genome sequencing or poor genome assembly.

To test whether penguins have specifically lost some of the 29 orthologous genes, we mapped these genes onto the species phylogeny of the two penguins and six related waterbirds^36^. Although 163 *OR*s were detected, a total of 69 *OR*s were not identified with a full-length and intact ORF (open reading frame) from the draft genomes of the emperor penguin (number of unidentifiable genes: 11), Adelie penguin (15), northern fulmar (9), great cormorant (12), crested ibis (1), little egret (7), Dalmatian pelican (6), and red-throated loon (8) (Fig. 1). It is understandable that the crested ibis has just 1 of the 29 *OR* genes that cannot be identified from its genome, because its genome coverage is the highest (105×). Similarly, the large number (12) of unidentifiable genes in the great cormorant could be attributed to its having the lowest genome coverage (24×) (Fig. 1). However, there are 11 and 15 unidentifiable genes in the emperor penguin and Adelie penguin with high-coverage genomes, respectively, and the numbers of unidentifiable genes in both penguins are even greater than those in three other waterbirds (the northern fulmar, red-throated loon, and Dalmatian pelican) with low-coverage genomes (Fig. 1). This finding suggests that penguins may have lost more functionally conserved *OR*s than other waterbirds. In particular, 11 *OR*s (*OR3-4*, *OR7*, *OR9*, *OR13*, *OR19*, *OR22*, *OR24-27*) were not identified in both penguins (Fig. 1), indicating penguin-specific losses.

### Pseudogenization of *OR* genes in penguins

**Figure 2:**
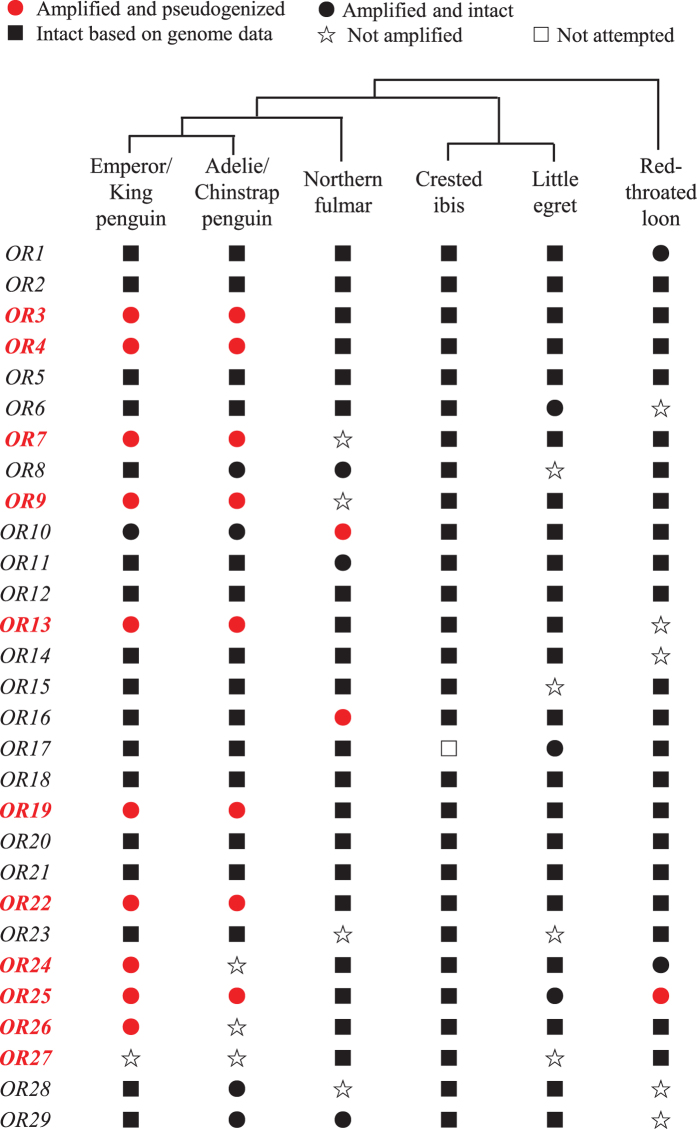
Survey of avian olfactory receptor genes by additional sequencing. In case that *OR* gene sequences are missing from the two penguin genomes, genomic DNAs of their congeneric species (King penguin and Chinstrap penguin) were used to perform amplification and sequencing. The great cormorant and Dalmatian pelican were not included because of the lack of genetic material, but the examined species still represent the four avian orders. Genes that are potentially lost in penguins were highlighted in red.

To examine whether some orthologous *OR*s were specifically lost in penguin lineages, we attempted to sequence all missing data (Fig. 1). In case of missing data from the two penguin genomes, we performed gene sequencing using the genomic DNAs of their respective congeners (King penguin and Chinstrap penguin); the northern fulmar, red-throated loon, and little egret were examined with PCRs; the crested ibis was also included for comparison without additional sequencing, because this bird only missed one *OR* gene from its genome, which appeared to be intact in penguins (Fig. 2). We did not examine the great cormorant and Dalmatian pelican due to the absence of genetic material, although this dataset of six birds still represents the four avian orders^36^. Of note, we did not sequence the two species of penguins with available genomes due to the lack of genomic DNAs, but their congeneric species are still appropriate to infer pseudogenization events in the common ancestor of all penguins by identifying shared frameshifts or interrupting stop codons.

**Figure 3:**
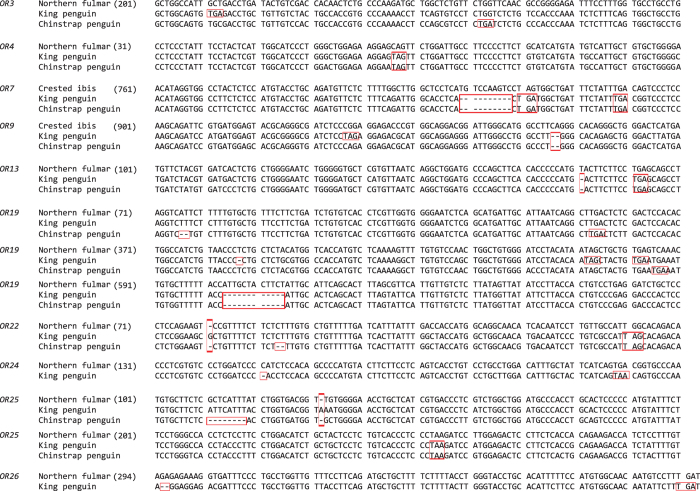
Nucleotide alignments of avian olfactory receptor genes. The first ORF-disrupting mutations and the followed common mutations were boxed. Dashes indicate alignment gaps and numbers in parentheses represent nucleotide positions following the reference sequences from either Northerm fulmar or Crested ibis.

We sequenced 34 *OR* gene segments from the five birds as mentioned earlier (Fig. 2), which ranged from 612 to 964 base pair (bp), with an average of 805 bp (National Center for Biotechnology Information accession numbers: KX171590-KX171621 and KX189196-KX189197). Phylogenetic analysis was used to confirm the orthology of each gene. The newly acquired 34 sequences were aligned with 121 *OR*s identified from draft genomes, which resulted in a total of 155 *OR* sequences for subsequent analysis. We found 134 out of 155 *OR*s to have intact open reading frames (ORFs) (Fig. 2). Among the four non-penguin birds (the northern fulmar, crested ibis, little egret, and red-throated loon), 99 out of 102 *OR*s (99/102 = 97.1%) were identified with intact ORFs (Fig. 2), supporting our assumption that these genes are functionally important across non-penguin waterbirds. By contrast, 36 out of 54 *OR*s (36/54 = 66.7%) were found to be intact in penguins (Fig. 2). Among those pseudogenized *OR*s containing ORF-disrupting mutations such as nonsense mutations and frame-shifting insertions or deletions, 18 were found in penguins; the remaining 3 genes are from non-penguin birds (Fig. 2). In 17 out of the 21 pseudogenized *OR*s, the first nonsense mutations resulted from ORF-disrupting mutations are located near 5′ end of each gene (Fig. 3), which would lead to the loss of multiple transmembrane domains of each protein. The 3 pseudogenes (*OR7* of the king penguin, *OR7* of the chinstrap penguin, and *OR9* of the king penguin) contained the first nonsense mutations located near the 3′ end of each gene (Fig. 3 and Supplementary Fig. S2), which could also result in the loss of the final transmembrane domain of an olfactory receptor^41^. We did not observe a nonsense mutation in the pseudogenized *OR9* of the chinstrap penguin, but we observed a 2-bp deletion shared by both penguins (Fig. 3). This finding suggested that none of the 21 pseudogenes could encode a functional olfactory receptor. Therefore, after examining these functionally important *OR*s common to non-penguin waterbirds, we found that the percentage of nonfunctional *OR*s is significantly greater in penguins (18/54 = 33.3%) than in their closely related non-penguin waterbirds (3/102 = 2.9%) (*p* < 0.0001, Fisher’s exact test), which suggested a penguin-specific reduction of olfactory capability.

Among the 29 orthologous *OR*s, 8 were sequenced from both penguins (the king penguin and chinstrap penguin) (Figs 2 and 3). With an exception of *OR19*, at least one common ORF-disrupting mutation was identified between the two penguins for each orthologous *OR* gene. For example, *OR3* contained one 2-bp deletion and one 2-bp insertion that are shared between the two penguins; *OR4* included one shared nonsense mutation; *OR7* has one shared 10-bp deletion and two common nonsense mutations (Fig. 3 and Supplementary Fig. S2). We did not observe any shared ORF-disrupting mutations in *OR19*, but we identified a relatively large deletion (12-bp) that are common in the two penguins; coupled with multiple nonsense mutations ahead of the 12-bp deletion, this finding suggested that *OR19* was pseudogenized prior to the divergence of the two penguins (Fig. 3). Given that the king penguin and the chinstrap penguin diverged at the origin of the order Sphenisciformes, our genetic evidence strongly suggests that at least 8 functionally important *OR*s in other waterbirds were lost in all penguins, and the relaxation of functional constraints on these olfactory receptor genes occurred predating the divergence of penguins. Because penguins diverged 23 million years ago (Ma) and penguins diverged from their closest relatives (order Procellariiformes) approximately 60 Ma^28^,^36^, the penguin-specific reduction of olfactory capability took place in the common ancestor of penguins between 23 and 60 Ma. In addition, we observed three independent pseudogenizations in non-penguin waterbirds (*OR10* and *OR16* in the northern fulmar, *OR25* in the red-throated loon) (Supplementary Fig. S2).

To understand why penguins could afford to lose some important *OR*s that are common in other waterbirds, we assigned each pseudogenized *OR* into a specific *OR* gene family following a recent study^42^. Among the 29 putative one-to-one orthologous *OR*s (*OR1*-*OR29*), 8 genes (*OR3*, *OR4*, *OR7*, *OR9*, *OR13*, *OR19*, *OR22*, and *OR25*) were found to have common disruptive mutations between the two penguins, suggesting an ancestral pseudogenization in the common ancestor of penguins; 2 genes (*OR24* and *OR26*) were sequenced in one of the two penguins because of the failure of PCRs; 1 gene (*OR27*) was not able to be amplified in both penguins even after trying several primer pairs (Figs 2 and 3). The failure of amplification suggests either severe degeneration or loss of these genes, we thus infer that 11 out of 29 *OR*s were pseudogenized in penguins. After using the *OR* family Assigner^42^, four genes (*OR3*, *OR4*, *OR7*, and *OR9*), three genes (*OR13*, *OR19*, and *OR22*), two genes (*OR24* and *OR25*), and the remaining two genes (OR26 and *OR27*) were assigned into the *OR* gene family 52, family 5, family 10 and family 6, respectively. According to the traditional classification based on sequence similarity, *OR*s are divided into 18 families. Class I families (i.e. *OR* gene family 51–56) are assumed to detect water-borne molecules, whereas Class II families (i.e. *OR* gene family 1–14) are believed to recognize air-borne compounds^42^,^43^,^44^. Consequently, penguins have pseudogenized 4 *OR*s that could function underwater and 7 *OR*s that could smell in the air. In addition, an earlier study suggested that *OR* gene family 5 was associated with the foraging behavior of predatory birds, and that *OR* gene families 6 and 10 were prominent in vocal-learning birds^15^. Therefore, penguins have lost several *OR*s that are important in vocal learner and birds of prey.

## Discussion

Through examination of 8 draft genomes of 2 penguins and 6 non-penguin waterbirds that are closely related to penguins, we identified 29 *OR*s that are putative one-to-one orthologs among all 8 waterbirds by phylogenetic analysis. We next attempted to survey the functionality of the 29 *OR*s by sequence analysis in penguins and their relatives. With the aid of additional sequencing, we found that, of these functionally important *OR*s common to other waterbirds, penguins were found to have a significantly greater percentage of pseudogenized *OR*s than other waterbirds, suggesting a major reduction of olfactory capability in penguins relative to other waterbirds. However, the small number of olfactory receptor genes does not necessarily represent a reduced importance of olfaction in penguins, because olfactory receptors could evolve to recognize more odorants and develop novel receptors, as reported in taste receptors^45^,^46^. Despite this, our genetic evidence suggests that penguins appear to have a less developed sense of smell than most other waterbirds, as a higher number of chemosensory receptor genes allows the evolution of more specialized receptors^45^. Since genetic evidence is indirect, our study awaits a behavioral test to verify the possible reduction of olfaction in penguins.

In support of our genetic evidence, anatomical reduction of the olfactory bulb in penguins was observed as compared to most other waterbirds, which suggested that penguins generally have a reduction of olfactory acuity relative to other waterbirds^33^,^34^,^47^. Fossil evidence revealed that ancient penguins had much larger olfactory bulbs than extant penguins, suggesting that the reduction of olfactory acuity started shortly before the divergence of penguins^48^. However, the reduced olfaction does not suggest that penguins do not use the sense of smell. Behavioral studies have convincingly demonstrated that penguins can use olfaction to locate prey and recognize kins^29^,^30^,^32^. Indeed, our genetic analysis also revealed that penguins still retain many intact *OR*s, suggesting a functional role of olfaction in these birds. In fact, penguins were found to possess even more *OR*s than vocal-learning birds, suggesting that they have a better sense of smell than some other birds^15^.

Why could penguins afford to reduce the reliance on olfaction compared to other waterbirds? Penguins are the only surviving birds that inhabit a secondarily aquatic environment with flightless wing-propelled diving behavior; their reduced reliance on olfaction parallels the reduction in marine mammals that independently occupy a secondarily aquatic niche^11^,^12^,^48^. Indeed, we found penguins to have lost several *OR* genes that encode olfactory receptors detecting air-borne molecules, suggesting that penguins do not need to smell some air-borne molecules in the aquatic environment. Within mammals, sensory tradeoffs were proposed^49^,^50^; the reduction of *OR* genes in trichromatic rather than dichromatic primates has been explained by a trade-off between vision and olfaction^13^. However, this explanation cannot be the case in penguins, because penguins are trichromatic while most other birds are tetrachromatic^27^,^28^. On the other hand, positive selection on phototransduction genes and accelerated evolution of visual opsin genes were detected in penguins, which were associated with aquatic adaptation^28^. Furthermore, the photic adaptation in penguins has also been detected by morphological specializations such as flat corneas and spherical lens^25^,^26^. In addition, unlike other diving waterbirds, penguins spend the entire search, chase and capture underwater, some species of penguins are even able to dive 200 meters in depth and 30 minutes in duration^51^. Therefore, the aquatic specializations for underwater vision in penguins may have rendered their olfaction less important. Other ecological traits may also account for the reduction of olfactory acuity in penguins. For example, since penguins originated in the coldest niche on Earth, the extremely cold temperature of the Antarctic may have influenced the evolution of olfactory perception^52^, a hypothesis awaiting future empirical investigation. Taken together, we found genetic evidence for a possible reduction of reliance on olfaction in penguins, and highlighted the power and necessity of in-depth genetic analysis based on draft genome sequences. Although the loss of three primary tastes in penguins has already been revealed^53^, future studies of other sensory systems in penguins and other waterbirds would provide a better understanding of how penguins could sense and survive in their unique ecological niche.

## Materials and Methods

### Genome data and gene identification

The draft genome sequences of the eight species of waterbirds were retrieved from the Avian Phylogenomics Project ( http://avian.genomics.cn/en/, last accessed March 25, 2015). Vertebrate *OR*s are single-exon genes that encode seven-helix transmembrane proteins^40^. To identify full-length and intact *OR*s from the eight waterbird genomes, we followed a standard protocol as described previously^54^. Briefly, we used full-length OR protein sequences^55^,^56^ as queries to conduct TBLASTN searches^57^ with an e-value cutoff of 1e-5. The best hits were determined with the criteria of the lowest e-value and the longest alignment, and the putative start and stop codons were identified by extending in both 5′ and 3′ directions. All these potential *OR* genes were then compared (BLASTX) back to the NCBI non-redundant database, and those with the best blast hit of a non-OR gene were discarded. Sequences that are longer than 250 amino acids and have no interrupting stop codons or frameshifts were aligned to known *OR* genes, and those with a gap of five or more amino acids within transmembrane domains or other conserved regions were excluded. The remaining sequences were considered to be the full-length and intact *OR* genes, which are used for further analysis.

### Phylogenetic reconstruction and *OR* gene family assignment

Phylogenetic reconstruction was conducted to identify putative one-to-one orthologous genes across the eight birds with draft genomes. A total of 344 complete and intact *OR*s identified from the eight avian genomes were analyzed with a zebrafish *OR* gene (GenBank: NM_001083869) as the outgroup. The 345 full-length *OR*s were translated into protein sequences in MEGA version 6^58^ and were next aligned by MUSCLE^59^ with manual adjustments. The protein sequence alignment was subsequently translated back to nucleotide sequence alignment, which was used to reconstruct phylogenetic trees. Phylogenetic analyses were performed with both Maximum Likelihood (ML) and Bayesian Inference (BI) approaches. The jModelTest2 program^60^ was used to infer the best-fitting substitution model, and the model GTR + I + G was selected. The RAxML version 7.2.6^61^ was used to reconstruct ML trees with bootstrap replicates of 1000. The Bayesian tree was constructed by MrBayes version 3.2^62^ with six Markov chains and six million generations.

Genes identified from the whole genomes were assigned into *OR* gene families using the OR family Assigner, ORA version 1.9^42^. Specifically, we undertook the domain-based hmmscan searches against the HMM (hidden Markov models) database using the program HMMER version 3.1b1^63^. We assigned a given *OR* gene into an *OR* gene family with the lowest e-value produced from the hmmscan searches. The nomenclature of each *OR* gene (Supplementary data set S1) followed the best hit after conducting BLASTN searches against the HORDE database^39^. For convenience, we also named each gene with the order in which they were identified (Supplementary data set S1).

### Taxon sampling and DNA sequencing

Due to the incomplete genome sequencing or poor genome assembly, some putative one-to-one orthologous genes were not identified with a complete and intact ORF (open reading frame) from the draft genomes (Fig. 1). To obtain the missing data, we designed new primers (Supplmentary Table S2) to resequence all missing sequences based on the nucleotide sequence alignments of each gene. The two penguins (i.e. the emperor penguin and the Adelie penguin) with genome sequences are from the two major clades of penguin species tree^35^, the ancestor of both penguins thus represents the common ancestor of all extant penguins. In case that *OR* gene sequences are missing from the penguin genomes, we attempted to amplify from the genomic DNAs of their congeneric species (King penguin, *Aptenodytes patagonicus*; Chinstrap penguin, *Pygoscelis antarctica*) (Fig. 2), which were left from a previous project^53^. The genomic DNAs of the northern fulmar and red-throated loon were from the same project in which we loaned all avian samples from the University of Michigan Museum of Zoology^53^. The muscle tissue of the little egret (Sample ID: IOZ-9748) was obtained from the Institute of Zoology, Chinese Academy of Sciences. We also included the crest ibis in our resequencing dataset but did not conduct the sequencing, because this bird only missed one *OR* gene from its genome, which appeared to be intact in penguins (Fig. 2). As a result, our dataset of resequencing contained six avian species: the king penguin, chinstrap penguin, northern fulmar, red-throated loon, little egret, and crested ibis (Fig. 2), which can still represent the four orders (Sphenisciformes, Procellariiformes, Pelecaniformes, and Gaviiformes)^36^.

Genomic DNA of the little egret was isolated with Qiagen DNeasy kit. Polymerase chain reactions (PCRs) and DNA sequencing were conducted according to procedures previously described^3^,^5^,^6^. Newly generated sequences by PCRs were aligned with their respective orthologous *OR* genes identified from the whole genomes. The phylogenetic tree of each gene was inferred to confirm the orthology relationship using a Bayesian approach as implemented in MrBayes version 3.2 62. Pseudogenes were identified when nonsense and/or frame-shifting mutations were observed in an *OR* sequence. All new sequences have been submitted to GenBank under accession numbers KX171590-KX171621 and KX189196-KX189197.

## Additional Information

**Accession Codes**: DNA sequences: Genbank accessions KX171590-KX171621 and KX189196-KX189197.

**How to cite this article**: Lu, Q. *et al*. Penguins reduced olfactory receptor genes common to other waterbirds. *Sci. Rep.* **6**, 31671; doi: 10.1038/srep31671 (2016).

## Supplementary Material

Supplementary Information
