# Supplementary material for: Penguins reduced olfactory receptor genes common to other waterbirds
Source: Sci Rep. 2016 Aug 16;6:31671. doi: 10.1038/srep31671 (PMC4985648; doi:10.1038/srep31671)
Supplement: Supplementary Information [file srep31671-s1.pdf]

Supplementary Information for

Penguins reduced olfactory receptor genes common to other waterbirds

Authors:

Qin Lu, Kai Wang, Fumin Lei, Dan Yu, and Huabin Zhao*

(*E-mail: huabinzhao@whu.edu.cn)

Affiliation:

Department of Ecology, College of Life Sciences, Wuhan University, Wuhan, China

This PDF file contains:

Table S1 to S2

Figures S1 to S2

Data set S1

Table S1. Species examined in this study. Numbers of intact *OR* genes identified in this study were shown in bold, whereas numbers of *OR* genes identified in Khan et al. (2015) were given in parentheses. The classification follows Jarvis et al. (2014).

**Classification (Order)**	**Common name**	**Scientific name**	**Number of intact *OR* genes identified from genomes**
**Sphenisciformes**	Emperor penguin	*Aptenodytes forsteri*	**32** (29)
	King penguin	*Aptenodytes patagonicus*	No genome data
	Adelie penguin	*Pygoscelis adeliae*	**26** (21)
	Chinstrap penguin	*Pygoscelis antarctica*	No genome data
**Procellariiformes**	Northern fulmar	*Fulmaris glacialis*	**33** (29)
**Pelecaniformes**	Great cormorant	*Phalacrocorax carbo*	**20** (19)
	Crested ibis	*Nipponia nippon*	**47** (38)
	Little egret	*Egretta garzetta*	**106** (101)
	Dalmatian pelican	*Pelecanus crispus*	**36** (31)
**Gaviiformes**	Red-throated loon	*Gavia stellata*	**44** (40)

Table S2. Primer information used in this study.

Amplified gene	Primer name	Primer Sequence (5'-3')*	Primer pair**
OR1	A54_1F	GTGTACATCTTGGCGGTCAC	forward
	A54_1R	ACACCTTGAGCCTCTCCTTC	reverse
OR3	OR3_1F	CCAACCGATCCAACACCAAC	forward
	OR3_1R	TCTCTCACCATCTGACCTGTC	reverse
OR4	OR4_2F	ACATCTGGATCTCTGTCCCC	forward
	OR4_2R	GCAGAAGAGGCGGACGAC	reverse
OR6	OR3_1F	CCAACCGATCCAACACCAAC	forward
	OR3_1R	TCTCTCACCATCTGACCTGTC	reverse
OR7	OR2_2F	AATTCGCATCCCACCAACAC	forward
	OR2_2R	GCATCTCTGAACGTACGCTG	reverse
OR8	OR8_1F	GCAACGCTTCCACTAAAGTCT	forward
	OR8_1R	CATGACGTGGACATCAACGG	reverse
	OR8_2F	GGGTTTCCAGCTTTCCAGGA	forward
	OR8_2R	TGATGGTGTTTAACAAGGGAGT	reverse
OR9	OR9_1F	CTCCCACCCTCCCTATTTCC	forward
	OR9_2R	TCACTGGAAGAGGAGGACC	reverse
OR10	A11_A12_1F	GGGATCCCACCCCACGTTC	forward
	A11_A12_1R	AGGCTTCGTTTCACTCCTCTTG	reverse
OR11	OR11_1F	CCTTTCCTCCCAGCCAAGG	forward
	OR11_1R	GCACGCTTCATGTCCTTGTT	reverse
OR13	OR13_1F	AGATAAGATGCAGAGGGTCAAC	forward
	OR13_1R	TTTAGCAGGGGAGTGAGGATTC	reverse
OR16	OR16_2F	CCAGCCCTTCCTCTTTGTTC	forward
	OR16_1R	GGTGACGATGCAGTACAGTG	reverse
OR17	A51_1F	GTTAATCAGGGCCAGCCCTCAGC	forward
	A51_1R	CTCCTCAAAGCCTCCTTCACCTCC	reverse
OR19	A24_25_1F	CTTGATCCTGCTTGGAAACATTAC	forward
	A24_25_1R	TTCCTGAGTGAGTAGATGTATGGGTTA	reverse
OR22	A50_1F	ACCTCCTGGCTGTGATGGC	forward
	A50_1R	CCTTGTTCCTCAGGCTGTAGA	reverse
OR24	OR24_1F	GGTGACCCAGTTCATCCTCC	forward
	OR24_1R	TTTTCGAAGTGCATCCTTGACC	reverse
OR25	OR25_2F	AATGCCTACAGAGCACAGGT	forward
	OR25_2R	TCTCAAGCTATAGATGATGGGGT	reverse
	A27_1F	TAATGCCTACAGAGCACAGGTGACC	forward
	A27_1R	GCCCCTTTGACCTCACTGTTTCTC	reverse
OR26	OR26_1F	TGGTCAGAAGAAACAAGACAACT	forward
	OR26_1R	CTAGGGAGTAGCTAGAACTGGG	reverse
OR28	OR28_1F	CCCAGTGCTGAATTTGTTCTCT	forward
	OR28_1R	TTGTTCCTCAGGCTGTAGATGA	reverse
OR29	OR29_2F	AACCAAACTGTTATCAATGAGTTCA	forward
	OR29_2R	TTAGAGGGTTCAGTGCTGGA	reverse
	OR29_1F	TCCAGACCTTACTTTACCTGGT	forward
	OR29_1R	GCTTCTGCCTATGATCTTTCTCA	reverse

*When the direct sequencing did not work, PCR products were cloned and sequenced with the M13 primer pair (M13-47: 5'-CGCCAGGGTTTTCCCAGTCACGAC-3' and M13-48: 5'-GAGCGGATAACAATTTCA CACAGG-3').
** Each forward primer was attempted to pair with each reverse primer whenver needed.

Figure Legends

Figure S1. Evolutionary relationships of 344 complete and intact *OR*s identified from the eight avian genomes. Phylogenetic analyses were performed with both Maximum Likelihood (ML) and Bayesian Inference (BI) approaches, which yielded similar topologies. A total of 293 codons were used and the tree was rooted with the zebrafish *OR135-1* gene (GenBank: NM_001083869). For convenience, we named *OR*s from each bird with the order in which they were identified **(Supplementary data set S1)**. The 29 putative one-to-one orthologs were identified as shown in red. We named each of the 29 genes numerically in the order of appearance on the phylogenetic tree (above the branch), or followed the HORDE database (Olender et al. 2004) to name the 29 genes (below the branch). Numbers (shown in blue) at the nodes of the 29 genes are the ML bootstrap values/Bayesian posterior probabilities as percentages, while numbers (shown in black) at the remaining nodes are the the ML bootstrap values.

Figure S2. Alignments of avian olfactory receptor genes showing shared ORF-disrupting mutations in the two penguins and the first ORF-disrupting mutations in non-penguin birds. Dashes indicate alignment gaps and numbers in parentheses represent nucleotide positions following the reference sequences from either Northerm fulmar or Emperor penguin.

Figure S1

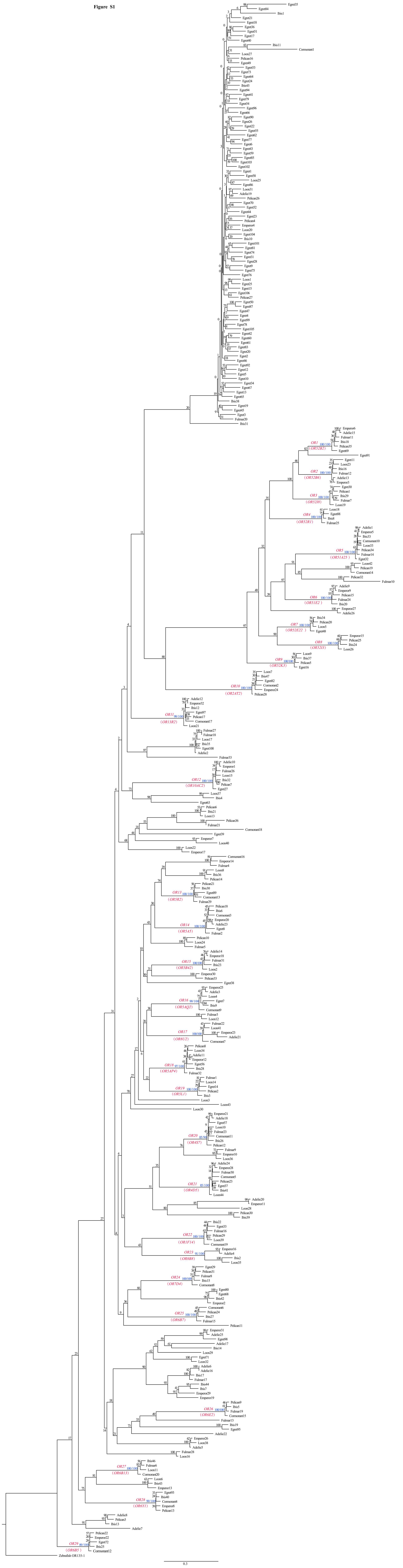

Figure S2

```
OR3    Northern fulmar (601)   GGCCTGGCAG TGCCTGTTGC A--ACAGCAG TGGTAGATGT TGTACTCATT GCTGTCTCCT ATGTCTTAAT TCTTCTGGCA -TTATTCAGA CTCCCTTCCA
       King penguin            GGGGTAGC-- TGCTGGCTTC CTTTCAGCCG GCTTGGATGC CATCTCCATC ACCATCTCCT ACGCGTTGA- TCTTCAGGTG CCTCTGGGGA CTGCCGTCCC
       Chinstrap penguin       GGGGTAGC-- TGCTGGCTTC CTTTCAGCCG GCTTGGATGC CATCTCCATC ACCATCTCCT ATGCGCTGA- TCTTCAGGTG CCTCTGGGGA CTGCCGTCCC

OR10   Emperor penguin (301)   CAGATGTACA GTTTTCATGG GTTGACAGTA ACCGAGGCGC TTCTCCTAGC AGTCGTGGCT TACGACCGCT ACGAAGCTAT CTGCAACCCC CTCTGTTGCC
       Northern fulmar         CAGATGTACA GTTTTCACGG GTTGACAGTA ACTGAGGTGC TTCTCCTGGT GGTCATGGCT TATGACCGCT ACGAAGC-AT CTGCAACCCC CTCCGTTATT

       Emperor penguin (401)   CGGCCAAGAT GACAAGAAGA GTGAACATCC ATCTGGCAGC GAGCGCCTGG ATCACTGCGC TGCTAATACC CGTTCCCATC ATCACGCAGA CCTCTCAGCT
       Northern fulmar         CAGCCAAGAT GACAAGAAGA GTGAACATCC AGCTGGCAGT GAGCACCT-- ---------- ---------- ---------C ATCATGCAGA CCTCTCAGCT

OR16   Emperor penguin (271)   TTGGTGGAAA AGAAAACCGT TTCTTACTCC GCCTGCTTTG CCCAGCACTT CTTTTTCCTT GTGTTTGTGA CCACGGAGGT GCTCTTGCTG GCTGTGATGG
       Northern fulmar         TTGCTGGAAA AGAAAACCAT TTCTTAC-CC GCCTGCTGCG CCCAGCATTT CTGTTCCCTT GTGTTTGTGA CCGCAGAGGT GCTCTCGCTG GCTGTGGTGG

OR25   Northern fulmar (421)   ATGAG----C AAGAAGA--- ---------- ------TGTG CCACTGTTTA GTGGTTGTTT CGTGGCTGAG TCGTCTCTGA TCCAGGCCTT CCTCACAGCC
       Red-throated loon       ATGAGTGCTC ATGTAGAGCA CATCCCCATG AGCGCTTATG TCACTGCTCA GTGGCTGTTT CATGGCTGAG TCATCTCTGA TCCAGGCCTT CCTCACAGCC
```

Supplementary Data set S1. Complete and intact olfactory receptor genes identified from the eight avian genomes. The nomenclature of each *OR* gene followed the best hit after conducting BLASTN searches against the HORDE database (Olender et al. 2004). For convenience, we also named each gene with the order in which they were identified as shown in parentheses.

>Emperor_OR10R2 (Emperor1)
ATGGAGTTCCTCCTGCTTGGCTTCTCTGAGCTGCTCTGTCTGCGGGTCCTCCTCTTCTTT
ATCTTTCTCATTGTCCATTTGGTCACATTGGCAGGGAATGTGATGATCTTCATGGCGGTG
GTTATGGAGCCTTCCCGTCCTCCCATGCTTTTCTTCCTCTGTCAGCTCTCTGTCATCGAG
CTCTGCTGTACTTTAGTCATTGTCCCTAAGGCACTCCTCAGCCTGATAGTGGTGGATGG
CAGCACCATTTCTTTCATAGGCTGTGCTGCACAGATGCACCTTTTTGTGACACTCGGTG
GGGCTGAATGCTTCCTCCTGGTAGCCATGTCGTACGACCGTTACGTTGCCATCTGTCAG
CCACTTCACTACGTAGCTGTGATGAGTGAGGGGCTCTGCCTCAGGCTGGCTGTGGCAT
GCTGCCTGGGAGGCTTTGCTGTTGCTCTGGGGTTGACGGTGGCTGTTTTCCGCTTACCT
TTCTGCCAGTCGCATCATATCAACCACTTCTTCTGTGATGTCCCTGCTGTGCTGCACCTG
GCCTGTACACAGAGTTACACCCCTGAGCTGCCCTTGCTGGCTGCCAGTGTGCTCCTCC
TGCTGCTCCCCTTCCTCCTAATCCTGACCTCGTATGTTTGCATTGCTGCTGCTTTGCTAC
GTGTCACCTCCTCTGCAGGAAGGGGCAAGGCCTTTTTCACTTGCATTTCACACTTGTCC
ATCACCTTGCTACACTATGGATGTGCCACCTTCATGTACATTCGCCCTAAGTCCAGTTAC
TCGCCAGCTCGGGACAAGATGGTGTCTCTGGTCTACACCAACATTACTCCATTACTGTA
TCCCCTCATTTATAGCCTGAGGAACGAGGAAATCAGAGGGGTCATCATGAAAATGTTG
AGGAGGAAGAAAATAGCTCAGCTGAACTGGGATACTATCAGAGCTGTGATGTGTGTGT
GTGGTAAATTTTAG
>Emperor_OR12D2 (Emperor2)
ATGCTGAACCAGACAGAGGTCAGTGAGTTCATCCTTTTGGGCCTCACCGACATCCAGG
GGCTGCAGCACTTTTTCTTCATCTCCTTTCTGTTGCTCTACTTGACCAGTCTTCTGGGAA
ATGGTGCCATTGTGACCATGGTGATATCTGAGCCCCGGCTCCACACACCGATGTACTTC
TTCCTGGGGAACCTGTCCTGCCTGGACATTTTCTACTCCACAGTCACTGTTCCCAAGAT
GTTGACTGGCTTCCTCTTTGGGCATCAGCCCATCTCTTTTGGTGGGTGCTTGGCCCAGC
TCCACTTCTTCCACCTCCTGGGCAGCACTGAGGCTGTGCTCCTGGCCACCATGGCCTAC
GACCGCTGTGTGGCCGTTTGCAACCCTTTGCGCTACGCCCTTGTCATGAGCCCACGGA
CTTGTCTGCTGCTGGCTGTGGCCAGCTGGTCCACTGGTTTTGTACATGCCATGACGCAC
TCAGTCATGACCTCTCAACTGAGATTCTGTGGCCACAACCACATCCATCACTTCTTCTG
TGACATCAAGCCACTGTTGAATTTGGCTTGCAGTAGTACCAGCCTCAACATGACCCTCC
TCAATGTCGTCACCACTTCTATTGTTCTAGGCCCCTTCACTCTCATAGTCCTCTCCTACC
TCTACATCATCTCCTTCATCTTCCAGAGAGTCCGGTCCCAGGAAGAAAGATGGACGCC
CTTCTGCACCTGTGCCGCCCACCTCACCACTGTGGCACTGTTTTACATACCAGTGCTCT
TCAATTATACACCACCCTCCTCAGGAAGCTCCCCTAAAAGGGATGTGCAAGTGTCTCTC
CTGCACAGTGCTGTCACCCCAGCTCTGAACCCCTTGATCTACACTCTTAGGAACCAGG
AGGTGAGATCTGCCCTGAAAAAAACGTTAGGGAGAAAACTCTTCCCTGCAGGAAAGT
GA
>Emperor_OR52B2 (Emperor3)
ATGGCGGCTCTCAATCAAACCAGCTTACAGCCTGCCTCCTTCCTTCTGCTGGGCATGGC
AGGCCTGGAGGACCTGCACACCTGGCTCTCCATCCCATTCTGCCTGATGTACATCGCGG

CGCTCCTTGGCAACTTCATCCTCTTATTTGTCATTGTGACAGAGCGAAGCCTCCACGAG
CCTATTTACCTCTTCCTGGCCATGTTAGCCATGGCAGATCTCGTATTATCCTCCTCCACA
GTGCCCAAAGCCCTGAGCGTATTCTGGTCCCTTTCCAAGGAGATTTCTTTCCATGCCTG
CCTTACCCAGATGTTCTTCACACACCTGAGCTTCATTGCAGAGTCAACCATTCTGCTGG
CCATGGCATTTGACCGGTACGTGGCCATCTGCAACCCCCTGCGATATGCCACAGTGTTC
ACGCACTCAGTGATAGCCAGGATAGGGCTGGCTGCAATAGCCAGGAGCTTTTGTGTGA
TGTTCCCAACAATATTCCTCCTTCAGAGGCTGCCATACTGCAGACACAGCATCATGCCG
CACACCTACTGCGAGCACATGGGCATCGCACGGCTGGCCTGTGCCGACATATCTGTTAA
CATCTGGTACGGCCTTGCCACCACCCTTCTGTCCCCAGGTGTGGACGTTGTGCTCATTG
GGGTATCGTACGTCCTCATCCTCCGGGCTGTCTTCAGGCTCTCATCCAAGGATGCCCAG
CTCAAGGCAGTTGGCACCTGCAGCTCTCATGCCTGCGTTATATTAATTTTCTACACACC
AGCATTTTTCTCATTTTTCACTCATCGGTTTGGCCGCAACATCCCCCACCATGTTCACAT
CCTGTTGGCCAATCTCTATGTGCTCTTGCCACCTATGCTAAACCCCATCGTCTACACTAT
GAAAAACAAACACATTCGAGAAAAGGTGTCCCAAGTACTCTTCAGGACTGGGCAAGT
GCGGTGA
>Emperor_OR14J1 (Emperor4)
ATGTCCAACGGCAGCTCCATCACTGAGTTCCTCCTCCTGGCATTCGCAGACACGCGGG
AGCTGCAGCTCTTGACCTTCTGGCTCTTCCTGGGCATCTACCTGGCTGCCCTCCTGGGC
AATGGCATCATCATCACCACCATAGCCTTCGACCACCGCCTCCACACCCCCATGTACTT
CTTCCTCCTCAACCTCGCCCTCCTCGACCTGGGCTCCATCTCCACCACTCTCCCCAAAG
CCATGGCCAATTCCCTCTGGGAGAACAGGGCCATCTCCTACTTGGGTTGTGCTGCCCA
GGTCTTTTTCTTTCTCTTCTTGATGACAGCAGAGTGTTCTCTCCTCACTGTCATGGCCTA
CGACCGCTACGTTGCCATCTGCAAACCCCTGCACTACGGGACCCTGCTGGGCAGCAGA
GCTTGTGCCCACATGGCAGCAGCTGCCTGGGGCAGTGGGTTTCTCCATGCTGTGCTGC
ACACTGCCAGTACATTTTCACTCCCACTCTGTCAAGGCAATGCCCTGGACCAGTTCTTC
TGTGAAATCCCCCAGATCTTCAAGCTCTCCTGCTCAGACTCAGACTACCTCGGGGAAG
CTGGGTTTCTTGTGCTTAGTGCCTGTTTAATTTTGGGGTGTTTTGTTTTCATTGTGCTGT
CCTACGTGCAGATCTTCAGAGCCGTGCTGAGGATCCCCTCCGAGCTGGGACGGCACAA
AGCCTTTTCCACGTGCCCCCCTCACCTGGCTGTTGTCTCCCTGTTTATCAGCAGTGGCA
TATTTGCCTACCTGAAGCCCCCCTCCATCTCCTCCCCAGTTCTAAATCTGGCACTATCAT
TTCTGTACTCGGTGGTGCCTCCAGCAGTGAACCCCCTCATCTACAGCATGAGGAATAAG
GAGCTCAAGGATTCCCTGAAGAGACTTATCACTGGATGTTTTTAA
>Emperor_OR51G2 (Emperor5)
ATGGAGCATGACTCACATACCACGTGGGAATTCAATGGCTCCTTCTATCAGCCTCCAGC
TTTCCTCATGATGGGCATCCCAGGCCTGGAAGCCCTTCACCACTGGATCTCCATCCCTT
TCTGTGCACTGTACCTTATTGCTCTCTTGGGAAACTGCATGATCCTATTCATCATAAAGA
AGACCCAAAGTCTTCACGAACCAATGTATTACTTCCTCTCCATGCTGGCAGTCACTGAC
CTGGGCTTGGTTCTATGTACGCTGCCTACTACTCTGGGCATTTTTTGGTTTAATATGCGA
AGGATTGGGTTTGATGCTTGCCTCACTCAGATGTATTTCATCCACATACTGTCCTTCATT
GAATCCTCTGTGCTCCTGGCAATGGCATTTGACCGCTTCATTGCCATCTCCCATCCACTG
AGACACCCATCCATACTGACCAAGACGACTGTCATAAAAATAGGTCTGGCAATTATATT
GAGAGGTATGGTCTCCCTCCTTCCCATACCCTTCTTGCTCAAGAGACTAACCTATTGCA
GGAAGACTGAGCTTTCTCATTCTTTTTGTTTCCATCCTGATATCATGAACCTAGCATGTG
CAGATATAAAAGTCAATGTCTTCTATGGTATGATTATTCTCTTATCAACAGTGGGGATGG

ACTTCATCTTCATTGTGCTGTCCTACATCCTGATCATTAAAACTGTTATCAGCCTTGCAA
CCAAGGAGGAGTGTCTCAAGGCTCTGAATACATGTGTCTCCCACATCTGTGCTGTTCTA
GTGTTCTTCATCCCAATGATCGGACTGTCCATGATCCATCGCTTTGGAAAGAACGTTCC
TCCTCTGGTTAACACTTTGGTGGCCTACACCTACCTTATAATTCCCCCAGCTCTCAACCC
CATTATCTACAGCATAAAATCCAGCCACATCCGTGAGGCTTTGCTCAGGGCACTGTGGA
GGAAGAGTGAACTGGTAGCTTCCTCCACCAGTTCTACCAGAAAGTGCTAG
>Emperor_OR52B2 (Emperor6)
ATGTATGAGCTCAATGAAAGCAGCTTCGATCCTATCACCTTCGTCCTGACGGGCATCCC
GGGCATGGAGTCGTCTCACATCTGGATCTCCGTCCCCTTCTGTCTGATGTACATCACTG
CGGTGTTTGGCAACTCTGTCCTCCTCTTTGTCATCATCACGGACAGGAGCCTCCACGA
GCCCATGTACCTCTTCCTTGCTATGCTGGCCGTTGCTGACCTTATGCTTTCGACCACGAC
GGTGCCCAAAATGCTGGCTATCTTCTGGTTCAGTGCCAGGGAAATTTCCTTCGATGCCT
GCATTACACAGATGTTCTTCACCCATTTCAGCTTCATTGTGGAATCATCCGTTCTGCTGG
CGATGGCATTTGACCGGTACGTGGCCGTCTGCGACCCGCTGCGGTACTCTTCAACCTTA
ACCCCCTCGGTGATTGGGAAAATAGCTGTGACTGCCGTTGTCCGGGGGTTCTGCATCAT
GTTCCCACCCATCTTCCTCCTGAAGCGGCTGCCGTACTGTGGACACAACGTCATGCCCC
ACACCTACTGTGAGCACATGGGCATCGCCCGCCTGGCCTGCGCCGACATAAAAGCCAA
TGTCTGGTACGGGCTGACAACAGCTCTTCTCTCCTCTGGCCTGGACGTCGTGCTCATCA
CTGTGTCTTACGCTCTGATCCTCAGGACGGTCTTTCGACTCCCGTCCCCGGAGGCCCGC
CTCAAAACCCTGAGCACCTGTGGCTCCCACCTCTGCGTGATCCTCATGTTCTACATGCC
TGCCTTTTTCTCCTTTCTCACGCATCGGTTTGGCCACCAAATCCCCAGTCACGTTCACA
TCCTCCTGGCCAACCTCTATGTCGTGGTCCCGCCGATGCTCAACCCCATTGTGTACGGG
GTGAGGACAAGGCAAATCCGGGACCGCGTCGTCCGCCTCTTCTGCCCCACGGGGGAG
TGCCCCTGCCCCAGCTGGGGGAGCAGGTGCTGA
>Emperor_OR10A4 (Emperor7)
ATGACCAGCTGGAACCACACAGTGGTGACCTATTTCGAATTTTTACCTTTCTCCAGCAT
TCCAGAGATCCAAGGCTCTCTCTTTTGTCTGGTATTGCTCATGTACCTCAGTACTTTGGT
GGGAAACATCCTCATCATTATGATCACTATGACAGATGCTGCCCTTCAGTCCCCCATGTA
TTTTTTCCTCAAGAACTTGTCCTTCCTGGAGATTGGCTACACTACATCCACAATCCCCA
AGATGCTGGTGAACTTCCTCACAAAAAGGAAAGGCATATCCTTTCTGGGCTGTGCCAC
ACAGATGTATGCCTTCTCCCTCTTAGGGATCACAGAATGTTGTCTTCTGGCTGCCATGG
CCTATGATCGCTATGTGGCCATATGCCACCCCCTGCGCTACACGACCGTGATGAGCTGG
AACATGTGCTTCCTGCTTTCAGCTATATCTTGGCTTACTGGGGTCTTGGTGGCCTTAGAG
CAGACAACTTTCATCTTTACCCTTCCATATTGTGGGCCTAACAGGATCAATCATTTCTTC
TGTGACCTGCTGCCTCTGCTGAAGCTGGCTTGTGTGGACACCTACAAGAATGAAATCA
TCACCTACATAATAGCTGTCCTCTGTGTCATGGTCCCCTTCTTACTCATAGTTGTGTCATA
TGTCCGGATTCTCCACACCATCCTCAAGATGCCATCAGCTGGGGGCAAGAGAAAAACG
TTCTCTACCTGCTCATCTCATCTGTTCGTGGTCACTCTGTTTTACGGATCTGGCATTGTG
ACCTACTTGAGGCCCAAAGCTTTTTATTCAAGCAGCAGTAACAAACTGCTTTCTCTCTC
TTACACACTGATGTCTCCAATGATGAACCCCTTGATTTACAGCTTGAGGAACAAAGAG
GTGAAACAAGCCTTGAAAAGACTGATAGCCAAAAATACAAATACGTGA
>Emperor_OR6Y1 (Emperor8)
ATGTATTTCATTCTCCTGGGTTTTCCCACCACTGCTGAACTGCAGCTGCTCCTCTTCTCT
GGTTTACTTCTGGCTTATTTATTAACTGTGTTGGAAAACTTCCTTATCATTCTCATCATCC

GAACTAACCACAGTCTGAAAAAACCCATGTATTTCTTCCTAGGAAATCTGTCTTTCTTA
GAGATCTGGTATGTTTCTGCCATTGAGCCAAAGATGTTCATCGATTTCTTCTCTCAAGAC
AAACATATCTCATTCCAGGGGTGCATGACCCAGTTGTATTTCTTTGTGACTTTTGTTTGT
ACTGAGTATATTCTGTTAGCTCTTATGGCCTATGGCCGTTTCTTGGCCATATGCAAACCT
TTCCAATATTCACTCATCATGAATCATCAGTTCTGTGTTCAGCTGACAGCTGGCTGTTGG
ATATGTGTTTTGATCACTTCTTCCATCGAGCTGAGCTTTATAGCCCAGCTCTCATTCTGT
GATGTAGACAAAATCAATCACTATTTCTGTGATATTTCACCCCTACTGAATATATCCTGC
AGCGATTCCTCTGCGGCTGAGCTAGTGGACTTCATCTTGGTTCTGATCGTCATCATGGT
GCCTCTGTGTACTGTGGTCACGTCCTATATTTGCATCATATTCACTGTGTTGAAGATCCC
TTCTTCTCAGGGGAGGCAAAAGGCCTTTTCCACCTGCAGCTCCCACTTGACTGTAGTG
ATATTGTTCTACTCTACCACTCTTTTCACTTATGCCCACCCTAAGGTCATGTATACCTACA
GTGCTAACAAGTTGGTATCAGTCTTGTATACGATAGTTGTGCCACTTCGGAGTCCTCTCA
TATATTGTCTTAGAAACAAAGAAGTCAGGTTTGCCCTGAGGAAGACCTTTACTTGCAC
GAGACACACCTAA
>Emperor_OR51E2 (Emperor9)
ATGCCCTTCCCCAACAGGTCTGACCTCAGCCCATCCTCCTTCATCTTGGCCAGCATCCC
GGGGCTGGAGGCCGCCCATTTCTGGATGGCGATCCTTCTGTGCTCCATGTACATCTTGG
CAGTCACAGGCAACTGCGCGGTGCTGTTCATTGTGAAGACGGAGCCCAGCCTGCACG
CTCCCATGTACTTCTTTCTCTGCATGCTGGCTGCCATCGACCTGGCCTTGTCCACGTCCA
CGGTGCCACGTGTTCTCGCCTTCTACTGGTTCAACACCAGGGAGATCAGCTTCGGCGC
TTGCCTTGTCCAGATGTTCCTCATCCACACCCTCTCAGCCATCGAGTCCACTGTCCTCC
TGGCCATGGCCGTGGACCGGTACGTGGCCATCTGCCACCCGCTGAGACACGCTGCCAT
CCTCACCAACGCTGTGACAGCGAAAATAGGGCTGGTAGCCATGGCCAGGGGCGTTCTT
TTCTTCCTGCCTTTGCCTTTGCTCCTCCTGCCCCTTCCATTCTGCAGCTCCCGGGTGCTG
TCACACTCCTTCTGCCTGCACCAGGATGTGATGAACCTGGCCTGCGCCAACACCACCC
CCAGTGTGGTGTATGGCCTCACCGCCATCCTGCTGGTCATGGGGCTGGATGCCATCCTC
ATCTGCCTCTCCTACGTCCTGATCCTCAAGGCTGTCTTGCGGCTAGCATCGTGGAAGGA
GAGGCTCAAGGTGTTCAGCACCTGCGTTGCCCATATCTGTGTGGTCCTAGCCTTCTACG
TGCCCCTGATTGGGCTGTCCATGGTGCACAGGTTTGGCAAAGATCTGGCTCCATTGGTC
CATATCATCATGGGGAACGTCTACATCCTGGTGCCTGCTGTGCTCAACCCCATCATCTAT
GGGGTGAGGACCAAACAGATACAGAGGAGGATCCTGAATTTAATTCATATATACAATGA
CAGAACTGCCCAGTGA
>Emperor_OR4S2 (Emperor10)
ATGGTGTCTATGGAAAACAAAAACAATGTGACGGAATTCATCCTCCGTGGACTAACAC
AAGATAAGACAGTAGCAAAAGTGTGCTTCTCATTATTCTTAGTCTTCTATGCCACTACCA
TTCTTGGAAACCTGCTTATCATCATTATTATAAAGACAAGTGAACAGCTGAACTCTCCC
ATGTACTTCTTCCTGAGCTACTTATCTTTTGTAGACATCAGTTACTCCACTGTCGCAGCT
CCCAAACTCATTTATGACCTTCTTGTTGAGAAGAAGACCATCTCTTTTGTGGGCTGCAT
AGCTCAACTGTTTGCGGGCCATTTCTTCGGGTGCACTGAGATCTTCCTTCTCACAGTGA
TGGCCTATGATCGTTGCGTTGCTATATGCAAGCCGCTTCATTACACAAATACTGTGAACA
AGCATGTCTGTGGCTGGCTGGTGGCAGCTTCTTGGGTGGGAGGCTTTGTACACTCAGT
GGTGCAGACCCTGCAGGCCATTCAGCTACCATTTTGTGGGCCCAATGAGATCGACCAC
TATTGCTGTGATGTTCACCCTTTACTGAAGCTGGCCTGCACTGACACCTACATCACTGG
TGTCATTGTGGCTGCCAATAGTGGTGTGATTTCCCTGAGCTGTTTTGTTGTTCTTATTGT

GTCCTATGCTGTTATCTTGGTTTCTCTGAGGAGACGCTCTTCCGAAGGGCGTCTCAAGG
CTCTCTATACCTGCACTTACCACATCACTGTTGTAGTTCTGTTCTTTGGGCCGTGCATTT
TCATCTATATGCGCCCTTCCACCACCTTCTCAGCAGACAAGACGGTGTCTGTGTTCTAC
ACCATCATCACGCCTATGCTCAATCCCTTGATCTACACCCTCCGAAATGAAGAGGTGAA
AAATGCCATGAAAAAGTTGTGGAGCAGAAAAGTGAAGAGGAGTGAGAAATGA
>Emperor_OR4D1 (Emperor11)
ATGGAGCCACAGAATGTAACCAATCCTGTGACGGAATTTGTGCTCCTGGGCTTCAACT
ACAGTCTTAAGATTCAGCAATTCCTCTTCACGGTCTTCTTCATTGTCTACCTGATGACCT
GCTTGGGAAACATCACCATCCTCACCACTGTTATCACTGACTACCACTTGCACATACCC
ATGTACTTCTTCTTGGCCAACTTAGAGTTCACAGATATCACCGAATCATCAGTAAATACT
CCCATTCTATTGTCAGGTTTCCTCTCCCAGCACAAAACTGTTCCATTCAAGGAGTGCAT
CTTTCATATGTTTGTCTTCCACTTCATTGGTGGTGCTCATATCTTCCTTCTTGCAGTGATG
GCAGCTGATCGGTATGTAGCTGTCCATAAGCCCTTGCAGTACTTGACAATAATGAACCG
TGAGGTGTGTTTAGGCCTAGTGACAGGGTCATGGGCAGGTGGATTCATTCACTCTGCA
ACACAGATTGCTCTGCTTCTCCCTTTACCTTACTGTGGTCCTAACACCCTGGACAATTTC
CACTGTGATGTCCCACAAGTACTGAAAGTGGCCTGCATTGACACCTACCAGACAGAGC
TGCTGATGGTCTCAAATGGTGGACTGCTCCTCATAGTAATTTTTGTCTTGCTGCTCATTT
CATACACTGTCATTTTAGTCAAGATAAGGAGGCAAGTCACCAGAGGAACGCACAAAGC
TTTGTCTACCTGCATAGCCCAGATAATGGCAATAAGCATAACATTCATTCCAGGAATATT
CATCTATGCTCGGCCCTTCAAGACATTTGAACTGGACAAAGTGGCCTCCATCTTCTTCA
CTGTGCTTGTTCCAGTGCTGAATCCCATGGTCTACACCCTGAGAAATACCGAAATGAAA
AAGGCCATCAGAAGACTTGTTTCTAGGGTTCTGTTCTCAGGAGAAAAGGGAATAGCTT
AA
>Emperor_OR5AR1 (Emperor12)
ATGGCGGAGCAGAATCACACCTCAGTGGCAGAGTTCATTCTCGAGGGCCTGAGTGACC
AAGCGGAGATGAAGGCACCCCTCTTTGTGCTGTTCCTGCTCATCTACACCGTCACCCTT
TTGGGCAACGTGGGGATAATCGTAGTCATCCGAGGTGACCCACGACTCCATACATCCAT
GTACTTCTTCCTTGGCAGCCTCTCCGTTGTTGACATCTGCTTCTCCTCTGTGATTGCCCC
CAGGACCTTGGTGAGCTTCCTATCGGAGAGGAAGACTATTTCCTTCGTTGGCTGCATGG
GCCAAGCCGCCTTCTACATCGTCTTCGTGACGACTGAGTGTTTCCTGCTGGCCGTCATG
GCGTATGACCGGTACGTGGCCATCTGTAACCCCCTGCTCTATTCCTCTGTTATGACTCGG
AGGTTGTGCATGTGGCTGCTGGTGGGGTCCTACACTGGGGGGGTCCTGACCTCCATCA
TACAGATGACCTTCATCATTAGGCTGCCCTTCTGCAGCTCCAATGTCATCAACCACTTCT
TCTGTGACGTTCCTCCCCTCCTGGCTCTGTCCTGTGCCAGCACCTACATCAACGAGATG
ATCCTCTTCTCCTTGGCTGGCGTCATTGAGCTCAGCACCATCTCCACCATCCTGGTCTC
CTACACTTTCATCTCCTTTGCCATCCTGAGGATCCGCTCAGCTGAAGGCAGGCAAAAA
GCCTTCTCCACCTGCGCGTCCCACCTGACAGCAGTGACCATGTTGTACGGGACGACAA
TCTTCATGTATTTACGCCCCAGCTCTAGTTACTCCCTGAACACTGACAAAGTGGTCTCC
GTCTTCTACACGGTGGTCATCCCAATGCTGAACCCCCTCATCTACAGCCTGAGGAACCA
GGAGGTGAAGGACGCTCTGAGGAGAACAGCAGAAAGAATCACAGTCAGGCTCTGA
>Emperor_OR6A2 (Emperor13)
ATGAGAGAGAGAGAGAGAGAGATAAAGGTATTCTAATTATTATAATCACTAATAATGTCATT
GATGTCATTGTCTTTACAGAGGTAGATGGAAGACATGGAGGTGAGCAATCACAGCATG
GTCAAAGAATTAATTCTCCTTGGATTTCCCATTGCTCTCGCTTGAAGATTCTCTTCTTTG

CAATATTTCTCATTGCATATCTCTTAGTTTTAGCAGAAAATATTATAATCATCCTGACTGT
CTGGACAAACTATAACCTCCACTCCCCCATGTATTTCTTTCTGAGTAATTTGTCCTTCCT
GGAGATCTGGTATGTGACAGTCACACTCCCCAAGGCAATGCTGAGCTTTTTGTCAGTG
ACAAAGCAAATCTCCTTCATGGGATGTATGACACAGCTGTACTTCTTCCTCAGCCTGGG
CAACACTGAGTGCCTTCTCCTGGCTGTCATGGCATATGATCGCTATGTTGCCATTTGCAA
GCCTTTCCACTACTCTACTATCATGAGACACGCTGTCTGTGTCTACCTTACTGTGGGATC
TTGGTTGACTGGTTTCTTAATTTCTGGATGCAAAGTATTTTTTATCTCTCAGCTAACATAC
TGTGGACCCCATATAATCAACCATTTCTTCTGTGATGTTTCTCCTCTCTTGAACTTAGCC
TGCACAGATATGGAAAGAGCTGCTCTTATGGATTTTGTGGCTGCTTTATTTATTCTCCTG
ATGCCTCTCTCCGTAGTAATCCTATCCTATACCTATATTATCTTCACTGTCCTTCACATTTC
ATCTGTACAAGGCTGCCAAAAAGCCTTCTCCACCTGTGCCTCCCACCTTATAGTGGTCA
TTGTCTTCTATGCAACAAGCATTTTCATCTATGTGAGGCCCAAAACACTTCCAGTTCAT
GACACAAACAAAATCGTGTCTGCTGTCTACGCTGTTGTTGTTCCTCTCTTCAATCCCAT
CATTTACTGCCTGAGGAACCAGGAAATCAAGGATGCTCTCCAAAAGATCTTGTTCAGA
AAAAGAGTTTCCCTGCAGAAATATTAG
>Emperor_OR5AP2 (Emperor14)
ATGGCTGGGGGAAGCCACTCCAGCGTGACCGAGTTTGTTCTCTTGGGCTTCACTGACC
TGCAGGAGCTGCTCTTCGTGATTTTTTTACTCATCTACATCACCACGCTGGTGGGGAGC
CTGGGGGTGATCGTCCTCATCAGGACCAGCTCTCAGCTTCACGTGCCCATGTGCTTCTT
CCTCAGGCACCTCTCTTTCCTGCACATCTGCTATTCTTCATCCATCACGCTGAAGCTCCT
GTCGGGTCTCCTTGCAGAGAGAAATGTCATTTCTTTCAACGGCTGCATCACACAGTTGT
TCTTCTTTGCAGTATTCGGCACCACGGAAGCCATCCTTCTCACCCTCATGGTGTACGAT
CGCGACGTGGCCATCCGTGAGCCTCTGCGCTACTTGGCTGCCATGTCCCATGGGGTCTG
TGTGCAGCTGGTGGTGGGCTCCTATGCTGCCGGGAGCCTGAATGCCCTCGTGCGCACC
AGCGCTCTCCTCCGACTCTCTTTCTGTGGCCCAAACCTTGTCAATAATTTCTACTGTGA
AATCCCACCACTTCTGCTACTCTCGTGGTCCAACGCCCAGCTCAACGAGATGGTGATG
GCCGCGTGCGTTGGCTTCATCATAACAACCTCAGTCTTGGCCATCGTTGTCTCCTATGC
CTGCATCCTGCTCACCATCTGGAGCATCTGCTCTGTGGAGGGCAGGCACCAAGCCTTC
TCCACCTGCACCTCCCACCTCATGGCTGTTGCCCTCTTCTACGGCTCTGCGGCTTTCTT
GTATTTCCATCCCTTTTCCAGACGCGCCGAAGATCAAGGGAAAACAGCCTCCGTCTTCT
ACACCATGGTGACCCCCATGCTCAACCCTTTCATCTACAGCCTGAGGAACAAGGAGGT
GAGGAGCACCCTCAGAAGAGCTACGAACGAGCTCCTCTCCTGCAAGTGTTCCCACAG
GTTCCGCTCTAACCAAGCCGAAGCAGATATCACCTGCCACAGTGGAGTCAGCAGAAG
AAATCAGGCCGACACCTGA
>Emperor_OR52I1 (Emperor15)
ATGGCTTCTGATCCCTTCAGCCACCCCAACAGCAGATCCTCCTCTTTCATCCTTGTGGG
TGTCCCCAGCCTGGAAGCTTTCCCTACCTGCCTGGACATCCTTTTCTGCTCAGGCTATA
ACATCGTCTTGGTAGGAAATGGTGTAGTTTTGCTTGTCATTGGGCTGGACAACTCCCTG
CGTGAGCCCATACATTGCTTCCTGGGCAGGCTGGTGGTCATCAGTGTGGTGATGGTGA
CATCCATCGTCCCCAAGATGCTGAGCATGTTCTGGCTGAACTCCACAGAGATTGGTTAC
ATGGCCTGTTTTGTTCAGACGTTCCTTGTTCACTCCACAACATCAGAGGAGTCGGGAG
TGCTCCTGGCCATGGCTGTTGACCACTACGTTGCAATTTGTCACCCCCTCTGGTACCAA
GCTATCTTGAATCGCCAAACACTTGCCCAAATAGGCCTGGCCATTGTGGTGAGAGCTCT
CCTTTTCATGGTCCCCTTGACAGGGATGGTGACAAAACTCCCCTGTTGCCATTTCCCTG

TGGTTCCCCATTCGTACTGCGAGCACATGGCGGTGGTGAAGCTGGCGTGCGCAGACCC
CAGACCCAGCAGGCTTTACAGTGTGGCAGGGTCCTCTCTTATAGTAGGGAAGGACATG
GCTTTCATTGCTGTGTCCTATGGGATGATGCTTAAAACTGTCCTGGGGAAGAAATTGTG
CTGGAAGGCCTTCAGCACCTGCAGGTGTCATATCTGCGTGATGCTGCTGTATTACATCC
CTGGGATAGTCTCCATATATGCACAGCAGTTTGGCAGTGGCATATCCGTGCATGCACAG
GTTCTGCTGGCTGATCTCTACCTGGCCCTCCCCACCATGCTGAACCCCATCGTTTACAG
CATGAGGACCAAGCAGATCCATCAGGCAGTGGTCAAAATGCTATTTTCCAGGAAGGTT
CATGCCTGA
>Emperor_OR5V1 (Emperor16)
ATGGAAAACCAAACAAGTGCAAGTGAGTTTGTTCTCTTGGGACTTACAAGTGACCCAC
ACCTTCAAAGCCTCCTCTTCTTTGTTTTTCAGTTATTTATTTAATCACTCTGTTTGGAAA
TATGGTGATCATGATTGTGATAAGTACTGATCCGCACCTTCACTCCCCTATGTACTTCTTC
CTTTTTCACTTAGCCCTCACAGACATCTGTTATGCTACCACCATTATTCCTTATATGCTGG
TGAAGTTCCTACTGAAGCAGAGAACCATTGACTTCAGTGCCTGTATTATCCAGATGTCC
TTAATCCTTCTCTCAGCTGGTAGTGAAATTTTCATGCTCTCAGTAATGGCATATGACCGA
TACATTGCCATCTGTAAACCACTACAATACCAAGAGGTTATGAACAAATCTGTCTGTAG
CCAGCTGGTGGGGGGTGCATGGGCAATGGGGGTCTTACACTCCATTATAAACACACTG
CCGATGCTAAATGTGCAATTCTGCAAGCACACAGAAATTAAGCATTTCAGCTGTGAGTT
GCCCCCTCTCTTAACCGCAGCCTGCGGCAGGACCTTCCTCAATAAACTTGTTCTTCTGT
CTTCTGCTGTGATCTCTGGGTCAAGCTCCTTTCTGCTCACTCTCATCTCCTATATCTATAT
CATCTCTACTGTCCTGAAGATACAGTCTGCAACGGGGAGACAGAAAGCTTTCTCCACT
TGCAGCTCCCACCTCATCATAGTGGTTTTGTTATACACAACTGCTTTGTTCCAGTACACA
AAACCCAGTTCAGTCTCATCATTCATTCTAGATCAACTGTTTTCCATCCAATACAGCATT
TTAACCCCCATGCTAAATCCCATCATCTACAGCCTGAAAAATAATGATGTGAAAACAGC
TTTGGGCAGAATGTTAGGGAAAATTCAAGTTTCACAATCAGTGTAA
>Emperor_OR10C1 (Emperor17)
ATGAAATCCTCTGAAGAAGGGACCACTGGAAATAAAACTTCTGCAATCACATTTGTTAT
CCTGGGTTTTTCTCATCGCCCAGATATGAAAGTCATCTTCTTCATCCTATTTCTATGTATT
TACATCATCACGGTGCTGGGAAACCTAATTATTCTTCTTCTAATTAACATGGACCCTGTC
CTCCACACCCCCATGTACTTCTTCCTCAGAAACTTGTCATTTCTGGAGATCTGCTACAC
CTCTGTCACCCTGCTGAGAGTGCTGGTCAGTCTTCTCTCAAGTGATACATCCATCTCTT
TTGCAGGTTGTGCTGCGCAGATGTATTTCTTTCTGTTCTTTGGGGCGACTGAGTGCTGC
CTCTTAGCTGCTATGGCATATGACCGCTACCGAGCCATATGCAACCCTGTGGATTACATG
GTTATCATGAATAAGAAAGAATGCATGCAGCTGGCTGCTTCCTCATGGATATGTGGCAA
CCTTGTGGCCCTTGGGCACACTACATTTATTTTCTCTTTGCCCTTCTGTGGCTCCAACGT
GATCAACCATTTCTTCTGTGAGATCCAGCCAGTGCTGATGCTGGTGTGTGTGGACGCTT
ATTGGAGTGAACTACAGATCATTCTGGCTGCTGCCTTTGTCATCATGATGCCTTTTCTTC
TCACTTTGGTGTCCTACGGCCTCATCATTTCCTCCATCCTCAAAATCGGGTCTGCCAAA
GAAAGGTATAAAGCATTCTCTACTTGCTTCTCACATCTCACTGTAGTGACATTATTCTAT
GGGACAGTTGTGTTCATCTATATACGTCCCAAATCCAGCTATTCCCTGGATGCGGACAA
GGTTCTCTCTCTGTTCTGTTCTGGGGTGACCCCTATATTGAACCCTTTTATCTACAGCCT
TAGGAATAGGGAGGTGAAAGGAGCTCTCTTTAAAATGAGAATGAAGCTATTTTACCAC
AACTTCTAG
>Emperor_OR5R1 (Emperor18)

ATGGGAGGAAATCACGTGCAGACTAAATTCATCCTCTTGGGAATTACAGACCGTCCATG
TGTGCAGGCCCCTCTTTTTGGGTTGTTTCTATTGATTTACATTGTCACTGTGGTGGGGAA
CGTTGGGATTATGGTCTTGGTTTGGGTGGTTCCCAGCCTCCACACCCCCATGTACTTCT
TCCTCACCCATTTTTCATTCGCTGACGTCTGCTATTCCACAGTCATTTCCCCCAAAATGC
TAGCAGACCTGTTATCAGAGAATAAAACCATTTCTTTCGCTGGCTGCGTGACGCAGTTC
CATGGCTTTGCTTTCTTTGCGACTGCCGAGTGTCACCTCCTGGCTGTGATGGCCTATGA
CCGGCATGTTGCTATCTGCAACCCCCTGCTTTACGTGACGGTCATCTCCAGCCGCATCT
GCCGGCAGCTGGTAGCATCGTCCTACCTCATCGCCTTTCTCAGTGCCATCATCTACACA
GGCTGCACGTTTGGGGGTTCCTTCTGTGGACCCAACCAGATCGACCACTTCTTCTGCG
ACGTCAGCCCCGTGCTGAAGCTTGCCTGCTCCGACACCCGCAGCAGCGAGATGGTCAT
CTTTGCCTTTGTCGCCATAAACGCGGTGGGCACGAGCGTGATCATTTCGCTTTCCTACA
TCTGTATCCTCCGCACAGTCCTGAGGATGCGCTCGGCACGGAGCAGGTCCAGAGCCTT
CAACACCTGCGCCTCCCACTTGACGGCTGTCTCCTTATTCTACGGGACAATATTCTTCA
CGTACCTACAACCTGCGTCTAGCCACAGCAGCCTGGATAAGGTGGCCTCCATCTTCTAC
ACCGTGGTCACCCCCATGCTCAACCCATTCATCTACAGCCTGAGGAACAAGGAGGTGA
AGGGCGCTCTGGTGAAGTGCAGGAGAAGGGTGTTAAACCGCTGTCAACATAGAAGAG
CTGTATCAGCTAGGCAGTGA
>Emperor_OR6B1 (Emperor19)
ATGAAAACACGTGAAGAGGACAATCAGACATCACCCATGGAGTTCCTGCTGCTGGGAA
TGGGGAATACGCCCACACTGCAGATTCCTCTCTTCCTCCTATCACTCATCATTTATTCAG
TGATTGTGATTGGGAATATCCTCATAGTTGTGCTGGTGGTGTCAGAGCAGCATCTGCAC
ACCCCCATGTACACTTTCCTGGGCACTCTGTCCTTTTTGGAGACCTGGTACAGCTCCAC
CATCCTGCCCCAGCTGCTTGCTAGCTTCCTAACTGGGGACAGGACTATCTCTGCTCACG
GCTGTATGGCTCAGTTCTACTTCTTCAGCTCTTTTGTAGCTACTGAGTGTTACCTGCTGG
AGGCCATGTCTTATGACCGGTACTTGGCTATATGCCAGCCCCTGCTCTATGCAAGCCTCA
TGACCTGGAAAATCTGTCTACAGCTGGTGGCTGTATCTTGGCTAGTGGGTATGCTGGTG
TCTGCAACAGTCACAGCCTTTTTATCCCAGATTAATTTCTGTGGCCGTAAGACAATCGA
CCACTTCTTCTGTGACTTTACCCCTCTGCTGGAGCTTTCCTGCAGTGACACCAGTGTGG
TCACACTTGTATCTTTCATCATGTCTTTCCTGAGCATTCTCTTCCCCTTCTTGTTCACGCT
GGTCTCCTATGTGTGCATCACAGCTGCCATCCTGAGGATCTCATCCAACATAGGCAGGC
AGAAGACCTTCTCCACCTGCTCCTCTCACCTCATTGTTGTCACTATTTTCTACGTCACCC
TTCTCATTGCCTACCTGCTGCCCAGAACAGCTCCGCTGAGGCAGCTCAACAAAGTGTT
CTCCTTTTTCTACACCATTCTGATGCCCCTGGTCAATCCGTTCATCTATATCCTGAGAAA
CAAGGAGGTCAGCGAGGCCCTGAGGAAAGCACTCAGAAAAGCCATGGCCAGCACCTA
G
>Emperor_OR5R1 (Emperor20)
ATGGTTGAAGATAATGATACATTTCCATCTGAGTTTATTCTCCTGGGCTTCACAAACCGA
GAAGACCTGCAGGTGACATGCTTTGTCTTATTCCTTGCCATCTATGTGGTCACTCTAATC
GGAAATCTGGGAGTAATTATATTAATCAGAATCGATTCGTGCCTACACACCCCCATGTAC
TTCTTCCTAAGCCACTTGTCTCTCCTGGACGTCTGCTACTCCTCCACCATCATCCCTCAA
ACCTTGCTGAATTTTTTAGTGGAGAAGAAGGTTATTTCCTTCGTTAGGTGTGCCACTCA
GCTCTTCTCCTTTGCGACTTGTGCCACCACCGAGTGCTACATGCTGGCTGCCATGGCTT
ATGATCGCTACGTGGCCATTTGTAACCCCCTGCTCTACTCCGTGGTCATGTCCCAGAGG
TTTTGCGTTGGGATGTTGGCTGGTGCCTACTTAGCTGGTGTGATCAGCTCCATCATACA

CACAGTCTCCATATTTCATTTCCCGTTCTGCCGGTCCAAGAGGATCAATCATTTCTTCTG
TGATGGACTACCGCTGCTAGCCCTCTCCTGCTCTGACACACATGTCAACGAGGCGATC
GTTTCTGCCGTGGTGGGGTTCAACATGCTAAGCACCATGGTCTTCATTCTAGTCTCCTA
CTCGTCGGTCCTCTCCACTGTCTTGCGGATGCGCTCCGCGGCCGGTTGTCACAAAGCC
TTCTCCACTTGCGCCCCTCACTTGGTCTCCATCGCTTTGTACTACGGCAGCTCCCTCTTC
ATGTACCTGCGCCCCGGCTCCAGACACTCCTTGGAGCGTGACAAGGTGGTCTCCATGC
TGTACTCCATTGCGCTCCCCATGCTGAACCCGCTCATCTACAGCCTAAGACACACGGAC
ATGAAGAACGCCATGAGAAAAGCAAAAGGTAGAGTCCTCTCCTCCTTGTCCATCCACG
GTTCCGGGCCAGGTGAAAGGAGAGGGCTACCCTTGCATGGTAAGAAGGGCTAG
>Emperor_OR4S2 (Emperor21)
ATGGAGAATGCAAGCAGTGTGAAGGAATTCATTCTTCTGGGCGTCTCAGAGAATCAAG
CGGTGCAGAAAATATGTTTTGTGATGTTTCTGTTCTTCTATATGGTTATTGTGGCAGGAA
ATCTGCTCATTGTTATCACTGTAATTAGCAGTCAATGTCTGAACTCTCCCATGTATTTCTT
CCTCTGCTACCTGTCCTTTGTAGATATCTGTTACTCTTCCGTCACAGCTCCCAAAATGAT
TGCCAACTTCCTTGTTGAAAATAAAACCATCTCCTTTGTGGGTTGCATAGCACAGCTGT
TTGGGGTACATTTCTTTGGCTGCACAGAGATCTTCATCCTCACAGTGATGGCCTACGAT
CGCTATGTTGCCATCTGCAGACCTCTCCACTACACCACCCTCATGACCAGGCGTGTGTG
TGGCTGGATGGTGATCGGCTCATGGGTAGGAGGTTTCGTGCACTCCACAGTGCAGACT
CTTCTAACCACTCAGCTCCCCTTCTGTGGCCCTAACAAAATTGACCACTACTTCTGTGA
TGTCCACCCCCTACTACAACTGGCCTGTACCAACACCTACGCTGTGGGCATCATTGTCG
TTGCCAACAGCGGAATGATAACTCTGAGCTGTTTCTTCATCCTGGTCATGTCCTACGTT
GTCATCCTGGTTTCCTTGAAAAGTCAAACATCCAAAGGGTGGCACAAGGCCCTCTCCA
CCTGTGGGTCCCACATCACTGTGGTGATTCTGTTCTTCGGGCCATGCATGTTCATCTACA
TACGTCCGTCCAGCAATCTGTCAGAGGACAAGAGCGTGGCAGTGTTTTACACTGTCAT
CACACCCATGCTGAACCCACTCATCTACGCGCTAAGAAATAAGGAGGTGAAGAGTGCC
ATGAGAAAACTATGGAGTAGAAAAGTGGGAAGTGAAGATGGAAAGGTGTAG
>Emperor_OR6B1 (Emperor22)
ATGAAGAACCAAACTGTTATCAATGAGTTCATTCTTCTGGGATTTTCCTATGGGCTGCA
GGTCCAGACCTTACTTTACCTGGTCTTTCTGGTCACCTACATGGTAACAATCACTGAGA
ATGCAATCATCATCTTTGTGGTGAAAAGGAACCGTCACCTCCAAAAGCCCATGTACTAT
TTCCTGGGGAACTTGTCCTTCCTGGAGATTTGGTACGTCTCAGTAACGTTGCCTAGGCT
TTTGTTTGGGTTCTGGTCACAGACCATGACCATCTCATTCTCCAGCTGCATGACCCAGT
TATACTTCTTTATCTCCCTTATGTGCACTGAATGTGTCCTCTTGGCTGTAATGGCCTATGA
CCGCTACTTGGCTGTCTGCCATCCCCTGCGCTACCCAGCCATCATGACCCACAAGTTGT
GTTTTCAGCTGTCGATTCTCTCATGGGCAGGAGGCTTTTCCATTTCCTTGGTCAAGGTG
TCTTTTATTTCACGCCTCACATTTTGTGGTCCACAAGTAATAAACCACTTCTTTTGTGAC
ATCTCTCCAGTACTGAACCTTTCCTGCACTGACATGTCCCTTGCAGAGACAGTGGACTT
TGCATTAGCCTTGGTGATTCTGCTGGTACCTCTCTTGATCATTGTTTTCTCCTACTGTGGT
ATCTTGTCAACTATTTTGTGTACGCCTTCAGCCCAGGGAAGGAGAAAAGCCTTTTCCAC
TTGTACCTCCCATTTCACTGTAGTCATTATCTTTTTCTCAGCCACTCTCTTCATGTATGCC
AGGCCCAGGAGGATCCATCCATTCAACCTCAACAAAATAGTGTCTGTCTTTTATGCTATA
TTCACTCCAGCACTGAACCCTCTAATCTATTGTTTGAGGAACAAGGAGGTGAAAGAGA
TTCTGAGAAAGATCATAGGCAGAAGCTGCTGTGCACACCGATAA
>Emperor_OR5AP2 (Emperor23)

ATGATTGTCTCCGAGCAGGTCCCACTGGAGAGAGGGTTTTCCACCTGGCAGATACCTG
CCTGTTGTAGAAGAAACCTCCAGCAGGGAGTTCACACAGAAAGCACGCATCCAACTA
ATGGCAGAAAGCAATTGCACCCAAGCGATTTGAATGAATTCAGCTTAACGGGGTTCAC
AGAGGGCCCAGTGACTCAGGTCACCCTCTTTCTGGTGTTTTTGCTCACCTATCTTGTCA
CCATCCTGGGGAACCTTGGGACGATTGCGTTAATCAGGGCCAGCCCTCAGCTTCACTC
CCCCATGTATTATTTCTTGGGTAACCTGGCTTTTGTAGACCTCTGTTCTTCCACCGTCAT
CACCCCTGAGATGCCGGTTGACTTTACGTCAGAGAAGAAGGGCACTGCTTACGCTGGG
TGCGTGGCTCAGGTGGTCATTTCTGATCTTTTTGGGATGACCAAATGCTTCCTGCTGGC
TATGATAGCGTATGACCATTACATGGCCATTTGCCATCCCCTGGTGTATCCGCTTGTCCT
GTCCCCAAAATGCTGTTTCCAGCTGGTGACTGGGTCATATCTCGTGGGGTTGACAAATG
GCATGGGACAGACTATTGGCACGACCAATTTATCCTTCTGCAGCTCCAGCGTCATCGAC
CTGCTCTTCTGTGACTTTTCCCCTCTGATAACACTCTCAAGCTCCGACACCACCCTCAG
ACACATATTAAGAACTTCAGCATCTTGGGTCGGTGTGGCCAGCAGCCTGGTTGTCCTG
GTCTCCCATGTGGCCATCATCTCTGCCACCCTGAGCAACAGTTCAGCGGAGGGCAAGC
ACAAAGTCCTCTCCACCTGCGCCTCCCACCTCACCACTGTGAGGATCTTCTGCGGGAC
ATCGCTTTTTATGTGCTTAAAGCCCAGCTCAGACAGCTCGAGAGAAGAAGATAAACGG
GCTGCGGTGCTCTACACTGTGGTGACTCCCATGCTGAACCCCTTGATCTACAGCCTGAG
GAATACGGAGGTGAAGGAGGCTTTGAGGAGACTCACAAATATCAAATGA
>Emperor_OR2AT4 (Emperor24)
ATGGAAAGCTGCAGCAGCAACGCTTCCACTAAAGTCTTTTTCTTGGTGGGGTTTCCAG
CTTTCCAGGATTTCCAGACCCCCCTCTTCGTTGTGTTTTTGCTATTCTACCTGCTGATCC
TGGTTGGTAAGGCCGTCATCATCACCGTGGTTGTGGTCGACCACACGCTCCACAAACC
CATGTACTTTTTCCTGATTAACCTCCCTGTGTTAGACGTGCTTTTCACAGCCACCACCAT
CCCCAAAACACTGGCAATGTTCCTGGGCAACGCTGAAACCATCTCGTTTTGGGGCTGT
TTTCTGCAGATGTACAGTTTTCATGGGTTGACAGTAACCGAGGCGCTTCTCCTAGCAGT
CGTGGCTTACGACCGCTACGAAGCTATCTGCAACCCCCTCTGTTGCCCGGCCAAGATG
ACAAGAAGAGTGAACATCCATCTGGCAGCGAGCGCCTGGATCACTGCGCTGCTAATAC
CCGTTCCCATCATCACGCAGACCTCTCAGCTGGCTTACAGGGACACAACCAGGGTTCA
CCACTGCTTTTGTGACCACCTGGCAGTGGTAGAAGCCACGTGCTTGGACTTCAGTGCC
AATTTCCAGACTTTCTTGGGGTTCTCCATCGCTATGACAGTGTTGGTCATCCCTCTGTTG
CTTGTCACCCTCTCGTACGTCCACATCATCCTCTCCATACTAAAGATCAACTCCAAAGA
AGGACGCATGAAAGCTTTTTCAACGTGTACTTCCCATCTGCTTGTGGTGGGCACTTACT
ACTCCCCCATCGCTGTGGCGTACATGTCCTACAGAGCGGACATCCCCGTTGATGTCCAC
GTCATGAGCAACGTTGTCTTCTCTATTCTAACTCCCTTGTTAAACACCATCATTTACACT
TTACAGAACAAGGAAGTAAAACCTGCAGTTAAAAAGTCTATTTTTCTGAAAATCTTTCC
CCTTTCTAAAAAATTTAATTTATTTGGGTAA
>Emperor_OR5J2 (Emperor25)
ATGCAGGACGGTAACACGGCTAAAGGCAATCGCACCGTGGTGACCCAGTTCATCCTCC
TAGGACTGACAAGCGAGCCTAAGCTGCAGAGGCCTCTCTTCATAATCTTCTTAATGATT
TATCTCATCACCCTGATGGGCAATCTTGGGCTGATCACATTGATGAAGACAAACCGCCG
GCTGCACACTCCCATGTACTTCTTCCTCTGCAATCTGTCTGTTGTTGATCTTTGCTACTC
CTCCGTCTTTTCTCCAGAGCTGCTTGTTGGCTTCTTGGTGGAAAAGAAAACCGTTTCTT
ACTCCGCCTGCTTTGCCCAGCACTTCTTTTTCCTTGTGTTTGTGACCACGGAGGTGCTC
TTGCTGGCTGTGATGGCGTACGACCGCTACGTAGCCATTTGCAACCCGCTGCTCTACAC

TATTTCTATGCCCAAGAGGGTCTGCGTTCAGCTGGTGGCCGGGTCGTACGCAGGGGGG
ATTTTGAACTCGCTAATCCAAACCTGTTGCTTGCTGCCGTTGCCTTTTTGTGGACCCAA
TGTCATCAACCATTACTTCTGTGACACTAACCCTCTGCTGAAACTCACCTGCTCCGATG
ACCGCCTCAATGAGCTCTTGCTTGTAACCTTCAATGGGACCATTTCCATGTCCGTGCTC
CTCATCATCATCATCTCGTACGTATACATCCTCTTCTCCATCCTGAGGATTAGGTCCGCC
GAAGGCAGGCACAAAGCCTTCTCCACCTGTGCCTCCCACCTCCTGACTGTTACCTTGT
TCTACGTGCCCGCGGGGCTGAGCCACATGCAACCGGGCTCCAAGTACTCCCTGGAGAT
GGAGAAAGTCACCGCCGTGTTTTATACCCTGATCATCCCTATGCTCAACCCTCTGATCTA
CAGCTTGAGGAACAAGGAGGTCAAGGATGCACTTCGAAAAGCAACAGCAAATAACAT
TTTTGCGAGTCGCCTGCTGACCAAACGGACCCTGATCAGTTGA
>Emperor_OR11L1 (Emperor26)
ATGACAAATGTTACAGCAATACTGGAATTCAGGCTATTGGGCTTCAGTAGCAACCCACA
CTGCCAGATCCTGCTATCCACAGTGTTTTTAGTTATTTATATTCTCACCATCCTAGGAAA
CATCATTATTATTTCAGTGGTGACACTGGAGCCACAACTTCATTCACCCATGTACAAATT
TCTCAAGAACCTCTCTTTCCTAGAGATCTGTTACACCACCACAATTGTACCCAAGATGC
TGGCCAATCTACTGGCAAAGAGGAAGAGCATCTCCTTCTCAGGATGCATGGCACAGCT
TTATTACTTCATTTCCCTGGGAGCCACTGAGTGCTACCTCTTGGCAGTGATGGCATATGA
CCGATACCTTGCAGTCTGTGAACCCCTGCACTATGGTATGGCCATGACTGCTGAGTCTT
ATACCTGTCTGGCTGTGGGCTCCTGGGTCACTGGTGTTTTCACTGGTTTTCTGCCCTGT
CTGATGGTCTCCAGATTGCATTTCTGCAGTTACAACCTCATTGATCACTTCTTCTGCGAT
ATCTCTCCACTGCTGAAGCTCTCATGCTCAGACACCACTGCCGCAGAAACTGTCATCTT
CATCCTCTCTCTCCTGGTCCTTTCCAGCTGCTTTCTGTTGACTCTTGTCTCATACCTACTT
ATAATTCTCAGTATACTGAAGATACCCTCTGCTTCTGGAAAAAGAATTACCTTTTCCACC
TGCAGCTCACATCTCATGGTAGTGACTATATACTATGGTACAATGATTTCCATGTATGTCC
GTCCCACCTACAACCTCTCCTCAGAGCTCAATAAGGCTGTATCTGTGCTCTACACAGTG
GTCACACCCCTTCTGAACCCAGTAATCTACAGCTTGAGAAACAAGGCATTCAAGAAGG
CCTTGGAAAAAATAGTCATCAGACACCATTGTCTTCATTCTTTCTAA
>Emperor_OR52K1 (Emperor27)
ATGTTTTTCTTGACTGGCATCCCTGGGCTGGAAGATATGCACCCCTGGACTTCCATCCC
ATTCAGCAGAGTGTTCAGCGTAGCCCTCCTTGGGAACTGCACCCTCTTGTACGTGATG
AAGACAGAGTCATCTCTCCGCAAGCCCATGTTTATTTCCTCTCCATGCTGGCTGTCATT
GACTTGGTGCTGTCCCTGACCACCATGCTCAAAATGCTGATCATCTTCTGGTTCAATGC
CCAGGAGATCACCTTCAGTTCCTGCCTTGTGCAGATGTTTTTCTTCACACTTTCTCAG
TCATGGAGTTGGTGGAGCTGCTGGCCATGGCTTTCCACATGTATGCTGCCATCTGCAAT
CCCCTGAGCTACAAGCTCCATCCTGACCATCTCCCTGGTAGTCAGGAACGGGCTGCTG
GCTCTAGCCAGGGCAGCCGGGCTCATGTTGCCTCTGCCTTTCTCCTCCGTCGCCTGCCG
TACTGCTGCTCCCACATCACCTCCCACTGCTACTGCAAACACATGGCAGTGGTGGAGC
TGTCCCGTGCCAACACCAGGTTCAGTAACATCATCGTGGTTCTCTTCATCGTGGGGTTG
GATCTGTTGTTCATTGGGCATTCCTACCTCAGGATCTTGAGGACTGTGTTGAGCCTGGC
ATTGAAGGAGGACAGACTGAAGGTACTTGGGACCTGCTTCTCCCACATCTGCGTCATC
CTGGTCTTCTATACCCCTGTGGTTCTCTCATTAGTAATCCACAGGTTTGGTTGCCATGTT
GCCTCTCACATGCACATCTTGATGGCTAATTTCTACCTCCCATTCCCCCCCATGGTGTAT
GGTGTCAAAACCAAGCAGATCCATGATGGGCCACAGAAATTAACCCTGCATACATAA
>Emperor_OR4D5 (Emperor28)

ATGGCACTGGGAAACTTCTCCCGGGTGACTGAATTCATCCTCCTGGGGCTTTCCGATAC
AAGGGAGCTGCAAGTCCTCTTCTTCACTTTCTTCTTCCTGGCCTATGCCATGGTTCTGC
TGGGGAACCTTCTCATCATTGTGACAGTCAGGACTGACCCCAAGCTGTCCTCGCCCAT
GTACTTTCTCCTCTGCAATTTGTCCTTCACAGATATCTGCTGCACTTCTGTCACCTCTCC
CAGGATGCTGGTGGACCTGCTCTCCCAGAGGAAGGCCATTGCTTTCGAAGACTGTATA
GCCCAGCTGTTTTTTCTGCACTTTGTCGGGGCATCAGAGATGTTCCTCCTGACTGTGAT
GGCGTATGACCGCTACACTGCCATCTGCAAGCCCCTGCACTACACAGCCATCATGAGCC
GGCAGGTATGCTGGGTCCTGGTGTCTGCCTGCTGGGTGGGGGGCTTCCTCCACTCCATT
GTCCAGACGCTGCTCACAATCCAGCTCCCCTTCTGCGGCCCTAACACAATCGACAACT
ACTTCTGTGACGTGCCTCCTGTCATTCGGCTTGCCTGCACAGACGTCTACATCACTGAG
TGGCTCATAGTATCCAACAGCGGCTTGATATCCCTGGTGTGCTTCCTGGTGCTGGTCAC
ATCTTACACGTTCATCCTGGTCACAATCAGGGTCCGCTTCACTGAGGGGCACTGGAAG
GCGCTCTCCACCTGTGCCTCACATGTGATGGTCGTCACCCTCTTCTTTGTACCCTGCATC
TTCATCTACCTCCATCCCTTTTCTACCTTCCCCTCCTACAAGCACATCTGTGTGATCTGC
ACTGTCTTCTCCCCGGTGATGAACCCCCTCATCTATACCCTGAGGAATAACGAGGTAAA
GGCGTCCATGTGGAAGTTGTGGAAGCGCTGCAGAGTCTTCTGA
>Emperor_OR6B1 (Emperor29)
ATGGAGAAAGGAGGACAGGATAATGGGACGTCGCCATTGGGGTTCCTCCTGGTGGGAA
TGGGGAATGTTCCCACGGCCCAGGCGGCGCTCTTCCTCCTCTCGCTCATCATCTACTTG
GTGACCGTGGTTGGGAACATCCTCATCATTGTGCTGGTGGTGGCAGAGCAGCATCTGC
ACACCCCCATGTACTTCTTCCTGGGCAATCTCTCCTCCTTGGAGACCTTCTACAGCTCC
GCTATCCTGCCCCGGCTGCTGGCCAGCTTCCTGACTGGGGAGAGGACCATCTCTGCTC
ATGGCTGTGTGGCTCAGTTCTATTTCTTTGCTGCTCTTGCAACTACCGAGTGTTACCTGC
TGGCAGCCATGTCCTACGATTGGTACTTGGCCATATGCCAGCCCCTGCTCTACGCAAGC
CTCATGACCTGGAAGGTCTGTCTACAGCTGGTGGCTGGGTCTTGGCTACTGGGATTGCT
GGTCTCCACGGTACTTACGTCTCTCTTGCCCCAGTTACAGTTCTGCGGCCCCAGGACGA
TCAACTGCTTCTTCTGTGATTTTACCCCATTGCTGGAGCTTGCCTGCAGTGACACCAGC
ATGGCCACACTTGTTGCTTTCATATTTGGCTTCTTGGATGTGGTCTTCCCCTTCCTCTTC
ACCCTGGCCTCCTACGTGTGCATCATAGCTGCCATCCTGAGGATCCCATCCAACGTGGG
CAGGCAGAAGGCCTTCTCCACCTGCTCCTCTCACCTCACCATCGTCGCCGTTTTCTATG
GCTCCCTCTTTGTTGTCTATGTCCTGCCCAGAACAGCCCCGCTGTGGCAGCTCAGCAA
AGTGTTCTCCTTCTTCTACACCGTCCTCACGCCCCTGGTCAACCCCCTCACCTACAGCC
TGAGGAACAGGGAGGTCAGGGAGGCCCTGAGGAAAGGACTCCGAAAAGCCCTGGCC
TCCACCCAGACTTTGTAG
>Emperor_OR8U9 (Emperor30)
ATGACACCTGTCAATCACACTGGTTTGCATGAGTTCGTTCTCTTGGGGCTGACCACCCG
CCCAGATCTCCTGGTCCCCCTTTTCTTGGCTTTCCTGGCCATGTACCTGGTGACTCTCTT
GGGGAACTTTGGGATAATTATATTAATCAGGACCGATCTTCACCTTCACACCCCCATGTA
CTACTTCCTCGGCCACTTGGCTTTTGTTGATATCTGCTATTCCTCCATCATCCTCCCCAA
AATGCTGGTGCAGGTCTTGACAGAGGAGAAAACCATTGGCTTCTCGGGGTGCGCAGC
CCAGCTCTGCTGCTTCGTTGTTTTTGGGGTCACTGAGTGCCTCTTGCTGGCCGTGATGG
CCTACGACAGGTATGTGGCCATCTGCAAACCCCTCCTGTACCCTGCCATCATGAGCGGA
TGGACGTGTCGGTGGCTCGTCGCTGGTTCCTACGCCATCGGCATCTTGCACGCGGTGA
CACACACCGCTTTCATCTTTACTTTCTCCTTCTGCCGCTCCAATGTTATCAACCACTACT

TCTGCGACATTGCCCCGCTCTTAGCTCTCTCCTGCTCCGACACCCACACCTACGAGGTG
GTTGTCCTTGCTCTCGTCAGCATAAACTGTCTCAGCACCATGACCATCATCTTTGTCTCC
TATACTTATATCCTCCCCACTGTCCTGAGGATCCGCTCTCCGGAGGGCAGGAGAAAAGC
CTTCTCCACCTGCGCCTCCCACCTGACGGTCGTCACCATGTTTTACGGGGCAATCTTGT
TCATGTACCTGCGCCCCAGCTCCACCTATGCGTTGGATGAGAACAAGACGGCCACGCT
GTTTTACACCATCATGACCCCCACGCTGAACCCCTTGGTCTACAGCCTGAGGAACAGT
GAGGTGAAGGCTGCCCTGAGAAGAGCGGTTGGGAGAAGGCAGTAA
>Emperor_OR5B21 (Emperor31)
ATGACAAAGCAAGAGTGGAAAAACAAAACGGTTGTTAAAGAGTTCATCCTCCTGGGA
TTTGGGAATGGCCCTGAACTGGATTCTCTTCTCTTCCTGATGTTTCTGTCAATCTACATT
GTGACCATAACTGGAAACTTCTTCATCATTGTGCTGGTGGTGGCTAATCGGCACCTTCA
CACCCCAATGTACTTCTTCCTGGGCAATTTGGCCTGCTTGGAAATCTGCTACAGCTCAA
ATATCTTGCCGAGGATGTTGCTTAGCTACCTGGGTGGAGACAGAAGTATTTCAGTCAAT
GGATGTTTTACACAATACTACTTCTTTGGTTGCTTGGCAGCTGCAGAATGCTATCTCCTC
GCAGTGATGTCCTATGATAGGTACCTAGCGGTGTGCAAACCCCTGCACTATCCATCCCG
TATGAACGGCAAGCTCTGTCTCCAGCTGGGTGCTGCATCTTGGATAAGTGGCTTTCTAG
CTAATTCTATACTAACATTCCTGATCTCAAATTTAGATTTCTGTGGGCCTAATGAAATCG
ACCATTTTTTCTGTGACTCATTCCCAATGATAAAACTCTCAGGTAGTGACAGTCATGTG
GCAGGACTTGTCACTTCTGTTGTGGCGGGTGTGTGCTCACTGCCTCTGTTTCTGCTGAC
CTTCTCATCCTATCTCTACATCATTATCACGGTCATGAGAATTCCTTCTGCCACTGGAAG
GAAAAAGGCCTTTTCCACTTGCTCCTCACATCTTATTGTGGTGATTCTTTTTTACTGGTC
AATATTAACTGTCTATGTACTTCCTCATCACGATACCCAAATATCTCCCAACAAAGTCTT
CTCTGTTTTTTATACCATTCTCACTCCCTTGGTCAATCCTCTCATCTACAGTCTGAGAAA
CAAAGAGGTAAAGGAAGCTCTGCAAAAACAGGCAAGTAAATTGCTGGCTTTCAGAGG
GCTGCTTTCTGTTAAAAAAAGGGGGTAA
>Emperor_OR2D2 (Emperor32)
ATGGCTAGGGAAAACCAAAGTGTAGTGACAGAATTCATCTTTCAAGGCCTTTCCTCCC
AGCCAAGGACACAGACTGTTCTTTTCACAGTGTTCCTGGTTTTTATCTGTTCACAATT
TTTGGGAACATCATGATCATTACAGTGATCAGAGCTGATTGCCAGTTGCAGGCACCCAT
GTACTTTTTCCTTGCCAACCTGTCCTTCTTAGATATCTGCTATGTCTCCAGCAACATCCC
CCAGATGCTGGTGAACCTCTTCACCAAGAAAAGGACCATCTCCTTCTCTGGATGTGCT
GCTCAGATGTATTTCTCTCTGGCTTTTGGCATGACAGAGTGTGTTCTGCTTGGGGTCAT
GGCCTATGATCGATACATGGCAATATGTCACCCTTTGCTGTACACCACTGTCATGAACA
GGAAGGTTTGCATTCACATGGTCATGGCTTCCTGGACCAGCAGCCTGCTGAGCTCCAT
GGTCATCAACAGCCTCACCTTGCGGCTGCCCTTCTGCGGGCCTGACATCTTGAACCATT
ACTTCTGCGAAGTGCCAGCAGTGCTGGCCTTGGCCTGTGCCGACACTGCCCTCATGGA
GTTGGTCATCTTCATCTTCAGCATCCTCATAGTCTTCATCCCCTTTCTTCTGATCATCACC
TCCTACGCCCATATCCTTTTCGCCATCTTGAAGATTCAGTCTGCACATGTGCAATCCAAG
GCCTTCTCCACCTGTGGATCCCACCTGACGGTGGTAACCATATTCTATGGGACAGCCAT
CTGCATGTACATGAATCCTAAGTCAAGGCCTCCACAGGACAGGGACAAAGTGGTTGCA
GTGTTTTACACCATTGTAGCCCCAATGCTGAACCCCCTCATCTACAGCCTCAGGAACAA
GGACATGAAGCGTGCCCTGAGAAGGGCAATGAATAGACCCAAATCCCTGTTTATTTAA
>Egret_OR14A16 (Egret1)
ATGTCCAACAGCAGCTCCATCACCCAGTTTCTCCTCCTGGCATTTACAGACACGCGGG

AGCTGCAGCTCTTGCACTTCTGGCTCTTCCTGGGCATCTACCTGGCTGCCCTCCTGGGC
AACGGCCTCATCATCACCGCCATAGCCTGTGACCACCACCTCCCCACCCCCATGTACTT
CTTCCTCTTCAACCTCTCCCTCCTCGACCTGGGCTCCATCTCCACCACTCTCCCCAAAG
CCATGGCCAATTCCCTCTGGGACACCAGGGCCATCCTTTACGCGGGATGTGTTGCCCAG
GTCTTCTTTATATTCTTCTTGTTTGGTGCAGAATATTCCTTTCTCACCATCATGGCCTATG
ACCGCTACGTTGCCATCTGCAAGCCCCTGCACTATGGGACCCTCCTGGGCAGCAGAGC
TTGTGCCCACATGGCAGCAGCTGCCTGGGGCAGTGCTTTCCTCTATGCTCTCCTGCACA
CGGCCAATACATTTTCACTACCCCTCTGCCAAGGCAATGCTGTGGACCAGTTCTTCTGT
GAAATCCCCCAGATCCTCAAGCTCTCCTGCTCAGACTCCTACCTCAGGGAAGTTGAGC
TTCTTGTGGTCAGTGGTTTTGGTTTTTTGGGATGTTTTTTTTCATCATGGTGTCCTATGT
GCAGATCTTCAGGGCCGTGTTGAGCATCCCCCTTGAGCAGGGACGGCACAGAGCCTTT
TCCATGTGCCTCCCTCACCTGGTTGTGGTCTCCCTGTTTGTCAGCACTGGCATGTTTGC
CTATCTGAAGCCCCCCTACATCTCCTACCCGTTGCTGGATCTGGTGGTGGCTGTTTTGTA
TTCAGTGCTGCCTCCAGCAGTGAACCCCCTCATCTACAGCATGAGGAACCAGGAGCTC
AAGGATGCACTGAAGAAGTTGATGCAATCAGCTGTCTCTTGGCAGCCTTAA
>Egret_OR14J1 (Egret2)
ATGTCCAACGACAGCTCCATCACCCAGTTCCTCCTCCTGGCATTCGCAGACACACGGG
AGCTGCAGCTCTTGCACTTCTGGCTCTTCCTGGGCATCTACCTGGCTGCCCTCCTGGCT
AATGCCCCCATCATCACAACTGTAGCCTGCTATCACCACCTCCACACCCCCATGTACTTT
TTCCTCCTCAACCTCTCCCTCATTGACCTGGGATACATCTCCACCACTCTCCCCAAAAC
CATGGCCAACTCCCTGTTGGATACCAGAGCAATTTCCTATGCAGGATGTGTTGCACAGG
TCTTTCTCGTTTCCTTCTTGCTCGGAGCAGAGTATTATCTTCTCACTGTCATGGCCTATG
ACCGCTACATTGCCATCTGCAAACCCCTGCACTACGGGACCCTCCTGGGGACCAGAGC
TTGTGTCCACATGGCAGCAGCTGCCTGGGGCACTGGCTTTCTCAATGCTGTGCTGCAC
ACGGTCAATACATTTTCAGTGCCACTCTGCAAGGGCAATGCTGTAGAGCAGTTCTTCTG
TGAAATCCCCCAGATCCTCAAGCTTTCCTGCTCAGATGCCTACCTCAGGGAGGTTGGG
CTTCTTGTGGTCAGTGGCTGTTTTGTCTTTGTGTGCTTTGTTTTCATTGTGCTGTCTTAT
GTGCAGATCTTCAGGGCCGTGCTGAGGATCCCTTCTGAGCAGGGACGGCACAAAGCC
TTTTCCACGTGCCTCCCTCACCTGGCTGTGTTTTCCCTGTTTCTCAGCACTTCATTTTTT
GCTTACCTGAAGCCCCCCTCCATCTCTTCTTCATCCCTTGATCTCGTTCTGGCAGTGCTG
TACTCGGTGGTGCCTCCAGCAGTGAACCCCCTCATCTACAGCATGAGGAACAAGGAGC
TCAAGGATGCACTGAAGAAGCTGATGTAA
>Egret_OR14J1 (Egret3)
ATGTACAATGGCAGCTCCATCACCCAGTTCTTCCTTATGGCATTCGCAGACACGTGGGA
GCTCTTGCACTTCTGGCTCTTCCTGGGCATCTACCTGGCTGCCCTCCTGGGCAATGGCC
TCATCATCACCGCCATAGCCTGCGACCACCGCCTCCACACCCCCATGTACTTCTTCCTC
CTTCTGAACCTCTCCCTCCTTGACCTGGGATCCATCTCCACTGTTCTCCCAAAAGCCAT
GGCCAATTCTCTGTTGGAGATCAGGGCCATGTCCTACTTGGGATGTGCTGCACAATTCT
TTTTCTTTTTCTTCTTGATTGGAGGAGAGTATTGTGTTCTGACTATCATGGCCTATGACC
GCTATGTTGCCATCTGCAAACCCCTGCACTACGGGACCCTCCTGGGCAGCAGAGCTTG
TGTCCACATGGCAGCAGCTGCTTGGGGCAGTGGCTTTCTCAACTCTCTTCTGCACACT
GCCAGTATATTGTCACTCCCACTCTGCCATGGCAGTGCCATGGAGCAGTTCTTCTGTGA
AATCCCCCAGATCCTCAAGCTCTCCTGCTCAGATGCCTACCTCAGGGAGGCTGGGCTTA
TTGTGGTCAGTTTGTGTTTAGCACATGGGTGTTTTGTTTTCATTGTGGTGTCCTATGTGC

AGATCTTCAGGGCTGTGCTGAGGATCCCCTCTCAGCATGGATGGCACAAAACCTTTTC
CACATGCCTCCCTCACCTGGCCATGGTCTCCCTCTTTATCAGCACTGCAGTGTTTGCCT
ACCTGAAGCCTTCCTCCATCTCCTCCACATCCTTGGATCACTTGTTGGCTGTTCTGTACT
CGGTGGTGCCTCCAGCAGTGAACCCCCTCATCTACAACATGAGGAACCAGGAGCTCAA
AGATGCCCTATAG
>Egret_OR14J1 (Egret4)
ATGTCCAACAGCAGCTACATCACCCAGTTCGTCCTTCTGGCCTTCGTGGACACACGGG
AGCTGCAGCTCTTGCACTTCTGGCTCTTCCTGGGCATCTACCTGGCTGCCCTCCTGGGC
AATGGACTCATCATCACCACCATAGCCTGCGACCCCCACCTCCACACCCCCATGTACTT
CTTCCTCCTCAACCTCTCCCTCATTGACCTGGCCTCCATCTCCACCACTGTCCCCAAAT
CCATGGCCAATTCCCTCTGGGACACCAGGACCATCTCTTATGCAGGATGTGCTGCCCAG
GTCTTCTTTGTATTCTTCTTCATAGCAGCAGAGTATTCTTTTCTCACTGTCATGGCCTATG
ACCGCTACGTTGCCATCTGTAATCCCTTGCATTATGGGACCCTCCTGGGCAGCAGAGCT
TGTGTCCACATGGCAGCAGCTGCCTGGGGCAGGGGGGTTCTCTATTCTCTCCTGCACA
CGGCCAATACATTTTCCATACCCCTCTGCAAGGGCAATGCTGTGGACCAGTTCTTCTGT
GAGATCCCCCAGATCCTCAAGCTCTCTTGCTCACTCTTCTACCTCAGGGAGGGTGGGCT
TATTGTGGCAAGTGTCTTTGTTGGGGTTGGATGTTTTATTTTCATTGTGCTGTCCTATGT
GCAGATCTTCAGGGCCGTGCTGAGGATCCCCTCTGAGCAGGGACGGCACAAAGCCTTT
TCCACGTGCCTCCCTCACATGGCTGTGGTCTCCCTGTTTCTCAGCACTGTAGTGTTTGC
CCACCTCAAGCCCCCTTCCATCTCATCCCCATCCCTGGATCTTGTAGTAGCAGTTCTGTA
CTCAGTGGTGCCTCCAGTAGTGAACCCCCTCATCTACAGCATGAGGAACCAGGAGCTC
AAGGAGGCCCTATCGAAACTGATCCAATGGGTGCTGTTTCACAGACAAAAGTCTCTGT
CACTTCCTCTGCGCTTTAGACTCTCCCAGATTTTGACTTAG
>Egret_OR14J1 (Egret5)
ATGTCCAACAGCAGCACCAGGTCTCAGTTCCTCCTCCTGGCATTTGCAGACATGAGGG
AGCTGCAGCTCTTGCACTTCTGGCTCTTCCTGGGCATCTACCTGGCTGCCCTCCTGGGC
AACGGCCTCATCATCACTGCTGTAGCCTGCAACCACCGCCACCACACCCCCATGTACTT
CTTCCTCCTCAACCTCTCCCTCTTTGACCTGGCCTCCATCTCCACCACTGTTCCCAAATC
CATGGCCAGTTCTCTGTGGGATAACAGGGCCATTTCTTATGCAGGATGTGCTGCACAGG
TATTACTCTTTACCTTTGTGGTTGTAGCAGAGATTTCTCTTCTCACCATCATGGCCTATGA
CCGCTATGTTGCCATCTGCCAACCCCTGCACTACGGGACCCTCCTGGGCAGCAGAGCT
TGTGTCCACATGGCAGCAGCTGCCTGGGGCAGTGGCTTTCTCAATGCTGTGCTGCACA
CGGCCAATACATTTTCACTACCACTCTGCCATGTCAATGTTTTGGACCAGTTCTTTTGTG
AAATCCCCCAGATCCTCAAGCTCTCCTGCTCAGATGCCTACCTCAGGGAAGCTGGGCT
CCTTGTGATTAGTGCATGTATAGTCTTTGGGTGTTTTGTTTTCATTGTGCTGTCCTATGTG
CAGATCTTCAGGGCTGTGCTGAGGATCCCTTCTCAGCAGGGGCAGCACAAAGCCTTTT
CCACGTGCCTCCCTCACCTGGCCGTGGTCTCCCTGTATGTCAGCACTGGCATATTTGCC
TACCTGAAGCCCCCCTCCATCTCCTCTCCTTTGCTGGACTTGGTGGTGGCAGTGCTGTA
CTCCGTGGTTCCTCCAACAGTGAACCCCCTCATCTACAGCATGAGGAACCAGGAGCTC
AAGGAGGCCCTATGGAAACTGGCTAAATGA
>Egret_OR14J1 (Egret6)
ATGTCCAACAGCAGCTCCATCACCCAATTTCTCCTCCTGGCATTTGCAGACACGTGGGA
GCTGCAGCTCTTGCACTTCTGGCTCTTCCTGGGCATCTACCTGGCTGCCTTCCTGGGCA
GTGGCCTCATCATTACTGCCATAGTCTCCGCCCCCCGCCTCCACACCCCCATGTACTTCT

TCCTCCTCAACCTCTCCCTCCTCGACCTGGCTTCCATCTCCACCATTGTCCCCAAATCAA
TGGCCAATTCACTTGGGGACAACAGAGCCATTTCCTATCAAGGGTGTGCGGTCCAACT
CTTTTACTTTTTCTTTTTTACATCAGCAGAGTTTTCTTTCTTAACTGTCATGTCCTATGAC
CGCTACATTGCCATCTGCAAACCCCTTCACCATGGGACCCTTCTGAGCAGCAGAGCTT
GTGTCCACATGGCAGCAGCTGCCTGGAGCGGTGGCTTTCTCAACTCTCTCCTGCACAC
GGCCAATACATTTTCACTCCCCCTCTGCCAAGGCAATGCTGTGGACCTGTTCTTCTGTG
AAATCCCTCAGATCATCAAGCTCTCCTGCTCAGACTCCTACCTCAGGGAAGTTGGGCTT
CTTGTGGTCAGTGCCTTATTAGGCTTTGGCTGTTTTGGTTTCATTGTGGTGTCCTATGTG
CAGATCTTCAGGGCCGTGCTGAGGATCCCCTCTCAGCAGGGACGGCACAAAGCCTTTT
CCACGTGCCTCCCTCACCTCTGTGTGGTCTCCCTCTTTATCAGCACCGGCACGTTTGCC
TACCTGAAGCCCCCCTCCATCTCCTCCCCATCTCTGGATCTGGTGATGGCAGTGCTGTA
CTCAGTGGTGCCTCCAGCAATGAACCCCCTCATCTACAGCATGAGGAACCAGGAACTC
AAGGATGCCCTATGGAAACTCTTTCATTGA
>Egret_OR5J2 (Egret7)
ATGAAGTACGGGACCGTGGCTAAAGACAACAGCACCCTGGTGACCCAGTTCGTCCTCC
TAGGGCTGACCAGCGAGCCCAAGCTGCAGACTCCTCTCTTCATAATCTTCTTAATGATT
TATCTCATCACCCTGGTGGGTAACCTTGGGCTGATCGCTCTGATCAAGACAAACCACCA
GCTGCACACTCCCATGTACTTTTTCCTCGGCAATCTGTCCATGGTTGATCTTTGCTACTC
CTCCGTCTTTTCTCCAAAGCTGCTCATTGGCTTCTTGGTGGAAAAGAAAACCATTTCCT
ACCCTGCCTGCTTTGCCCAGCATTTCTTTTTCCTTGCGCTTATCACCACGGAGGTGCTCT
TGCTGGCTGCGATGGCGTACGACCGCTACGTAGCCATTTGCAAGCCGCTGCTCTACGCT
ATTTCTATGCCCAAGAGGGTCTGCATTCAGCTGGTGGCCGGGTCATATGCGGGGGGGG
TTTTGAACTCACTGATCCAAACTTGTTGCTTGCTGCCGTTGCCTTTTTGTGGCCCCAAC
GTCATCAACCATTACTTCTGTGACACCAACCCGCTGCTGAAGCTCGCCTGCTCAGATGG
CCGCCCCAATGAGCTTTTGCTCGTAACCTTCAACGGAACCATTTCCATGTCCGTGCTCG
TCATCATCATCATCTCCTACGTTTACATCCTCCTCTCCATCCTGAGGATTAGGTCTGCCA
AGGAAGGCACAAAGCCTTCTCCACCTGCGCCTCCCACCTCCTGACCGTGACCTTGTT
CTACGTCCCCGCCGGGCTGAGCCACATGCAGCCGGGCTCCAGGTACTCGCTGGAGATG
GAGAAAGTCACCGCCGTGTTTTACACCCTGATGGTTCCTCTGCTCAACCCTCTGATCTA
CAGCTTGAGGAACAAGGAGGTCAAGGACGCGCTTCAAAAAACGACGGCGAATAACGT
TTTGGCAAGCTGCTTGCTGGCCAAACTGGCCCTGGGCAGTTGA
>Egret_OR5AU1 (Egret8)
ATGGAGGAAGGTAACGACACATTTGCATCTGAGTTTATTCTCCTGGGCTTCACGAACCG
AGATGACCTGCAGGTGACTTTCTTTGTCTTATTCCTTGCCATCTATGTGGTCACTCTAAT
AGGAAATCTGGGAGTAATTCTATTAATCAGAATCGATTCGTGCCTACGCAGCCCCATGT
ACTTCTTCCTAAGCCACTTGTCTCTCCTGGATGTCTGCTACTCCTCCACCATCATCCCTC
AAACCTTGCTGAATTTTTTAGTGGAGAAGAAGGCTATTTCCTTCGCTGGGTGTGCCACG
CAGCTCTTCTCCTTCGCCGCTTGTGCCACCGCCGAGTGCTACGTGCTGGCTGCCATGGC
CTATGACCGCTACGTGGCCATTTGCAACCCCCTGCTCTACCCTGTGGCCATGTCCCAGA
GCCTTTGCTTTGGGATGTTGGCTGGTGCCTACTTTGCTGGTGTGATCAGCTCCACCATA
CACACGGTTTCCATATTTCGCCTCCCGTTCTGCCAATCCAAGAGGATCAATCATTTCTTC
TGTGACGGCCCACCGCTGCTAGCCCTCTCCTGCTCTGACACCCGTGTCAATGAGGCGA
TGGTTTCTGCCGTGGTGGGGTTCAACGTGCTTAGCACCACGGTCTTCATTTTAGTCTCC
TACTTGTTGGTCCTCTCCGCCATCATGCGGATGCGCTCCGGGGCCAGTTGGCACAAAG

CCTTCTCCACTTGTGCCTCTCACTTGGTCTCCATCGCTTTGTACTACGGCAGCTCCCTCT
TCACGTACCTGCGCCCCGGCTCCAGACGCTCCTTGGAGCATGGCGAGGTGGTCTCCGT
GCTCTACTCCGTTGCAGTCCCCATGCTGAACCCACTCATCTACAGCCTGAGAAACACA
GACATGAAGAACGCCATGAGGAAAGCAAAAGGTAGAGTCCTCTCCTCCTTGTCCATCC
ATGGTTCCTGGTCAGCTGAAAGGAGAGGGATGCCCTTCCATGGAGAAGAGGGTTAG
>Egret_OR14J1 (Egret9)
ATGTCCAACATCAGCACCATCACCCAGTTCCTCCTCCTGGCATTTGCAGACACGCGGG
AGCTGCAGCTCTTGCACTTCTGGCTCTTCCTGGGCATCTACCTGGCTGCCCTCCTGGGA
AATGGCCTCATCATCACTGCCATAGCCTGTGACCACCGCCTCCACACCCCCATGTACTT
CTTCCTCCTCAACCTCTCCCTCCTCGACCTGGGCTCTATCTCCACTATTGTCCCCAAATC
CATGGCCAATTCCCTCTGGAACAACAGAGCCATCTCTTCCTTTGGATGTGCTGCCCAAG
TCTTTTGGTTTGTCTTTTTAGTTTTAGCAGAGTATTCTCTTCTCACTGTCATGGCCTATGA
CTGCTATGTTGCCATCTGCAAACCCCTGCACTATGGGACCCTCCTGGGCAGCAGAGCTT
GTGTCCACATGGCAGCAGCTGCCTGGGGTGGTGGATTTTTAAATTCTCTCTTTCACACT
GTCACTACATTTTCCATTCCCCTCTGCAAGGACAATTCTGTGGACCAGTTCTTCTGTGA
AATCCCCCAGATCCTCAAGCTCTCATGTTCACACTCCTACCTCAGGGAAGCTGGCCTGG
TTGTGGTCAGCAGTTTTCTTTTTTGGGGATGTTTCATTTTCATTGTGCTGTCCTATGTGC
AGATCTTCAGGGCCGTACTGAGGATCCCCTCTCAGCAGGGACGGCACAAAGCCTTTTC
CACGTGCCTCCCTCACCTGGTCGTGGTCTCCCTCTTTGTCAGCACTGGCACATTTGCCT
ACCTGAAGCCCCCCTCCATTTCTTCCCCATTCCTGGATCTGGTCATGGCAGTGCTTTACT
CCGTTCTGCCTCCAACAGTGAACCCCCTCATCTATAGCATCAGGAATCAGGAGCTCAA
GGATGCCCTGAGGAAACTGATCACACGCTTTTCAACAGCCATAGACTGTGTACCTTCCT
CCACAAATGACTCCCAGAGTCTGTCATGA
>Egret_OR14J1 (Egret10)
ATGCAGCAGATCAAGGATGTTCCTCCTTGGACAGGCCCCCATGTGCAGAGGGAACAAA
TATTCAATGGCAGCTCCATGAATGAGTTCCTCCTCTTGGTGTTTGCAGACACACGGGAG
CTGCAGCTCTTGCACTTTTGGCTCTTCCTGGGCATCTACCTGGCTGCCCTCCTGGGAAA
TGGACTCATCATCACCGCCGTAGCCTGCGACCACCGCCTCCACACCCCCATGTACTTCC
TCCTCCTCAACCTCTCCCTTATTGACCTGGGCTGCATCTCCACCATTCTCCCTAAAACCA
TGGCCAATTCTCTCTGGAACACCAGGGCCATTCCCTACTTGGGATGTGCTGCACAGGTC
TTTCTGTTTGCCGCCTTAATCTCAGCAGAGATTTACCTTCTCACAGTCATGGCGTATGAC
CGCTACGTGGCCATCTGCAAACCCTTGCACTATGGGACCCTCCTGGGCATCAAAGCTT
GTGTCCACATGGCAGCAGCTGCCTGGGGCAGTGGCTTTCTCAGTGCTGTGCTGCACAC
GACCAACACATTTTCCCTAACCCTCTGCCATGGCAATGCTGTGGACCAGTTCTTCTGTG
AAATCCCCCAGATCCTCAAGCTCTCCTGCTCAGACTCAGGCTACCTCAGGGTAGTTGG
CCTTCTTGTGGTTGGTGTTTGTTTAGCATTTGGGTGTTTTGTTTTCATTGTGGTGTCCTAT
ATGCAGATCTTCAGGGCTGTGCTGAGGATCCCCTCTCAGCAGGGACGGCACAAAGCCT
TTTCCACGTGCCTGCCTCACCTGGTTGTCATCTCCCTGTTTATCAGCACTGCCATGTTTG
CCTACCTGAAGCCCCCCTCTATCTCCTCCCCTTCCCTGGATCTGGCGGGTTCAGTTCTGT
ACTCAGTGGTACCTCCAGCAGTGAACCCCCTCATCTACAGCATGAGGAACAAGGACCT
CAAGGATGCCTTGAGAAAACTGATCCAATGGACTTTGTGCCATGCACAGAAATTTTCTA
CCCCTTCTCTGCAGCACTCCCAGAGTATATTTTAG
>Egret_OR52B2 (Egret11)
ATGGCAGCTCGCAATCAAACCAGCTTGCAGCCTGCCTCCTTCCTTCTGCTGGGCATGG

CAGGCCTGGAGGACCAGCACGCCTGGCTCTCCGTCCCCTTCTGCCTGATGTACATCAC
GGCGCTCCTCAGCAACCTCATCCTCTTAATTGCTATCGTGACAGAGCGAAGCCTCCACA
AGCCAATGTACCTCTTCCTGGCCATGTTAGCGGTGGCAGATCTCGTGTTGTCCTCCTCC
ACGGTGCCCAAAGCCCTGAGCGTATTCTGGTCCCTTTCCAAGGAGATATCTTTCCGCGC
CTGCCTTACCCAGATGTTCTTCATACACCTGAGCTTCGTTGCAGAGTCGACCGTTCTGC
TGGCCATGGCGTTTGACCGGTACGTGGCCATCTGCAGCCCCCTGCGATACGCCACAGT
GTTCACGCACTCAGTGGTAGCCAAGGTAGGGCTGGCTGCAATAGCCAGGAGCTTTTGT
GTGATGTTCCCAACGATATTCCTCCTTCAGAGGCTGCCATACTGCAGGCACAGGGTCAT
GCCTCACACCTACTGCGAGCACATGGGCATTGCCCGGCTGGCCTGCGCCGATATCTCCA
TTAACATCTGGTACGGCTTCGCCACCACCCTTCTGTGCCCAGGTGTGGACGTTGTGCTC
ATCGGGGTATCGTACGTCCTCATTCTCCGGGCTGTCTTCAGGCTCTCGTCCAAGGATGC
CCAGCGCAAGGCAGTTGGCACCTGCAGCTCCCATGCCTGTGTGATATTCGTGTTCTACA
CACCAGCATTTTTCTCATTTTTCACTCATCGCTTTGGCCACAACGTCCCCCACCATGTTC
ACATCCTGTTGGCGAATCTCTATGTGCTCCTGCCACCCATGCTAAACCCCATCATCTATG
CTATGAAAAACAAACTCATTCGAGAAAAAGTGTCCCAAGTACTCTTCAGGATGGTGCA
AGTGCGGTGA
>Egret_OR14A16 (Egret12)
ATGAATGAACTAAATTTTGCTCAGGAAACCTTCTCTGAATTTTTCTGGTTTTATCTCCTT
CGGCAGTTTCCACACCCAGAAAGCTCCATCACCCAGTTCCTCCTCCTGGCATTTGCAG
ACACACGAGAGATGCAGCTCTTGCACTTCTGGCTCTTCCTGGCCATCTACCTGGCTGCC
CTCCTGGGAAACGGCCTCATCATCACTGCCGTAACCTGTGACCACCACCTGCACACCC
CCATGTACTTCTTCCTCCTCAACCTCTCCCTCCTCGACCTGGGATCCATCTCCACCACTG
TCCCTAAAGTCATTGCCAACTCTCTGTGGGACACCAGGGCCATTTCCTATGCAGGATGC
GCTGCCCAAGTCTTTCTGTTTGCCTTCTTGGTTGGAGCAGAGATTTCTCTTCTCACCAT
CATGGCCTATGACCGCTACATTGCCATCTGCAAACCCCTGCACTACGGGACCCTCCTGG
ACAGCAGAGCTTGTGTCCACATGGCAGCAGCTGCCTGGGGCAGTGGGTTCATCACTGC
TGTGCTGCACACAGCCAATACATTTTCACTTCCCCTGTGCCAAGGCAATGCTGTGGACC
AGTTCTTCTGTGAAATCCCCAAGGTCCTCAAGCTCTCCTGCTCAGACACCTACATCAGG
GAAGTTTGGCTTCTTGTGGTCAGTGTCTGTTTAGGCTTTGGGTGTTTTCTTTTCATTGTG
GTGTCCTATGTGCAGATCTTCAGGGCTGTGCTGAGGATCCCCTCTCAGCAGGGACAAC
ACAAAGCCTTTTCCACGTGCCTCCCTCACCTGGCCGTGGTCTCTCTGTTGGTCAGCACT
GGCATGTTTGCCTACCTGAAGCCCCCTCCATCTCCTCCCCAGTTCTGGACATTGTGGT
TTCATTTCTGTACTCAGTGGTGCCTCCAGCAGTGAACCCCCTCATCTACAGCATGAGGA
ACAAGGAGCTCAAGACTGCACTGTGGAAACTGGCTCAATGGGTGCTGTTAACCCTGCC
TCCCCTTCTCTGCACATTTCCCAGAGTTTATCTTAATCCATAA
>Egret_OR14J1 (Egret13)
ATGTCCAACAGCAGCTCCATTACCCAGTTCCTCCTCCTGGCATTTGCAGACAAGCGGG
AGCTGCAGCTCTTGCACTTCTGGCTCTTCCTGGGCATCTACCTGGCTGCCCTCCTGGGC
AACAGCCTCATCATCACCGCCATAGCCTGTGACCACCGCCTCCACACCCCCATGTACTT
CTTCCTCCTCAACCTCTCCCTCATTGACCTTGGATCTGTCTCCACCAGTATCCCCAAATC
CATGGCGAATTCTCTGTGGGAGACCAGGGCCATCACCTACCAAGGTTGTGCTGCCCAG
ATCTTTATGCTTGCCTTCTTAATCTCAGCAGAATTTTCTCTTCTCACTATCATGGCCTATG
ACCGCTACGTGGCCATCTGCAAACCCCTGCACTACGGGACCCTCTTGGGCAGCAGAGC
TTGTGTCCACATGGCAGCAGCTGCCTGGGGCAGTGGCTTTCTCACTGCTGTGCTGCAC

ACGGCCAATACATTTTTACTCCCCCTCTCTGTGGACCAGTTCTTCTGTGAAATCCCTCA
GATCCTCAGGCTCTCATGCTCAGACTCCTACCTCAGGGAAGTTGGGCTTCTTGTGGTTA
CTGCCTGTTTAGTCTTTGGCTGTTTTGTTTTCATTGTGCTGTCCTATGTGCAGATCTTCA
GGGCTGTGCTGAGGATCCCCACTCAGCAGGGGCGGCACAAAGCCTTTTCCATGTGCCT
GCCTCACCTGGCCGTTGTCTCCCTGTTCGTCAGCACTGCTTTGTTTGTCTCCCTGAAGC
CTCCCTCCATCTCCTCATCCCTGGATTTGGCGGTGGCAGTGCTATACTCGATTTTGCCTC
CAGCAGTGAACCCCCTCATCTACAGCATGAGGAACAAGGAAATCAAAGCAGCTCTGC
AGAAACTATTTGAATATGGGCTTCTACAGTATCAGTAA
>Egret_OR5AR1 (Egret14)
ATGGTCAGAAGAAATGAGACAACTGTGGATGAGTTCATTCTCTTGGGAATCACAGATAT
TTGGGAGCTGCAGGTCATTCTCTTTCTGCTGTTCCTTCTGATCTGCCTCACCTCATTGGT
GGGGAATCTTGGCATGATTGCATTAATCAGGCTTGACTCTCGACTCCACACCCCCATGT
ACTTCTTCCTCTGCCACCTCTCTCTGGTAGACCTAGGTAATTCCTCAGCAGTTGCTCCC
AAAATGCTAGTGAGCTTCTTTGAAGAAAGGAAGCCCATCTCTCTGCCAGGCTGTGCAG
CCCAAATGTACTTTTGTGGCGTCTGCATAATCACCGAGTGTTACCTGCTGGCTGCCATG
GCCTACGACCGGTACGTGGCCATCTGTAACCCTCTGCTCTATGTGGCCACCATGTCTCA
AAAGGTTTGTGTCCAACTGGCCGTGGGATCCTACATAGTAGCTACTGTGAATGAAATAA
TGCTTGTCAGCTCGGTGTTCAGCCTGCACTTCTGTGGCCCTAAGGTCATCAATCACTTC
TTCTGTGACATTCCTCCGGTCCTGAAACTTTCCTGCTCCAGTACTGCTGTCAACGAGCA
CATGCTTTTTACCATCGCTGCATTTATTGCACTCAGCACTTTGGGATTCATTGTTGTCTCT
TATGGTTATATCCTTACCGCTGTCCTGAGGATCTGCTCCCCAGAGGGCAGGCACAAAGC
TTTCTCCACCTGTGCCTCACATCTGACATCAGTCTCGGTTTTTTATGGGACTATGATCTT
CATGTACCTCCGCCCCAGTTCTAGCTACTCCCTGGACCAGGACAAGGTGGTATCCGTTG
CCTACACCCTGGTGATTCCCATGCTGAACCCCCTGATCTACAGCCTGAGGAACATGGA
GGTGAAGGATGCTCTCAAGAGACTCCTAGGAAAAGTTCGTGTTTATTTTAGAAATCAA
ACTGGTAAAGAGGTGTCATAA
>Egret_OR14J1 (Egret15)
ATGTCCAACAGCAGCTCCATCACCAAGTTCCTCCTCCTGGCATTTGTAGACACACAGG
AGCTGCAGCTCTTGCACTTCTGGCTCTTCCTGGGCATCTACCTGGCTGCCCTCCTGGGA
AATGGCCTCATCACCCCCGCCATAGCCTGTGACCACCGCCTCCACACCCCCATGTACTT
CTTCCTCCTCAACCTCTCCCTCCTCGACCTGGCCTCCATCTCCACCACTGTCCCCAAAT
CAATGGCCACTTCCCTCTGGGACACCAGGACAATTTCCTATGCAGGATGTGCTGCCCA
AGTATTTCTGTTTTCCTTCCTGATTACAGCAGAGTTTTCTCTTCTCACTGTCATGGCCTAT
GACCGCTACATGGCCATCTGCAACCCCCTGCACTACGGGACCTTCCTGGGCAGCAGAC
CTTGTGCCAATATGGCAGTGGCTGCCTGGGGCAGTGGATTTCTCCACGGTCTGCTGCAC
ACGGCCAATACATTTTCACTACCCCTCTGCCAAGGCAACGCCATCAACCAGTTCTTCTG
TGAAGTTCCCCAGATCCTCAAGCTCTCCTGCTCAGATGCCTACCTCAGGGAAGTTGGG
GTTATTCTGGTTGGTGTCTGTTTAGCATCCGGGTGTTTTATTTTCATTGTGCTGTCCTATG
TGCAGATCTTCAGGGCTGTGCTGAGGATCCCCTCTCAGCAGGGGAGGCACAAAGTCTT
TTCCACGTGCCTCCCTCACCTGGCCGTGGTCTCCCTGTTTACCAGCACTGCCATGGCTG
CTAGCCTCAAACCCCCCTCCTTTGCCTCCCCACCCTTGGAGCTCCTGATGGCTGTTCTG
TACTCAGTGGTGCCTCCAGCAGTGAACCCCCTCATCTACAGCATGAGGAACCAGGAGC
TCAAGGATGCCATCAGGAGGGTGGTTTCAGAGATGTTTCTCACTAGTGATAAATTTCCC
TCCTCTCTGCACAAATGA

>Egret_OR52K1 (Egret16)
ATGCCCCACTCATGTCCCACCAACACCTCGCTGCCAGAGCTGCACCTGACGGGCGTCC
CGGGGCTGCAGCACCTGCACCACTGGATCTCCATCCCCTTCTGCATCATGTACCTCGTC
ACGCTGGCCGGGAACAGCACCCTCCTGTGCGTGATAAGGGCTGATCCTCGCCTGCACA
ACCCCATGTTCCTCTTCCTGTCCATGTTGGCTGTCATTGACCTGGTGATGTCCACCTCCA
TCACCCCCAAAATGCTGGGCATCTTCTGGTTTCAATCCACTGCCATCAGCCTGGATGCT
TGTCTTACCCAGATGTACTTTGTTCATGCTTTCTCTGTGATGGAGTCAGGGGTGCTGGT
GGCAATGGCCTTTGACCGCTACGTGGCCATTTGCAAGCCACTGCGGTACTTGTCCATCC
TGACCAGCCCCGTTGTGGCTGCCATTGGCTTGGCTACCTTGCTCAGGGCTGTGGTTTTC
ATGAGCCCCCTCACCTTCCAGATTCACCGCCTGCCTCTCTGCAGCCCGGCAGTCGTGG
ACCACTCGTACTGCGAGCACATGGCCGTGCTCAAACTGGCCTGTGGGGACGCTGCCTT
CAGCAACACCTACAGCCTCTCCATCTCCACCTACGTGGGCAGCTTCGACTCGCTGCTCA
TCGCCCTCTCCTATGCGCTCATCCTCCGAGCTGTGCTCAGCCTTTCCTCCCCACAAGCC
CGCAAAAAAACCTTCAGCACCTGCGGCTCACACCTCTGCATCATGGCCCTCTTCTACAT
CCCTGGGCTGCTCTCCATGTACATGGAGAGGTACCACCAGGAGCTCCCACCCCACGTC
CAGGTCCTGTTGGCTGATCTCTACCTCCTCATCCCACCGGCATTCAACCCCCTGATCTA
CGGCATCAGGATGAAGCAGATTCGTGATGGAGCACGCAGGGCGATCTCCCGGAGAAG
ACCCGTGGCAGGAGGGATTGGGCCCGGCCTTCAGGACACAGGGCTGGATCTCGTGAA
GACAAAGTCCATTCCCTAG
>Egret_OR14I1 (Egret17)
ATGTGCAACAACAGCTCCATCACCCAGTTCCTCCTCCTGGCATTCGCAGACACGTGGG
AGCTGCAGCTCTTGCACTTCTGGCTCTTCCTGGCCATCTACCTGGCTGCCCTCCTGGGC
AACAGCCTCATCATCACTGCTGTAGTCTGCAACCACCGCCTCCACACCCCCATGTACTT
CTTCCTCCTCAACCTCTCCCTCCTTGACCTGGGATCCATCTCCACCACTGTCCCCAAAT
CCATGGCCAATTCCCTCTGGAACACTAGGGCCATCTCCTATGCAGGATGTGCTGCACAG
GTTTTTCTCTCTGTCTCTTTTATTTCAGCAGAGTATTTTCTCCTCACCGTCATGGCCTATG
ACCGCTATGTTGCCATCTGCAAACCCCTGCACTACGGGACCCTCCTGGGCAGCAGAGC
TTGTGTCCACATGGCAGCAGCTGCCTGGGGCAGTGGGTTCTTCTATGCTGTGCTGCCCA
CGGCCAATACATTTTCCATTCCCCTCTGCCAAGGCAATGCTGTGGACCAGTTCTTCTGT
GAAATCCCCCAGATCCTCAAGCTTGCCTGCTCAGAGTCCTACCTCAGAGAACTTCGAC
TTCTTGCATTTAGTGCCTTTCCCTTTTTGGGGTGTTTTGTTTTCATTGTGGTGTCCTATGT
GGAGATCTTCAGGGCCGTGCTGAAAATCCCCTCTCAGCAGGGACGGCACAAAGCTTTT
TCCACATGCCTTCCTCACCTAGCTGTGGTCTCCCTGTTTATCAGCATCTCTTTTTTTGCCT
ACCTGAAACCCCGCTCCATCCTCTTCCCAGCTCTGGATCTGGTTATGGCAGTGCTGTAT
TCAGTGGTTCCTCCAGCACTGAACCCCCTCATCTACAGCATGAGGAACCAGGATCTCA
AGGATGCAGTGTGGAAACTGGTGACTGAATGA
>Egret_OR14J1 (Egret18)
ATGTCCAACACCAGCTTCATCACGCAGTTCCTCCTCCTGGCATTTGCAGACAAGCGGG
AGCTGCAGCTCTTGCACTTCTGGCTCTTCCTGGGCATCTACCTGGCTGCCCTCCTGGGA
AACAGCCTTGTCACCAGTGCTATAGCCTGCGACCACTGCCTTCACACCCCCATGTACTT
CTTCCTCCTCAACCTCTCCCTCCTTGACCTGGGATCCATCTCCACCATTGTCCCCAAAG
CTATGGCCAGTTCCCTGTGGGATACCAGGGCCCTCTCTTTCTTGGGATGTGCTGTTCAG
GTCTTTCTGTTTCTCTTCCTCATTTCAGCAGAGTTTTCTCTTCTCACCATCATGGCCTATG
ACCGCTATGTTGCCATCTGCAAACCCCTGCACTACGGGACCCTCCTGGGCAGCAGAGC

TTGTGTCCACATGGCCGCAGCTGCCTGGGCCTGTGGGTTCCTCAATTCTCTTGTGCACA
CGGCCAATACATTTTCACTACCACTCTGCCATGACAACGCTCTGGACCAGTTCTTCTGT
GAAATCCCCCAGATCCTCAAGCTCTCCTGCTCAGATGCCTACCTTAGGGAGTTTGGGCT
TATTGTGGTCAGTGCCTGTTTAGGCGTCGGGTGTTTTATTTTCATTGTGCTGTCCTATGT
GCAGATATTCACATCCGTGTTTATGGTCCCCTCTCAGCAGAGACGGCACAAAGCCTTTT
CCACGTGCCTCCCTCACCTGGCTGTGGTCTCTCTGTTCATCAGCACTACCATATTTACCT
ATCTGAAGCCCCCCTCCATCTCCTCCGCAGTTCTCGATCTGGTTATGGCAGTGCTGTAC
TCAGTTGTGCCTCCAGCAGTGAACCCCCTCATCTACAGCATGAGGAACAGGGAGCTCA
AGGATGCAGTGTGGAAGCAGATGATGGGATGTTTTTAA
>Egret_OR14A16 (Egret19)
ATGTCCAATAGCAGCTCCACCACCAAGTTTCTCCTCCTGGCATTCACAGACACATGGGA
GCTGCAGCTCTTGCACTTCGGGCTCTTTCTGGGCATCTACCTGGCTGCCCTCCTGGGCA
ATGGACTCATCATCACCGCTGTAGCCTGTGACCACCACCTCCACACCCCCATGTACTTC
TTCCTCCTCAACCTCTCCCTTGTCGACCTGGGATCCCTCTCCACAACTGTCCCCAAATC
AATGGCCAACTCCCTGTTGGATACCAGGACCATCTCTTTCTTTGGATGTGCTGCTCAAG
TCTTTTGGTTTCTCTTTTTAGTTGTAGCAGAGTATTGTCTTCTCACTGTTATGTCCTATGA
CCGCTACATTGCCATCTGCAGACCCCTGCACTATGGAACCCTGCTGGGCAGCAGAGCC
TGTGTCCACATGGCAGCAGCTGCCTGGGGCAGTGGCTTTCTCTATGCTGTCCTGCACAC
GGCCAATACATTTTCACTTCCCCTCTGCAGAAGCAATGCCATAGACCAGTTCTTCTGTG
AAATCCCCCAGATCCTCAAGCTCTCCTGCTCAAATGCCTACCTCAGGGAAGTTCGGCTT
CTTGTGGTTAGTGGCATTTTATTCTTTGGGTGTTTTGTTTTTATTGTGGTGTCTTATGTGC
AGATCTTCAGGGCTGTGCTGAGGATCCCCTCTCAGCAGGGGCGACACAAAGCCTTTTC
CACGTGCCTCCCACACCTTACTGTGGTCACCCTGTTTATCAGCACCTCATTTTTTGCCTA
CCTGAAGCCCCCTTCCATCTCCTCCCCATCCCTGGATCTGGTGATGGCAGTGCTGTACT
CTGTTGTACCTCCAGCAGTGAACCCCCTCATCTACAGCATGAGGAACCAGGATCTGAA
GCGTGCCTTGAGGGACCTATGTGACTTCAGCATCCATAATGTGTCCATTATTTTTATAGG
ACTTCGTTGTGGTTCAACCTTGCCTGGCAACTAA
>Egret_OR14J1 (Egret20)
ATGTCCAACAGCAGCTCCATCACCCAGTTCCTACTCCTGGCATTCATGGAGACACGGG
AGCTGCAGCTCTTGCACTTCTGGATCTTCCTGGGCATCTACCTGGCTGCCCTCCTGGGA
AACGGCCTCATCATCACCGCTGTAGCCTGCGACCACCGCCTCCATACCCCCATGTACTT
CTTCCTCCTCAACCTATCCGTCATTGACCTGGCCTCCATCTCCACCACTATTCCTAAATC
CATGGCCAATTCCCTCTGGGACACCAGCGCAATTTCCTATTTGGGATGTGCTGTCCAGC
TCTTTCTCTTTACCTTCTTCGTAGCAGCAGAGTATTGTCTTCTCACCGTCATGGCCTATG
ACCGCTACGTTGCCATCTGCAAACCCCTGCACTATGGGACCCTCCTGGACAGCAGAGC
TTGTGCCCACATGGCAGCAGCTGCCTGGGGCAGTGCTTTTATCAATTCCTTCCTTAACA
CTGCCACTACATTTTCCTTACCCCTCTGCAAGGGCAATGCTGTGGACCAGTTCTTCTGT
GAAATCCCCCAGATCCTCAAGCTCTCCTGCTCAGACGCCTACTTCATTGAAGTTGCGCT
TCTTGTGGTCAGTGTGTGTTTAGCACTTGGCTGTTTTTTGTTCATTGGGCTGTCCTTTGT
GCAGATCTTCAGGGCTGTGCTGAGGATCCCCTCTCAGCAGGGACGGCACAAAGCCTTT
TCCACGTGCCTCCCTCACCTGGCCGTGGTTTCCCTCTTTATCAGCACTGTCATGTTTGCC
TACCTGAAGCCCCCCTCCATCTCCTCTCCTTTGCTGGACTTTGTGGTAGCAGTGCTGTA
CTCAGTGGTGCCTCCAGCATTGAACCCCCTCATCTACAGCATGAGGAACCAGGAGCTC
AAGGATGCACTGAAAAAGCTGATTCCATCAGTAGTATTTCTTCAGAAGTAA

>Egret_OR14A16 (Egret21)
ATGGCATTCGCAGGCACACGGGAGCTGCAGCTCTTGCACTTCTGGCTCTTCCTGGGCA
TCTACCTGGCTGCCCTCCTGGGCAACGGCCTCATCATCACCGTCATAGCCTGCAACCAC
CGCCTCTACACCCCCATGTACTTCTTCCTCCTCAACCTCTCCCTCCTCGACATGGGATCC
ATCTCTACCACTGTCCCCAAATCTATGGCCAATTCCCTCTGGGATACCAGGGCCATCTCT
TACCAAGGATGTGCTGCTCAAGTTTTTATGATTGCCTTCTTAATCTCAGCAGAGTTTTCT
CTTCTCACCATCATGGCCTACGACCGCTATGTTGCCATCTGCAAACCCCTGCACTACAG
GACCCTCCTGGGCAGCAGAGCTTGTGTCCACATGGCAGCAGCCGCCTGGGGCACTGG
GTTCTTCTATGCTGTGCTGCACACGGCCAATACATTTTCCATACCTCTCTGCCAAGGCAA
TGCCCTGGACCAGTTCTTCTGTGAAATCCCCCAGATCCTCAAGCTCTCCTGCTCAGATG
CCTACCTCAGAGAAGATGGCCTTCTTGTGGTTAGCATCTGTTTAGGCTTTGGGTGTTTT
GTTTTCATTGTGGTGTCCTATGTGCAGATCTTCAGGGCCGTGCTGAGGATCCCCTCACA
GCCGGGACAGCACAAAGCCTTTTCCATGTGCCTCCCTCACCTGGCCGTGGTCTCCCTG
TTTATCAGCACTGGCACTTTTGCCTACCTGAAGCCCCCATCCATCTCTTCCCCATCCCTG
GACATGGGGGTTTCATTTCTGTACTCAGTGGTGCCTCCAGCATTGAACCCCCTCATCTA
CAGCGTGAGGAACAGGGAGCTCAAGGATGCAGTGTCAAAACTGATAACTGGGTGGTT
TTAA
>Egret_OR14J1 (Egret22)
ATGTCCAATGCCAGCTTGATCACCCATTTCCTCCTCCTGGCATTCGCAGACACACGGGA
GCTGCAGCTCTTGCACTTCTGGCTCTTCCTGGGCATCTACCTGGCTGCCCTCCTGGGCA
ACGGCCTCATCATCACTGCCATAGCCTGCGACCACCGCCTCCACACCCCCATGTACTTC
TTCCTACTCAACCTATCCCTTGTTGACCTCGGCTCCATCTCCACCACCATCCCTAAATCC
ATGGCCAATTCTTTCTGGGGCACCCAGGACATCTTCTACCGAGGGTGTGTGGCCCAGC
TCTTTTTCTTATTCTTTTTTATGTCAGTTGAGTATTTTCTTCTCACTGTCATGGCATATGAC
CGCTACATTGCCATCTGCAAACCCCTGCACTACGGGACCCTCCCGGGCAGCAGAGCTT
GTGTCCACATGGCAGCAGCTGCCTGGGGCAGTGGGTTACTCTATGCTGTCCTGCACAC
GGCCAATACATTTTCACTTCCCCTCTGCAAGGGCAATGCTGTGGACCAGTTCTTCTGTG
AAATCCCCCAGATCCTCAAGCTCTCATGCTCACACTCCTACATCAGGGAGGCTGGGCTT
ATTGTAGTCAGTGTGTGTTTAGGTTTTGGGTGTTTTCTTTTCATCATGGTGTCCTATGTG
CAGATCTTCAGGGCTGTGCTGAGGATCCCCTCTCAGCACGGACGGCACAAAGCCTTTT
CCACGTGCCTCCCTCACCTGGCTGTGGTCTCCCTGTTTCTCAGCACCGGCACGTTTTCA
CACCTGAAACCCCCCTCTGTCTCCTCCTCCTCATCCCTGGACCTGGTGGTGGCAGTCCT
GTACTCAGTGGTTCCTCCAGCAGTGAACCCCCTTATCTACAGCGTGAGGAACCATGAG
CTCAAGGACGCCCTATGGAAACTGGCGCAATGGATGCAGTATCACCAACAATAA
>Egret_OR14A16 (Egret23)
ATGTCCAACAGCAGCTCCATCACCCAGTTCCTCCTCCTGGCATTCACAGACACGCGGG
AGCGGCAGCTCTTGCACTTCTGGCTCTTCCTGGGCATCTACCTGGCTGCCCTCCTGGGC
AACGGCCTCATCATCACCGCCATAGCCTTTGACCACCACCTCCACACCCCCATGTACTT
CTTCCTCCTCAACCTTTCCATCCTTGACCTGGCCTCCATCTCCACCACTCTCCCCAAATC
CATGGCCAATTCCCTCTGGAACACCAAGGCCATTTCCTTCTCAGACTGTGCTGCCCAG
GTCTTTCTGTATCTCTTTTCAATGGGGGGAGAGTATTTTCTTCTCTCCGTCATGGCCTAC
GACCGCTACGTTGCCATCCTCCAACCCCTGCACTATGAGACCCTCCTGGGCAGCAAAG
GTTGTGTCCGCATGGCCGTAGCTGCCTGGGGCAGTGGCTTTCTCACTGCTCTGCTGCAC
ACAGCCAACACATTTTCACTCCCCCTCTGCAAGGGCAATGCTCTCAACCAGTTCTTCTG

TGAGCTTCCCCAGATCCTCAAGCTCTCCTGCTCAGACTCGGGCTACCTCAGGGCAGTT
GGGCTTGTCATGCTTACCGCTTTTTTTGCGCTCAGTTGTTTTGTGCTCATCGTGCTGTCC
TATGTGCAGATCTTCAGGGCCGTGCTGAGGATCCCCTCTCAGCGGGGTCGGCACAAAG
CCTTTGCCACGTGCCTCCCTCACCTGGCTGTGGTCTCCCTGTTTCTCAGCACAGGCATC
TTTGCCGGCCTCAAGCCCCACTCCATCTCCTCCCCATCCCTGGATCTGGTGGTGACTGT
TCTCTACTCGGTGGTGCCTCCAGTAGTGAACCCCCTCATCTATAGCATGAGGAACCAGG
AGCTCAAGGACGCCATCAGGAAAGTGGTTTCCTGGATGTTTTTCAGTAATTCAAAAGT
TCTCAGCACTCTCCAAAAATGA
>Egret_OR14A16 (Egret24)
ATGTCCAACACCAGCTCCATCGCCCAGTTCTTCCTTCTGGCATTCACAGACACACGGG
AGCTGCAGCTCTTGCACTTCTGGCTCTTCCTGGGCATCTACCTGGCTGCCCTCCTGGGC
AACTGTCTCATCATCACCGCCATTGCCTGCGACCACCACCTCCACACCCCCATGTACTT
CTTCCTCCTCAACCTCTCCCTCCTCGACCTGGGCTCCATCTCCACCGCTCTCCCCAAAT
CCATGGCCAATTCCCTCTCAGACAGCAGGGCCATTTCTTACACAGGATGTGCTGCCCAA
GTATTTCTCTTTGTATTTTTTTTTTCAGCAGAATTGTATCTTCTCACCGTCATGGCCTATG
ACCGCTACGTTGCCATCTGCAAACCCCTGCACTACGGGACCCTCCTGGGCAGCAGAGC
TTGTGCTCACATGGCAGCAGCTGCCTGGGGCTGTGGGTTCCTCAATTCTGTCCTGCAC
ACTGCAAATACATTTTCCATACCCCTCTGCCAAGGCAATGCTGTTGACCAGTTCTTCTG
TGAAGTGCCCCAGATCCTCAAGCTCTCCTGCTCAGACAGTTATCTCAGGGAATTTTGGC
TTATGTTGTTTAGTGTATTTTTTGTCTTTGGGTGTTTCTTTTTCATCATGCTGTCCTATGTG
CAGCTTTTCAGGGCTGTGCTGAGGATCCCCTCTCAGCAGAGACGGCACAAAGCCTTTT
CCATGTGCCTCCCTCACCTGGCCGTGGTCTCCCTGTTTCTCAGCACTGCAGTGTTTGCC
CACCTGAAGCCCCCCTCCATCTCATCCCCATCTCTGGACCTCGTGGTGGCCATGCTGTA
CTTGGTGGTGCCTCCAGCAGTGAACCCCCTCATCTACAGCATGAGGAACAAGGAAATC
AAGGTTGCCCTGTTGAAACTGATGCAATGGGTGCGGTTTCACTGA
>Egret_OR14J1 (Egret25)
ATGTCCAACAGCAGCTCCATCACCCAGTTCCTCCTCCTGGCATTTGCAAATACACGGGA
CCTGCAGCTCCTGCACTTCTGGCTCTTCCTGGGCATCTACCTGGCTGCCCTCCTGGGCA
ATGGCCTCATCATCACCGCTGTAGCCTGTGACCACCGCCTCCACACGCCCATGTACTTC
TTCCTCCTCAACCTCTCCATAATCGATCTGAGCTTCATCTCCACCACTGTCCCCAAATCC
ATGGCCAATTCCCTCTGGTACACCAGGGATATTTCCTACTTGGGATGTGCTACCCAGGT
CTTTCTCTTTGTCTTTCTCATTGTTGCAGAATTGTATCTCCTCACGGTCATGGCCTACGA
CCGCTACGTTGCCGTCTGCAAACCCCTGCACTACGGCACCCTGCTGGGCAGCAGAGCT
TGTGCCCACATGGCAGCAGCTGCCTGGGGCAGTGGGTTCCTCCATGCCACCTTGCACA
CTGCCAACACATTTTCACTACCCCTCTGCCAAGGCAATGCCATAGACCAGTTCTTCTGT
GAAATCCCCCAGATCCTCAGGCTCTCCTGCTCAGCTGACTACCTCAGGGAAGTCGGGC
TTATTGTGTTTGGTGTCTGTTTAGCATTTGTTTGTTTTATTTTCATCATGCTGTCCTATGTG
CAGATCTTCAGGGCCGTGCTGAGGATCCCCTCCGAGCAGGGACAGCACAAAGCCTTTT
CCACGTGCATCCCTCACCTGGCCGTGGTCTTCCTGTTTGTCAGCACTGCCATGGTTGCT
GGCCTCAAACCTCCCTCCTTTTCCTCCCCATCCCTGGATCTGGTGATGGCTGTTCTGTAC
TCAGTGGTGCCTCCAGCAGTGAACCCCCTCATTTACAGCATGAGGAACCAGGAGCTCA
AGGATGCCATTAGGAAAGTGCTTTCAGGCTTTTACTCTGCTTATCGTTATCGTCATCAGT
ATCTGTGTTGTGGTTTGTGTCTATGA
>Egret_OR14A16 (Egret26)

ATGTTCAACAGCAGCTCTATCACTGAATTCCTCCTCCTGGCATTCGCAGACACACGAGA
GCTGCAGCGCTTGCACTTTTGGCTCTTCCTCGGCATCTACCTGGCTGCCCTCCTGGGAA
ACGGCCTCATCATCACCACCATAGCCTGCGACCACCGCCTCCACACCCCCATGTATTTC
TTCCTATTAAACCTCTCCCTTGTTGACCTGGGATCCATCTCCGCCACTATCCCCAAAGCC
ATGGCCAATTCCCTCTGGGACACAAGGGTCATCTTCTACCAAGGATGTGCTGCCCAGCT
CTTTTTCTTTTTCTTTTTTATGTCAGCAGAGTATTTTCTTCTCAATGTCATGGCCTATGAC
CGCTATGTTGCCATCTGCAAACCCCTGCACTATGGGACCCTGCTGAGCAGCAGAGCTT
GTGTCCACATGGCAGCAGCTGCCTGGGGCTGCGGGTTCCTCAATTCTCTGGTGCACAC
GGTCAATACATTTTCACTCCCCCTCTGCAAGGGCAATGCCATGGACCAGTTCTTCTGTG
AAATCCCCCAGATCCTCAAGCTCTCCTGCTCAGATGCCTACCTCAGGGAGGCTGGCTTT
ATTCTGCCTGGTGTCTTTATTGCATTTGGGTGTTTTGTTTTCATTGTGCTGTCCTATGTGC
AGATCTTCAGGGCTGTGCTGAGGATCCCCTCTCAGCAGGGACGGCACAAAGCCTTTTC
CACGTGCCTCCCTCACCTGGTCGTGGTCTCCCTCTTTGTCAGCACTGGATTGTTCGCCT
ACCTGAAGCCTGCCTCCATCTCTTCCCCATCCCTGGATCTGGTGGTGTCAGTTTTGTAC
TCGGTGGTGCCTCCAGCAGTGAACCCACTCATCTACAGCATGAGGAACCAGGAGCTCA
AGGAGGCCCTATGGAAACTGGCCCAATGGACGCTGTTTCACTGA
>Egret_OR10K2 (Egret27)
ATGGAGAGGGACAATAAGAGCAGTGATGCAAGCATGGAGTTCCTCCTGCTCGGCTTCT
CCGAGCTGCTCTGTCTGCGGGCCCTCCTCTTCCTTATCTTTCTCATTGTCCATTTGGCCA
CGTTGGCAGGGAATGTGATGATCTTCATGGCAGTGGTTATGGAGCCTTCCCGTCCTCCC
ATGCTTTTCTTCCTCTGTCAGCTCTCTGTCATTGAGCTCTGCTATACCTTAGTCATTGTCC
CTAAGGCACTCCTCAGCCTGGCAGTGGCGGATGGCAGCACCATTTCTTTCATAGGCTGT
GCTGCGCAGATGCACCTTTTTGTGGCCCTCGGTGGGGCTGAATGCTTCCTCCTGGTAGC
CATGTCGTACGACCGCTACGTTGCCATCTGCCAGCCGCTTCACTACGTAGCTGTGATGA
GCGAGGGGCTCTGCCTCAGGCTGGCTGTGGCATGCTGTCTGGGAGGCTTTGCTGTTGC
CCTGGTGTTGACAGTGGCTGTTTTCCGCTTACCTTTCTGCCAGTCGCATCGTATCAACC
ACTTCTTCTGTGACGTCCCTGCTGTGCTGCACCTGGCCTGTACCCAGAGTTATGCCCGT
GAGCTACCCTTGCTGGCTGCCTGTGTGCTCCTCCTGCTGCTCCCCTTCCTCCTGATCCT
GACCTCGTATCTTTGCATTGCCACTGCTTTGTTACATGTCACCTCCTCTGTGGGAAGGG
GCAAGGCCTTTTCCACCTGCATTTCGCACTTGGCCATCACCTTACTACACTATGGATGT
GCCACCTTCGTGTACATTCGTCCTAAGTCCAGTTATTCACCAGCTCGAGACAAGATGGT
GTCTCTGGTCTACACCAACATTACTCCATTACTGTATCCCCTCATTTATAGCCTGAGGAA
CAAGGAAATCAGAGGGGTCCTTGGGAAAATGTTGAGGAGGAAGAATATAGCTCAGCT
GAACGGGGATACTCTCAGAGATGTGATGTGTGTGTGTGCTAAATTCTAG
>Egret_OR14J1 (Egret28)
ATGTCCAACAGCAGCTCTGGCACTGGCTTCCTCCTCCTGGCAATCACAGACACACAGG
AGCTGCAGCTCTTGTGCTTCTGGCTCTTCCTGGGCATCTACCTGGCTGCCCTCCTGGGC
AACGGCCTCATCATCACCACCATAGCCTGTGACCACCACCTCCACACCCCCATGTACTT
CTTCCTTCTCAGCCTCTCCCTTCTTGACCTGGGATCCATCTCCACTATTGTCCCCAAATC
CATGGCCAATTCCCTCTGGGACTCCAGAGCCGTATCCTACGCAGGATGTGCTGTCCAGG
CCTTCTTTGTATTTTTTTTGGTAACAGCAGAGTATTTTCTTCTCACTGTCATGGCCTATGA
CCGCTACGTGGCCATCTGCAAACCCCTGCACTACGGGGCCCTCCTGGGCAACAGAGCT
TGTGTCCACATGGCAGCAGCTGCTTGGGGCAGTGGTTTTCTCAATGGCCTCCTGCACA
CGGTCAATACATTTTCACTGCCCCTCTGCAGAGGCAATGCTGTGGGCCAGTTCTCCTGT

GAAATCCCCCAGATCCTCAAGCTCTCCTGCTCACACTCCTACCTCAAGGAATTTGGGCT
TCTTGTGGTTAGCCTCTGTTTAGCACTTGGCTGTTTTGTTTTCATTGTGCTGTCCTATGT
GCAGATCTTCAGGGCCGTGCTGTGGATCCACTCTCAGCAGGGACGGCACAAAGCCTTT
TCCACGTGTGTCCCTCACCTGGCCGTGTCCTCCCTGTTTGTCAGCACTGGCACATTTGC
CTACTTGAAGCCTCCCTCCATCTCCTCTCCTATACTGGACTTTGTGGTGGCAGTGCTATA
CTCCATGGTTCCTCCAGCAGTGAATCCCCTCATCTATAGCATGAGGAACCAAGAGCTCC
AGGTGGCAGTCTGGAAACTGATAACTGGGTGGTTTTCTGAAGCAATAAAATTCTTCTG
CGTAGCAGTAAAAATGTAA
>Egret_OR5V1 (Egret29)
ATGGCAAGCAAGAACCATACTCATGTGACGGAGTTCATGCTCCTGGGCTTTTCCCATGG
CCAGCCCTTCTTCTTTGTTCTTTTCCTGGTCATTTACCTGGCCACGCTGCTGGGGAACT
CTGTAATGCTCGCCCTCGTGTCCCTGGATCCCCATCTCCAGAGCCCCATGTATTTCTTTC
TCAGTCACCTGTCCTGCTTGGACATTTGCTATTCATCAGTGACGGTGCCCAAGATCCTG
GCAAATGCCCTGCGCCCGCAGGCGACCATCTCCTACTGTGGGTGCCTGGCACAGATGT
TCTTCCTGATGGGGTGTGCAGGGGCCGAGTGTGCACTCCTGGCTGTCATGGCCTATGA
CCGCTACGCAGCCATATGCCAGCCCCTGCGCTACGCCCATGCCATGAGCCGGGGCATCT
GTGCTGGGGCAGCTGCCGGCTGCTGGCTCTGGGGGATGCTGGACTCGGCCGTGCACA
CCCTCCTGGCCACCAGGCTCTCCTTCTGCGGGGCTGCCCAGCTCCAGCACATCTTCTGT
GACGTCCCCCCACTGCTCAGGGCTGCGTGCAGCAACACCCACCCCAGCGAAGTGGCA
CTCCATGCTGCCAGCGTCTTTGTGGGCCTCAGCCCCTTCCTGCTTGTCATCGTCTCCTA
CCTCCGCATCCTGGCCACTGTCCTCAGGATGCCCGTGGCCACCAGCCGGCACAAGGCT
TTCTCCACATGCTCTGCCCATCTGCTCGTGGTCATCCTGTACTTTGCGACAGCCAATCTG
AACTACAACCGGCCCAGCTCCGGCTACTCCCCGGCATCTGACATATTGGTCTCCACACT
GTACTGCATCGTCACCCCCATGCTGAACCCCCTCATCTACAGCCTCCGCAACCAGGAG
GTGCGGGGGGCCCTGAGGAAGGCTGTGTGGGGACGGGGCACACCAGGCTCCCCAGG
CAGCAATGCATGA
>Egret_OR52B2 (Egret30)
ATGCCACCATTCAACCTTACCAGCTTGACACCAGCAACGTTCATCCTGGCTGGCATCCC
AGGCATGGAGAAGTCTCACATCTGGATCTCGATCCCATTCTGCTCCATGTATCTGGCTG
CCCTGCTGGGAAATGGTGTCATGTTGTTTGTCATACGGACAGAACGTAGCCTCCACCA
GCCCATGTACCTCTTCCTGTCCATGCTGGCCGTTGCCGACCTGGTGCTGTCGACCACGA
CTCTGCCCAAGATGCTGGCCCTGTTCTGGTTCAGTGCTGGGGAGATTTCCTTCGGTGCC
TGCCTGACCCAGATGTTCTTCCTGCACTTCAGCTTCTCGGCAGAGTCGGTGATCCTGCT
GGCCATGGCCTTTGATCGGTTTGTTGCCATCTGTTACCCCCTGCGGTACATGGCGGTGC
TGACCCAGTCAACCGTGGTGAAAGCCGGGTTGGTGGCTTTGCTGAGAAGTTTCTGCAT
CATTTTCCCGTGTATATTTCTTTTGAAAAGGCTGCCGTTCTGTGGGCACAATGTCATCCC
GCACACCTACTGCGAGCACATGAGCATCGCCCGGCTGGCCTGCGCCGACATCTCTGTC
AATATCTTGTATGGCCTCGCAGTGCCTTTTGCAGCAATAGTGGTAGATGTTGTACTCATT
GCTGTCTCCTATGTCTTAATTCTCCTGGCATTATTCAGACTCCCTTCCAGGACTGCCCGC
CACAAGGCTTTCCACACCTGCGTCTCTCATGTCTGTGTTATATTGCTTTTCTATATTCCCG
CTTTCTTCACTGTTTTAACGCACCGCTATGGGCAGCAAATCCCCCACCACATCCATATTC
TGCTGGCCAACCTGTACGTGCTCTTCCCCCCGCTGCTCAACCCCATCGTGTATGGTGTG
CGCACACAACAGATAAAAGAGAAGGTTGTGAATGTGTTCCTCTGCCCCAAGAGCCGTT
TGCTGCAGGAATAA

>Egret_OR14I1 (Egret31)
ATGTCCAACAGCAGCTCCACCACAAAGTTCCTCCTCCTGGCATTCACAGACACACGGG
AGCTGCAGCTCTTGCACTTCTGGCTCTTCCTGGGCATCTACCTGGCTGCCCTCATGGGC
AACGGCCTCATCATCACCGCCATAGCCTTTGACCACCGCCTCCACACCCCCATGTACTT
CTTCCTCCTCAACCTCTCTGTCATCGACCTGGGATCCATCTCCACCACTCTCCCCAAAT
CAGTGGCCAATTCCCTCTGGGACATTACGGACATCTGTTACTTGGGATGTGCTACTCAA
GTCTTTTTTTATTTTTCTGTGCTACTGCAGAGTTTTCTCTTCTCACTGTCATGGCCTATG
ACCGCTATGTTGCCATCTGCAAACCCCTGCACTATGGGACCCTCCTGGGCAGCAGAGC
TTGTGTCCACATGGCAGCAGCTGCCTGGGGCAGTGTTTTTCCCTATGCTGTGCTGCACA
CAGTCAATACATTTTCAGTCCCCCTCTGCAAGGGCAATGCTGTGGACCAGTTCTTCTGT
GAAATCCCCCAGATCCTCAAGCTCTCCTGCTCAGATGCCTACCTCAGGGAGGTTGGGC
TTATTGTGGTCAGTGCCTGTTTAGCACTTGGGTGTTTTGTATTCATTGTGGTGTCCTATG
TGCAGATCTTCAGGGCCGTGCTGAGGATCCCCTCTCAGCAGGGACGGCACAAAGCCTT
TTCCACGTGCCTGCCTCACCTGGCCATGGTTTCCCTGTTTATCAGCACTGGCACGTTTG
CCTACCTGAAGCCCCCCTCAATCTCTTCTCCTTTGCTGGACTTTGTGGTTGCAGTCCTG
TACGCGGCGGTTCCTCCAACAGTGAATCCCCTCATCTACAGCATGAGGAACCAGGAGC
TCCAGGATGCTGTGTGGAAATTGATAACTGAATGTTTTTTAAAGCAACAAAATTCTTC
TGGGCAGCAGCTATAATGTAA
>Egret_OR51G2 (Egret32)
ATGGAGCATGACTCACATACCACGTGGGAATTCAATGGCTCCTTCTACCAGCCTTCAGA
TTTCCTCATGATGGGTATCCCAGGCCTGGAGGCCCTTCACCGCTGGATCTCCATCCCTTT
CTGTGCACTGTACCTTATTGGTCTCTTGGGAAACTGCATGATCCTATTCATCATAAAGAA
GACCCAAAGTCTTCATGAACCAATGTACTACTTCCTCTCCATGCTGGCGGTCACTGACC
TGGGCTTGGTTCTAAGTACACTGCCTACTACTCTGGGCATTTTCTGGTTTAATATGCGAA
GGATTGGGTTTGACGCATGCCTCACTCAGATGTATTTCATCCACATACTGTCCTTCATTG
AATCCTCTGTGCTCCTGGCAATGGCATTTGACCGCTTCATTGCCATCTCCCATCCACTGA
GACACCCATCCATACTGACCAAGACGACTGTCATAAAAATAGGTCTGGCAATTATATTG
AGAGGTATGGTCTCCCTCCTTCCCATACCCTTCTTGCTCAAGAGACTAACCTATTGCAG
GAAGACTGAGCTTTCTCATTCTTTTTGCTTCCATCCTGATATCATGAACCTAGCATGTGC
AGATATCAAAGTCAATGTCTTCTACGGTATGATTATTCTCTTATCAACGGTGGGGATGGA
CTTCATCTTCATTGTGCTGTCCTACATCCTGATCATTAAAACTGTTATCAGCCTTGCAAC
CAAGGAGGAGTGTCTCAAGGCTTTGAACACATGCGTCTCCCACATCTGTGCTGTTCTA
GTGTTCTTCATCCCAATGATCGGACTGTCCATGATCCATCGCTTTGGAAAGAACGTTCC
TCCTCTGGTTAACACTTTGGTGGCCTACACCTACCTTATAATTCCCCCTGCTCTCAACCC
CATTATCTACAGCATAAAATCCAGCCACATCCGTGAGGCTTTGCTCAGGGCACTGCGGA
GGAAGTGTGAATCTGACTGGTAG
>Egret_OR5V1 (Egret33)
ATGAAAGATAAGATGCAGAGGGTCAACCTATCTACGGTATCTGAATTTGTTCTTGTAGG
CCTTTCCGATGCTCCAGAAGACCGTTTTCTTCTCTTTGTGCTGTTTTTGATCATTTATTTG
GCTACCTTGGCAGGCAACATCACAATCCTTGTTGCCGTTATCACAGACACTCGTCTGCA
CAACCCCATGTACTTTTTCCTTGGCAACTTATCCTTACTGGATATCTTGTGTCCCACTATC
ACTGTGCCGAAGATGCTGGAGGCCTTGTTGCTTGAGAACAAGGTGACTTCGTTCATTG
GCTGCATGTTCCAGCTGTTCTTCCTCATTGATGTTGTAGGCACAGAGATTTTTCTCTTGG
CTGTGATGGCGTACGACCGTTACATTGCAATATGTCATCCACTGCAGTACATGAATATTG

TGAGTATGAAACTGTGTGCTCACCTGGCCATTGGCACCTGGGTAGTAGGGTTTTTAAT
TCTCTGTTGCACACATCTTTGATTTTTACACTCCTGTTTTGTGATTCTAACAAAGTTGAC
CAATATTACTGTGATATTCCTCCTATGCTGGCCCTCTCCTGCTCACCTACTTACAGCAGG
GAACTGGTAATTCTCACAGTTGCTGGGGTCCTTGGAAGCAGTGCCTTTGTAGTCACTCT
GATCTCGTATATCTGCATCCTCTTGGCTGTCCTGTGCATGAACTCTTCAGAGAGCAGGC
ACAAAGCTTTCTCTACTTGTGGTTCTCACTTGGCAGTAGTATGCCTCTTCTATGGCACC
ACAATTTGCACATATGTACGGCCTTCCTTCACCTACTCGCCTAATCGAGATAGGATAGTT
TCTATGCTCTATGGAATCCTCACTCCCCTGCTAAACCCCATAATCTACAGTCTGAGGAAC
AAAGAAATTAAATGTGCCCTCAGAAGAGTGACCAGCCAGGTAAGAACTGCTTTAACA
AGACAAGAACATGTCTCTCGCTCTCTGGTGTCCTCTGGAGCCCTAGCAGTTGGATCGG
CACTGCAGTTTGACTGA
>Egret_OR14J1 (Egret34)
ATGTCCAATGGGACCTACATCAACCAGTTCCTCCTCATGGCATTTGCAGACACACGGGA
GCTGCAGCTCTTGCACTTCTGGCTCTTCCTGGGCATCTACCTGGCTGCCCTCCTGGGCA
ACGGCCTCATCATCACCGCCATAGCACGTGACCACCGCCTCCACACCCCCATGTACTTC
TTCCTCGTGAACCTCTCCCTCCTTGACCTGGGATCCATCTCTACCACTGTCCCCAAAGC
CATGGCCAATTCCCTGTGGGACACCAGGGCCATCTCTTTCTTGGGGTGTGCTCTTCAAG
TCTTTGTATCTGTCTTTTTAATTTCAGCAGAGTATTTTCTTCTCACTGTCATGTCCTATGA
CCGCTACATTGCCATCTGCAAACCCTTGCACTATGGCACCCTCCTGGGCAGCAGAGGTT
GTCTCTACATTTCGGCAATCGCCTGGGGCAGTGGATTTCTCAATGCTGTCCTGCACACA
GCCAATACATTTTCAATCCCCCTCTGCAAGGGGAATGCTATAGACCAGTTCTTCTGTGA
AATACCCAAGATTCTCAGGCTCTCCTCCACAAATGCCTACCTCAGGGAGGTTGGCCTTA
TTGCGGTTACTGCCTGTTTACTCTTTGGTTGTTTTGTTTTCATTGTGGTGTCCTACGTCC
AAATCTTCAAGGCAGTGATGAGGATCCCCTCTCAGCAGGGGCGGCACAAAGCCTTTTC
CACGTGCCTCCCTCACCTGGCCGTTGTCTCCCTGTTTCTCAGCACTGTTATATTTGCCCA
CCTGAAGCCCCCCTCCATCTCCTCCTCAGTCCTGGATCTGGTGGTGTCATTTCTTTACTC
CGTAGTGCCTCCAGTACTGAACCCGCTCATGTACAGCTTGAGGAACAAGGAGATCAAG
GAAGCCTTGCAGAAAATATTTTAA
>Egret_OR14A16 (Egret35)
ATGTGCAACAGCAGCTCCATCACGCAGTTCCTTCTACTGGCATTTGGAGACACAGGGG
AGATGCAGCCCTTGCACTTCTGGTTCTTCCTGGGCATCTACCTGGCTGCCCTCCTGGAC
AATGGCCTTATCATCCCCGCCATAGCCTGCAACCACTGCCTCCACACCCCCATGTACTT
CTTCCTCCTCAAACTCTCCGTTGTCGACCTGGGATCCATCTCCATCACTGTCCCAACAT
CCATGGCCAATTCCCTGTGGGACACCAGGACCATTTCCTATGCAGGATGTGCTGCCCAA
ATTTTCCTGCTTTTGTTTTTGATGTCAGCAGAATTCTATCTTCTCACTGTCATGGCCTATG
ACCGCTACATTGCCATCTGCAAACCTCTGCACTACAGGACCCTGCTGGGCAGCAGAGC
TTGTGTCCACATGGCAGCAGCTGCCTGGGGCAGTGGCTTTTTCATTTCCATGCTGCACA
CAGCCAATACATTTTCACTCTCCCTCTGTCAAGGCAATGTCGTGGACAAGATCTTCTGT
CAAATTCCAAAGATCCTCAAGCTCTCCTGCTCAGATGTTTCTCTCAGGGAAGTTGGCCT
TCTTGTGGTTAGCATCTGTTTAGGCTTTGGGTGCTTCCTTTTCATTGTGCTGTCCTATGT
GCAGATCTTCAGGGCTGTGCTGAGGATGCCCTCACAGCTGGGACGGTACAGTCTTTTC
TACATGCCTCCCTCACTTGGCCGTGGTCTACCTGTTTATCAGCACTGTCACGTTTGCCTA
CCTGAAGGCCCCCTCCATTTCCTCCTCATCGCTGGGTCTGGTGGTGGCAGTTCTGTAAG
CTGTGGTGCCTCCAGCAGTGAACCTCCTCATCTACAGCATGAGGAACAAGGAAATCAA

GGATGA
>Egret_OR14J1 (Egret36)
ATGTCCAATGGCAGCTCTATCACCCAGTTCCTCCTCCTGGCATTCGCAGACACACGGGA
GATGCAGCTCTTGCACTTCTGGCTCTTCCTGGGCATCTACCTGGCTGCCCTCCTGGGCA
ATGGCCTCATCATCACCGCTGTAGCCTGTGACCACCACCTCCACACCCCCATGTACTTC
TTCCTCCTCAACCTCTCCCTCCTAGACCTGGGATCCATCTCAACCACTGTTCCCAAAGC
CATGGCCAATTCCCTGTGGGAAACCAGGGCCATTTCCTACGGGGGATGTGCTGCACAG
TTGTTTCTGTTTGTCTCTTTCATTTCAGCAGAGTATTTTCTTCTCACTGTCATGGCCTATG
ACCGCTACGTTGCCATCTGCAAACCCCTGCACTATGGGACCCTCCTGGGCAGTAGAGC
TTGCGTCAACATGGCAGCAGCTGCCTGGGGAAGTGGTTTCTTCTATGCTGTACTGCATA
CAACCAATACATTTTCATTACCCCTCTGCCATGGCAATGCCCTGGACCAGTTCTTCTGTG
AACTTCCCCAGATCCTCAAGCTCTCCTGCTCAGATACCTACCTCAGGGAATTTCGACTT
CTTGTATTCTGTACTTCTGTCTTTTTGGGATGTTTTGTTTTCATTGTGGTATCTTATGTGC
AGATCTTCAGGGCTGTGCTGAGGATCCCCTCTCAGCAGGGACGGCACAAAGCCTTTTC
CACATGCGTGCCTCACCTGGTTGTGGTCTCACTGTTTTTGAGCACCGCCACATTTGCCT
ACCTGAAGCCCCCCTCCACCTCCTCCCCATCCCTGAATCTAGTGGTTGCTGGTCTGTAT
TCTGTGGTGCCTCCAACAGTGAACCCCCTCATCTACAGCATGAGGAACCAGGAGCTCA
AGGATGCAGTGTGGAAACTGATAACTGAATGA
>Egret_OR4D5 (Egret37)
ATGGCACTGGGAAACTTCTCCCAGGTGACTGAATTCATCCTCCTGGGGCTTTCTGATAC
AAGGGAGCTGCAAGTCCTCTTCTTCACCTTCTTCTTCCTGGCCTATGCCATGGTTCTTCT
GGGGAACCTTCTCATCATTGTGACAGTCAGGACTGACCCCAAGCTCTCCTCACCCATGT
ACTTTCTCCTCTGCAATCTGTCCTTCATAGATATCTGCTGGACCTCTGTCACCTCTCCCA
GGATGCTGGTGGACCTGCTCTCCCAGAGGAAGGCCATCACGTTTGAAGGCTGTATAGC
CCAGCTGTTTTTTCTGCACTTTGTTGGGGCATCAGAGATGTTCCTCCTGACTGTGATGG
CATACGACCGCTACACTGCCATCTGCAAGCCCCTGCACTACACTGCCATCATGAGCCGG
CAGGCGTGCTGGGTGCTGGTGTCTGCCTGCTGGGCAGGGGGCTTCCTCCACTCCATTG
TCCAGACGCTGCTCACAATCCAGCTCCCCTTCTGTGGCCCTAACACAATCGACAGCTA
CTTCTGTGACGTGCCTCCTGTCATCCGGCTTGCCTGCACAGACATCTATGTCACCGAGT
GGCTCATGGCTTCCAACAGTGGCTTAATATCCCTGGTTTGCTTCCTGGTGCTGGTCACA
TCTTACATGTTCATCCTGGTCACAGTCAGGGTCCGCTTCACTGAGAGGCACTGGAAGG
CGCTCTCTACCTGTTCCTCACATATGATGGTCGTCACCCTCTTCTTTGTACCCTGCATCT
TCATCTACCTCCGGCCCTTTTCTACCTTCCCCTCTGACAAGCACATCTGTGTGATCTACA
CTGTCTTCTCCCCGGTGATGAACCCCCTCATCTATACCCTGAGGAATAATGAGGTGAAG
GCATCCATGTGGAAATTGTGGAAGCGCCGCAGAGTCTTCTGA
>Egret_OR5F1 (Egret38)
ATGATGGAAGTGGAAAACCTCACTGCTCTGCCCGGCTTCATCCTCTTGGGCTTCTCTGA
TGCCCCAGAGCTCCAAGCCACCATCTTCCCAATTTTCTTATCCCTGTACATTATGATGGT
GCTGGGGAACCTGGTGATGATCCTGCTGATCAATGCCGACCCCGAGCTCCACACCCCC
ATGTATTTCTTCCTGACCCACTTATCGTTCATAGATTTCTGCCTTTCCTCCACGATCGTCC
CGAAGGCGCTGGAGAACCTCCTGCGGGGGAGAGGCCACATCTCTTTTTTGGGTTGCTT
TGCCCAGTTGTATTTTTCCCTTGCTCTGATTGTCTGCGAGTGCTTCCTCCTGGGGGTGAT
GGCGTACGACCGATACGTGGCTGTGTGCAAGCCGCTGCTTTATGCCACCATCATGTCCA
GGGCGCGTTGCTACGGCATGATGGGGCTGGTGTACACCACGGGCTTCCTCACCTCCCT

GGGCCACACCGTCCTGGTGGGGAGGTTGTCCTTCTGCCAGGCCAGGAGCATCAACCA
CTTCTTCTGCGAGCTGCCCGCCCTCCTGCAACTCTCTTGCTCCAACACCCGCGCGAAC
AAGCTCCTGCAGGTTTCCAATGCTGAGCTGAACACTGCGGGCTCTGTCCTGATGATCC
TGGTGTCCTACACCTACATCCTCCATACCATCCTGCGCATGCCCTCGGCCAGAAGGAGG
CTCAAAGCTTTCTCTACCTGCGCTTCCCACCTGGCTGTCATCACCATCTTCTACGTGCC
GGGGATGCTCGCCTACACCCAGCCTCGCAAGGCCTGCTCCCGGGATCAGGTAAAACTG
GTTTCGGCATGTTACACCGTCCTGACCCCCACCCTCAACCCTTTCATCTACAGCCTGAG
GAACAAGGAGGTGAAGGGGGCCCTGAGGAGGCTGTGGGTGCGAAAGCTGGTGCCTC
GTCTAGCCAGGCTTTGA
>Egret_OR10A7 (Egret39)
ATGCCCCAAAGGAAAGGCTTGGAGAATCACACCGTTGGATCTGGATTCATTCTTCTGG
GATTTGCTGAGCTCTCCAGCCTGCAGGGAATGTGCTTCACAGTATTCCTGGTCATCTAC
CTCGTGGTCCTCACAGGGAACAGCATGATCGCACTCATCACCGTGGTGGACTCAAGCC
TCCACAGCCCCATGTATTTCTTCCTGAGGAACTTGTCTGTCCTGGAGATCTGCTACACA
TCAGTCACTCTGCCAAAAATGCTGATGGGTTTCCTGCTGGCAGATGGCAGGATCTCCTT
CCTTGGCTGTGCTGCCCAGCTGTATTTCCTGGTTTCCTTGGGCAGCATTGAATGCCTCC
TCCTGACTGCCATGGCCTACGACCGCTGCATAGCCATATGTGACCCCCTGCACTATACC
CTCATCATGAGCAGGGGTCTCTGCATCAGGCTGGTGGTGGGGTTGTGGCTGGTTGTCAT
ACCAGTGCAAGTAGGACAGACCTACCAGGTTTTCACTTTGCCCTTCTGTGCATCCCATG
ACCTTCATCACTTTTTCTGTGATGTCCCCCCTCTGCTGGAACTGGCTTGTGCAGACACT
TTCTGGAACCAACTGGTGCTGCACACTATCATCCTGGTGTTTGCAATCCTTCCCTTCTC
CTTAATAGTTATTTCTTACATTAAAATTATCAGGGCAATTCTGAAAATACCTTCAGTTCTG
GGCAGACACAAAGCCTTTGCCACCTGCTCTTCACACCTCGGGACGGTGACACTCTTCT
ATGGCTCGGCCACAGTCATCTACTTAAAGCAACGGTCAAGGGATTCTGGAGACACTGA
CAAATACCTGGCCCTGTTTTACACAATTGTGACCCCCATGGTCAACCCTGTTATCTATAG
CCTGAGGAATACAGAAGTGAGAATTGCCTTGAAGAGGCTCCTGTGGACAAAGTGA
>Egret_OR14J1 (Egret40)
ATGTCCAACAGAACCTTCATCACCCAGTTCCTCCTCCTGGCATTCGCAGACACACGGG
AGCTGCAGCTCTTGCACTTCTGGCTCTTCCTGGGCATCTACCTGGCTGCCCTCCTGGGC
AATGGACTCATCATCACCGCCATAGCCTGTGACCACCACCTCCACACCCCCATGTACTT
TTTCCTCCTCAACCTCTCCCTCCTTGACCTGGGATCCATCTCCACCACTGTCCCCAAAT
CTATGACTAATTCCCTGTGGGGCACCAGGGACGTTTCCTACACAGGATGTGCTGCTCAA
GCCTTTCTGTTTGTATTTTTTATTTCAGCAGAGTATTCTCTTCTCACTGTCATGGCCTATG
ACCGCTACGTTGCAATCTGTAACCCCCTGCACTACAGGACCCTCCTAGGCAGCAGAGC
TTGTGTCCACACGGCAGCCGCTGCCTGGGGCAGTGGGTTCCTCTATGCTTTCATGCACA
CAGCCAATACATTTTTACTCCCCCTCTGCCAAGGCAATGCCCTTGACCAGTTCTTCTGT
GAAATCCCCCAGATCCTCAAGCTCTCCTGCTCAGAGACCTATCTCAGGGAGGTTGGGC
TTATTGTGTTCAGTGCCTTTGTGGTCTTTGGGTGTTTTGTTTTCATTGTGCTGTCCTATGT
GCAGATCTTCAGGGCCGTGCTGAGGATCCCCTTTCAGCAGGGACGGCACAAAGCCTTT
TCCACATGCCTCCCTCACCTGGCCATGGTCTCCCTGTTTTTGATCACTGTCATGTTTGCC
TACCTGAAGCTCCCCTCCGTGTCCTCACCATCATTGGACCTGGTGGCGGCTGTTCTGTA
TTCAGTTGTGCCTCCAGCAGTGAACCCCCTCATCTACAGCATGAGGAACCAGGAGCTC
AAAGATGCAGTCTGGAAACTGGTAACTGGATGTTTTTGTGAAGCAAGAAGCTGCCCTT
TGTATTCTGCATAG

>Egret_OR14J1 (Egret41)
ATGTCCAACAGCAGCTCCACCACGATATTCTTCCTCCTGGCATTTGGAGATAGACGGGA
GCTGCAGCTCTTGCACTTCTGGCTCTTCCTGGGAATCTACCTGGCTGCCCTCCTGGGCA
ACAGCCTCATCATCGCCACCATAGCCTTCGACCACCGCCTCCACACCCCCATGTACTTC
TTCCTCTTCAACCTCTCCCTCCTTGACCTGGGATCCATCTCCACCACTGTTCCCAAGTC
AATGTTGAATTCCCTGTTGGAGACCAGGGCCATCTCCTATGCAGGATGTGCTTCTCAGC
TTTTCATGTTCATCTTTTTGATGTCAGCAGAGTATTATCTTCTCTCTGTCATGGCCTATGA
CCGCTACGTTGCCATCTGCAAACCCCTGCACTATGGGACCCTGCTGGGCAGCAGAGCT
TGTGTCCACATGGCAGCAGCTGCCTGGGGCACTGGCTTTCTCAGTGCTGTGCTGCACA
CGGTCAATATATTTTCACTCCCCCTCTGCAAGGGCAATGCTGTGGACCAGTTCTACTGT
GAAGTTCCCCAGATCCTCAAGCTCTCCTGCTCAGACACCTACCTCAGGGAGGTTGGAC
TTCTTATGTTTAGTGTGTTTGTGGACTTTGTTTTTTTTCTTTTCACTGTGCTGTCCTATGT
GCAGATCTTCAGCGTTGTGCTGAGGATCCCCTCTCAGCAGGGACGGCACAAAGCCTTT
TCCACATGCCTCCCTCACCTGGCCGTGGTCTCCCTGTTTATCAGCACTGTAGTGTTTGC
CCACATAAAGCCCCCTTCCATCTCCTCTCCTTCCCTGGATCTGGTGGTGGCAGTGCTGT
ACTCGCTGGTGCCTCCCACAGTGAACCCCCTCATCTACAGCATGAGGAACAAGGAAAT
CAAGGATGCCCTTTGGAAAATGATCCAAAGGGTGCTCTTCCACTGA
>Egret_OR14J1 (Egret42)
ATGTCCAACAGCAGCTCCATCACCCAGTTCCTTCTCCTGGAGTTCACAGACACACGGG
AGGTGCAGGTCTTGCACTTCTGGCTCTTCCTGGGCATCTACTTGGCTGCCCTCATGGGA
AACGGCCTCATCATCACTGCCATAGCTTGCAACCAACGCCTCCACACCCCCATGTACTT
CTTCCTCCTCAACCTCTCCCTTGTTGACTTGGGCTCCATCTCTACCACTGTTCCCAAATC
CATGGCCAATTCCCTGTGGGACACCAGGACCATCTCCTACACAGGATGTGCTGCCCAG
CTCTTTCTGTTTACATTCTTGACAGTAGCAGAGTATTGTCTTCTCTCCGTCATGGCCTAT
GACCGCTATGTTGCCATCTGCAAACCCCTGCACTACGGGATCCTCCTGGGAGGCAGAG
GATGTGTCCACATGGCAGCAGCTGCCTGGGGCAGTGCTTTTCTCAATTCTTTCCTGCAT
ACTGTCAATACATTTTCCATACCCCTCTGCAAGGGCAATGCCCTGGACCAATTCTTCTG
TGAAATCCCCCAGATCCTCAAGCTCTCCTGTGCAGAAGCCTACCTCAGGGAAGTTGGG
CTTCTTGTGGTTAGTTTTTCTTTAGCTTTTGTTTGTTTTGTTTTCATTGTGCTGTCCTATG
TGCAGATCTTCAGGGCTGTGATGAGGATCCCCTCTCAGCAGGGGCGGTGCAAAGCCTT
TTCCATGTGCCTCCCTCACCTGGCCGTGGTCTCCCCGTATATCAGCACTGGCATGTTTGC
CTACCTGAAGCCCCCTTCTGTCTCCTCCCCATCCCTGGATCTGGTGGTTAGTGTGTTGTA
TTCAGTGGTGCCTCCGACAGTGAACCCCTTCATCTACAGCATGAGGAACCAGGAGCTC
AAAGATGCACTCAAGAAGCTGATTTCATGGGTAGTATTTCAGCAGCAATAA
>Egret_OR14J1 (Egret43)
ATGTCCAACAGCAGCTCCATCACCCACTTCCTCCTCCTGGCATTCGCAGACACACGAG
AGCTGCAGCTCTTGCCCTTCTGGCTCTTCATGGGCATCTCCCTGGCTGCCCTCCTGGGC
AACGGACTCATCATCACCGCTGTTGCCTTTGCCCACCGCCTCCACACCCCCATGTACTT
CTTCCTCCTCAACCTCGCCCTCCTTGACCTGGGCTCCATCTCCACCACTGTCCCAAAAG
CCATGGCCAATTCCCTCTGGGACAGCAGGGCCATTTCTTATGCAGATTGTGCTGCCCAA
GTTTTTCTTGTTTTCTTTTTGATGTCAGCAGAATTGTGTCTTCTCACTGTCATGGCCTAT
GACCGCTATGTTGCCATCTGCAGACCCCTGCTCTACAGGACCCTCTTGGGTAGCAGAG
CTTGTGTCCATATGGCAGCAGCTGCCTGGGGCAGTGGATTCCTCAATGCTGTGCTGCAC
ACTGCCAATACATTTTCAATTCCCCTCTGCCAAGACAATGTTTTGGACCAGTTCTTCTGT

GAAATCCCCCAGATCCTCAAGCTCTCCTGCTCAGATGTTTCTCTCAGGGAAGTTGGGCT
TCTTGTGTTTGGTGTCTGTACTGCATTGGGGTGTTTTGTTTTCATTGTGGTGTCCTATGT
GCAGATCTTCAGGGCCGTGCTGAGGATCCCCTCTCAGCAGGGACAGCACAAAGCCTTT
TCCACGTGCCTCCCTCACCTGGCCGTGGTCTCGCTATTTCTCAGTACTGCAGTATTTGA
CCACCTGCATCCCCCCTCCATCTTGTCCCATCCCTGGATCTTGGGGTGGCAATCCTGTA
CTCGGTGGTGCCTCCAGCAGTGAACCCCCTCATCTATGCCTTGAGGAACCAGGAAATC
AAGGATGCCTTATGGAAACTGATGCAAAGGACGCTGTTTCACTGA
>Egret_OR14A16 (Egret44)
ATGTCCAACAGCAGCTCCATCACCCAGTTTCTCCTCCTGGCATTTGCAGACACGCGGG
AGCTGCAGCTCTTGCACTTCTGGCTCTTCCTGGGCATCTATCTGGCTGCCCTTCTGGGC
AACGGCCTCATCATCGCCACCATAGCCTGTGACCACCGCCTCCACACCCCCATGTACTT
CTTCCTCTTCAACCTCTCCCTCCTCGACCTGGGATCCATCTCCTCCACTCTCCCCAAGT
CCATGGCCAATTCCCTCTGGGACACGAGGGCCATCTCCTACTTGGGATGTGCTGCACA
AGTCTTTTTCTTTCTCTTCTTGATTGTAGGGGAGTATTTTCTTCTCACTGTCATGGCCTAT
GACCGCTACGTTGCCATCTGCAAACCCCTGCACTATGGGACCCTGCTGGGCAGAAGAG
CTTGTGTCCACATGGCAGCAGCTGCCTGGGGCAGTGGGTTTGTCAATGCTCTGCTGCA
CACTGCCAACACATTTTCACTACCCCTCTGCCAAGGCAATGCTGTGGACCAGTTCTTCT
GCGAACTGCCCCAGATCCTCAAGCTCTCCTGCTCAGACTCCTACCTCAGGAAGGCTGG
GCTTCTTGTGTTGGCTTTTTCTTTATCTTCAGGGTGTTTTGTTTTCATTGTGCTGTCCTAT
GTGCAGATCTTTAGGGCTGTGCTGAGGATCCCCTCTGGGCAGGGACGGCTCAAAGCCT
TTTCCATGTGCATCCCTCACCTGGCCGTGGTCTCCCTGTTTATCAGCACTTCCATGTTTG
CCTACCTGAAGCCTCCATCCTCCATGGAGGAGTCCCCATCTCTGGACCTACTGGTGGCA
TTTTTGTATTCGGTGCTGCCTCCAGCAGTGAACCCCATCATCTACAGCATGAGGAACAA
GGAGCTCAAGGGTGCACTGAAGAAGCTGATTCAATCATTTGTCTTTCAACAGCGATAA
>Egret_OR14A16 (Egret45)
ATGCCCAATGGCAGCTCCACCACCGAGTTTCTCCTTCTGGGATGCGGGGATACATTGGA
GCTGCAGCTCTTGCACTTCTGGCTCTTCCTGGGCATCTACCTGACTGCCCTCCTGGGCA
ACGGCCTCATCATCACCACCATAGCCTGCGACTACCGCCTCCACACCCCCATGTACTTC
TTCCTCCTCAACCTCTCCCTCCTCGACCTGGGATCCATCTCCACTACACTCCCAAAAGC
CATGGCCAATTCCCTCTGGGAAACCAGGGCCATTTCCTACTCAGGATGTGTTGCACAG
GTCTTTTTCTTTCTCTTCCTGATTGGAGGAGAGTATTGTCTTCTCACTGTCATGGCCTAT
GACCGCTATGTTGCCATCTGCAGACCCCTGCACTATGGGACCATCCTGACCAGCAGAG
CTTGTGTCCACATGGCAGCAGCTGCCTGGGGCAGTGGCTTTCTCTATGCTGTGCTGCAC
ACGGCCAATACATTTTCACTCCCACTCTGCCAAGGCAATGCTGTGGACCAGTTCTTCTG
TGAAATCCCCCAGATCCTCAAGCTCTCCTGCTCAGATGCCTACCTCAGGGAGGTTGGG
CTTCTCATGGTTAGTGGCATTTTACTCTTTGGTTGTTTTGTTTTCATTGTGCTGTCCTATG
TGCAGATCTTCAGGGCCGTGCTGAGGATCCCCTCTGAGCAGGGGCGGCACAAAGCCTT
TTCCACATGCCTCCCTCACCTGACTGTGTTCTCCCTGTTTATCAGCACCGGCACATTTGC
TTACTTGAAGCCCCACTCCATCTCCTTCCCATTGCTGGATCTGGTAGTGGCCGTGCTGT
ACTCTGTGGTGCCTCCAGTGGTGAACCCTCTCATCTACAGCATCAGGAACAAGGATCT
GAAGAATGCCTTGAGGAACTTATTTCAATACATGCTTCTTCAGCATCCATAA
>Egret_OR14J1 (Egret46)
ATGTCCAACAGCAGCTCCATCACCCAGTTCCTCCTCCTGCCATTCGCAGACACATGGG
AGCTGCAGCTCTTGCACTTCTGGCTCTTCCTGGGCATCTACCTGGCTGCCCTCCTGGGA

AATGGCCTCATCATCACTGCTGTAGCCTGCGACTACCGCCTCCACACCCCCATGTACTT
CTTCCTCCTCAACCTCTCCCTCCTTGATCTGGCCTCCATCTCCACCACTCTTCCCAAATC
CATGGCCAATTCCTTCTGGGATACCAGGGACATCTCCTACTTTGGATGTGTGACCCAGG
TCTTCCTGTTTGCCTTCTCTGTTGGAGCAGAGTATTACCTTCTTACCATCATGGCCTATG
ACCGCTACATTGCCATCTGCAAACCCCTGCACTACGGGACCCTCCTGGGCAGCAGAGC
TTGCGTCCACATGGCAGCAGCTGCCTGGGGCAGCGGCTTTCTCAGTGCTGTGCTGCAC
ACTGCCAATACATTTTCACTACCACTGTGCAAGGGCAATGCTCTGGACCAGTTCTTCTG
TGAAATACCCAAGATCCTCAAGCTCTCCTGCTCAGAGGCTTATCTCAGGGAGGTTTGG
CTTCTTGTCCTTAATGCCTGTTTAGTTTTAATGTGTTTTGTTTTCATTGTGGTGTCCTATAT
GCAGATCTTCAGGGCTGTGCTGAGGATCCCATCTCAGCAGGGACAGCACAAAGCCTTT
TCCACGTGCCTCCCTCACCTGGCCGTGGTCTCCCTGTTTATCAGCACTGCAGTGTTTTC
TTACCTGAAGCCCCACTCTGTCTCCTCCCCATCCCTGGACCTGGTGGTGGCAGTTCTGT
ACTCGGTGGTGCCTCCAGCAGTGAACCCTCTCATCTATAGCATGAGGAACCAGGAGCT
CAAGGATGCCCTGAGGAAACAGATTCAATGGGTTCACCTTCGGCAACAGTAA
>Egret_OR14J1 (Egret47)
ATGTCGAACACCAGCACCATTACCGAGTTCCTACTGCTGTTCGTAGATAGATGGGAGTG
GCAGCTCTTGCACTTCTGGCTCTTCCTGGGCATCTACCTGGCTGCCCTCCTGGGCAACG
GCCTCATCATCACTGCCATAGCTTGCAACCACTGCCTCCACACCCCCATGTACTTCTTC
CTCCTCAACCTCTCCATCATTGACCTGGCCTCCATTTCCACCACTGTCCCCAAATCCATG
ACCAATTCCTTCTGGCACTCCAAGGCCATCTCCTATGCAGGATGTGCTGCTCAGCTCTT
TCTGTTTACCTTCTTTGCAGTAGCAGAGCATTTTCTTCTCACTGTCATGGCCTATGATCG
CTACGTGGCCATCTGCAAACCCCTGCACTACGGGACCCTCCTGGGCAGCAGAGCTTGT
GTCCACATGGCAGCAGCTGCCTGGGGCAGTGGCTTTCTCGATTCTCTCCTCCACACTG
CCAATACATTTTCACTAACTCTCTGCAAGGGCAATGCTGTGGAGCAGTTCTTCTGTGAA
ATCCCCCAGATCCTCAAGCTCTCTTGCTCAGATGCCTACCTCAGGGAGGTGTGGCTTAT
TGGTTTTAGTACCTTTGTTGTCTTTGGCTGTTTTGTTTTCATTGTACTGTCCTATGTGCAG
ATTTTCAGGGCAGTGCTGAGGATCCCCTCTCAGCAGGGACGGCACAAAGCCTTTTCCA
CCTGCCTCCCTCATCTGGCCGTGGTCTCCCTGTTTCTCAGCACTGCAGTGTTTGCCTAC
CTGAAGCCCCCCTCCATCTCTTCCCCAGTTCTGGATCTGGTGGTGGCTGTTCTGTACTC
AGTTGTGCCTCCAGTAGTAAACCCCCTCATCTACAGTGTGAGGAACCAGGAGCTCAAG
GATGCCCTGAGGAAACTGATTCAATGGGTTCACCTTCAGCACCAGTAA
>Egret_OR52K1 (Egret48)
ATGTCAACCGTCAACCGATCCAACACCAACTCTTCGCCTTTTCTCCTGATGGGCATCCC
TGGCCTGGAAGCTCTCCACGTCTGGATTTCCATCCCATTCTGCTTCACATACATCATGAC
CTTGCTGGGAAATAGCATGGTCCTTCTCACAGTGAGGCTGAACAAAAGCCTCCATGAA
CCTATGTACTATTTCATTTCCATGTTGGCGGTCATTGACCTCATCTTCTCAACTGCTGTAG
TTCCCAAAATGCTGGGTGTATTCTGGTTGGGTTCAAGGGAGATTGGTTTTGAGGCCTGC
TTCATCCAGATGTTCTTCATCCACACATTCACTGCAGTGGAGTCAGGGGTGCTCCTGGC
AATGTCCTTTGACCGCTACGTAGCCATCTGCAAACCCTTGAGATACAACACCCTCCTAA
CGAGCTCAAGGACCATCCAGCTAGGACTGCTGTCCCTGGCCCGGGGAGCTGGTGTCAT
GACACCTTTAATGTGCCTCCTCACCAGCTTACCCTACTGCAAAACCAGAGTCATCCCTC
ATTCCTACTGTGAGCACATGGCCGTAGTGGAGCTGGCCTGCGCAGACCCGGCTGTCAG
CGACCTCTACAGCCTCATCGTGGCAACACTATTGGTGGGGACAGACTCCGTTTTCATCG
CCTTCTCCTATGGTATGATCCTCAGGTCTGTGCTGAGGCTGCCGTCCCAAGAGGCACGT

CTCAAGGCCCTCCGGACCTGTGGGTCCCATGTTTCCATCATCCTGTTGTTCTACATAGG
TGGCCTACTCTCCATGTACCTGCAGATGTTCTCTTTTGGCTTAGCACCTCACATCCAAGT
CCTGGTGGCTGATTTCTATCTGACGGTCCCTCCCATGCTCAACCCCCTCATTTACGGCAT
AAAGATGAAGCAGATCCAGGAAGGGATCCTCAAACTGTTGGGGCAGTTGTCAGGACT
CAGTATCTCACAAGCTGATAAAGACAGGTCAGATGTTGAGAGAAAGAGGCACTGCCA
GACAATAATTCATCCCATCCCAGAATAG
>Egret_OR14J1 (Egret49)
ATGTCCAATAGCAGTTCCATCTTTGAGTTCCTCCTCCTGGCATTCGCAGACACACGGGA
GCTGCAGCTCTTGCACTTCTGGCTCTTCCTGGGCATCTACCTGGCTGCCCTCCTGGGCA
ACGGCCTCATCATCACCGCCATAGCCTGTGACCACCGCCTCCACACCCCCATGTACTTC
TTCCTTCTCAACCTCTCCCTCCTCGACCTGGGCTGCATCTCTACCACTGTCCCCAAATC
AATGGCCAATTCCTTGTGGGACACCAGGGCCATCTCCTACGCAGGATGTGCTGCCCAA
GTATTTCTGTTTGTCTTTTTGATGTCAGCAGAGTATTGTCTTCTTACTGTCATGGCCTATG
ACCGCTACGTTGCCATCTGCAAACCCCTGCACTACGGGACCCTCCTGGGCAGCAGAGC
TTGTGTCCACATGGCAGCAGCTGCCTGGGGCTGTGGCTTTCTATATTCTCTCCTGCACA
CAACTACTACATTTTCCATACCTCTCTGCCATGGCAATGCAGTGGACCAGTTCTTCTGTG
AAATCCCCCAGATCCTCAAGCTCGCCTGCTCAGATGCCTACCTCAGGGAGGTTGGGGT
TATTGTTGTCAGTGTCTTTGTGATTCTTGGATGTTTTGTGTTCATTGTGCTGTCCTATGTG
CAGATCTTCAGGGCTGTGTTGAGGATCCCATCTGAGCAGGGACGGCACAAAGCCTTTT
CCATGTGCCTTCCTCACCTGGCTGTGGTCTCCCTATTTCTCAGCACTGTAGTGTTCGCCT
ACCTGAAGCCCCTCTCCATCTCTTCCCCATTCCTGGACCTGGTGGTGGCGGTGCTGTAC
TCAGAGGTGCCTCCAGCAGTGAACCCCCTCATCTATAGCATGAGGAACCAGGAGCTCC
AGGATGCTGTGTGGAAACTGATGACTGGGTGGTTTTCTGAACCAACAAAATTATTCTAC
GCAGCAGCTATAATGTAA
>Egret_OR14J1 (Egret50)
ATGGAGCTGAAGGAAGGTCACCTGGGAAAGGAATGGGTAGTGAGGAGTATCGCATGG
CGTAGGTCTAGGAGAAAGGCTTTGATTTGCCTCAGAGAAGTCTCCCTTAACTCTTCATT
GACTCTCCCTCTAAGAACAGGTCCCAGTGCCCAGAGGAAGCTACTTTTCAACAGCAGC
TCCGTCACTCAATTCCTCCTCCTGGCATTCGCAGACACACGGGAGCTGCAGCTCTTGC
ACTTCTGGCTCTTCCTGGGCATCTACCTGGCTGCCCTCCTGGGCAATGGCCTCATCATC
ACCGCCATAGCCTGTGACCACTGCCTCCACATCCCCATGTACTTCTTCCTCCTAAACCT
CTCTGTCATTGACCTGGCCTCCATTTTGACCACAGTCCCCAAATCCATGGCCAGTTCCC
TCTGGGACACCAGAGACATCTCCTATGCAGGATGTGTTGCTCAGCTCTTTCTCTTTGCC
TTCTTGGTAACAGCAGAATATTTTCTTCTCACTGTCATGGCCTATGACCACTACATTGCC
ATCTGCAAACCCCTGCACTACAGGACCCTCCTGGGCAGCAGAGCTTGTGTCCACATGG
CAGCTGCTGCCTGGGGCAGCGCATTTTTCACTGCTCTCCTTCACACGGCCAATACATTT
TCACTCCCCCTCTGCCAAGGCAATGCTGTGGACCAGTTCTTTTCTGAAATCCCCCAGAT
CCTCAAGTTCTCCTGCACAGATGCTTATCTCAGAGAGGTGTGGCTTATTGGTTTTAGTA
CCTTTATTGTCTTTAGGTGTTTCATTTTCATTGTGGTGTCCTACATGCAGACCTTCAGGG
CTGTGCTGAGGATCTCCTCTCAGCAGGGACGGCACAAAGCATTTTCCATGTGCCTCCC
TCACCTGGCCGTGGTCTCCCTGTTTATTATATTTTCCAACCTGAAACCCCCTTCCACCTC
TTACCCATCCCTGGACCTGGTGGTGTCATTTCTGTACTCAGTGGTTCCTCCAGCAGTGA
ACCCCATCATCTACAGCATGAGGAACCAGGAACTCCAGGATGGAGTGTGGAAACTGAT
CCAATGGGTTTGA

>Egret_OR14I1 (Egret51)
ATGTCCAATGCCAGCTCCATCTCCGAGTTCCTCCTCCTGGCATTTGCAGACACGTGGGA
GCTGCAGCTCTTGCACTTCTGGCTCTTCCTGGGCATCTACCTGCTTGCCCTCCTGGGAA
ACGGCCTCATAATCACCACCGTAGCCTGCGAACAATGCCTCCACACCCCCATGTACTTC
TTCCTCCTCAGTCTCTCCATCCTCGACCTGGCCTCCATCTCTACCACTGTCCCCAAAGC
CATGGCCAATTCCTTGCAGGACACCAGGGACATTTCCTACCTGGGATGTGCTGCACAG
GTCTTTCTGTTTGTCTTTTTCATGTCAGCAGAGTTTTCTCTTCTCACTGTCATGGCCTAT
GACCGCTACGTTGCCATCTGCAAACACCTGCACTACGGGACCGTCCTGGGCAGCAGAG
CTTGTGTCTGCATGGCAGCAGCTGCCTGGGGCAGTGGGTTCCTCTATGCTGTCCTTCAC
ACTGCCAATACACTTTCCCTCCCACTCTGCCAAGGCAATTCTGTGGACCAGTTCTTCTG
CGAACTTCCCCAGATCCTCAAGCTCTCCTGTTCAGATGCCTACCTCGGGGAATTTCAAC
TTCTTGTGTTTAGTGCTTTTGCCTTTTTGGGGTGTTTTATTTTCATTGTGGTGTCCTATGT
GCAGATTTTCAGGGCCATGCTGAGGATCCCCTCTCAGCTGGGACAGCACAAAGCTGTT
TCCACGTGCCTCCCTCACCTGGCTGTGGTCTCTCTGTTTATGAGCACTGGCATGTTTGC
CTACCTGAAGCCCCCCTCGATCTCTTCCCCATCCCTGAATCTAGTGGTTGCTGCTCTGTA
TGCATTGGTGCCTCCAGCAGTGAACCCCCTCATCTACAGCATGAGGAACCAGGAGCTC
AAGAAAGCAGTGTGGAAAGTGTTGACTGAATGTTTTCCAGAAGCTATAGTGTGTCTAT
CTTCTTCAACTTAA
>Egret_OR14A16 (Egret52)
ATGTCCAACAGCAGCTCCATCACCCAGTTCCTCCTCCTGCAGTTTGCAAATACATGGGA
GCTGCAGTTCTTGCACTTCTGGCTCTTCCTGGGCATCTACCTGGCTGCCCTCCTGGGCA
ATGGTCTCATCATCACTGTTGTAGCCTGTGACCACCACCTCCACACCCCCATGTACTTC
TTCCTCCTCAACCTCTCCCTCCTTGACCTGGGCTCCATTTCCACTACTCTCCCCAAATCC
ATGGCCAGTTCTGCCTGGCAGACCAGGGCTATCTCCTTCTCAGGATGCGCTGCCCAGG
TTTTTTTCTTTCTCTTTTTGATGTCAGCAGAGTTTTATTTTCTCACCATCATGGCCTATGA
CCGCTACGTTGCCATCTGCAAACCCCTGCACTATGGGACTCTGTTGGGCAGCAGAGCT
TGTGTCCACATGGCAGCAGCTGCCTGGGGCAGTGGTTTCCTCTATGCCTTAGTGCACAC
TGCCAACACATTTTCACTACCCCTCTGCCAAGGCAATGCTGTGGACCAGTTCTTTTGTG
AAATCCCTGAGATCCTCAAGCTTTCCTGCTCACGTTCCTTACTCAGGGAATTTGGGGTT
CTTATATTTAGTGCTATTTTAGCTTTTTCATGTTTTTTTTTCATCATGCTCTCCTATGTGCA
GATCTTCAGGGCTGTGCTGAGGATCCCCTCTGAGCAGGGAAAGCACAAAGCCTTTTCC
ACGTGCCTCCCTCACCTGGCTGTTGTCTCCCTGTATGTCAGTACTGCCATGTTTGCCTAC
CTGAAGCCTCCCTCCATCTCCTCTCCATCTCTGAATCTGGTGGTAGCAGTTCTGTACTC
AGTGGTGCCTCCCACAGTGAACCCCGTCATCTACAGCATGAGGAACGAGGAGCTTAAG
GATGCCATTTGGAAAGTAATTTAA
>Egret_OR14J1 (Egret53)
ATGCCCAATACCAGCTCCATCACCCAGTTCCTCCTCCTGGCATTTGCAGACAAGCGGGA
GCTGCAGCTCTTGCACTTCTGGCTCTTCCTGGGCATCTACCTGGCTGCCCTCCTGGGCA
ACAGCCTCATCATCCCTGCCATAGCCTGTGACCACCGTCTCCATACCCCCATGTACTTCT
TCCTCCTCAACCTCGCCCTCATCAACATGGGCTCCATCTCCACTACGGTCCCCAAATCC
ATGGTCAATTCCCTCTGGGACACCACCACCATCTGCTATGTAGGATGTGTATCACAAGT
CTTCCTGTTTGTATTTTTCATTTCAGCAGAATTGTATCTCCTCACTGTCATGGCCTATGAC
CGCTATGTTGCCATCTGCAAACCCCTGCACTATGGGACCCTCCTGTGCAGTAGAGCTTG
TGTCCACATGGCAGCAGCTGCCTGGGGCAGTGGCTTCCTCTATGCTGTCCTTCACACTG

CCAATACATTTTCTCTACCACTCTGCAAGGGCAGTACCATAAACCAGTTCTTCTGTGAT
GTGCCCCAGATCCTCATGCTTTCCTGCTCAGATGCTTATCTCGGGGAATTTGGTCTTATT
GTGTTTAGTGCCTTTGTGGTCTTTGGGTGTTTTGTTTTCATTGTGGTGTCCTACATGCAG
ATCTTCAGGGCCGTGCTGGGGATCCCCTCTGAGCGTGGACGGCAGAAAGCCTTTTCCA
CGTGCCTCCCTCACCTGGCCATGGTGTCCCTGTTTCTCAGTACTGCAGTGTTTGACCAC
CTCAAGCCCCCTTCCATCTCCTCCCCATCCCTGGATCTGGTAGTGGCAGTGCTGTACTC
AGTGGTGCCTCCAGCTGTGAACCCACTCATCTACGGCATGAGGAACACGGTAATCAAG
GAGGCCTTATGGGAACTGATCCAATGGGTGCTGTTTCACTGA
>Egret_OR14A16 (Egret54)
ATGTCCAACAGCAGCTCCATCACCCAGTTCTTCCTCATGGCCTTTGCAGACACACGGG
AGCTGCAGCTCTTGCACTTCTGGCTCTTCCTGGGCATCTACCTGGCTGCCCCCCTGGGC
AACGGCCCCATCATCACTGCCATAGCCTGCAACCACCACCTCCATACCCCCATGTACTT
CTTCCTCCTCAACCTCTCCCTCCTTGACCTGGGCTGCATCTCCACCACTCTCCCCAAAT
CCATGGCAAGTTCCCTCTGGGGCACCAGGGCTATCTCCTACGCAGGATGTGCTGCCCA
AGTCTTTCTTTTTCTCTTTTTGTTGTCAGCAGAGTATTACCTTCTCACTGCCATGGCCTAT
GACCGCTACGTTGCCATCTGCAAGCCCCTGCACTACGGGACCCTCCTGGGCAGCAGAG
CTTGTGCCCACATGGCAGCAGCTGCCTGGGGCGGTGGGTTCCTCTATGCTGTGCTGCA
CACAGCCAATACATTTTCACTCCCCCTCTGTCAAGGCAATGCTGTGAACCAGTTCTTCT
GTGAAATCCCCCAGATCCTCAAGCTCTCCTGCTCAAACTCCTACCTAATGGAGGTTTGG
CTTATTGTGTTTAGTGTGCTTGTAGACTTAGGTTTTTTTGTTTACATTGCACTTTCTTACG
TGCAGATCTTCAGGGCTGTGCTGAGGATCCCCTCTCAGCAGGGACGGCACAAAGCCTT
TTCCACGTGCCTCCCTCACCTGGCTGTGGTCTCCCTTTTTATCATGACTGGCATGTTTGC
CTACCTGAAGCCCCCCTCCATCTCCTATACATCCCTGGATTTGGTGGTGTCATTTCTGTA
CTCAGTGGTTCCTCCAACAGTGAACCCTCTCATCTACAGCTTGAGGAACAAGGAGCTC
AGGGATGCCCTTTGGAAACTGATTCAATGGCTGCTGTTTCACTGGCAATAA
>Egret_OR14J1 (Egret55)
ATGTCCAATGTGAGTTCCATCGTTGAGTTTCTTTTACTGGCATTCCCAGACAAGCGGGA
GCTACAGCTCTTGCACTTCTGGCTCTTCCTGGGCATCTACCTGGCTGCCCTCCTGGGCA
ACGGCCTCATCACCACTGCTGTAGTCTGTGACCACCGCCTCCACACCCCCATGTATTCC
TTCCTCCTCAACCTCTCCCTTGTTGACCTGGCCTCCATCTCCACTACTGTCCCCAAATCC
ATGGCCAATTCCCTCTGGGATACTACGGTCATCTCCTACCAGGGGTGCGTGACCCAACT
CTTTTTCTTTTTCTTCTTTATGTCAGCAGAGTATTTTCTTCTCACTGTCATGGCCTATGAC
CGCTACGTTGCCATCTGCCGACCCCTGCACTATGGGATCCTCCTGGGCAGCAGAGCTTG
TGTCCACATGGCAGCAGCGGCCTGGGGCTGTGGGTTCCTCTATGCTGTGCTGCACACG
GCCAATACATTTTCCATATCTCTCTGCAAGGGCAATACCATAGACCAGTTCTTTTGTGAA
GTCCCCCATATCCTCAAGCTCTCCTGCTCACACTCCTACCTCAGGAAGATTTGGCTCCT
TGTGTTTGGTGCCTGTTTAGCACTTAGCTGTTTTGTTTTCATTGTGCTGTCCTATGTGCA
GATCTTCCGGGCTGTGCTGATGATTCCCGCTCAGCAGGGATGGCACAAAGCCTTTTCC
ACGTGCCTCCCTCCCCTGGTTGTGGTCTCCCTCTTTGTCAGCACTGGCACATTTTCCTA
CCTGAAGCCTCCCTCCGTCTCATCCCCATCCCTTGATTTTGTGGTGGCAGTTCTGTACA
CGGTTGTACCTCTGGCAGTGAACCCCCTCATCTACAGCATGAGGAACCAGGAGCTCAA
GGAGGCCCTAAGGAAACTGATCCAATGGACGCTGTCTCACTGA
>Egret_OR5AR1 (Egret56)
ATGTTCAGGCTCTCCTCTTCCTTGGTAGATGCGAAGCCCCGAATGGCAGAGCAGAATC

GCAGCTCAGTGGCAGAGTTTGTTCTCAAGGGCCTGAGTGACCAAGCGGAGATGAAGG
TGGCCCTCTTTGTGCTGTTCCTGCTCATCTACACCGTCACCCTTTTGGGCAACGTGGGG
ATAATCGTAGTTATCCAAGGTGACCCACGACTCCACACATCCATGTACTTCTTCCTTGGC
AGCCTCTCCATTGTTGACATCTGCTTCTCCTCTGTGATTGCCCCCAGGACCCTGGTGAA
CTTCCTGTCAGAGAGGAAGACCATTTCCTTCGTTGGCTGCATGGGCCAAGCCTTCTTCT
ACATCCTCTTAGTGACGACGGAGTGTTTCCTGCTGGCTGTCATGGCGTACGACCGATAC
GTGGCCATCTGTAACCCCCTGCTCTATTCCTCTGTTATGACTCGGAGGTTGTGCATGTGG
CTGGTGGTGGGCTCCTACTTTGGGGGTGTCCTGAACTCCATCATACAGATGACCTTCAT
CATTAGGCTGCCCTTCTGCAGCTCCAATGTCATCAACCACTTCTTCTGCGACATTCCTCC
CCTCCTGGCTCTGTCCTGTGCCAACACCTACATCAATGAGATGATCCTCTTCTCCTTGG
CCAGTGTAATTGAGCTCAGCACCATCTCCACCATCCTGGTCTCCTACATCCTCATCTCCT
TTGCCATCCTGAGGATCCGTTCAGCTGAAGGCAGGCAAAAAGCCTTCTCCACCTGCGC
GTCCCACCTGACAGCAGTGACCATGCTGTATGGGACGACAATCTTCATGTATTTACGCC
CCAGCTCTAGTTACTCCCTGAACACTGACAAAGTGGTCTCAGTCTTCTACACGGTGATC
ATCCCCATGCTGAATCCCCTCATCTACAGCCTGAGGAACCAGGAGGTGAAGGATGCTC
TGAGAAGAAGAGCAGAAAGAATCACAGTCAGGCTCTGA
>Egret_OR4S2 (Egret57)
ATGGAGAATGCAAGCAGTGTGAAGGAATTCATTCTTCTGGGCCTCTCGGAGAATCAAG
GGGTGCAGAAAGTATTTTTTGTGATGTTTCTGTTCTTCTATATGATTATCGTGGCAGGAA
ATCTGCTCATTGTTGTCACTGTAATTAGCAGTCAATGTCTGAACTCCCCCATGTATTTCT
TCCTCTGCTACCTGTCCTTTGTAGATATCTGTTACTCTTCCGTCACAGCTCCCAAAATGA
TTGCTGACTTCCTCGTTGAAAAGAAAACTATCTCCTTTGTGGGCTGCATAGCACAGCTC
TTTGGGGTGCATTTCTTTGCCTGCACAGAGATCTTCATCCTCACAGTGATGGCCTACGA
TCGCTACATTGCCATCTGCAGACCTCTCCACTACACCACCCTCATGACCAAGCGTGTAT
GTGGCCGGATGGTGATCGGCTCGTGGGTAGGAGGCTTCGTGCACTCTACAGTGCAGAC
TCTTCTAACCACTCAGCTCCCCTTCTGTGGCCCTAACAAAATTGACCACTACTTCTGTG
ATGTCCACCCCCTCCTAAACCTGGCCTGTACCAACACCTACGCTGTGGGCATCATTGTC
GTTGCCAACAGTGGAATGATAACTCTGAGCTGTTTCTTCATCCTGGTCATGTCCTACATT
GTCATCCTGGTTTCCTTGAAAAGTCAAACATATGAAGGGCGATACAAGGCCCTCTCCA
CCTGTGGGTCCCACATCACTGTGGTGATCCTATTCTTCGGGCCATGCACCTTCATCTACA
TACGTCCGTCGAGCAATATGTCAGAGGACAAGAACGTGGCGGTGTTTTACACTGTCAT
CACACCCATGCTGAACCCAATCATCTATGCGCTAAGAAATGAGGAGGTGAAGAGTGCC
ATGAGAAAACTGTGGAGTAGAAAAGTGGGAAGTGAAAATGGAAAGTTGTAG
>Egret_OR14J1 (Egret58)
ATGTCCAATATCACCCCCATCACCCAGTTTGTCCTCCTGGCATTCACAGACACGCGGGA
GCTGCAGCTCTTGCACTTCTGGCTCTTCCTGGGCATCTACCTGGCTGCCCTCTTGGGCA
ACGGCCTCATCATCACCACCATAGCCTGTGACCACTGCCTCCACACCCCCATGTACTTC
TTCCTCCTCAACCTCTCCCTCCTCGACCTGGGATCCATCTCCACCACTGTCCCCAAACA
CATGGACAATACCCTCTGGGACACCGAAGCTATCTCTTACTCTGGATGTGTTGCACAGG
TCTTCTTTATACTCTTCTTTTTTGGTGCAGAGTATTATCTTCTCACTGTCATGGCCTATGA
CCGCTACGTTGCCATCTGCAAACCCCTGCACTATGGGACAGTCCTGGGCAGCAGAGCT
TGTGTCCACATGGCAGCAGCTGCCTGGGGAAGTGGGCTTCTCAGCGCTGTCCTGCACA
CTGCCAATATATTTTGCTTACCCCTCTGCCACGGCAATGCCCTGCACCAGTTCTTCTGTG
AAATCCCCCAGATCCTCAAGCTCTCCTGCTCAGACTTCTATCTCAGGGAGCTTGGGCTT

ATTGTATTTAGTGTGGTTGTGGACTTTGTCTGTTTTATTTTCATTGTGGCATCCTACGTGC
AGATCTTCAGGGCTGTCCTGAGGATCCCCTCTGTGCAGGGACAGCACAAAGCCTTTTC
CATGTGCCTCCCTCACCTGGCCGTGGTCTCTCTGTTTATCAGCACCGTTGGGTTTGCCC
ATTTGAAGCCCCCCTCTATCTCCTCCCCATCCTTGGATCTGGTGGTGGCAGTGCTGTACT
CGGTGGTGCCTCCAGCACTGAACCCCCTCATCTACAGCATGAGGAACCAGGAGCTCAA
GGATGCCCTTCGGAAACGGATGCAATGGGTGCTATTTCACTGGCAATAA
>Egret_OR14J1 (Egret59)
ATGTCCAACAGCAGCTCCATCACCCACTTCCTCCTCCTGGCATTCGCAGACTCACGCG
AGCTGCAGCTCTTGCACTTCTGCCTCTTCCTGGGCATCTACCTGGCTGCCCTCCTGGGA
AACGGACTCATCATCACCGCCATAGTCTGCAACCACCACCTCCACAGCCCCATGTACTT
CTTCCTCTTCAACCTCTCCCTCCTTGACCTGGGATCCATCTCTAACACTGTTCCCAAATC
CATGGCCAATTCACTCTGGGACATCAGGGTCATTTCCCACACAGGATGTGCTGTCCAA
GTCTTTTTGTTTTTCTTTTTGATGTCAGCAGAATTGTATCTTCTCACTGTCATGGCCTATG
ACCGCTACATTGCCATCTGCAAACCCCTGCACTACAGGACCCTGCTGGGCAGCAGAGC
TTGTATCAACATGGCAGCAGTTGCCTGGGGCAGTGGCTTTCTCAATGCTCTCATTCACA
CGGCCAATGCATTTTCCATACCCCTTTGCCATGGAAATGATGTGAACTTGTTCTTCTGTG
AAGTCCCCCAGATCCTCAAGCTCTCCTGCTCAGATGCTTCTCTCAGGGAGGGCTGGCT
TCTTGTGTTTAGTGTCTGTATTGCATTTGGGTGTTTTATTTTCATTGTTTTCTCGTATGTAC
AGATCTTCAGGGCTGTGCTGAGGATCCCCTCTCAGCAGGGACAGCACAAAGTCTTTTC
CACGTGCCTGCCTCACCTGGCCGTGGTCTCCCTGTTTCTCAGCACTGCAGTGTTTGATC
ACCTCAAGCCCCCCTCCATCTCCTCCCCAACCCTAGATCTCATCGTGGCAGTTCTGTAC
TCGGTGGTGACTCCAACAGTGAACCCCCTCATCTATACCTTGAGGAACAAGGAAATCA
AGGATGCCTCATGGAAAATGATCCGATGGGTGCTGTTTCACTGGCAATAA
>Egret_OR14J1 (Egret60)
ATGTCTAGGAGAAAGGCTTTGATTTTCCTCAGAGACCTCTCCCCTAACTCTTTGTTGTC
TCTTCCTCTGAGAACAGGACCCCTTGCGTGGAGACAGCAAATATCCAACAGTAGCTCC
ACCACCCAGTTTCTCCTCATGGCATTCACAGGCACACGTGAGCTGCAGCTCTTGCATTT
CTGGCTCTTCCTGGGCATCTACCTGGCTGCCCTCCTGGGAAACAGCCTCATCATCACCG
CCATAGCCTGCGACCACCGCCTCCACACCCCCATGTACTTCCTCACCCTCTCCGTTGTC
GACCTGGGCTCCATCTCTACCACCATCCCCAAATCTATCGCCAATTCCCTCTGGGAAAC
CAGAGGCATCTCCTATGTAGGATGTGCTGCCCAGCTATTTATGTTTGCCTTCTTAGTAGT
AGCAGAGTATTATCTTCTCACCGTCATGGCCTATGACCGCTACGTTGCCATCTGCAAAC
CCCTGCACTATGCGACCCTCCTGGGCAGCAGAAATTGTGTCCACATGACAGCAGCTGC
CTGGGGCACTGCTTTTCTCAATTCTCTCCTGCACTCTGCCAATACATTTTCCATACCGAT
CTGCAAGGGCAATGCTTTGAATCAGTTCTTCTGTGAAATCCCCCAGATCCTCAAGCTCT
CCTGCTCAGATGCCTACCTCAGGGAAGTTGGCCTTCTTGTTGTTAGTGTCTTTGTAAAC
TTTGGCTGTTTTGTTTTCATTGTGCTGTCCTATGTGCAGATCTTCAGGGCTGTGCTGAGG
ATCCCCTCTCAGCAGGGACGGCACAAAGCCTTTTCCACGTGCCTCCCTCACCTGGCAG
TGGTCTCCCTGTTTATCATCACTTCCATGTTTGCCTACCTGCATCCCCCCTCCCTCTCCTC
CCCATCCCTGGATCTGGTTGTTTCTGTTCTTTATTCGGTGGTTCCTCCAGCAGTGAACCC
TCTCATCTACAGTATCAGGAACCAGGAGCTTAAAGATGCACTCAAGAAGTTGATTTGA
>Egret_OR14A16 (Egret61)
ATGTCCAACGGTACCTTCATCTTCGAGTTCCTTCTATTGGCATTCGCAGACACACGGGA
GCTGCAGCTCTTGCACTTCTGGCTCTTCCTGGGCATCTACCTGGCTGCCCTCTTGGGAA

ACAGCCTCATCATCCCCGCCATAGCCTGTGACCACCGCCTCCACACCCCCATGTACTTC
TTCCTCCTCAACCTCTCCCTCCTTGACCTGGGATCCATCTCCACCACTGTTCCCAAATC
CATGACCAATTCCCTCTGGGACACCAGGGCCATCTCCTATTCTGGATGTGCTGCCCAGC
TCTTTCTCTTTGCCTTCTTGGCACTAGCAGAGTATTTTCTTCTCACTCTCATGGCCTATG
ACCGCTACATTGCCATCTGCAAACCCCTGCACTATGGGACCCTGCTGGGCAGCAGAGC
TTGTGTCCACATGGCAGCAGCTGCCTGGGGCTGTGCATTTTTCACTGTTCTCCTGCACA
CTGCTAATACATTTTCACTCCCCCTCTGCCAAGGCAATACTGTGGACCAGTTCTTCTGT
GAAATCCCCCAGATCCTCAGGCTCTCCTGCTCACATGCCTACCTCAGGGAAGTTGGGC
TTATTGTGGTCAGTATCTTTGTGGAATTTGGCTGTTTTGTTTTCATTGTGCTGTCCTATGT
GCAGATCTTCAAGGTCGTGATGAGGATCCCCACACAGCAGGGTCAGCACAAAGCCTTT
TCCACATGCCTCCCTCACCTGGCTGTGGTTTCCCTGTTTATCAGCACTGTCATGATTGCC
TACCTGAAGCCCCCTTCCATCTCCTCCACACCCCTGGATGTGGTGGTGGCAGTTATGTA
CTCAGTGGTGCCTCCAGCAGTGAACCCCCTCATCTACAGCATGAGGAACCAGGAGCTC
AAAGATGCACTTAAGAAGCTGATTCCATCTTTAGTATTTTAG
>Egret_OR14J1 (Egret62)
ATGGCCAAAATGTCCAACAGCAGCTCCACCACCAAGTTCCTCCTCCTGGCATTTGCAG
ACACACGGGAGCTGCAGCTCTTGCACTTCGGGCTCTTCCTGGGCATCTACCTGGCAGC
CGTCCTGGGCAATGGCCTCATCATCATGGCTGTATCCTGCGACCACCACCTCCACACCC
CCATGTACTTCTTCCTCCTCAACCTCTCCCTCCTTGACCTGGGGTGCATCTCTACCACTA
TCCCCAAATCCATGGCCAATTCTCTCTGGGACATTAGGGACATCTCCTACCAAGGGTGT
GTGGCCCAACTATTTTTATTTTTATTTTTTATGTCAGCAGAGTATTATCTTCTCACTGTCA
TGGCCTATGACCGCTACGTTGCCATCTGCAAACCTCTGCACTACGGGACCCTCCTGGGC
AGCAGGGCTTGTGTGCACATGGCAGCAGCTTCCTGGGGCAGTGGGTTCCTCTATGCTC
TCCTGCACACTGTAACTACATTTTCCATACCCCTCTGCAAGGGCAATGCTGTGGACCAG
TTCTTCTGTGAAATCCCCAAAATCCAAAAGCTCTCCTGCACAGATGATAACCCCATGGA
AGTTGGGGTTCTTGTGTTTAGTGCCTGTATAGCGTTAATATGTTTTGCTTTCATTGTAGT
GTCCTATGGGCTCATATTCAGGGCTGTGCTGAGGATCCCCTCTGAGCAGGGGTGGCAC
AAAGCCTTTTCCACGTGTTTCCCTCATCTGGCCGTGGTCTCCCTGTTTGTCAGCACTGG
CATGTTTGCCTACCTGAAGGCCCCCTCCATCTCCTCCCCATCCCTGGACCTGGTGGTCG
CTGTTCTGTACTCGATTCTGCCTCCAACAGTGAACCCCCTCATCTATAGCATCAGAAATC
AGGACCTTAAGGATGCCCTGCAGAAACTGAGTAGACACTTTTCAGCAGCCAGAGACC
ACGTATCTTCCTCCACAAATGACTTTCAGTGTCTGTCATGA
>Egret_OR10K2 (Egret63)
ATGGGGAACAACAGCCAAACCCTGGCCACAGACTTCATCTTCCTGGGTTTCTCCAGCC
TGGCGGAACTGCAGAAGCTGCTTCTTGTCGTGTTTTTGCTGCTGTACCTGGTCACTCTG
AGCATGGATACCACTCTAATGATTATCATATGGGTTGATCGGAGCCTTCACACACCCATG
TACTTTTTCCTTTGTGTCTTGTCATTTTCTGAGACTTGCTACACATTTGCCATTGTCCCC
AAGATGCTGGTAGATTTGACAGCAGAGACAAAAACCATTTCCTTCCTGGGCTGTGCTG
TACAAATGTACTTCTTCCTTTTCTTGGGGTGCTCTCACTCCTTCCTCCTGGCAGCCATGG
GCTATGACCGCTGTGTTGCCATCTGCTACCCCCTGCACTACAACAGCATCATGACTTGG
AGAGTCTGTGCTCAGCTGCTGGTTGCTTCTGCTCTGAGCGGTTTGCTAGTTGCCCAGGT
GGTTACCCCCTTGATATTTTGCTTGCCCTTCCAGGCATCCAGGAAACTCAACCATTTCTT
CTGTGACATCTCCCCTGTCCTCCGGGTGGTCTTGACTCACACAAACCTCAGCGAGGCC
ATTATCTTCGCACTGGGCATCTCCGTCCTTACGATCCCACTGATGCTGATCCTCATTTCAT

ACCTCTGCATTGTCTTGGCCATCTTGCAGATCCCTTCAGCCGCAGGGCGGCACAAAGC
CTTCTCCACCTGCAGTGCTCACCTGATAGTCGTGGTTGTTCATTATGGCTGTGCCTCCTT
CATCTACCTGAGACCCAGCTCCAGCTACTCATCGGATCAGGATGCATTGATCTCTGTCA
CTTATACGGTCCTCACTCCCCTGCTCAACCCTATGATTTACAGCCTAAGGAACAAGGAT
GTCAAAATGGCTCTTCAAAATGCAATCAGCAAAAACATATTCTCTCAGAAGGTTTTCCA
GTGA
>Egret_OR14J1 (Egret64)
ATGTTCAATAGCAGCTCCATCACCCACTTCCTCCTCCTGGCATTTGCAGACACACGGGA
GCTGCAGCTCGTGCACTTCGGGCTCTTCCTGGGCATCTACCTGGCTGCCCTCCTGGGCA
ACGGCCTTATCATCACCACCATAGCCTGTGATCACCGCCTCCACACCCCCATGTACTTCT
TCCTCCTCAACCTCTCCCTCCTTGACCTGGGCTCCATCTCCACCACTGTTCCCAAAGCC
ATGGCCAACTCCCTCTGGGACACCAGGGATATCTCCTATGCAGGATGTGCTGCCCAAGT
ATTTCTCTTTTTGTTTTTCATTTCAGCAGAACTGTCTCTTCTCACTGTCATGGCCTATGA
CCGCTACGTTGCCATCTGCAAACCCCTGCACTACGGGCCCCTCCTGGGCAGCAAAGCT
TGTGTCCACATGGCAGCAGCTGCCTGGGGCAGTGGGTTCCTCTATTCTCTCCTGCACAC
TGTCAATACATTTTCACTCCCTCTCTGCAAGGGCAATGCTGTAGACCAGTTCTTCTGTG
AAATCCCTCAGATCCTCAAACTCTCCTGTGCTGATATTTATCTAAGGGAGGTTTGGCTTA
TTATGTTTAGTGTGCTTGTGATCTCTGGATGTTTTATTTTCATTGTGCTGTCCTATGTGCA
GATCTTTAGGGCCGTGCTGAGGATCCCCTCTCAGCAGGGACGACACAAAGCCTTTTCT
ATGTGCCTCCCTCACCTGGCTGTGGTCTCCGTTTTTATCAGCACTGCAGTGTTTGCATAC
CTGAAGCCCCCTTCCATCTCTTCCCCATCCCTGGACCTGGTGGTGGTACTGCTTTACTC
AGTGGTGCCTCCAACAGTGAACCCCCTCATCTACAGCATGAGGAACAAGGACCTCAA
AAAAGCCCTTTTGAAACTGATGGAATGGGTGCTGTTTCACTGGAAATAA
>Egret_OR14I1 (Egret65)
ATGTCCAACAGCAGCTCCATCACTCAGTTCCTCCTGCTAGCGTTTGCAGACAAGAGGG
AGCTGCAGCTCTTGCACTTCTGGCTCTTCCTGGGCATCTACCTGGCTGCCCTCCTGGGC
AATGGACTCATCATCCCCGCCATAGCCTGTGACCACCACCTCCACACCCCCATGTACTT
CTTCCTCCTCAACCTCTCCCTACTCGACCTGGGCTCCATCTCCACCACTGTCCCCAAAT
CCATGGCCAATTCCCTCTGGGACACCAGGGACATCTCCTATGCAGGATGTGCTGCTCAA
GTCTTTCTGTTTGTCTTTTTCATTTCAGCAGAGTTTTATCTTCTCACTGTCATGGCCTATG
ACCGCTATGTGGCCATCTGCAAACCCCTGCACTACGGGACCCTCCTGGGCAGCAGAGC
TTGTGTCCACATGGCAGCAGCTGCCTGGGGCAGTGTTTTTCCCTATGCTGTGCTGCACA
CAGCCAATACATTTTCACTCCCCCTCTGCAAAGGCAATGCTGTGGACCAGTTCTTCTGT
GAAGTCCCCCAGATCCTCAGTCTTTCCTGCTCAGATGCCTACCTCAGGGAAATTTGCCT
TCTTGTGGTCAGTGCTTGTTTAGCACTTGGGTGTTTCGTTTTCATTGTGCTGTCCTATGT
GCAGATCTTCAGGGCTGTGCTGAGGATCCCCTCTCAGCAGGGACAGCACAAAGCCTTT
TCCACGTGCCTTCCTCACCTGGCCGTGGTCTCTCTATTTATAAGTACTTTAATGTTTGCC
TACCTGAAGCCTCCCTCCATCTCCTTCCCATCTCTGGATCTGGTGGTGTCAGTTCTGTAC
TCAGTGGTGCCCCCAGCATTGAACCCTCTCATCTACAGCATGAGGAACAAGGAGATCA
AGGAGGCCCTGAAGAAACTGTTTGAACACATACTATTTCAACATCATTAA
>Egret_OR14A16 (Egret66)
ATGTCCAACATCAGCTCCATCACCCAGTTCCTCCTCCTGGCATTCGCCGACACACGGGA
GCTGCAGCTCTTGCACTTCTGGCTCTTCCTGGGCATCTACCTGGCTGCCCTCCTGGGAA
ATGGCCTCGTCATCCCCGCTGTAGCCTGCAACCACCGCCTCCACACCCCCATGTACTTC

TTCCTCCTCAATCTCTCCCTCATCGACCTGGGCTCCCTCTCCAATACTGTCCCCAAATCC
ATGGCCAATTCCCTCTGGGACACCAGGGCCATTTCTTACACAGGATGTGCTGCTCAAGT
CTTTCTGTTTGTATTTTTAATGGCAACAGAATTTTATCTTCTCACCATCATGGCCTATGAC
CGCTATGTTGCCATCTGCAAACCCCTGCACTATGGGACCCTCCTGGGCAGCAGAGCTT
GTGTCCACATGGCAGCAGCTGCCTGGGGCAGTGGGTTCTTCTATGCTGTGCTGCACAC
AGCTAATACATTTTCACTCCCCCTCTGCAAGGGCAACGCTGTGGACCAGTTCTTCTGTG
AAATCCCCCAGATCCTCAAGCTCTGCTGCTCAGAGGCCTACCTCAGGGAGGTTGGGCT
CATTGTGGTCACTGTCTTCGTGGGTTTTGGCTGTTTTGTTTTCATAGTGGTGTCCTATGT
GCAGATCTTCAGGGCCGTGCTGAGGATCCCCTCTCAGCAAGGACAACACAAAGCCTTT
TCCATGTGCCTCCCTCCCCTGGCCGTGGTCTCCCTGTTTGTCATGCCTGCCATGTTTGCC
TACCTGAAGCCCCCCTCCATCTCCTCCCCATCCCTGGATCTGCTGGTGGCTGTTCTGTA
CTCAGTGGTGCCTCCAGTAGTAAACCCCTTCATCTACAGCATGAGGAACCAGGAGCTC
AAGGATACCCTTTCGAAAATCATCCAATGA
>Egret_OR14J1 (Egret67)
ATGTCCAACAGCAGCTCCAACACCCAGTTCCTCCTCCTGGCATTCACAGACACACGGG
AGCTGCAGCTCTTGCACTTTTGGCTCTTCCTGGGCACCTACCTGGCTGCCCTCCTAGGC
AATGGCCTCATCATTGCAGCAGTAGCCTATGACCACCACCTCCAAACCCCCATGTACTT
CTTCCTCCTCAGCCTCTCCCTCCTTGACCTGGCCTTCATCTCCACCACTGTCCCCAAAA
CCATGGCAAACATCCTGTGTGGCTCCAGGGCCATTTCCTACCAAGGCTGTGCTGCCCA
GGTCTTTATGCTTGTCTTCTTGTTCTCAGCAGCGTTTTCTTTCCTCACTGTCATGGCCTA
TGACCGCTACGTGGCCATCTGCAAACCCCTGCACTACGGGACCCTCCTGGGCAGCAAA
GCTTGTGCCCACATGGCAGCAGCTGCCTGGGCCAGTGGCTTTCTCCATGCTGTGGTGC
ACACGGCCAATATATTTTCAGTCCCCCTCTGCAAGAGCAATGCTGTGGGCCAGTTCTTC
TGTGAAATCCCCAAGATCCTCAAACTCGCTTGCTCAGATGCTTACCTCAGGAAACTTG
GGCTTATTGTGGTCAGTGCCTGTTTATGCTTTGGGTGTTTTGTTTTCATTGTGCTGTCCTA
TGTGCAGATCTTCAGGGCTGTGCTGAGGATCCCCTCTAAGCAGGGAAGGCACAAAGCC
TTTTCCACGTGCCTCCCTCACCTGGCTGTAGTCTCTCTATTCCTCAGCAGTGTTGTGTTT
GCCCACCTGAAGCCCCCCTCCATCTCCTCCTCAGCCCTGGATCTGGGTGGTGGCAACCT
ATACTCGGTGGTTCCTCCAGTAGTGAACCCCCTCATCTACAGCTTGAGGAACAAGGAG
ATAAAGGAGGCCCTGCAGAAACTATTTGGACACAGAGTACTACAACAACAATAA
>Egret_OR12D3 (Egret68)
ATGCTGAACCAGACGGAGGTCCGTGAGTTCATCCTTTTGGGCCTCACCGATACGCGGG
GGCTGCAGCACTTTTTCTTTGTCTCCTTTCTGTTGCTCTACTTGAGCAGTCTTCTGGGC
AATGGTGCCATTGTGACCATGGTGGTATCTGAGCCCCAGCTCCACACACCGATGTACTT
CTTCCTGGGGAACCTCTCCTGCCTGGACATTTTCTACTCCACAGTCACTGTTCCCAAGA
TGTTGACTGGCTTTCTCTTTGGGCATCAGCCCATCTCTTTTGGTGGCTGCTTGGCCCAG
CTCCACTTCTTTCACTTCCTGGGCAGTACTGAGGCCGTGCTCCTGGCTGCCATGGCCTA
CGACCGCTATGTGGCCATTTGCAACCCTTTGCGCTATGCACTTGTCATGAGTCCACGGA
CCTGTCTGCTGCTGGCTGTGGCCAGCTGGTCCACTGGCTTTGTACATGCCATGATGCAC
TCAGTCATGACCTCTCGACTGAGCTTCTGTGGCCACAACCACATTCATCACTTCTTCTG
TGACATCAAGGCACTCCTGAGTTTGGCTTGCAGCAGTACCAGCCTCAACATGACCCTC
CTCAATGTCGTCACCACATCTATTGCTCTATGCCCCTTCACTCTCACAGTCCTCTCTTAC
CTCTACATCATCTCCTTCCTCATCTATAAAGTCCAGTCCCATGAAGGAAGATGGAAGCC
CTTCTCCACCTGCGCCTCCCACCTCACCGTTGTAGCACTGCTTTACATACCAGTGCTCTT

AAATTATACACCACCCTCCTCAGGAAGCTCCTTGAAAAGGGATGTGCACGTGTCTCTCA
TGTACAGTGCTGTCACCCCAGCTCTGAACCCCTTGATCTACACTCTTAGGAACCAGGA
GGTGAGATCTGTCCTGAAAAAAATATTAGGGAGAAAACTCTTTCCTGGAGGAAAGTGA
>Egret_OR52B2 (Egret69)
ATGTATGAGCTCAATGAAAGCAGCTTTGATCCCATCACCTTCGTCCTCACGGGCATCCC
AGGCATGGAGGAGTCCCACGTCTGGATCTCTGTCCCCTTCTGCCTGATGTACATCACTG
CAGTGTTTGGCAACTCTGTCCTCCTCTTTGTCATCATTGTGGAAAGGAGCCTTCATGAG
CCCATGTACCTCTTCCTCGCTATGCTGGCTGTCGCTGACCTCATGCTTTCCACCACCAC
AGTGCCCAAAATGCTGGCGATCTTCTGGTTCAGTGCCAGGGAAATTTCCTTTGACGCC
TGCATTACACAGATGTTCTTTACCCATTTCAGCTTCATTGTGGAATCATCTGTCCTGCTG
GCGATGGCGTTCGACCGCTACGTGGCGGTCTGCGACCCGCTGCGGTACTCCTCAACCT
TAACCCCCTCGGTGATCGGGAAAATAGCTGTGACTGCTGTCGTCCGGGGGTTCTGCAT
CATGTTCCCACCCATCTTCCTCCTCAAGCGGCTGCCCTACTGCGGGCACAACGTCATGC
CCCACACCTACTGCGAGCACATGGGCATCGCCCGCCTGGCCTGCGCCGACATCAAAGC
CAACGTCTGGTACGGGCTGACAACTGCTCTTCTCTCCTCCGGCCTGGATGTCGTGCTCA
TCGCTGTCTCTTACGCTCTCATCCTCAGGGCGGTCTTTCGGCTCCCGTCCCCAGAGGCC
CGTCTCAAGACCCTGAGCACCTGCGGCTCCCACATCTGTGTGATCCTCATGTTCTACGT
GCCGGCCTTCTTCTCCTTCCTCACGCATCGTTTTGGCCACCACATTCCAAGTCACGTTC
ACATCCTCCTGGCCAACCTCTATGTCGTGGTCCCGCCGATGCTCAACCCCATCGTGTAC
GGGGGGAGGACAAGGCAGATCCGGGAGCGTGTCGTCCGCCTCTTCTGCCCCGCGGGG
GACTGCCCCTGCCCCGGCTGGGGGGGCCGGTGCTGA
>Egret_OR14A16 (Egret70)
ATGTCCAACAGCAGCTCCATCACCCAGTTCCTCCTCCTGGATTTTGCAAATACACCGCA
CCGGCAGCTCCTGCACTTCTGGCTCTTCCTGGGCATCTACCTGGCTGCCCTCCTGGGCA
ACGGCCTCATCATCATCGCTGTAGCTTGTGACCACCGCCTCCACACGCCCATGTACTTC
TTCCTCCTCAACCTCTCCATCCTCGACCTGGGCTCCATCTCTACCACTCTCCCCAAAGC
CATGGACAATTCTCTCTGGGACACCAGGACCATTTCCTACTATGGATGTGCTGCCCAGG
TCTTTTTCTTTCTCTTTTTGATGTCGGCAGAATTTTCTCTTCTCACCGTCATGGCCTATGA
CCGCTACGTTGCCATCTGCAAACCCCTCTACTATGGGACCCTGCTGGGCAGCAGAGCTT
GTGCCCACATGGCAGCAGCTGCCTGGGGCAGTGGGTTCCTCTATGCCTTAATGCACAC
TGCCAACACATTTTCACTACCCTTCTGCCAAGGCAATGCCATAGACCAGTTCTTCTGCG
AACTGCCCCAGATCCTCAAGCTCTCCTGCTCAGACTCCTACCTCAGGGAAGTCGGGCT
TCTTGCGGTTTGCATCTCTCTTGCTTTTGGGTGTTTTGTTTTCATTGTCCTGTCCTATGTG
CAGATCTTCAGGGCTGTGGTGAGGGTCCCTGCTGAGCAGGGATGGCACAAAGCCTTTT
CCACGTGCGTCCCTCACCTGGCCGTGGTCTCCCTGGTTATCAGCACTGGCATGTTTGTC
TACCTGAAGCCCCCCTCAATCTCCTCACCAGTTCTGGATCTGGCAGTGGCTGTTCTGTA
CTCAGTGGGGCCCCCAGCAGTGAACCCCCTCATCTACAGCATGAGGAACCAGGAGCTC
AAGGATTCCCTCAGGAAACTGATTCAATCACTTGTCCTTCAGCAGCAATAA
>Egret_OR2B2 (Egret71)
ATGGAAAACGCAGTCGCGGGGAATCGCTCATCCATCACAGAAGTCACCCTCCTGGGAT
TCATCCATCACCCTGGGCTGCAGGTTCCTCTCTTCCTGCTTTTCCTGATGATCTACATCA
TGACCATGGCTGCAAACATCTCCGTCATTGCGCTAGTGGCGGCTGACTGGCACTTTCAT
ACCCCCATGTACTTCTTCCTGGGCAACTTGTCCTGCCTGGAGGCCTGCTACAGCTCCAC
TATCCTGCCCAGGCTGCTGGCCAGCTTCCTGACGGGGGACAGGGCCATCTCCATCCAC

TGCTGCATCCTGCAATTCTATTGCTTTGGTTCTCTGACAGCAGCCGAGTGCTGTCTCCT
GGCTGTGATGTCGCTGGACCGCTACGTGGCCATCTGCAGACCGCTGCGCTACGCAGCC
TTGATGAGGGTCAGGTCCTGCCTTCACCTGGCGGCTGGGGCATGGATCAGTGGGTTCC
TGTCCACCGTTGCCAATGCCTACTGGATGTCAGGCATGACCCTATGTGGTCCCACTGAC
CTCGACCATTTCTTCTGCGATTTCACCCCACTGTTAAAACTGTCCTGCTCTGACACCAG
CCTGATGGCTCTGGTGACGTTGCTGCTTTCTGCTTTGTTCACTCTGCCCCCCTTCCTCCT
CACCCTCACCTCCTACATGTGCATCATAGCGGCCATCCTGAGAATCCCATCCTCCACTG
GCCGGCACAAAGCCTTCTCCACCTGCTCCTCCCACCTCATTGTGGTCACCCTCTACTAT
GGAACCATCATCTTTGTGTACGTCCTCCCAGACACTGACACGCTGAGGCCCCTGAACA
AACTCTTCTCTCTCTTTTACACCGTCATCACTCCTTTTGCTAATCCCTTGATCTACACCCT
GAGAAACCATGAAGTCAGGGAGAGCCTGAGGAGGGCTTTCACTGCTAGGGTCTGCAC
CACCTAG
>Egret_OR6B1 (Egret72)
ATGAAGAATCAAACTTCTGTGAATGAGTTCATTCTTCTGGGATTTTCCTATGGCCTGCA
AGTTCAGCCCTTACTTTACCTGGTCTTTCTTGTCACCTACATGGTAACAATCACTGAGA
ATGCAATCATCATCTTTGTGGTGAAAAGGAACAATCATCTCCAAAAGCCCATGTATTATT
TCCTGGGGAACTTGTCCTTCCTGGAGATTTGGTATGTCTCAGTAACATTGCCTAGGCTT
CTGTTTGGGTTTTGGTCGCAGAGAATGACCATGTCATTCTCCAGCTGCATGACCCAGTT
ATACTTCTTTATCGCCCTTATGTGCACTGAATGTGTCCTATTGGCTGTAATGGCCTATGAC
CGCTATTTGGCTGTCTGCCATCCCTTGCGCTACACCGCTATCATGACCCACAAGCTCTG
CTTTCAGCTATCGATTCTCTCATGGGCAGGAGGCTTTTCCATTTCCTTGGTCAAGGTGTC
TTTTATTTCACGCCTCTCATTTTGTGGTCCACAAGTTATAAACCACTTCTTTTGTGACAT
CTCTCCAGTCCTGAACCTTTCCTGCACTGACATGTCCCTTGCAGAGACAGTGGACTTT
GCATTAGCATTGGTGATACTGCTGTTACCTCTCTTGGTCATTGTTTTCTCCTACTGCTGTA
TCTTGACAACAATCTTGTGTATGCCTTCAGCCCAGGGAAGGAGAAAAGCCTTTTCCAC
TTGTACTTCCCATTTCACTGTAGTCATTATCTTTTTCTCAGCCACTCTCTTCATGTATGCC
AGACCCAGGAGGATCCATCCATTCAACATCAACAAAATAGTGTCTGTCTTTTATGCTGT
ATTCACTCCAGCACTGAACCCCCTAATCTATTGCCTGAGGAACAAGGAGGTGAAAGAG
ATTCTGAGAAAGATCATAAGCACAAGCTGCTCTGCACACAAATAA
>Egret_OR14J1 (Egret73)
ATGCCCAATACCAGCTCCATCACCCAGTTCCTCCTCCTGGCCTTCGTCGACACACGGGA
GCTACAGCTCTTGCACTTCTGGCTCTTCCTGGGCATCTACCTGGCTGCCCTCCTGGGCA
ATGGACTCATCATCACCGCCATAGCCTGTGACCACCACCTCCACACCCCCATGTACTTC
TTCCTCCTCAACCTCTCCCTACTCGACCTGGGCTCCATTTCCACTACTGCGCCCAAATC
CATGGCCAATTCCCTCTGGGACACCAGGACCATCTCCTATGCAGGATGTGCTGCCCAGG
TCTCTGCGTTTGTCTTGTTTGTCACAGCAGAGTATTCTCTTCTCACTGTCATGGCCTATG
ACCGCTACGTTGCCATCTGCAAACCCCTGCACTACGGGACCCTCCTGGGCAGCAGAGC
TTGTGTCCACATGGCAGCAGCTGCCTGGGGCAGTGGCTTTCTCAATGCTGTCCTGCATA
CTGCCAATACATTTTCACTACCCCTCTGCAAGGGCAGTACCATAGGCCAGTTCTTCTGT
GAAATCCCCCAGATCCTCCTGCTTTCCTGCTCAGACACTTATCTCAGGGAATTTGGGCT
TATTGTGGTCAGTGCCTTTGCGGTCTTTGGGTGTTTTGTTTTCATTGTGGTGTCCTATGT
GCAGATCTTCAGGACTGTGCTAAGGATCCCCTCTCAGCAGGGGCGGCACAAAGCCTTT
TCCACGTGCCTCCCTCATTTGGCCGTGGTCTCTCTGTTTGTCAGCACTGTAGTGTTTGC
CTACCTGAAGCCCCCATCCATCTCCTCACCATCCCTGGATCTGCTGGTAGCAGTTTTGTA

CTCAGTGGTGCCTCCAGCAGTGAACCCCCTCATCTACAGCATGAGGAACCAGGAGCTC
AAGGAGGCCCTATGGAAAGTGGCACAATGGATGCTGTTTCACAGACCATGA
>Egret_OR14J1 (Egret74)
ATGACCAACAGCAGTTCCATCACCCAGTTCCTCCTCCTGGCATTCTCGGATACACGGAA
GCTGCAGCTCTTGCACTTTTGGCTCTTCCTGGGCATCTACCTGGCTTCCCTCCTGGGCA
ATGGCCTCATCATCACCGCCATAGCCTGCAACCACCGCTTCCACACCCCCATGTACTTC
TTCCTCCTCAACCTTTCCCTCATCAACCTGGCTTCCATCTCCACCACTGTCCCTGTATCC
ATGACCAATTCCCTCTGGGACACCATGGCCATCTCCTACTCTGGATGTGCTGCCCAATT
CTTTCTGTTTGTCTTTTTCCTTTCAGCAGAATTGTATCTTCTCACCGTCATGGCCTATGA
CCGTTACGTTGCCATCTGCAAACCCCTGCACTATGGGACCCTCCTGGGCAGCAGAGCT
TGTGTCCACATGGCAGCAGCTGCCTGGGGCAGTGCTTTTCTCAATTCTTCCCTTCACAC
TGCCAAGGCATTTTCTATTCCCCTCTGTGGAGGCAATGCTGTGCACCAGTTCTTCTGTG
ACATCCCCCAGATCATCAAGCTCTCCTGCTCACAATCCTACCTCAGGAAAGTTTGGCTT
CTTGTGGTCAGTGTCTGTTTAGCTTTTGTGTGTTTTGTTTTCATTGTGGTGTCCTATATGC
AGATCTTCAGGGCCGTGCTGAGGATCCCCTCTCAGCAGGGACGGCACAAAGCCTTTTC
CACGTGCCTCCCTCACCTGGCCGTGGTCTCCCTATTTATCAGCACTGGCATGTTTGCCTA
CTTGAAACCTCCCTCCATGTCCTCCCCATCACTGGATCTGATGGTGGCAGTGCTGTATG
CAGTGGTTCCTCCAGCAGTGAACCCCCTCATCTACAGCATGAGGAATAAGGAACTCAT
GGATTCTCTGAGAAAACTGATGACTGGATGTTTTCATAAGCAATAA
>Egret_OR14A16 (Egret75)
ATGTCCAACAGTAGCTCCATCACGGAGTTCCTCCTCCTGGCGTTTGCAGACACATGGG
AGCTGCAGCTCTTGCACTTCTGGCTCTTCCTGGGCATCTACCTGGCTGCCCTCCTGGGC
AACGGCCTCATCATTGCAGCTGTAGCCTGTGACCACCGCCTCCACACCCCCATGTACTT
CTTCCTCTTCAACCTCTCCCTCCTTGACCTGGGCTCCATCAGCACCATTGTCCCTAAATC
CATGGCCAATTCCCTGTTGGATACCAGGGCCATCTCCTTCTTTGGGTGCGCTACTCAAA
CCTTTTGGTTCCTCTTTTTAGTTGTAGCAGAGTATTCCCTTCTCACTGTCATGGCCTATG
ACCGCTATGTTGCCATCTGCAAACCCCTGCACTACGGGACCCTCCTGGGCAGCAGAGC
TTGTGTCCACATGGCAGCAGCTGCCTGGGGCTCTGGGTTTCTCTATGCTGTGCTGCACA
CAGCCAATACATTTTCCCTACCCCTCTGCCATGGAAATGCTGTGGACCAGTTCTTCTGT
GAAATACCTAAGATTCTCAAGCTCTCCTGCTCAGGCTCCTACCTTAAGGAAGTTCGGCT
CATTGTGGCTGGTGTCTGTTTATTCTCTGGGTGTTTTGTTTTCATTGTGCTGTCCTATGTG
CAGATCTTCAGGGCCGTGCTGAGGATCCCCTCTCAGCAGGGACAGCACAAAGCCTTTT
CCACGTGCCTCCCTCACCTGGCTGTGGTCTCTCTGTTCGTCACCACAGGAATGTTTGCC
TACCTGCATCCCCCCTCCATCTCCTCCCCATGCCTGGATCTGGTGATGGCAGTGCTGTAC
TCAGTTCTGCCTCCGACAGTGAACCCCCTCATCTATAGTGTCAGGAACCAGGAGCTCA
AAGATGCCCTGAAGAAACAGATCAGGCATTTTTCAGCAGCCATAGACTGTGTACCTTC
CTCTGCAAATGACTCCCAGTGTACGTCATGA
>Egret_OR14J1 (Egret76)
ATGGAAGTGCCCCATTGTGAGAGGAAGAAAATGTCCAATGGTACATCCATCACGCAGT
TCCTCCTCCTGGCATTCTCAGACACAAGAGAGCTGCAGCTCTTGCACTTCTGGCTCTTC
CTGGTCATCTACCTGGCTGCCCTCCTGGGAAACGGCCTTGTCATCACCACCATAGCCTG
CGACCACCGCCTTCACACCCCCATGTACTTCTTCTTCCTTAACCTCTCCCTCCTCGACCT
GGGATCCATCTCTACCACTGTTCCCAAATCCATGGTGAATTCCCTGTGGGAAACCAGGG
ACATTTCCTACTCAGGATGTGTTGCCCAGGTCTTTCTGTTTATCTTTTTGTTATCGGCAG

AATATTATATTCTCACTGTCATGGCCTATGATCGCTACGTGGCCATCTGCAAACCCCTGC
ACTATGGGACTCTCCTGGGCAGCAGAGCTTGTGTCCACATCGTAGCAGCTGCCTGGGG
CAGTGGGTTCCTCAATGCTGTGCTGCACACAGCCAGTATGCTTTCCATACCTCTGTGTA
AGGGCAATGCTGTGGACCAATTCTTCTGTGAAATCCCCAGATCCTCAAGCTCTCCTGC
TCAGATTCCAACCTCAGGGAAGTTGGGCTTCTTGTGCTTAGTTTTTGTTTCTCTATTGG
GTGTTTTGTTTTCATCCTGATGTCCTATGTGCAGATCTTCAAGGCCGTGCTGAGGATCCC
CTCTCAGCAGGGACGGCACAAAGCCTTTTCCACGTGCCTCCCTCACCTGGCCATGGTC
TCCTTGTTTATCATCACTGCCATTATTTCCTACCTTAAGCCCCCCTCCATCTCTTCCACAT
CTCTGGATCTGGTAGTGGCAGTTCTCTACTCAGTGGTGCCTCCAGCAGTGAACCCTCTC
ATCTACAGCATGAGGAACCAGGAGCTCAACGACGCACTAAAGAAGCTGATTCAATCAG
TTGTGTTTCTGCAGCAATAA
>Egret_OR14J1 (Egret77)
ATGTCCAACAGCAGCTCCATCACCCAGTTCCTCCTCCTGGCATTTCCAGATCTCTGGGA
GCTGCAGCTCTTGCACTTCTGGCTCTTCCTGGGCATCTCCCTGGCTGCCCTCCTGGGCA
ATGGCCTCATCATCACCACCATAGCCTGTGTCCACCACCTCCACACCCCCATGTACTTC
TTCCTCCTCAACCTCTCCCTCCTTGACCTGGCCTCCATCTCCACCATTGTTCCCAAATCA
ATGGCAAATTTGCTGGGAGGAAAAAGGACCATTTCCTACCAAGGGTGTGTGGTCCAAC
TCTTTTATTTTTTCTTTCTTATGTCAGCAGAATATTCTCTTCTCACCGTCATGGCCTATGA
CCGCTATGTTGCCATCTGCAAACCCCTGCACCACGGGACCCTCCTGGGCAGCAGAGCT
TGTGTCCACATGGCAGCAGCTGCCTGGGGCAGTGGGTTCATCAATTCTCTCCTGCACA
CGGCCAGTACATTTTCCCTACCCCTCTGCCAAGGCAATTCTCTGGACCAGTTCTTCTGT
GAAATCCCACAGGTCCTCAAGCTCTCCTGCTCAGATGCCTACCTCAAGGAAGTTGGGC
TCATTGTGGTCAGTGCCTGTTTATGCTTCGGGTGTTTTGTTTTCATTGTGCTGTCCTATG
TGCAGATCTTCAGGGCTGTGCTGAGGATCCCCTCTCAGCAGGGACGGTACAAAGTCTT
TTCCACGTGCCTGCCTCACCTGGCCGTGGTCTCCCTGTTTCTCAGCACTGCAATGTTTG
CCTACCTGAAGCCCCCCTCTATCTCCTCCCCATCTCTGGATCTGGTGGTGGCAGTCCTGT
ACTCAGTAGTGCCTCCAGCAATGAACCCCCTCATCTACAGCATGAGGAACAAGGAGAT
CAAGGATGCCCTATGGGAACTGTTCCAATGGACATGGTTTAATCAACAGTAA
>Egret_OR14J1 (Egret78)
ATGTCCAACAGCAGCTCCATCACCCAGTTCCTCCTCCTGGCATTCGCAGACACACGGG
AGCTGCAGCTCTTGCACTTCTGGCTCTTCCTGGGCATCTACCTGGCTGCCCTCCTGGGC
AACGGCCTCATCATCACCGCCATAGCCTGCGACCACCACCTCCACACCCCCATGTACTT
CTTCCTCCTCAACCTCTCCCTCCTTGACCTGGGATCCATCTCTACCATTGTCCCCAAATC
CATGGCCAGTTCCCTCTGGAACACCAGGGCCATTTCCTATGCAGGATGTGCTGCACAG
GCCTTTCTGTTTGCCTTCTTGGTAGCAGCAGAGTATTCTCTTCTCACTGTCATGGCCTAT
GACCGCTACGTTGCCATCTGCAAACCCCTGCACTACGGGACCCTCCTGGGCAGCAGAG
CTTGTGTCCACATGGCAGCAGCTGCCTGGGGCAGTGGGTTCCTCAGTGCTCTCTCCTG
CACACTCAATACTTTTTCAGTGCCCCTCTGCAAGGGCAATGCTTTGGGCCAGTTCTTCT
GTGAAATCCCCCAGATCCTCAAGCTCTCCTGCTCAGATGCCTACCTCAGGGAAATTTGG
CTTCTTGTATTCAGTGTCTCTATTGCATTTGGGTGTTTTATTTTCATTGTGCTGTCTTATGT
GCAGATCTTCAGGGCTGTGCTGAGGATCCCCTCTGAGCAGGGATGGCACAAAGTCTTT
TTCACATGCCTCCCTCACCTGGCTGTAGTCTCCCTGCTTGTCAGCACTGGCATGTTTGC
TTACCTGAAGCCTTCCTCCATCTCCTCCCCATCCCTGGATCTTGTGGTGGCAGTGCTTTA
CTCAGTGGTGCCTCCGACATTGAACCCTCTCATCTATAGCATGAGGAACCAGGAGCTC

AAGGCTGCCCTGAGGAAACTGATCACACGCTTTTCAACAGCCATAGACCGTGTACCTT
CCTCCACAAATGACTCCCAGAGTCTGGCGTGTCAAGGCAGGGTGGGTCATTTTTATTTG
TTCACTTGCTTTATTTTCCTTTTTCAACTTCTGATGATGTTGTCTGCAAAAACCCCCCTC
CTTCTCAAGTATCCATCATGTTGTGTGACCCAGAAAGTTTGTGTGAATAAGGAGCGAG
GCTCTCTGTGTATTTAA
>Egret_OR14J1 (Egret79)
ATGTCCAACAGCAGCTCCACCACTCAGTTCCTCCTCCTGGCATTCGCAGACACACGGG
AGCTGCAGCTCTTGCACTTCTGGCTCTTCCTGGGCATCTACCTGGCTGCCCTCCTGGGC
AACGGCCTCATCATCACCGCCATAGCCTGTGACCACCGCCTCCACACCCCCATGTACTT
CTTCCTCCTCAACCTCTCCCTCCTCGATCTGGGATCCATCTCCACCAATGTTCCCAAGTC
CATGGCCAATTCCATCTGGGATACCAGGGCCATCTCCTATGCAGGATGTGCTGCCCAGG
TCTTTATATTTATGTTTTTGATGTCAGCAGAGTATTATCTTCTCATTGCCATGGCTTATGAC
CGCTACGTTGCCATCTGCAAACCCCTGCACTATGGGACCCTCCTGGGCAGCAGAGCTT
GTGTCCACATGGCAGCAGCTGCCTGGAGCATTGGATTTCTCACTGCTGTGCTGCACAC
GGCCAATACATTTTCACTTCCCCTGTGCCAAGGCAATGATGTGGAGCAGTTCTTCTGTG
AAGTCCCCCAGATCCTCAGGCTCTCCTGCTCAGACTCCTACCTCAGGGAATTTGGGCTT
ATTGTGTTTAGTGTGTCTGTGGGCTTTGTTTTTTTCATTTTCATTGTGGTGTCCTATGTGC
AGATCTTTAGGGCTGTGCTGAGGATCCCCTCTCAGAAGGGGTGGCACAAAGCCTTTTC
CACATGCCTCCCTCATATGGCTGTGGTCTCCCTATTTCTCAGCACTGTAATGTTTGCCCA
CCTAAAGCCCCCCTCCATCTCCTCCCCATCCCTGGATCTCATGGTGGCAGTTCTCTACTC
AGTGGTGCCTCCAGCAGTGAACCCCCTCATCTACAGCATGAGGAACCAGGAGCTTAAA
AAGGCCCTTTGGAAACTGATGCAATGGGTGCTATTTCACTGGCAATAA
>Egret_OR12D3 (Egret80)
ATGCTGAACCAGACGGAGGTCCGTGAGTTCATCCTTTTGGGCCTCACCGATACGCGGG
GGCTGCAGCACTTTTTCTTTGTCTCCTTTCTGTTGCTCTACTTGAGCAGTCTTCTGGGC
AATGGTGCCATTGTGACCATGGTGGTATCTGAGCCCCAGCTCCACACACCGATGTACTT
CTTCCTGGGGAACCTCTCCTGCCTGGACATTTTCTACTCCACAGTCACTGTTCCCAAGA
TGTTGACTGGCTTTCTCTTTGGGCATCAGCCCATCTCTTTTGGTGGCTGCTTGGCCCAG
CTCCACTTCTTTCACTTCCTGGGCAGTACTGAGGCCGTGCTCCTGGCTGCCATGGCCTA
CGACCGCTATGTGGCCATTTGCAACCCTTTGCGCTATGCACTTGTCATGAGTCCACGGA
CCTGTCTGCTGCTGGCTGTGGCCAGCTGGTCCACTGGCTTTGTACATGCCATGATGCAC
TCAGTCATGACCTCTCGACTGAGCTTCTGTGGCCACAACCACATTCATCACTTCTTCTG
TGACATCAAGGCACTCCTGAGTTTGGCTTGCAGCAGTACCAGCCTCAACATGACCCTC
CTCAATGTCGTCACCACATCTATTGCTCTATGCCCCTTCACTCTCACAATCCTCTCTTAC
CTCTACATCATCTCCTTCCTCATCTATAAAGTCCAGTCCCATGAAGGAAGATGGAAGCC
CTTCTCCACCTGCGCCTCCCACCTCACCGTTGTAGCACTGCTTTACATACCAGTGCTCTT
AAATTATACACCACCCTCCTCAGGAAGCTCCTTGAAAAGGGATGTGCACGTGTCTCTCA
TGTACAGTGCTGTCACCCCAGCTCTGAACCCCTTGATCTACACTCTTAGGAACCAGGA
GGTGAGATCTGTCCTGAAAAAAATATTAGGGAGAAAACTCTTTCCTGGAGGAAAGTGA
>Egret_OR14J1 (Egret81)
ATGTCCAACAGTAGCTCCATCACCCAGTTCCTCCTCCTGGCCTTCGCAGACACACGGG
AGATGCAGCTCTTGCACTTCTGGCTCTTCCTGGGCATCTACCTGGCTGCCCTCCTGGGA
AATGGCCTCATCATCACCGTTGTAGCCTGCGACCACCGCCTCCACACCCCCATGTACTT
CTTCCTCCTCAACCTCTCCCTCCTCGACCTGGGATCCATCTCCACTACTCTCCCCAAATC

CATGGCCAACTCCTTCTGGGACATAACAGCCATCTCATATGTAGGATGTGTTGTCCAGC
TCTTTATGTTTGTATTTTTGCTTTCAGCAGAGTATTTTCTCCTCACTGTCATGTCCTATGA
CCGCTATGTTGCCATCTGCAAACCCCTGCACTATGGGACTCTCCTGGGCACCAGAGCTT
GTGTCCACATGGCAGCAGCTGCCTGGGGCAGTGGCTTTCTCAGTGCTGTGCTACACAT
GGCCAATGCATTTTCACTCCCCCTCTGCAAGGGCAATGCCTTGGATCAGTTCTACTGTG
AAGTCCCCAAGATCCTCAAGCTCTCTTGCTCAAACTCCTATTTCAGGGCAGTTGGGCTT
ATTGTAGTCAGTGTCTGTTTAGCACTTGGCTGTTTTGTTTTCATTGTGGTGTCCTATGTG
CAGATCTTCAGGGCTGTGATGAGGATTCCCTCTGAGCAGGGACGACACAAAGCCTTTT
CCACGTGCCTCCCTCACTTGGCCGTCGTCTCTCTGTTTCTCAGCACTGGCATATTTTCCA
ACCTGAAACCCCCTTCCACCTCTTACCCATCCCTGGACCTGGTGGTGTCATTTCTGTAC
TCAGTGGTTCCTCCAGCAGTGAACCCCATCATCTACAGCATGAGGAACAAGGAGCTCA
TGGATTCCCTGAGGAAACTGATGACTGAGTTTTTTCAGAGGAAATGA
>Egret_OR2AT4 (Egret82)
ATGGAAAGCTGTGGCAGCAATGCTTCCACTAAAGTATTTTTCCTGGTGGGTTTTCCAGC
TCTCCAGGATTTTCAGACCCCCCTCTTCGTTGTGTTCTTGCTGTTCTACCTGCTGATCCT
GGGTGGCAATGCCGTTATCATCACCGTGGTGGTGGTCGACCGCATGCTCCACAAACCC
ATGTACTTCTTCCTGATTAACCTCTCTGTGTTAGACGTGCTATTCACAACCACCACCATC
CCCAAAATGCTGGCAATGTTCCTGGCCAACGCTAAAACCATCTCGTTTCCGGGCTGCTT
TCTGCAGATGTACAGTTTTCACGGGTTGACGGTAACCGAGGCGCTTCTCCTGGTGGTC
ATGGCTTACGACCGCTATGAAGCCATCTGCAACCCCCTCCATTACCCGGCCAAGATGAC
AAGAAGAGTGAACGTCCAGCTGGCAGCGAGCGCCTGGCTCACCGCGCTGCTAATCCC
CGTGCCCGTCATCATGCAGACCTCTCAGCTAACTTACGGGGACACAACCAGGGTTTAC
CACTGCTTTTGTGACCACCTGGCCGTGGTACAAGCTGCGTGCTCAGACTTCAGCGCCG
AGTTCCAGACCTTCTTGGGGTTCTCCATCGCTATGACAGTGTCGGTCATCCCTCTGTTG
CTCGTCACCCTCTCGTATGTCCACATCATCCTCTCCGTGCTAAAGATCAACTCCACAGA
AGGACGCATGAAAGCTTTTTCAACGTGTAGCTCCCATCTGCTTGTGGTGGGCACCTACT
ACTCCTCCATCGCTGTGGCGTACGTGTCCTACAGAGCGGACATCCCTGTTGATGTCCAT
GTCATGAGCAACGTTGCCTTCTCTATTTTAACTCCCTTGTTAAACCCCATCATTTACACT
TTACGGAACAAGGAAGTAAAATCTGCCATTAAAAAGTCTATTTTTCTGAAAATCTTTCC
CTTTTCTGAAAAAAATTAA
>Egret_OR14J1 (Egret83)
ATGTCCAACAGCAGCTCTATCACCCACTTCCTCCTCCTGGCATTCGTGGAGACACGGG
AGCTGCAGCTCTTGCACTTCTGGCTCTTCCTGGGCGTCTACCTGGCTGCCCTCCTGGGA
AACAGCCTCATCATCACCGCCATAGCCTGCGACCACCGCCTCCACACCCCCATGTACTT
CTTCCTCCTCAATCTCTCCCTCCTTGACATGGCCTCCATCTCCACCACTGTCCCTAAATC
CATGGCTAATTCCCTCTGGGACACCAGGGCAATTTCCTATGTAGGATGTGCTGCCCAGC
TATTTATGTTTGCCTTCTTGGTAGCAGCAGAGTATTGTCTTCTCACCATCATGGCCTATG
ACCGCTACGTTGCCATCTGCAAACCCCTGCGCTACAGGACCCTCCTGGGCAGCAGAGC
TTGTGTCCATGTGACAGCAGCTGCCTGGGGCAGCGGCTTTCTCAATGCTCTCCTGCAG
ACGGCCAATACATTTTCCCTACCCCTCTGCAAGGGCAATGCTGTAGAGCAGTTCTTCTG
TGAAATCCCTCAGATCCTCAAGCTCTACTGCTCAGAATCAGACTATCTCAGGGAAGCTG
GGCTTCTTGTACTTAGTTTTTGTTTAGCAGTTGGTTGTTTTGTGTTCATTGTGCTGTCCTA
TGTGCAGATCTTCAGGGCCGTGCTGAGGATCCCCTCTCAGCGGGGACGACACAAAGC
CTTTTCCATGTGCCTCCCTCACCTGGCCGTGGTCTCCCTGTTTCTCAGCACTGGCATGT

TTGCCTACCTGAAGCCCCCCTCCATCTCATCCCCATCCATGGATCTGGTGCTGGCTGTTC
TGTACTCAGTAGTGCCTCCAGCAGTGAACCCCTTCATCTACAGCATGAGGAACCAGGA
GCTCAAAGATGCACTCAGGAAGCTGATTCCATCGGTAGTATTTCAGCAGCAGTTAGCTC
CCCATTTGTCTTCAACAAGTGGTTTCTAG
>Egret_OR14A16 (Egret84)
ATGAGTGAGCAAACGTCCAACAGCAGCTCCGTCATCCAGTTCCTCCTCCTGGCATTCG
CAGACACACGAGAGCTGCAGCTCTTGCATTTCTGGCTCTTCCTGGGCATCTACCTGGCT
GCCCTCCTGGGCAACGGCCTCATCATCATCACTGTAGCCTGTGACCACCGCCTCCACAC
CCCCATGTACTTCTTCCTCTTCAACCTCGCCCTCCTTGACCTGGGATACATCTCTACCAC
TGCCCCCAAGTCCATGGCCAATTCCCTATGGAACACCAGGGCCATCTCTTTTTTGGGCT
GTGCTGTTCAAGTCTTTCTGTTTCTCTTCTTCATTTCAGCAGAGTTTTCTCTTCTCACCA
TCATGGCCTATGACCGCTACGTTGCCATCTGCAAACCCCTGCACTACGGGACCCTCCTG
GGCAGCAGAGCTTGTGTCCACATGACAGAGGCTGCCTGGGGCACTGGGTTCCTCTATG
CTTTGCTGCACACAGCCAATACATTTACACTACCCCTTTGCCAAGGCAATGCTGTGGAC
CAGTTCTTCTGTGAAATCCCCCAGATCCTCAAGCTCTCCTGCTCAGACGCCTACCTCAG
GGAGGTTGGGCTTCTTATAGTTAGCATCTGTTTAGGCTTTGGGTGTTTCATTTCCATTGT
GCTGTCCTATGTGCAGATCTTCAGGTCTGTTCTGAGGATCCCCCCTCAGCAGAGGCGGC
ACAAAGCCTTTTCCACGTGCCTCCCTCACCTGGTTGTGATCTCCCTGTTTCTCAGCACT
GCCATGTTTGCCTACCTGAAGCCCCCATCCATCTCATCCCCATCCCTGGATATGGTGGTC
TGTTCTGTACTCGGTGGTGCCTCCAGCAGTGAACCCCCTCATCTACAGCATGAGGAAC
AGGGAGCTCAAGGATGCAGTGTGGAAACTGATAACTTCATGTTTTTAAGCAGCAACAA
GCTTCCCATCGTCTTCTGCATAGTACTTATGGTGTAA
>Egret_OR14A16 (Egret85)
ATGTCCAACATCAGCTCCATCATCCAGTTCATCCTCCTGGCATTTGCAGACACATGGGA
GCTGCAGCTCTTGCACTTCTGGCTCTTCCTGGGCATCTACCTGGCTGCCCTCCTGGGCA
ATGGCCTCATCATCACCGCCATAGCCTGCAACCACCGCATCCCCACCACTATGTACTTCT
TCCTCCTCAACCTCTCCCTCCTCGACCTGGGATCCATCTCTACCACTGTCCCCAAAGCC
ATGGCTAATTCCCTCTGGGACACCAGGGACATTTCCCACACAGGATGTGTTGCACAAG
TCTTTCTGGTTATCTTTTTCATGTCAGCAGAACTGTGTCTTCTCACTGTCATGGCCTATG
TCCATCACATTGCCATTTGCAAACCCCTGCACTATGGGACCCTGCTGGGCAGCAGAACT
TGTGTCCACATGGCAGCAGCTGCCTGGGGCAGGGGCTTTTCCAATGCCACGGCCAATA
CTTTTTCCATATCCTTCTGCCATGGCAATGCCGTGGACCAGTTCTTCTGTGAAATCCCCC
AGATACTCAAGCTCTCCTGCTCAAATGCTTATCTAAGGGAGGTTGGGCTTCTTGTGTTT
AGTGTCTGTATTACATTTGGGTGTTTTGTTTTTATTGTGGTGTCCTATGTGCAGATCTTCA
GGGCTGTGCTGAGGATCCCCTCTCAGCAGGGACGGCACAAAGCCTTTTGCACGTGCCT
CCCTCACCTGGCCATGGTCTCCCTGTTTCTCAGCACAGGCATGTTTGCCTACCTGAAGA
CTTCCTCCTTTCCTTCCCCACCCCTGGATCTGTTGATGGCGGTTCTGTACATGGTGGTGC
CTCTAGCACTGAACCCCCTCATCTACAGCATGAGGTACCAGGAGCTCCAAGATGCAGT
GTGA
>Egret_OR14J1 (Egret86)
ATGTCCAACAGCAGCTCCATCACCCAGTTCCTCCTCCTGGCATTTGCAGACATGCGGG
AGCTGCAGCTCTTGCACTTCTGGCTCTTCCTGGGCATCTACCTGGCAGCCATCCTGGGC
AACGGACTCATCATCACCGCCATAGCCTGTGACCACCGCCTCCACACACCCATGTACTT
CTTCCTCCTCAACCTCTCTGTACTTGACATGGGCTCTGTCTCCACCACTGTCCCCCTATC

CATGGCCAATTCCCTCTGGGATACCCGCACAATCTCCTACTCAGGATGTGCTGCCCAGG
TCTTCTTTCTCTTCTTCTTTTTTGGTGCCGAGTATTCCCTTCTCACGGTCATGGCCTATGA
CCGCTACATTGCCATCTGCAAACCCCTGCACTATGGGACCCTCCAGGGCAGCAGAGCT
TGTGTCTACATGGCAGCAGCTGCCTGGGGCAGTGGCTTTCTCCATGGGCTGCTGCACA
CTGCCAACACATTTTCGCTACCTCTCTGCCAAGGCAATGCTGTGGACCAGTTCTTCTGT
GAAATCCCCCAGATCCTCAGGCTCTCCTGTTCAGACTCCTACCTCAGGGAAGTTGGCC
TTCTTGTGTTTAGTCTTGCTTTAGCTTTTGGGTGTTTTGTTTTCATTGTGCTTTCCTATGT
GCAGATCTTCAGGGCCGTGCTGAGGATCCCCTCTGAGCAGGGACGACACAAAGCTATT
TCCACCTGCCTCCCTCATTTGGCCGTGGTCTCCTTGTTTCTTAGCAGTTGTATGATTGCC
TACCTGAAGCCTCCCTCCATCTCTTCTCCATCCCTGGACCTGGTGGTGGCAGTTCTGTA
CTCGGTGGTGCCTCCAGCAGTGAATCCCCTCATCTACAGCATGAGGAACCAGGAGCTT
AAGGGCGGCTTGAGAAAACTCATTTAA
>Egret_OR14J1 (Egret87)
ATGGAGCTGACGGAAGGTCACCTGAAAAAGGAATGGGTAGCGAGGAGTATCACATGG
TGTAGGTCTAGGAGAAAGGCTTTGATTTTCCTCAGAGAAGTCTCCCTTAACTCTTCATT
GACTCTCCCTCTAAGAACAGGTCCCAATGCTCAGAGGAAGCTACTTTTCAACAGCAGC
TCCATCACCCAGTTCCTCCTCATGGCATTCACAAACACACGGGAGCTGCAGCTCTTGC
ACTTCTGGCTCTTCCTGGGCATCTACCTGGCTGCCCTCCTGGGCAACGGCCTCATCATC
ACCACCATAGCCTGCGACCACCGCCTCCACACCCCCATGTACTTCTTCCTCCTCAACCT
CTTTGTCATCGACCTGGCCTCCATTTCGACCACAGTCGCCAAATCCATGGCCAGTTTCC
TCTGGGACACCAGAGACATCTCCTATGCAGGACGTGTTGTTCAGCTCTTTGTCTTTGCC
TTCTTGGTAACAGCAGAGTATTTTCTTCTCACTGTCATGGCCTATGACCGCTATGTTGCC
ATCTGCAAACCCCTGCAATACAGGACCCTCCTGGGCAGCAGAGCTTGTGCCCACATGG
CAGCTGCTGCCTGGGGCAGTGCATTTTTCACTGCTCTCCTTCACACAGTCAATACATTT
TCACTCCCTCTCTGCCAAGGCAATGCTCTGGACCAGTTCTTTTGTGAAATCCTCCAGAT
CCTCAAGCTCTCCTGCACAGATGCGTATCTCAGAGAGGTGTGGCTTATTGGTTTTAGTG
CCTTTATTGTCTTTAGGTGTTTTGTTTTCATCGTGTTGTCCTACATGCAGATCTTCAGGGT
TCTGCTGAGGATCTCCTCTCAGCGGGGACAGCACAAAATCTTTTCCATGTGCCTCCCTC
ACCTGGCCGTGGTCTCCCTGTTTATCAGCACTGCAGTGTTTGACCACCTGAAGCCTCCC
TCCATCTCCTCCCCATCCCTGGATCTGGTGGTGGCAGTGCTGTACTCAGTGGTTCCTCC
AGCAGTGAACCCCATCATCTACAGCATGAGGAACCAGGAGCTCCAGGATGGAGTGTG
GAAACTGATCCAATGGGTTCGATTTGTCCAATAA
>Egret_OR52R1 (Egret88)
ATGCCTCTGAACTCTACTGCCTCCTCCCACCCTCCCTCTTTCCTACTCATTGGCATCCCT
GGGCTGGAGAAGGAGCAGTTCTGGATTGCCTTCCCCTTCTGCGTCATGTATGCCATTGC
TGTTCTGGGGAACATCACCCTTCTCCTCATTATAAAGGCAGAGACGAGCCTGCAAGAG
CCCATGTACCTCTTCCTGGCCATGCTGGCCCTCACTGACCTGGTCCTATCAACATCCATG
CTACCCAAAATGCTTGGCATCTTCTGGCTGGGCTCCGGGGAGATTGGGTTTCTCTCCTG
CCTTGCTCAGTTGTTCTTCATCCATACCTTCTCATCGGTGGAGTCAGGCGTGCTCATGG
CCATGGCCTTGGATCGCTACATCGCTATTTGCTGCCCACTGCGGCATTCCAGCATCCTTT
CTGTGCCGGTGGTGGTGGCCCTCGGGAGCCTGGTGCTGGTGCGTGGGGTCGTCCTGGT
GAGTCCCTTCTGCTTCCTCCTCCACAGGATGCCCTTCTGCCAGCATCACATCATCTCCC
ACTCCTACTGTGAGCACATGGCCGTGGTGAAGCTGGCATGCGGGGACACCAGAGTCAA
TGTCATTTATGGCCTCTTTGTGGCTTTCACAGTGACAGGATCTGACATGATCCTGATCTC

TGTGTCCTACACCATGATCCTGCGGGTGGTAATGAGGCTGTCATCCACAGAGGCACGG
CTGAAAGCCTTCAGCACTTGCGCATCCCATGTCTGTGTCATACTCGCCTTTTATGTCCCT
GCCCTCTTCACATTCCTCACCCACCGGTTTGGGCAGAGCGTCCCTCCCCACGTCCATAT
AATGGTGGCAAATCTCTACCTGCTAGTGCCCCCCATGTTAAACCCCATTGTTTATGGGG
TGAGAACCAGGAAGCTCTGGGACAGGGTGGTCCTCCTCTTCCAGCCAAAGGGAACCT
GA
>Egret_OR5AR1 (Egret89)
ATGGCGGCCAAAGGAAATTGCACCCCCAGTGCCGAATTTGTTCTCTTGGGGTTCTCAG
AGCAGGGAGATGTCCAGGCTGTCCTCTTCACAGTCTTCTTGGTGATCTACGTGATCACT
CTGCTGGGGAATCTGGGGATGCTGGTGTTAATCAGGCTGGACGCCCAGCTTCACACCC
CGATGTACTTCTTCCTGAGCAGCCTGTCCTTCCTAGACATCTGCTACTCCTCCTCAATTG
CCCCCCGACTGCTCTCAGATCTCCTGGCAGAACAGAAGGTCATTTCTCACTCTGCGTG
CCTCGCACAATTTTATTTCTACGCAGTCTTTGCCACCACTGAGTGCTACCTCTTGGCTGT
GATGGCATATGACCGCTACGTGGCCATCTGCAGCCCACTGCTCTATGCCATCTCCATGTC
CAGCAGAGTGTGTGCGCTTCTGGTAGCTGGCTCATACCTCGCTGGGATCGTGAATGCC
GCCATCCACACGGGGCTTGCACTTCGGCTCTCCTTCTGCAGTCCCACCATCATCAACCA
CTTTTACTGTGAGGGGCCCCCGCTTTACGCCATCTCTCACACGAACCCCACCGTCAATG
AGATTACAATGTTTGTGGTTGTTGGCTTCAACCTGTTTGTCACCAGCCTGACCATCCTC
ATCTCCTACACCTACATCCTGGCCACCATCCTGAGGATGTGCTCAGGCGTGGGCAAGC
ACAAAGCCTTCTCCACGTGCACGTCCCACCTGACCGCTGTGACCCTCTTCTACGGATCT
GCTGCGTCCATGTACTCACGATCCAGCTCCAGACGCTCCCAGAACCTTGACAAAGTGG
CCTCTGTGTTTTACACCATGGTGACCCCCATGCTGAACCCCCTCATCTACAGCCTGAGG
AACAAGGATGTGAAGAATGCGCTGGGGAGAGCGGTGGAGAGGAAACGTTTGTCTGAA
AAATAG
>Egret_OR14J1 (Egret90)
ATGTCCAACAGCACCTCCATCACCCAGTTCCTCCTCCTGGCATTTGCAGATACACGGGA
GCTGCAGCTCTTGCACTTTGGGCTCTTCCTGGGCATCTACCTGGCTGCCCTCTTAGGCA
ACAGCCTCATCATCACTGCCATAGCCTGCGACCACCGCCTCCACATCCCCATGTACTTC
TTCCTATTAAACCTCTCCCTTGTTGACCTGGGCTCCATCTCTGCCACCATCCCCAAATCC
ATGGCCAATTCCCTGTGGGACACAAGGGTTATCTCCTATGAAGGGTGTGCTGCCCAGCT
CTTTTTCTTTTTCTTTTTTATGTCAGTGGAGTACTTTCTTCTCACTGTCATGGCCTATGAC
CGCTACGTTGCCATCTGTAAACCTCTGCAGTACGGGACCCTCATGGGCAGCAGAGCTT
GTGTCCACATGGCAGCAGCTGCCTGGGCCTGTGGGTTCCTCAATTCTCTTGTGCACACT
GCCAGTACATTGTCACTCCCACTCTGCCAAGGCAATGCTGTCGACCAGTTCTTCTGTGA
AATCCCTCAGATCCTCAAGCTCTCCTGCTCAGACGCCTACCTCCGTGAGGTTGGGCTGC
TTGTGGTTAGTGCCTGTTTAGTGTTCTGGTGTTTTGTTTTCATTGTCCTGTCCTATGTGC
AGATCTTCAGGGCCGTACTGAGGATCCCCTCTCAGCAGGGACGGCAGAAAGCCTTTTC
CACGTGCCTCCCTCACCTGGTTGTGGTCTCCCTCTTTGTCAGCACTGGCACGTTTGCCT
ACCTGAAGCCGCCCACCATCTCCTCCCCATTCCTGGATCTGGTGGTGGCAATTCTATAC
TTGGTGGTGCCTCCAGCAGTGAACCCCCTCATCTACAGCATGAGGAACCAGGAGCTCA
AGGAGGCCCTATGGAAACTGGCACAACTGACACTGTTTCACCGACAATAA
>Egret_OR52B2 (Egret91)
ATGTTGCATCTGGCCATGCCAGCAGCCAACCTGTCAGGATCCCACCCCATGTTCTTCAT
CTTGGTGGGGATACCTGGGATGGAGAAGTCTCACACCTGGATCTCCATCCCTGTCTGCA

TGATGTACTCTGCTGCCCTTCTCGGGAACGTGGTCCTGCTGGTCACCATCATGAAGGAC
CACAGCCTCCATGAGCCCATGTGCATCTTCCTGTGCATGCTGGCAGTGTGCGACCTGCT
GTTGTCCACTGCCACTGTGCCCAAGACCCTCAGCGTTCTCTGGTCTGTCTCCACCAAA
ATCTCTTTCAGTGGCTGCCTGGTGCAGAAGTTCTCCATCTATTTGTTTTTGTGGCGGA
GTCAGCCATTTTACTGGCAATGGCCTTCGATCGGTACATCGCCATTTGTGACCCCCTGC
AATACCCAGCCATCCTGACATGGTCCGTCATGGGGAAAGTTGGCATAGTGGCTTTAATC
AGGAGTTTCTGCATCATGTTCCCACCCATCTTCCTCCTCAAGCGGCTGCCCTACTGCGG
GCACAACGTCATGCCCCACACCTACTGCGAGCACATCGGCATGGCCCGCCTGGCGTGC
GCCGACATCTCCATCAACATCTGGTATGGGGTAGCTTCTGGCTTCCTTTCAGCCGGCTT
GGATGCCATCTCCATCACCATCTCCTACGTGCTGATCTTCAGGTGCCTCTGGGGGCTGC
TGTCCTGGCGCGCCTCCCCCAAGGCTCTGCACACCTGCGGCTCCCATGTTTTACACGCC
ATGTTCATCGCCATGTTTTACACGCCAGCCTTCTTCTCCTTTCTAGCACACTGGTTTGGC
CAGCATGTTCCTCAGCATGTCCTTGCCTCCTTCGCCAACCTCTACGTCGTGGTGCCGCC
CATGCTCAACCCCATCATCTACGGGGTGAGAACAAGGCAGATCCGGGAGCAAGTGGCT
CACTTGCTCAGGCTGGGGGGCTGGGGCGGCCGGGCATAG
>Egret_OR14A16 (Egret92)
ATGACCAATGGCAGCTCAATGAACAGATTCCTCCTCCTGGCCTTCGTGGACATGCGGG
AGCTGCAGCTCCTGCACTTCTGGCTCTTCCTGGGCATCTACCTGGCTGCCCTCCTGGGC
AACGGCCTCATCATCACCGCCATAGCCTGCGACCACCACCTCCACACCCCCATGTACTT
CTTCCTCTTCAACCTCTCCCTCCTTGACTTGGGCTCCATCTCCACCATTGTCCCCAAATC
AATGGCAAATTCTCTCTGGGACACCAGGGCCATTTCCTACGCAGGATGTGCTGCCCAG
GTCTTTCTGTTTGTTTTCTTTGTTGGAGCAGAGATTTCTCTTCTCACCATCATGTCCTTT
GACCGCTACATTGCCATCTGCAAACCCCTGCACTACGGGACCCTCCTGGGCAGCAGAG
CTTGTGTCCACATGGCAGCAGCTGCCTGGGGTGGTGGGCTCCTCTATGCTGTCCTTCAC
ACAGCCAATACGTTTTCCATTCCCCTCTGCCAAGGCAATGCTGTAGACCAGTTCTTCTG
TGAGATCCCCCAGATCCTCAAGCTCTCCTGCTCAGATGCCTACCTCAGGGAAGCTGGG
CTTCTTGTGGTCAGTGGTTTTTTAATCTTTGGGTGTTTTGTTTTCATTGTGGTGTCCTATG
TGCAGATCTTCAGGGCTGTCCTGAGGATCCCCTCTCAGCAGGGACGGCACAAAGCCTT
CTCCACGTGCCTCCCTCACCTGGCCGTGGTTTCCCTGTTTGTCAGCACTGGCATGTTTG
CCCACTTGAAGCCCTCCTCCATCTCCTCACCATTTCTAGACCTAGTGGTGTCAGTGCTTT
ACTCAGTAGTGCCTCCATCAATGAATCCCCTCATCTACAGCATGAGGAACAGCGAGCTC
AAGGAGGCCCTATGGAAACTGGCACAAAGGACACTGTTCCACCTACAGTAA
>Egret_OR6Y1 (Egret93)
ATGGGTGGGAGGAATGAAACCAGTGTCGTGTATTTCATTCTCCTAGGTTTTCCCACCAC
TGCTGAACTGCAGCTGCTCCTCTTCTCTGCTTTACTTCTCGCTTATTTATTTACTGTGTTG
GAAAACTTCCTTATCATTTTCATCATCCGAACCAACCACAGTCTGCAAACCCCCATGTA
TTTCTTCTTAGGAAACCTGTCTCTCTTAGAGATCTGTTATGTCTCTGTCATTGAGCCAAA
GATGCTCATAGGTTTCCTCTCTCAAGACAAACATATCTCATTCCAGGGGTGCATGACAC
AGCTGTATTTCTTCGTGACTTTTGTTTGTACTGAGTATATTTTGTTAGCTGTTATGGCCTA
TGACCGTTTCTTGGCCATATGCAAACCTCTCCGATATTCACTCATCATGAATCATCAGTT
CTGTGCTCAGCTGACAGCTGGCTGTTGGACTTGTGGTTTGATCACTTCTTCCATCAAGA
TGAGCTTTATAGCCCAGCTCTCATTCTGTGATGTAGACAAAATCAATCACTATTTCTGTG
ATATTTCACCCCTACTGAATATCTCCTGCGGTGATTCTTCTTTGGCTGAGCTAGTGGACT
TCATCTTAGCTCTGATCGTCATCATGCTGCCTCTGTGTACTGTGGTGACCTCCTATATTTG

CATCATATTCACTGTGTTGAAGATCGCTTCTTCTCAGAGGAGGCAAAAGGCCTTTTCCA
CCTGCAGCTCCCACCTGACTGTAGTGATACTCTTCTACTCTACCACTCTTTTCACTTATG
CCCACCCTAAGGTCATGTATACCTACAGTGCTAACAAGTTGGTATCAGTCTTGTACACA
GTAGTCGTGCCACTTCTGAATCCTCTCATATATTGTCTTAGAAACAAAGAAGTCAGGTT
TGCCCTGAGGAAGACCTTTGCTTGCACAAGACACACCTAA
>Egret_OR14J1 (Egret94)
ATGTCCAACAGCAGCTCCATCACCCAGTTCCTTCTCCTGGCATTCACAGACACACGGG
AGCTGCAGCTCTTGCACTTCGGGCTCTTCCTGGGCATCTACCTGGCTGCCCTCCTGGGA
AACGGCCTCATCATCACTGCCGTAGCCTGTGACCACCGCCTCCACACCCCCATGTACTT
CTTCCTCCTCAACCTCTCCCTCCTCGACCTGGGATCCATCTCTAACACTGTTCCCAAATC
CATGGCCAATTCCCTGGGGGACACCAGGGCCATTTCACACAAGGGATGTGTTGCCCAA
GTCTTTCTGTTTGTATTTTTGATTTCAACAGAGTATTACATTCTCACTGTCATGGCCTATG
ATCGCTACGTAGCCATATGCATCCCCCTACACTATTGGACTCTCCTGGGCAGCAGAGCT
TCTGTCCACATGGCAGCAGCTGCCTGGGGCAGTGGCTTTCTCAATGCCGTGCTGCACA
CTGCCAATACATTTTCACTCCCTCTCTGCCAAGGCAATGCTGTGGACCAGTTCTTCTGT
GAAATCCCCCAGATCCTCAAGCTCTCCTGCTCAGATGCCTACCTCAGGGAGCTTGGGC
TTATTATGTTTAGCGTCCTTGTTGTCTTTATGTGTTTTCTTTTCATTGTGCTGTCCTATGTG
CAGATCTTCAGGGCTGTGCTGAGGATCCCCTCTCAGCAGGGACGGCACAAAGCCTTTT
CCACGTGCCTCCCTCATTTGTCTGTAGTATCTCTGTTCCTCAGCACTGCAGTGTTTAACC
ACATCAAGCCTCCCTCCATTTCCTCCCCATCCGTTGATCTGGTGGTGGCAGTTCTGTACT
CAGTGGTGCCTCCAGTAGTGAACCCCCTCATCTACAGCATGAGGAACCAGGAGCTCAA
GGATGCCCTATGGAAACTGATCCAATGA
>Egret_OR6M1 (Egret95)
ATGAGACCAGAAAATGAAACTGCAGTTACTGCGTTCATCCTAGAGGGTTTCTCAGGGC
TTGATCAAAGACTACAGCTATTTGTCTCTGTGGTCCTTCTGCTCATGTACCTGACAACA
GTGATGGGGAACACAACAGTCATTTACCTCGTGTGTGTGGATCACCGCCTGCAAACCC
CCATGTACTTGTTCATCAGCAATCTGGCCTTCCTGGAAATCTGGTTTGCATCCTCCACA
AGCATCAAATTGTTTGTGATCCTGGGTTCTGGTAGGAGAACAATCTCACTAAGCAGCTG
TTTTGCCCAATCCTATTTCTATTTTGCCCTGGGCTGTACAGAGTTTGTTCTACTTGTTGTC
ATGTCCTTCGACCGCTATGTTGCCATCTGCCAGCCTTTGCGTTATGCTGCAATCATGAAG
CCACAGCTGTGTATCCACCTGGTTGTTGCTGCTTGGGTCATAGGCATCACACTCTTGAG
TTACCGTCTGATCCTCCTCTATAAGCTGACTTTCTGTGGCTCGAACAAGATCCACCATTT
TTTTTGTGACAGCTCCCCCTTATTCAAATTGTCCTGCTCTGACACCAGCCTGCTTTGGA
AAATAGACTCTATTTTATTTTCATTTGTCATACTGGGTTCCTTATGTTTAACGCTGGCATT
TTACATGGGCATCCTTTTCTGTATTCTACACCTGCCAGCAGCCTCTGAAAGGAAAAAAG
CTTTTACTACATGTTCTTCCCATCTCACCACCTTGGCCATTGCATATGGGAGCTGCATTG
CTCTCTATGTGTGTCCTTCCAAAGATGTTTCCTTGGAAACAAACAAAATTGTAGCTTTG
CTGAACACTGTCCTGTACCCATTCCTAAATCCATTCATCTACAGTCTTAGAAACAGAAC
TGTGATACTGGCCCTGAACGAAGCCATTGCCCATGCAACAACAGAGCTTTTCTCCTATT
TGTCATGCATTTCTGGACAGCTATTCCAATGA
>Egret_OR14J1 (Egret96)
ATGTCCAACACCAGCTCCATCAATGTGTTCCTTCTACTGGCTATTGCAGATACACGGGA
GCTGCAGCTCTTGCACTTCTGGCTCTTCCTGGGCATCTACCTGGCTGCCCTCCTGGGAA
ACAGCCTCATCATCACCACCGTAGCCTGTGAGCACCGCCTCCACACCCCCATGTACTTC

TTCCTCCTCAACCTCTCTCTCCTTGACCTGGGTTCAGTCTCCACCATTGTCCCCAAATCT
ATGGTGAATTCCCTGTGGGACAGCAGGGCCATCTCCTATGTAGGATGTGCTGCCCAAGT
CTTTCTGTTTTTCTTTTTGATGTCAGCAGAATTGTATCTTCTCACCATCATGGCCTATGAC
CGCTACGTTGCCATCTGCAAACCCCTGCACTATGGGACCCTCCTGGGCAGCAGAGCTT
GTGTCCACATGGCAGCAGCTGCCTGGGGTAGTGGGTTAATCTATGGTCTCCTTCACACT
GCAAATACGTTTTCCATACCTCTCTGCCAGGGTAACGCTGTGAACCAGTTCTTCTGTGA
GATCCCCCAGATCCTCAAGCTCTCCTGCTCAGATGCCTACCTCAAAGAGCTTTGGTTTC
TTGTTGTTAGTATCTTTGTGGGTTTTGGTTGTTTTGTTTTCATTGTGCTGTCCTATGTGCA
GATCTTCAGGGCCGTGCTGAGGATCCCCTCTCAGCAGGGACAGCGCAAAACCTTTTCC
ACCTGCCTCCCTCACCTGGCCGTGGTCTCGCTGTTTCTCAGTACTTCTATGTTTGCCCA
CTTGAAGTCCCCTTCTATCTCATCCCCATCCCTGGACTTGGTAGTGGCAGTGCTGTACTC
AGTGGTGCCTCCAGTAGTGAACCCCATCATCTACAGCATGAGGAACCGGGAGCTCAAA
GATGCGCTGAAGAATTTGATTCAACTGGTTGTCTCTAAGCAGCTGTAA
>Egret_OR2D2 (Egret97)
ATGGCCAGGGACAACCAAAGCATAGTGACAGAATTCATCTTTGAAGGCCTTTCCTCCC
AGCCAAGGACCCAGACCATTCTTTTCATAGTGTTCCTAGTTTTTATCTGTTCACAGTTG
CTGGGAACATCATGATCATCACAGTGATCAGAGCTGATTGCCAGTTGCAGTCACCCATG
TACTTTTTCCTTGCCAACCTGTCCTTCCTAGATATCGGCTACATCTCCAGCAACATCCCC
CAGATGCTGGTGAACCTCTGGACCAAGAAGAGGACCATCTCCTTCTCTGGATGTGCTG
CTCAGATGTATTTCTCTCTGGCTTTTGGCATGACAGAGTGTGTTCTGCTTGGGGTCATG
GCCTATGATCGATACATGGCAATATGTCACCCCTTGCTCTACACCACTGTCATGAACAA
GAAGTTCTGCATTCACATGGTCGTGGCTTCCTGGACCATCAGCCTGCTGAGCTCCATGG
TCATCAACAGCCTCACGTTGCGGCTGCCCTTCTGTGGGCCTGACATCTTGAACCATTAC
TTCTGTGAAGTGCCAGCCATGCTGGCTTTGGCCTACACTGACACTACCCTCGTGGTGTT
GGTCATCTTCATCTTCAGCATCCTCATAGCCTTCATCCCTTTTCTTCTGATCATCACCTCC
TACATCCATATCCTTTTCGCCATCTTGAAGATTCAGTCTGCACACGTGCAATCCAAGGC
CTTCTCCACCTGTGGATCCCACCTAATGGTGGTAAGCTTATTCTATGGGACGGCCATCTG
CAGGTACATGAATCCTAAGTCAAGGCCTCCACAGGACAGGGACAAAGTGGTTGCTGT
GTTTTACACCGTTGTAGCCCCAGTGATGAACCCCCTCATCTACAGCCTCAGGAACAAG
GACATGAAGCATGCCCTGGGGAGGGCAATGAATAGACCCAAATCCTTGTTTATTTAA
>Egret_OR11A1 (Egret98)
ATGACAAAGCAAGAGTGGAAAAACACAATGGCTGTTACAGAGTTCATCCTCCTGGGAT
TTGGGAATGGCCCTGAGTTGGATTGTCTTCTGTTCCTGATGTTTCTGTTAATCTATGTTG
TGACCATAACAGGAAACATCTGCATCATCGTGCTGATGGTGGCTAATCAGCACCTTCAC
ACCCCAATGTACTTCTTCCTGGGCAATTTGGCCTGCTTGGAAATCTGCTACAGCTCAAA
TATCTTGCCAAGGATGTTGCTTGGCTATCTGGGTGGTGACAGACGCATTTCAGTCAACG
GGTGCTTTACGAAGTACTATTTCTTTGGTTGCTTGGCAGCCGCAGAGTGCTATCTTCTG
GCAGCAATGTCCTACTATAGGTACCTCGTGATGTGCAAACCCTTGCACTACCTCTCCCAT
ATGAATGGCAAGCTCTGCTTCCAGCTGGGTGCCTCATCTTGGATAAGTGGCTTTCTGTC
TAATTCTATACTGACATTCCTCATCTCGAGTTTAGATTTCTGTGGGCCTAATGAAATTGA
ACATTTTTTCTGTGACTCATTCCCAATGATAAAACTTTCCTGCAGTGATGTTCACGTGGC
AGGACTTGCCACTTCTGTTGTGGCAGGTGTGTGCTCACTGCCTCCCTTTCTGTTTACCT
TCTCATCCTACCTCTACATCATCATCACAGTCATGAGAATTCCTTCTGCCACTGAAAGG
AAAAAAGCCCTTTCCACTTGCTCCTCACTTCTTATTGTGGTGATTCTTTTTTACTGGTCA

ATAATAACTGTCTACGTCCTTCCCCATCATGATACCCAAATATCCCCCAACAAAGTCTTC
TTGGTTTTTTATACCACTCTCACTCCCTTGGTCAATCCTCTCGTCTGCAGTCTGAGAAAC
AAAGAGGTCAAGGAAGCTCTGCGAAAACAGACATGTAAATTGCTGGCTTTCAGAGGA
CTGCTTCCCGTTAAATAA
>Egret_OR14J1 (Egret99)
ATGTCCAACAGCAGCTCCATCACCCAGTTCCTCCTCCTGGTCTTTATGGACACACAGGA
GCTGCAGCTCTTGCACTTCTGGCTCTTCCTGGCCATCTACCTGGCTGCCCTCCTGGGCA
ACACCCTCATCATCACCGCCATAGCCTGTAACCACCACCTCCGCACGCCCATGTACTTC
TTCCTCCTCAACCTCTCCTTCATTGACCTGGCCTCCATCTCCACCACTGTTCCCAAATCC
ATGGCCAATTCCCTCTGGCACACCAAGGCCATCTCCTATGCAGGTTGTGCTGCCCAGGT
GTTTCTGTTTGCCTTATTGGTTGCAGCAGAGTATTCTTTTCTCACTGTCATGGCCTATGA
CCGCTACATTGCCATCTGCAAACCCCTGCACTACGGGACCCTCCTGAGCAGCAGAGCT
TGTGTCCACATGGCAGCAGCTGCCTGGGGCAGTGGCTTTCTCAACTCTCTCCTGCACA
CGGCCAATACATTCTCCGTACCCCTCTGCAAGGGCAATGTTGTGGACCAGTTCTTCTGT
GAAATTCCCCAGATCCTCAAGCTCTCCTGCTCAGATGCCTACCTCAGGGAGGTTGGGC
TTCTTGTGGTCAGTCTCTTGGTAGCCCTTGGGTGTTTTATTTTCATCATGCTTTCCTATGT
GCAGATCTTCAGAGCTGTGCTGAGGATCCCCTCTCAGCAGGGACGGCACAAAGCCTTC
TCCACGTGCCTCCCTCACCTGGTCGTAGTCTCCATGTTTTTGATCACTGCCATGTTTACA
TACCTGAAGCCTCCCTCCATCTCCTCCCCATCCCTGGATCTGGTGGTGGCAGTGCTGTA
CTCAGTGGTGCCTCCAGCATTGAATCCCCTCATCTACAGCATGAGGAATCAAGAGCTTA
AGGATGCAGTGTGGAAACAGATAACCGGATGTTTTTCTGAATTAATAAAATACGGCATA
GTGGTTATAATATAA
>Egret_OR2A2 (Egret100)
ATGCAGAATGAAACGTCTGTCACACAATTCATCCTCCTGGGGTTCTCCAGCAACCCAG
CCCTGCGGCTCTGCCTCTTTGGCATTTTCTCTGTCCTCTACTCCATCACTCTGATGGGAA
ACGCACTTGTCTTTGTGCTTATCTGCCTGGACTATCGCCTCCACAGCCCCATGTACTTTT
TCCTCTGCCACCTCTCCATTGTGGACATCTGCTATGCCTCCAACAATGTCCCCCATATGC
TGAGGAACCTCCTTGGACAAGGCAGAACCATCTCCTTTGCTGGGTGTGGGACACAGAT
ACACCTTTATTTAATTTTTGCACTTACAGAGTGTGTGCTGCTGGCCGTGATGTCTTATGA
TCGCTACGTGGCAATCTGCCATCCCCTCCGCTATGCCCTTATCATGAATCGGAGGGTGT
GCCTCACCCTTGCTGCAGTTTCATGGGCTTTTGGGTTCCTATTTGGCACACTACAAGCC
TCTCTGGCTTTACATCTGCCTTTCTGTGGCCCCTGTGAGGTTGACCACTTCTTCTGTGA
AATCCTTGCTGTCTTAAATCTGGCCTGCACTGACACTACTGCCAATAAAGTCCTGATCT
TTGCTGTTTGTGTGTGCTTCCTCCTCTTCCCTTTAGCCTTAATCCTAATTTCCTACCTGTA
CATCCTGGCCACCATTCTGCGCATCCGCTCTGCAGCAGGATGGCACAAAATCTTCTCCA
CCTGTGGCTCCCACCTGACCGTGGTGGGTCTGTTTTATGGAAATGCCATCTTCATGTAC
ATGGTGCCCGGGAGCAGTAACTCATCTGGGAAGGAGAAAGTTCTTTCCCTTTTCTACA
GTCTTGTCAGCCCAAGTTTGAACCCATTGATTTACAGTCTGAGGAACAAGCAGGTGAA
GGAAGCCTTGCTGAAGCTTCAGAGAGGGAAAAGAGTTTTTCATTCCATGTAG
>Egret_OR14J1 (Egret101)
ATGGGGAGTGAGGAGCGGTTAAGGTCTGGGAGAAAGGCTTTGATTTTCCTCACAGAAA
TCTCCCTTAACTCTTTATTGTCTCTTCCTCTAAGAACAGGACCCCATGCCCAGAGGCAG
CAAGCATCCAACAGCAGCTCCATCACCCACTTCCTCCTCCTGGCCTTCATGGACACAC
GAGAGCTGCAACTCTTGCACTTCTGGCTCTTCCTGAGCATCTACCTGGCTGCCCTCCTG

GGCAATGTCCTCATCATCACCGCCATAGCCTGTGACCACTGCCTCCACACCCCCATGTA
CTTCTTCCTCCTCAACCTCTCCATCCTCGACCTGACCTCCATCACTACCACTATCCCTAA
ATCCATGGCCAATTTCCTCTGGGACACCAGGGCCATCTCCTATATAGGATGTACTGTCCA
GCTCTTTATGTTTACATTCTTGGTAGTAGCAGAGTATTGTCTTCTCACCATCATGGCCTAT
GATCGCTATGTTGCCATCTGCAAACCCCTGCACTACGGGACCCTCCTGGGACACAGGG
CTTGTGTCCACATGGCAGCAACTGCCTGGGGAAGTGCTTTTCTCTGTTCAGTCCTGCA
CACTGCCAATACATTTTCACTCCCCCTCTGCAAGGGCAATGCTGTGGACCAGTTCTTCT
GTGAAATCCCCCAGATCCTCAAGCTCTCCTGCTCACGCGCCTACCTCAGGGAAGTTGG
GCTTCTTGTATTCAGTGTCTGTTTAGCACTTGGTTGTTTTGTTTTCATAGTGGTGTCCTAT
GTGCAGATCTTCAGGGCCGTGCTGAGGATCCCCTCTGAGCAGGGACGGCAAAAAGCC
TTTTCCACGTGCATCCCCCACCTGGCCGTGGTCTCTCTGTTTCTCAGCACTGCCATATTT
TCCAATCTGAAGCCTCCCTCCGTCTCTTCTCCATCACTGGATTTTGCGGTGGCAGTGCT
GTACTCGGTGTTTCCTCCAGCTGTGAATCCCCTCATCTACAGCATGAGGAACAAGGAA
CTCATTAATTCCATGAGAAAACTCATGACTGTTTTTTTTCCAAAAGAAATAAACTGTATA
TCATCTTCTGGCATTACTCATAATGTGGCTTCTCACAGGAGGAATATGTGA
>Egret_OR14J1 (Egret102)
ATGTCCAACAGCAGCTCCATCACCCAGTTCCTCCTCCTCGCATTCTCAGACACACGGG
AGCTGCAGCTCTTGCACTTCTGGCTCTTCCTGGGCATCTACCTGGCTGCCCTCCTGGGC
AACGGCCTCATCATCACCACCATTGCGTGCGACCCCCGCCTCCACACCCCCATGTACTT
CTTCCTGCTCAACCTCTCCCTCCTCGACATGGGATCCATCTCCAACACGGTCCCCAAAT
CTGTGACCAATTCCCTATGGAACACCAGGGCCATCTCCTACTCTGGATGTGCTGCCCAA
GTCTTTCTGTTTTTCTTTTTGTTGTCAGCAGAATTGTATCTTCTCACCATCATGGCCTATG
ACCGCTACATTGCCATCTGCAAACCCCTGCACTACGGGACTCTCCTGGGCAGCAGAGC
TTGTGTCCACATGGCAGCAGCTGCCTGGGGCAGTGGCTTTCTCAATGCTGTGCTGCAC
ACGGCCAATACATTTTCACTCCCACTCTGCAAGGGCAAAGCTGTGGAGCAGTTCTTCT
GTGAAGTGCCCCAGATCCTCAAGCTCTCCTGCTCAGATGCTTCTCTCAGGGAGGTTTG
GCTTCTTGTGTTTGGTGTCTGTATTGCATTTGGATGTTTTATTTTCATTGTGCTGTCCTAT
GTGCAGATCTTCAGGGCTGTGCTGAGGATCCCCTCTCAGCAGGGACAGCACAAAGCCT
TTTCCACGTGCCTCCCTCACCTGGCCGTGGTCTCCCTGTATGTCAGCACTGGCATGTTT
GCCTACCTGAAGCCCCCCTCCATCTCCTCCCCATCTATAGATTTTCTGGTGGCTGTTCTG
TACTCAGTGTTGCCTCCAGCAGTTAACCCCCTCATCTACAGCATGAGGAACCAGGAGA
TCAAAGAGGCCCTATGGAGACTGTCACAATGGATGCTGTTTCACAGACAATGGTCTTC
CACCCATTCTCTGCACATTTCCCAGAGTTTACCTTAG
>Egret_OR14A16 (Egret103)
ATGTCCAACATCAGCTCCATCACCCAGTTCCTCCTCCTGGCATTTGCAGACACACGGGA
GCTGCAGCTCTTGCACTTCTGGCTCTTCCTGGGCATCTACCTGGCTGCTCTCCTGGGCA
ATGGCCTCATCATCATCACCGTCGGCGCCCCCCCCTCCACCCCCCTATGTACTTTTTCC
TTCTCAACCTCTCCCTCCTTGACCTGGGATCCATCTCCACCACTGTCCCAACATCCATG
GCCAATTCCCTCTGGGAGACCAGAACCATTTCCTACCCTGGATGTGCTGCCCAAGTCTT
TCTGCTTTTCTTTTTGATGTCAGCAGAGGTATATCTTCTCACTGTCATGGCCTATGACCG
CTACGTTGCCATTTGCAAACCCCTGCACTATGGGACCCTGCTGGGCAGCAGAACTTGT
GTCCACATGGCAGCAGCTGCCTGGGGCAGTGGCTTTCTCAATGCTGTCATGCACACGG
CCAATACTTTTTCCATATCCTTCTGCCATGGCAATGCCGTGGACCAGTTCTTCTGTGAAA
TCCCCCAGATACTCAAGCTCTCCTGCTCAGATGCTTATCTAAGGGAGGTTGGGCTTCTT

GTGTTTAGTGTCTGTATTACATTTGGGTGTTTTGTTTTTATTGTGGTGTCCTATGTGCAGA
TCTTCAGGGCTGTGCTGAGGATCCCCTCTCAGCAGGGACGGCACAAAGCCTTTTCCAC
GTGCCTCCCTCACCTGGCCGTGGTCTCCCTGTTTATCAGCACTGGCATGTTTGCCTACT
TGAAGCCCCCTTCCATCTCCTCCCCATTCCTGGATCTGGTGGTGGCAGTTCTGTACATG
GTGGTGCCTCCAGCAGTGAACCCTCTCATCTACAGCATGAGGAACAAGGAAATCAAGG
ATGACTTACGGAATCTGATCCAATGGGTGCTGTTTCATCATGCTGTAACAGCTTTCTTAC
AGACATAA
>Egret_OR14J1 (Egret104)
ATGTCCAACGGCAGCTCCATCACCCAGTTCCTCCTCCTGGCATTCGCAGACTCACGGG
AGCTGCAGCTCTTGCACTTTTGGCTCTTCCTGGGCATCTACCTGGCTGCCCTCCTGAGT
AATGCCCTCATCATCACTGCTGTAGCCTGTGACCATCGCCTCCGCACCCCTATGTACTTC
TTCCTCCTCAACCTCTCCCTCATCGACCTTGGCTCCATCTCTACCACTGTGCCCAAATCA
ATGGCCAATTCCCTCTGGGACACCAGGGCCATTTCCTACTGGGGATGTGCTGCACAGCT
CTTTTTCTTTCTGTTCTTGATCACTGCAGAGTTTTCTCTTCTCACCGTCATGGCCTATGA
CCGCTACGTGGCCATCTGCAAACCCCTTCACTACGGGACCCTCCTGAGCAGCAGAGCT
TGTGTCCACATGGCAGCAGCTGCCTGGGGCAGTGGGTTCCTTACTGCTTTGCTGCACA
CGGCCAATACATTTTCACTCCCTCTCTGCCAAGGCAATGCTGTGGACCAGTTCTTCTGT
GAAGTCCCCCAGATCCTCAAGCTCTCCTACCCAGACGCTTATCTCGAGGAGTTCGGGC
TTGTAGTGTTTAGTGTATTTGTGGTCTTTGGGTGTTTCATCTTCATTGTGCTGTCCTATGT
GCAGATCTTCAGGGCCGTGCTGAGGATCCCCTCTGAGCAGGGACGGCACAAAGCCTTT
TCCACATGCCTCCCTCACCTGGCCGTGGTCTCCCTATTTGTCAGCACTGGCATGTTGGC
CTACCTGAAGCCCCCCTCCATTTCCTCCCGATTTCAAGACCTAGTGGTGTCACTTCTGT
ACTCAGTGGTGCCTCCAGCAGTGAACCCTCTCATCTACAGTATGAGGAACCAAGAGCT
CAAGGAGACCCTATGGAAACTGGCACAATGGATGCTGTCTCAGCGACAATAA
>Egret_OR14J1 (Egret105)
ATGACTCTTCCTTTAAGAACAGGTCCCCATGCCCAGGGGCAGCAATTGTCCAACAGCA
GCTCTGTCATCCAGTTCCTCCTCCTGGCCTTCGTGGACACATGGGAGCTGCAGCTCTTG
CACTTCTGGCTCTTCCTGTGCATCTACCTATCCGCCCTCCTGGGCAACGGACTCATCATC
ATCACCATAGCATGTGACCACCGCCTCCACACACCCCTGTACTTCTTCCTCCTCAACCT
CTCCATCATCGACCTTGGATCCATCTCAACCACTGTTCCCAAAACCATGGCCAATTCCC
TCTGGCACACCAGAGAAATCTCTTATAGAGGATGTGCTCTCCAGCTCTTTCTGTTTACC
TTCTTGGTAGTAGCAGAGTTTTCTCTTCTCACCATCATGGCCTATGACCGGTACGTTGCC
ATCTGCCAACCCTTTCACTACGGGACCCTCCTGGGCAGCAGAGCTTGTGTCCATATGGC
AGCAGCTGCTTGGGGCTGTGGCTTCCTCAATGCTCTCCTGCACACTGTCACTACATTTT
CCATTCCCCTCTGCCAAGGCAATGCTGTGGATCAGTTCTTCTGTGAAATCCCCCAGATC
CTCAAGCTCTCCTGCTCACACTCCTACCTCAGGGAAGTTGGGCTTCTTGTGTTTGGTGT
CTGTATTGTATTCGGGTGTTTTGTTTTCATTGTGGTATCTTATGTGCAGATCTTCAGGGCC
ATGCTGAGGATCCCCTCTCAGCAGGGACGGCACAAAGCTTTTTCCACGTGCCTCCCTC
ACCTCACTGTGGTCTCCCTTTATGTCAGCACTGGCACATTTGCCTACCTGAAGCCCCCC
TCCATCTCTTCCCCATCCCTGGATCTGGTGGTGGCTGTTCTGTACTCGGTGGTGCCCCC
AACAGTGAACCCCCTCATCTACAGCATGAGGAACAGGGAGCTCAAGGATGCCCTGAG
GAAACGGATTCAATGGGTTCACCTTCGGCATCCGTAA
>Egret_OR14J1 (Egret106)
ATGTCCAACAGTAGCTCCATCACTGAGTTCCTCCTCCTGGCCTTCGTGGACACACGGG

AGCTGCAGCTCTTGCACTTCTGGCTCTTCCTGGGCATCTACCTGGCTGCCCTCCTGGGC
AATGGACTCATCATCACCGCCATAGCCTGCGACCCCCACCTCCACACCCCCATGTACTT
CTTCCTCCTCAACCTCTCCCTCATTGACCTGGCCTCCATCTCCACCACTGTCCCCAAAT
CCATGGGCAATTCCCTCTGGGACACCAGGACCATCTCCTATGCAGGATGTGCTGCCCAG
GTCTCTGTGTTTGTCTTGTTTGTCACAGCAGAGTATTCTCTTCTCACTGTCATGGCCTAT
GACCGCTACGTTGCCATCTGCAAACCCCTGCACTACGGGACCCTCCTGGGCAGCAGAG
CTTGTGTCCACATGGCAGCAGCTGCCTGGGGCAGTGGCTTTCTCAATGCTGTCCTGCAT
ACTGCCAATACATTTTCACTACCCGCCCTTGACCAGTTCTTCTGTGAACTTCCTGAGAT
CCTCAAGCTCTCCTGTTCAGATGCCTACCTCAGGGAAGTTGGACTTCTTGTTTTTGGT
TTTGTTCAGGTACTGGGTATTTATTTTCATCATGTTGTCCTATGTGCAGATCTTCAGGGC
CGTGCTGAGGATCCCCTCTCAGCAGGGACGGCACAAAGCCTTTTCCACGTGCCTCCCT
CACATGGCTGTGGTCTCTCTGTTTCTCAGCACTGCCATGTTTGCCTACCTGAAGCCTCC
CTCCATCTCCTCCCAGTCCCTGAATCTAGTGGTAGCAGTTCTGTACTCGGTGGTGCCTC
CAGTAGTGAACCCCCTCGTTTACAGCATGAGGAACCAGGAGCTCAAGGATTCCCTGAG
GAAACGGATTTTATGGACATTTTCCTGTAGTCATAAGGCTCCCATCACTCTCCACAAGT
GA
>Fulmar_OR5AR1 (Fulmar1)
ATGGTCAGAAGAAACAAGACAACTGTTGATGAGTTCATTCTCTTGGGAATCACAGATA
TTTGGGAGCTGCAGGTCATTCTTTTTGTGCTGTTTCTTCTGATCTGTGTCACCTCGTTGG
TGGGGAATCTCAGCATGATTGCATTAATCAGGCTTGACTCTCGACTCCACACCCCCATG
TACTTCTTCCTCTGCCACCTCTCCCTGGTAGACCTAGGTAATTCCTCAGCGGTTGCTCCC
AAAATGCTAGTGAGCTTCTTTGAAGAAAGAAAAGCCATCTCTCTGCTAGGGTGTGCAG
CGCAGATGTACTTTTGTGGAGTCTGCATAATCACCGAGTGTTACCTGCTGGCTGCGATG
GCCTATGACCGGTACGTGGCCATCTGTAACCCTCTGCTCTACATGGTCACCATGTCTCA
AAAGTTTTGTGTCCAACTGGCTGTGGGATCCTACATAATAGCTGCTGTGAGTCAAACAG
TGCTTGTCAGCTCAGTGTTCAGTTTACACTTCTGTGGCCCTAATGTCATCAATCACTTCT
TCTGTGACATTCCTCCGCTCCTGAAACTTTCCTGCTCCAGTACTACTGTCAACGAACAT
GTGCTTTTTACCATTGCTACTTCTATTGCATTCAGCACTTTAGCGTTCATTGTTGTCTCTT
ATAGTTATATCCTTACCACTGTCCTGAGGATCTGCTCCTCAGAGGGCAGGCACAAAGCT
TTCTCCACCTGTGCCTCACATTTGACATCAGTCTCAATTTTTTATGGGACTATGATCTTC
ATGTATCTCCGCCCCAGTTCTAGCTACTCCCTAGACCAGGACAAAGTGGTGTCTGTTGT
CTACACCATGGTGATTCCCATGCTGAACCCCCTGATCTACAGCCTGAGGAACATGGAG
GTGAAGGATGCTCTCAAGAGACTCCTAGAAAAAGTTCTTGTTTCCTTTAGAAATCAAA
CTGGTAAAGAGGTGTCATAG
>Fulmar_OR5R1 (Fulmar2)
ATGGTTGACGATAATTATACATTTGCATCTGAGTTTATTCTCCTGGGCTTCACAAACCGA
GAAGACCTGCAGGTGACATGTTTTGTCTTATTCCTTGTCATCTATGTGGTCACTCTAATA
GGAAATCTGGGAGTAATTCTATTAATCAGAGTCGATTCGTGCCTACACACCCCCATGTA
CTTCTTCCTAAGCCACTTGTCTCTGCTGGACGCCTGCTACTCCTCCACCATCATCCCCC
AAACCTTGCTGAATTTTTTAGTGGAGAAGAAGGTTATTTCCTTCGTTGGGTGTGCCACT
CAGCTCTTCTCCTTTGCGACTTGTGCCACCACCGAGTGCTACGTGCTGGCTGCCATGGC
TTATGATCGCTACATGGCCATTTGTAACCCCCTGCTCTACTCTGTGGTCATGTCCCAGAG
GCTTTGCGTTGGCATGTTGGCTGGTGCCTACTTAGCTGGTGTGGTCAGCTCCACCATAC
ACACGGTTTCCATATTTCGTCTCCCCTTCTGCCGGTCCAAGAGGATCAATCATTTTTTCT

GTGACGGACCACCGCTGCTAGCCCTCTCCTGCTCTGACACCCGTGTCAACGAGGCCAT
GGTTGGGGGGGTGGTGGGCTTCAATGTGCTAAGCACCACGGTCTTCATTCTAGTCTCCT
ACTTGTCGATCCTCTCCACCATCTTGCGGATGCGCTCCACGGCCGGTTGGCACAAAGC
CTTCTCCACTTGTGGCTCTCACTTGGTCTCCATCGCTTTGTACTACGGCAGCTCCTTCTT
CATGTACCTGCGCCCCGGCTCCAAACACTCCTTGGAGAATGACAAGGTGATCTCCGTG
CTGTACTCCGTTGCAGTCCCCATGCTGAACCCGCTCATCTACAGCCTGAGAAACACGG
ACATGAAGAACGCCATGAGGAAAGCAAAAGGTAGAGTCCTCTCCTCCTTGTCCATCCA
CGGTTCCTGGACAGCTGAAAGGAGAGGGCTATCCTTCCATGGTAAGGAGGGTTAG
>Fulmar_OR5AP2 (Fulmar3)
ATGGCAGAAGAGAATCACACTGTGGTAACTGAGTTTATCTTGTTGGGATTCACAGACA
ACCTGAAGTTGCAGGTCCTCCTCTTCATAGTGTTTCTACTGATCTACCTGTTGACTCTAG
TGGGAAATCTGGGGATGATTACGCTCATCCTGATTGACTCCAAGCTCCACACCCCCATG
TACTTCTTCATCAGCAGCCTTTCCTTCTTAGATGTCAACTACTCCACCATCATTATACCTA
GCACGCTCATGACTTTTGTGTCTGAGACAAAAGTCATATCCTACACAGCATGCGCAGCT
CAGCTCTTCCTATTCTGCATTGCAGTGACAGGAGAGTGCTGCCTCCTGGCCGTCATGGC
ATATGACCGGTTTATAGCCATCTGCAACCCCTTGCTCTACCCTGTCATTATGTCCAAGAG
GTTCTGCATGCTCCTTGTGTGTGGCTCCTACTTCATGAGCTGCATGAATGCAACTATTCA
GACATCATTTGTATTCCGCCTGTCCTTCTGCAATTCCAACATCATCAATCATTTCTTCTGT
GATATGCCTCCCATGCTGAAGCTCTCCTGCTCTGACACTTACGTTGCTGACCTGGTCCA
TTTCACCTGTGCTACTGTGCTTGTCACCTCAACCATGCTCCTCATTCTCATCTCTTACAT
CTGTATTGGGGTTACCATCCACAAGATTAAATCTGCCAAAGGCAGACGCAAAGCCTTCT
CCACCTGTGCTTCCCACCTGACTGCTGTCACCATGTTCTATGGGACAGGGTCCTTCATG
TACTTACGACCCAGTTCAAAATACTCTGTGGAGCAGGACAAGATCATCTCTGTGTTTTA
TACTCTGGCAATTCCCATGCTGAACCCTATGATTTATAGCCTGAGGAACAAGGAAGTCA
AAGAAGCCCTTCGAAGGACAGTAGCAAGGCTATATTACTCTCCGTGTGTGCCACAGAG
ATTCAGAAGTCCAAGCAGGACTGAGAGAACCTGA
>Fulmar_OR5AP2 (Fulmar4)
ATGGCTGGGGGAAACCACTCGAGTGTGACCGAGTTTGTCCTCTTGGGCTTCACCGACC
TGCAGGAGCTGCTCTTCGTGATTTTTTCACTCATCTACATCACCACGCTGGTGGAGAAC
CTGGGGATGATTGTCCTCATTAGGATCAACCCTCAGCTTCACGTGCCCATGTACTTCCT
CAGCCACCTCTCTTTCCTGGACGTCTGCTATTCTTCACCCATCATGCCGAAGCTCCTGT
CAGGTCTCCTTGCAGAGAGAAATGTCATTTCTTTCAAGGGCTGCATCACACAATTTTTC
TTCTTTGCAGTACTTGGCACCACGGAAGCCGTCCTTCTGACCATCATGGCATACGATCG
CTACGTGGCCATCCGTGAGCCCCTGCACTACTTGGCTGCCATGTCCCATGGGGTCTGTG
TCCAGCTGGTGGTGGGCTCCTATGCTGCCGGGAGCCTGAACGCCCTCGTGCACACCAG
CGCTCTCCTCCGACTCTCTTTCTGTGGCCCGAACCTTGTCAACCATTTCTACTGTGAAA
TCCCACCGCTCCTGCTACTCTCGTGCTCCGACACCTGGCTCAATGAGATGGTGATGGCC
GCGTGCATTGGTTTCATCATAACAACCTCAGTCTTGGCCATCGTTGCGTCCTACACCTGT
ATCCTCCTCACCATCTGGAGCATCTGCTCTGCAGAGGGCAGGCACAAAGCCTTCTCCA
CCTGCACCTCCCACCTCATGGCTGTCGTCCTCTTCTACGGCTCTGCGGCTTTCTTTTATT
TCCATCCCTTTTCCAGACACACAGAAAATCAAGGGAAAACAGCCTCCGTCTTCTACAC
CGTAGTGACCCCCATGCTCAACCCTTTCATCTACAGCCTGAGGAACGAGGAGGTGAGG
AGCACCCTCAGAAGAGCTACGAACCAGCTCCTCTCCTGCGTGTATTCCCACAGGTCCC
ACTCTAACCAAACCGAAGCAGATATCACCTGCCGCAGTGGAGTCAGCAGAAGAAACA

AGGCTGACACCTGA
>Fulmar_OR5AS1 (Fulmar5)
ATGGCTTGGGAAAACTGGACTTCAGTGACAGAATTTGTTTTCAAGGGCTTCACGGATC
GCCTTGATCTCCAGTTTACCCTCTTTGTGGTTTTCCTGCTCACCTATGTCATCACCGTGG
CGGAAAACCTCGGCATCATCGCTGTAGTCCAGCTCAATTCCCAGCTTCAAACCCCCATG
TACTTCTTTCTCAGCAACTTGTCCTTCTTGGACCTGTGCTACTCCTCAGTTGTGACACC
CAAAATGCTGCTGCACCTCTCATCGGAAAGGAAGACTATTTCTTTTGCTGGCTGCTTCA
CACAGCTGTTCTTCTACGCTGCTTTTGTAATCGTGGAGTGTTACCTCCTGGCCGCGATG
GCGTATGATCGCTACGTCGCCATCTGCTACCCGCTGCGCTACCCTGTCGTCATGTCCAA
GAAGCTCTGCGTTTTCCTCCTGGTTGGGTCCTACGGGGTTGGGTTTTTTAATTCGATGG
TGTTCACAGTGTTTGCGCTGAGGGTGTCCTTCTGCGGCCCCAACGTCATCGACCACTT
CTTCTGCGATGGGCCACCACTGATAAAACTGGCCTGCTCCGATACTCGCCTCAACCAA
CTGCTGCTACTTGCTTTTGGGGCCTTCAACGAGGTCACCACCGTATCAGTCGTCCTCAT
CTCCTACGGCTGCATCCTCTTCAGCATCCTGAGGATGGGCTCTGCGCCGGGCAAGCGC
AAAGCTTTTAGTACCTGTGCATCCCACCTGCTGGTCGTCACCATCTTCTACGGGACCCT
TCTCTTCATGTACCTGCGGCCCAGCTCCAGCTACTCCCTGGGCAGGGACAAAATCATTT
CTGTCTTCTATGCGGTGGTGACCCCCATGCTGAACCCCCTAATCTACTGCCTGAGGAAC
CAGGAGGTGAAGAGTGCTCTGAAGAGAGCAGTGGGGAGAATAATTATTTCCCTGCTAA
AGGCAGGGAAGTAG
>Fulmar_OR6B1 (Fulmar6)
ATGCCCTCAGATAACCTGACCCATGTGGTTGAATTCATTCTGGTTGGTTTTCCAGGTAA
ACAGGAAATCAAGCTGCTGCTCTTTTTTATGTTCTTCCTGGCTTACGTGCTGACAGTGA
CAGAAAATGCAATGATTGTTGTGCTTGTTTCGACAAATCTCCAGCTTCACAAGCCAATG
TATGTTTTCCTGGGCAATCTTTCCTTTCTGGAGATCTGGTATGTCTCTGTGACAGTGCCC
AAAGTGCTTGTGAGCTTGGTGACAAAGAGACAAGGCATCTCCTTCACAGGCTGCATG
GCTCAGCTATTCTTCTTCCTGGCATTGGCCTGCAGTGAGTGCACTCTCTTGGCTGTCAT
GGCCTACGATCGCTATGTGGCCATCTGCAACCCATTGCGTTACCCAATCATCATGGATCA
CACTCTTTGCACCCGTCTGGCCATTGGATCCTGGATAAGTGGCTTCCTGATTTCCACAG
GGAAGGTTTACTTCATTTCACGTCAGACCTACTGTGGGCCCAATATCATCAACCACTTC
TTTTGTGATGTCTCTCCCTTACTGAAGCTAGCCTGCACCGACATGTCAGCAGCTGAGCT
TATGGATTTCTTACTGGCCCTGCTCATCCTTCTTGTACCACTCATTGTGATTATGGTCTCC
TACGTGTGCATCATCTCTGCTGTCTTGGGCATTCCCTCAGCCCAGGGGCGTCACAAGGC
CTTCTCTACCTGTGCCTCTCACCTTTTAGTGGTCACGGTGTTCTATACAGCCTCCCTGTT
TATCTATGCCAGGCCCCAGGCTATTGATTCCTTCAGCTCCTACAAACTGGTTTCTATGGT
ATACACTGTCCTGACACCCCTCGTCAACCCGGTCATCTATTGCCTAAGGAACCAGGAAT
TCAAAAGTGCTCTTAGGAAAACAATATACTGGAGAGACATCTTGTCCTAG
>Fulmar_OR52B2 (Fulmar7)
ATGCCACCATTCAACCTTACCAGCTTGACGCCAGCAACATTCATCCTGGCTGGCATCCC
AGGCATGGAGAAGTTGCACATCTGGATCTCAATCCCATTCTGCTTCATGTATCTGGTGG
CCCTGCTAGGAAACGGTGCCGTGTTGTTTGTCATAAGGACAGAAGGTAGCCTCCACGA
GCCCATGTACTTCTTCCTGTCCATGCTGGCCATTGCTGACCTGATACTGTCGACCACAA
CTCTGCCCAAGATGCTGGCTCTGTTCTGGTTCAACGCCGGGGAGATTTCCTTTGGTGCC
TGCCTGACCCAGATGTTCTTCCTGCATTTCAGCAGCTCGGCAGAGTCGATGATCCTGCT
GGCCATGGCGTTTGATCGGTTTGTTGCCATCTGTTATCCCCTGCGGTACACGGCAGTGC

TGACCCAGTCAGTCGTTGTCAAAACCGGGCTGGTGATTTTGCTGAGAAGTTTCTGCAT
CATTTTCCCATGTATATTTCTTTTGAAAAGGCTGCCGTTCTGCGGGCACAATGTCATCCC
GCACACCTACTGCGAACACATGGGCATCGCCCGGCTGGCCTGTGCCGACATCTCCATC
AATATCTTGTATGGCCTGGCAGTGCCTGTTGCAACAGCAGTGGTAGATGTTGTACTCAT
TGCTGTCTCCTATGTCTTAATTCTTCTGGCATTATTCAGACTCCCTTCCAGGACTGCCCG
TCACAAGGCTTTCAACACCTGTGGCTCTCATGTCTGTGTTATATTACTTTTCTATATCCCT
GTTTTTTTCACTGTTTTAACGCACCGCTTTGGTCGGGAAATCCCCCAGCACATCCACAT
TCTGCTGGCCAACCTGTACATGGTCTTCCCCCCGATGCTGAACCCCATCGTGTATGGTG
TGCGGACACAACAGATAAGAGAGAAGGTTGTGAAAGTGTTCATCTGCCCCAAGAGTC
ATTTGCTGCAATAA
>Fulmar_OR5V1 (Fulmar8)
ATGGCAGGTGAGAACCAGACTCATACAATGGAATTCGTGCTCCTGGGCTTTTCCCATGG
CCAGCCCTTCCTCTTTGTTCTTTTCCTGGCCATTTACCTTGCCACACTGCTGGGGAACT
CTGCAATACTCGCCCTCGTGTCCCTGGATCCCCATCTCCACAGCCCCATGTACTTCTTCC
TCAGTCACCTGTCCTGCCTGGACATTTGCTATTCATCAGTGACGGTGCCCAAGATCCTG
GCAAACGCCCTGCGCCCGCAGGCGACCATCTCCTACCACGGGTGCCTGGCACAGATGT
TCTTCCTGATGGGGTGCGCGGGGGCTGAGTGCGCACTCCTGGCTGTCATGGCCTACGA
CCGCTACGCAGCCATATGCCAGCCCCTGCGCTACACCCATGCCATGAGCCGGGGTGTCT
GTGTGGTGGCAGCCGCCAGCTGCTGGCTCTGGGGGATGCTGGACTCAGCCGTGCACA
CCCTCCTGGCCTCCAGGCTCTCCTTCTGCGGGGCTGCCCAGCTCCAGCATATCTTCTGT
GATGTCCCCCCACTGCTGCGGGCGGCGTGCAGCAACACCCGCCCCAGCGAAGTGGCA
CTCCACGCTGCCAGTGTCTTTGTGGGCCTCAGCCCCTTCATGCTTGTCGTCGTCTCCTA
CCTCCGCATCCTGGCCACTGTCCTCGGGATGCCCGTGGCCACCGGCCGGCACAAGGCC
TTCTCCACATGCTCTGCCCACCTGCTCGTGGTCGCCCTGTACTTTGTGACGGCCAACCT
GAACTACAACCGGCCCAGCTCCGGCTACTCCCCGGCAGCCGACACACTGGTGTCCGC
ACTGTACTGCATCGTCACCCCCATGCTGAACCCCCTCATCTACAGCCTCCGCAACCAGG
AGGTGCGGGGTGCCCTGCGGAAGGCTGTGCGGGGACGGGGCACGCTGGGCTCCCCGG
GTAGCAATGCATGA
>Fulmar_OR4S2 (Fulmar9)
ATGGTGTCTATGGATAACAAAAACAACGTGATGGAGTTCATCCTCCATGGACTGACAC
AAAATAAGACAGTAGCAAAAGTGGGCTTCTCGTTATTCTTAGTCTTCTATGCCACTAGC
ATTCTTGGAAACCTGCTTATCATCATTACTATAAAGACAAGTGAACAGCTGAACTCTCC
CATGTACTTCTTCCTGAGCTACTTGTCATTTATAGACATCAGTTACTCTACTGTCACATCT
CCCAAACTCATTTATGACCTTCTGGTTGAGAAGACGACCATCTCTTTTGTGGGCTGCAT
AGCTCAACTCTTTGCGGGCCATTTCTTTGGGTGCACTGAGATCTTCCTTCTCACAGTGA
TGGCCTATGATTGTTGCATTGCTATATGCAAGCCGCTTCATTACACAAATATTGTGAACA
AGCATGTCTGTGGCTGGCTGGTGGCAGCTTCATGGATAGGAGGCTTTGTACACTCAGT
GGTGCAGACCCTGCTGGCCATTCAGCTACCGTTTTGTGGGCCCAATGAGATTGACCACT
ATTTTTGTGATGTTCACCCTTTACTGAAGCTGGCCTGCACTGACACTTATATCATTGGTG
TCACTGTGGCTGCCAATAGTGGTGTGATTTCCCCGAGCTGTTTTGTTGTTCTTGTTGTGT
CCTATGCTGTTATCTTGGTTTCTTTGAGGACATGCTCTTCTGAAGGGTGTCTCAAAGCA
CTCTATACATGTGCTTCCCACATCACTGTTGTAGTTCTGTTCTTTGGGCTGTGCATTTTCA
TCTATATGCGCCCTTCCACCACCTGCTCAGCAGACAAGATGGTCTCTGTGTTCTACACC
ATCATCACACCTATGCTCAATCCCTTGATCTATACCCTCCGAAATGAAGAGGTGAAAAA

TGCCATGAAAAAGTTGTGGAGCAGGAAAGTGAAGAGGAGGGAGAAATGA
>Fulmar_OR51G2 (Fulmar10)
ATGACCAACAGTAGCTTCCTTAGATCTTCTACCTTCCTTCTAACAGGAATCCCAGGAAT
GGAAAGTGGGAACGTGTGGCTTGCCATCCCTTTCTGCTGCGTGTATGTTATTTCCATCC
TGGGAAATAGTGCAATCCTCTTTGTCATCAAAGCAGAGCGCAGCCTCCATGAGCCCGT
GTACCTCTTCCTGTGCATGCTGGCTATCGCAGAGCTTGATGTGTCTCTGTCTATGCTCCC
CACAGTGCTGAGCATGCTGCTCTGTGATTCACAGATCAGGTTTCACACCTGCCTCGCCC
AGATGTTCTTCATTTGCTCCTTCTCCATCCCGGACTCTGGGGTGTTGAGGGCCATGGCT
TTTGACCTCTTCATGGCCATCTTGCAGTATGCACCCATCCTGACCAACCCCAGGATAGG
TGTCATTGGGCTGGGGTTCACAGTGAGGAGCATTAGCCTCTTGCTCCCCTTGCCAATAC
TTCTGAAGAAGCTGTCGTTCTGCAGGTCCCAGGTGCTGGCTCACTCCTTCTGTTTGCA
CCCCAACTTACTCCAGCTGCCCTGTGCAGATATCAAGGTGAATAGCATGTATGGCTGCT
TTGTCATCCTGGCCACCTTTGGGCTAGACTTGTCTATCCTCCTGTCCTGTGCCATGATCA
TTAAGTCTGTGCTGAGTATCACATCCAAGGAAGAGTGTCTCAAGGCCCTAAACACCTG
CCCTAAACATCTCTCATATCTGTGCTGTTTTGATTTATTATATTCCAATGATTGGCTTGTC
CATGGCATACAGGTTTGGGAAACCTGCCTCTCCTCTGATTCATGTCCTCATGGCCAATAT
CTACTTCCTTGTACCCCCTGTGCTAAATCCCATTATTTACAGTGTAAAAACTAA
>Fulmar_OR52B2 (Fulmar11)
ATGTATGAGCTCAACGAAAGCAGCTTCGATCCTATCACCTTCGTCCTGATGGGCATCCC
GGGCATGGAGGAGTCCCACATCTGGATCTCCGTCCCCTTCTGTCTGATGTACATCACTG
CGGTGTTTGGCAACTCTGTCATCCTCTTCGTCATCATCACGGACAGGAGCCTCCATGAG
CCCATGTACCTCTTCCTTGCTATGCTGGCTGTTTCTGACCTCATGCTTTCGACCACGACG
GTGCCCAAAATGCTGGCGATCTTCTGGTTCAGTGCCAGGGAAATTTCCTTCGATGCCTG
CATTACACAGATGTTCTTCACCCATTTCAGCTTCATCGTGGAATCGTCCGTTCTGCTGGC
GATGGCGTTCGACCGGTACGTGGCCGTCTGCGACCCGCTGCGGTACTCCTCAACCTTA
ACTCCCTCGGTGATCGGGAAAATAGCAGTGACTGCCGTTGTCCGGGGGTTCTGCATCA
TGTTCCCCGCCATCTTCCTCCGGAAGCGGCTGCCGTACTGTGGACACAACGTCATGCC
CCACACCTACTGTGAGCACATGGGCATCGCCCGCCTGGCCTGCGCCGACATAAAAGCC
AACGTCTGGTACGGGCTGACAACGGCTCTTCTCTCCTCCGGCCTGGATGTCGTGCTCAT
CGCTGTCTCTTACGCTCTGATCCTCAGGACAGTCTTTCGGCTCCCGTCCCCGGAGGCCC
GTCTCAAAACCCTCAGCACCTGCGGCTCCCACCTCTGCGTGATCCTCATGTTCTACGTG
CCCGCCTTTTTCTCCTTTCTCACGCATTGGTTTGGCCACCACATCCCAAGTCACATTCA
CATCCTCCTGGCCAACCTCTACGTCGTCGTCCCGCCGATGCTCAACCCCATCGTGTACG
GGGTGAGGACAAGGCAGATCCGGGAGCGCGTCGTCCGCCTCTTCTGCCCCGCGGGGG
AGTGCCCCTGCCCCGGCGGGGGGAGCCGGTGCTGA
>Fulmar_OR52B2 (Fulmar12)
ATGGCGGCTCTCAACGACACCAGCTTGCAGCCTGCCTTCTTCCTTCTGCTGGGCATAGC
AGGCCTGGAGGACCTGCACATCTGGCTCTCCATCCCATTCTGCCTGATGTACATCGTGG
CGCTTCTTGGCAACTTCATCCTCTTATTTGTCATTGTGATGGAGCGAAGCCTCCACAAG
CCAATGTACCTCTTCCTGGCCATGTTAGCGGTGGCAGATCTCATATTATCCTCCTCCACA
GTGCCCAAAGCCCTGAGCATATTCTGGTCCCTTTCCAAGGAGATGCCCTTCCATGCCTG
CCTTACCCAGATGTTCTTCACACACGTGAGCTTCATTGCAGAGTCGACCATTCTGCTGG
CCATGGCGTTCGACCGGTACGTGGCCATCTGCAACCCCCTGCGATATGCCACGGTGTTC
ACGCACTCGGTGATAGCCAAGATAGGGCTGGCTGCAATAGCCAGGAGCTTTTGTGTGA

TGTTCCCAACAATATTCCTCCTTAAGAGGCTGCCATACTGCAGACACAACATCATGCCG
CACACCTACTGCGAGCACATGGGCATTGCCCGGCTGGCCTGTGCCGACATCTCCATTAA
CATCTGGTATGGCTTTGCCACCACCCTTCTGTCCCCAGGCGTGGACATTGTGCTCATCG
GGGTATCGTACATCCTCATTCTCCGGGCTGTCTTCAGGCTCTCATCCAAGGATGCCCAG
CTCAAGGCAGTTGGCACCTGCAGCTCTCATGCCTGCGTTATATTAATGTTCTACACACC
AGCATTTTTCTCATTTTTCACTCATCGGTTTGGCCGCAACGTCCCACACCATGTTCACAT
CCTGTTGGCCAATCTCTATGTGCTCCTGCCGCCCATGCTAAACCCCATCGTCTACACTTT
GAAAAACAAACTCATTCGAGAAAAGGTGTCCCAAGTACTCTTCAGGACTGGGCAAGT
GCGGTGA
>Fulmar_OR6C75 (Fulmar13)
ATGGAAAACCGAACCATGGTCAAGAATTTCATCCTTGTGGGTTTCACAGCTGGCAGGA
AGTTGCAGATCTTTATGTTTGTGGTTCTCCTTGTCATCTACATCTCAATTATAATTGGAAA
CATAGTGATTGTCACCATCAGCATCGTGGACCGTGGGCTCCGTGTGCCCATGTATTACTT
TATTTGGAACTTTTCACTCTTGGAAATTGGTTTCACTTCTTCTGTCATTCCTAAGGTCTT
GTTCAACCTCGCATCAGGAGAAAACACAATTTCTGTGATTGGTTGCTTTGTTCAACTTT
TTTTATATTTCATCTTTGGCACGACAGAGTTTCTTCTCTTGGCAGCAATGTCATTTGACA
GGTATGTAGCCATCTGCTACCCCTTGTCTTACTCCACCATAATGAGCAATGGGCTTTGTA
GCTTACTAGTGCTTGGTTCATGGGGGCTGAGTTTTGCACTTCTGATTGGCCCATCAATT
GTTGTCTTCCAGTTACCCTTTTGTAACCAAAACATTAACCATTTCTTCTGTGACAGTGC
ACCTTTAATAAAACTTTCCTGCACAGAAACACCTGTTCTAGATCTTGTTAACTTCTCCAT
AGCTGTTTTTCTCTCCTGGGCACTCTTATTATCACAGTCCTGTCTTATGCCAATATAATT
TCCACTATATTGCACATTTCATCTGCTTCAGGGAAGCAGAAAGCCTTTTCCACTTGTGC
ATCTCACCTTATTCTTGTCTTCATCTCTTATGGGAGTTGCATTTTCATGTATGTTAAACCC
ACACACATGGGTGGGCTTGACATCAGCAAAGGGGTAGCTATTCTCAACACAGTGGTAT
CTCCTCTGCTCAGCCCATTCATCTTCAGTCTGAGGAACAGGCAGGTTCGAGAAGCCTT
GCAAAAATATTTCTCCATGTGCAGTTCTTACAGAACAATTCTGAGTCTGATGAGAAGTG
AAAAGTAA
>Fulmar_OR51G2 (Fulmar14)
ATGGAGCATGACTCACATACCACGTGGGAATTCAATGGCTCCTTCTATCAGCCTTCAGC
TTTCCTCATGATGGGCATCCCAGGCCTGGAAGCCCTTCACCACTGGATCTCCATCCCCT
TCTGTGCACTGTACCTTATTGCTCTCTTGGGAAACTGCATGATTCTATTCATCATAAAGA
AGACCCAAAGTCTTCACGAACCAATGTACTACTTCCTCTCCATGCTGGCAGTCACTGA
CCTGGGCTTGGTTCTGTGTACACTGCCTACTACTCTGGGCATTTTTTGGTTTAATATGCG
AAGGATTGGGTTTGATGCGTGCCTCACTCAGATGTATTTCATCCACATACTGTCCTTCAT
TGAATCCTCTGTGCTCCTGGCAATGGCATTTGACCGCTTCATTGCTATCTCCCATCCACT
GAGACACCCATCCATACTGACCAAGATGACTGTCATGAAAATAGGTCTGGCAATTATAT
TGAGAGGTATGGTCTCCCTCCTTCCCATACCCTTCTTGCTCAAGAGACTGACCTATTGT
GGAAAGACTGAGCTTTCTCATTCTTTTTGCTTCCATCCTGATATCATGAACCTAGCATGT
GCAGATACAAAAGTCAATGACTTCTACGGTATGATTATTCTTTTATCAACGGTGGGGATG
GACTTCATCTTCATTGTGCTGTCTTACATCCTGATCATTAAAACTGTTATCAGCCTTGCA
ACCAAGGAGGAGTGTCTCAAGGCTTTGAATACATGCGTCTCCCACATCTGTGCTGTTCT
AGTGTTCTTCATCCCAATGATCGGACTGTCCATGATCCATCGCTTTGGAAAGAATGCTC
CTCCTCTGGTTAACACTTTGGTGGCCTACACCTACCTTATAATTCCCCCCGCTCTCAACC
CCATTATCTACAGCATAAAATCCAGCCACATCCGTGAGGCTTTGCTCAGGGCACTGCGG

AGGAAGAGTGAATCTGACTGGTAG
>Fulmar_OR10A7 (Fulmar15)
ATGAGCCCCAAATCCCTGCAGCGGGGAAATCTCAGCAGCCCCACCATGTTCCTTCTCTT
GGGATTTTCCAATGCCTACAGAGCACAGGTGACCCTCTGCCTGTGCTTCTCGCTCATTT
ATCTGGTGACGGTTGTGGGGAACCTGCTCATCGTGACCCTCGTCTGGCTGGATGCCCA
CCTGCACTCCCCCATGTATTTCTTCCTGGGCCACCTCTCCTTCCTGGACATCTGCTACTC
CTCTGTCACCCTCCCTAAGATCCTTGGAGACTCCTTCTCACCACAGAAGACCATCTCCT
TTGTGGGCTGCATCACACAGATCTATTTCTTCCTTTGCTTTGGGGGCTCCGAGTGCGTG
CTTCTGGCTGCCATGGCCTACGATCGGTACCTGGCCATCTGCCACCCCCTCCACTACCC
GGTACTCATGAGCAAGAAGATGTGCCACTGTTTAGTGGTTGTTTCGTGGCTGAGCGGC
TCCTCCTCGTCTCTGATCCAGGCCTTCCTCACAGCCCGCTTGCCCTTCTGCGGGTCCAA
CATGATCAACCACTTATTCTGCGAGCTGCCCTTCTTGCTGAAAGCATCCTGCAGCCCTA
ATGCCCCCCTCAACAAGGCCGCCTTGTATGCTTCGGCTGGGACTATTGCGATGGGCTCT
TTCCTCCTTACCCTCATGTCGTACGTTCACATCATCAGGGCCATCCTCCAGAAGGGAGC
AGGGACGCAGAGGGCCTTTGCCACCTGCACCTCCCACCTGACCGTGGTGTCCCTGTTC
TTCGGTGCCGGGGCCATTGCATACCTGGTGCCTCACTCCAGCGGCTCCAAGGAAATGG
ACGAGGGCCTCGCGCTGCTGTACGCTGTTGTGACCCCCATGCTCAACCCCATCATCTAT
AGCTTGAGAAACAACGAGGTCAAAGGGGCCATCAGGAAAGCCCTGCGCAGGGGGGT
GTTACAGATGTCCACCAAGGGTGCAGGCGTCGCCGCTGAGCAACCCCCACCCTCCCCA
TGGCAGGGATGTGGCTCTCGGTGA
>Fulmar_OR5V1 (Fulmar16)
ATGAAAGATAAGATGCAGAGGGTCAACCTATCAACGGTTTCTGAATTTCTTCTTGTAGG
CCTTTCTGATGCTCCAGAAGTCCGTTTTCTTCTCTTTGTGCTGTTTTTGATCATTTATTTG
ACCACCATGGCAGGCAACATCACAATCCTTGTTGCCATTGGCACAGACACTTGTCTGC
AGAACCCCATGTACTTCTTCCTTGGCAACTTATCCTTACTGGATATCTTATGTCCCACTAT
CACTGCGCTGAAGATGCTGGAGGCCTTGTTGCTTGAGAACAAAGTGATTTCATTCACG
GGCTGCATACTGCAGCTGTTCTTCCTCATTGATGTTGTAGGCACAGAGATTTTTCTCTTG
GCTGTGATGGTGTATGACTGTTACGTTGCAAAATGTCATCCGCTGCAGTACATGAATATT
GTGAGTATGAAACTGTGTGCTCACCTAGCTATTGGCACCTGGGTAGTAGGATTTTTTAA
TTCTCTGTTGCACACATCTTTGATTTTTACACTCTTCTTTTGTGATTCTAATGAAGTTGA
CCAACATTACTGTGATATTCCTCCTATGCTGGCCCTCTCCTGCTCACCTACTTACAGTAG
GGAACTGGTAATTCTCACAGTTGCTGGGGTCCTTGGAAGCAGTGCCTTTGTGGTCACT
CTGATCTCATATATCTATATCCTCATGGCTATCCTGTGCATGAACTCTTCAGAGAGCAGG
CACAAAGCTTTCTCCACTTGTGGTTCTCACTTGACATTAGTATGCCTTTTCTACGGGAC
CACCACTTGCACGTATGTACGGCCTTCCTCCACCTACTCACCTAATCAGGATAGGATAAT
TTCTATGCTCTATGGAATCCTCACTCCCCTGCTAAACCCCATAATCTACAGTCTGAGGAA
CAAAGAAGGTAAATGTGCCCTAAGAAGAGTAATCAGCCAGGTAAGAACTGCTTTAACA
AGACAAGAACATCTCTCTCAGTCTCTGGTGTCCTCTGGAGCCGTTGCAATTGGATAG
>Fulmar_OR5B21 (Fulmar17)
ATGGAGAGAGGGGAATGGGACAACCAGACATTGCTGATGGAGTTCCTCTTGCTGGGA
CTGGGGGATGCCCGTGAGCTCCAGACACCTCTCTTTCTCCTCTGTCTGACCATATACAC
AGTGACCATGGTTGGGAACATCCTCATCGTTGTGCTCGTGGTCACAGACCCGCATCTCC
ACACACCCATGTACTTCTTCCTGGTGAATCTGTCTAGCCTAGAGACCTGCTACAGCTCC
ACCATCCTGCCCAGGCTGCTAGCCAGCTTCCTGACTGGAGACAGGACCATCTCTGTGC

AGGGATGTATGGCACAGTTTTCTTCTTTGGCACTTTTGCAACTTCTGAGTGTTACTTG
CTGGCTGCCATGTCCTATGATCGGTATGTGGCCATATGTCAACCCCTGCTTTATGCAAGC
CTCATGAACTGGAAAGTATGTCTCCAGCTGGTGGCTGGATCATGGGTTGTGGGACTGCT
AATTTCTACAGGAATCACATCTTTCATCTCTCGCCAAAGGTTCTGTGGCCCCAGTGCAA
TTGACCACTTCTTCTGTGAAGAAGCTCCATTGCTGGAACTCTCCTGCAGTGACACTGA
GATGATCAGAATTCTTATTATCGTACTGTCTTTCCCAGATGTAGTTTTCCCATTTCTGTTC
ACTCTGGCATCCTATGTCTGCATCATAGCTGCCATCCTGAGGATCCCATCCAGCCTGGG
GAAGCACAAGGCCTTTTCCACCTGCTCCTCTCACCTCACTATGGTCATTGTTTTCTATG
GGACCCTCATCATTGTCTACATGCTGCCCAGAACAGCGGCACTGAGACAGCTCAACAA
AACATTCTCTTTTTTTTACACTGTCCTCACACCTCTCATCAATCCACTCATCTACAGTCT
GAGGAACAGAGAGGTCAAGGGGGCACTGGGGAGGGTGCTAAGGAGGGCTGCTGCCT
GCACTGACAGCTCCAACTAG
>Fulmar_OR2A2 (Fulmar18)
ATGCAGAATGAAACATCTGTCACAGAGTTCATCCTCCTGGGCTTCTCCAGCAACCCAA
CCCTGCAGCTCTGCCTCTTCGGCATTTTCTCTGTCCTCTACTCTGCCACTCTGATGGGA
AACGCACTTGTCTTTGTGCTTATCTGCCTGGACTACCGCCTCCACAGCCCCATGTACTT
CTTCCTCTGCCACCTCTCCATCATGGACATCTGCTATGCCTCTAACAATGTCCCCCATAT
GCTGAGGAACCTCCTTGGACAAGGCAGAACCATCTCCTTTGCTGGGTGTGGGACACA
GATACATCTTTATTTAATCCTTGCACTTACAGAGTGCGTGCTGCTGGCCGTGATGTCTTA
TGATCGCTACGTGGCAATCTGCCATCCCCTCCGCTACGTCCTTATCATGAGCTGGAGGG
TGTGCCTCACCCTTGCCGCAGTTTCATGGGCTTTGGGGTTCCTATTTGGTACACTACAA
GCCTCTCTGGCTTTACACCTACCTTTTTGCGGCCCCTGTGAGGTTGATCACTTCTTCTGT
GAAATCCTGGCTGTCTTAAAGCTGGCCTGCGCTGACACTACCGCCAATAAAGTCCTGAT
CTTTGCTGTTTGCGTGTGCTTCCTCCTCTTCCCTTTAGCCTTAATACTAATTTCCTACCTG
CATATCCTGGCCACCATTCTGCGCATCCGTTCTGCAGCAGGATGGCACAAAACCTTCTC
CACCTGTGGCTCCCACCTGACCGTGGTGTGTCTGTTTTATGGAAACGCCATCTTCATGT
ACATGGGGCCCGGGAGCGGTAACTCATCTGGGAGGGAGAAAGTTCTTTCCCTTTTCTA
CAGTCTCATCAGCCCAAGTTTGAACCCCCTGATTTACAGTCTGAGGAACAAGCAGGTG
AAGGAAGCCTTGCTGAAGCTTCAGAGAAGGAAAAGAGTTTTTCATTCCGTTTCATTCC
GTGGGTCTGTTTTATGGAAACGCCATCTTCATGTACATGGGGCCCAGGAGCAGTAA
>Fulmar_OR6C4 (Fulmar19)
ATGGGCCTTTTTTTTTCTTTTTCTGCTAGCAATGAAGCCTCAATGAACCACACAACAGT
GGTGGAATTTGTCCTCTTGGGGCTGACCAATAGCCGCCACTTGGAGATCATCCTCTTTC
TGTTTCTTGTGATTGCCTACTTCTTGATCCTGCTTGGAAACATTACTGTCATCAGCATCA
CTCTTGTGAATCATTTCCTTCAGACCCCAATGTACTACTTCCTCAGAAATTTTGCCCTTT
TGGAAATCACTTTCACCTCCACATTCATTCCCAGCACCCTCTACAGCCTTCTGACAGAG
AGAAAGGTGATTTCCCTGCCTGGTTGTTTCCTTCAGATGCTGCTTTTCTTTTACCTGGGT
ACCTGCACATTTTTCCATGTGGCAACAATGTCCTTTGATCGGTATGTTGCCATTTGCCGC
CCTTTGCATTACACAACAATTATGAACAACAGATTTTGCCTCCAGCTGGTCCTGGCTTG
CTGGGCAGTGAGTTTTCTCTTGATGTTTCCTCCCACCATTATGATTGTCCAGTTGCCATT
TTGTGGTCCCAATGTCATGAACCACTTTTACTGTGATACTTCCCTGTTGTTGCAACTGTC
CTGCACAGACACAGGGTTCATCGAAGGACTGATGCTTATCATACTAATTATCATCATACC
TGGTACCTTAATAGTAACTGCTGTGTCTTATGGCTGCATTATTATCACCATCTTGAATATA
CCATCTTCAACAGGTAGGAAGAAGGCATTTTCCACTTGCTCAGCTCACCTCATGGTGGT

GATGATATTTTACAGCACATGTATTTACAGGTATATCCGCCCAGCACAGCGAGGTGGGC
AGGACTCTGACAAAGTTCTTTCTTTCTTCTTCTCTGTGGTGACTCAGATGCTTAACCCA
TACATCTACTCACTCAGGAACAATCAAGTCAAACAAGCCTTAAAGGAGAGAATGTTGA
GGGCATTTTCTAGCTCGCTGAGGCAGTTGTGA
>Fulmar_OR14J1 (Fulmar20)
ATGTCCAATAGCAGCTCCATGAATGAGTTCCTTCTCCCGGCATTCACAGACATGTGGGA
GCTGCAGCTCTTGCATTTCTGGCTCTTCCTGGGCATCTACCTGGCTGCCCTCATGGGAA
ACGGCCTCATCATTACAGCTATAGCCTGTGACCAGCGCCTTCACACCCCCATGTACTTC
TTCCTCCTCAACCTCTCCCACCTTGACCTGGGCGCCATCTCCACCATTCTCCCAAAAGC
CATGGCCAATTCCCTGTGGGACACCAGGGCCATCTCCTACTTGGGATGTGCTGCACAG
GTCTTTTTCTTTCTCTTCTTGATTGGAGGAGAGTTTTGTCTTCTCACTGTCATGGCCTAT
AACCGCTTTGTTGCCATTTGCAAACCTCTGCACTACAGGACCCTCCTGGGCAGCAGAG
CTTGTGTCCACATGGCAGCAGCTGCCTGGGGCAGTGGGTTTCTCAATGCTGTGCTGCC
GACTGCCAATACATTTTCACTACCCATCTGCCAAGGCAATGCTGTGGACCAGTTCTTTT
GTGAAATCCCCCAGATCCTCAAGCTCTCCTGCTCAGATGCCTACCTCAGGGAGGCTGG
GCTCGTCACATTTAGTGCTTTTCTGTTTTTGGGGTGTTTTGTTTTCATTGTGCTGTCCTAT
GTGCAGATCTTCAGGGTTGTGCTGAAGATCTCCTCTGAGCAGGGATGGCACAAAGCCT
TTTCCATGGGCCTCCCTCACCTGGCCATGGTCTCCCTGTTTATCAGCACCTTCATGTTTG
CCCTTCTGAAGCCCCCCTTCATCTCCTCCGCATCCCTGGATCAGGTGGTGGCATTGCTG
TACACTGTGGTGCCTCCAGCACTGAACCCCCTCATATACAGCATGAGGAACAAGGAGA
CCAAGGAGGCCCTGCGGAAACTATTTGAAGATGGACTACTGCAGCATCGATAA
>Fulmar_OR10A7 (Fulmar21)
ATGAACCATACAGAAAAAGTTGGTAGAAGCAACTGCACCACAGTGACCATGTTCATTC
TCCTGGGCCTCTTCAGACATACTGAGCTGCAGATCTTGTTTTTCTTCATGTTTCTCCTGA
TTTACACTATCACTATCATTGGGAACAGCCTTATCATCATTGTCACAGTTTACCCATCCTT
TTATACACCCATGTACTTTTTTCTTAGGGTCCTCTCCTTCGTGGATATTTGTACTACTTCA
GTTATTGTTCCCAAGATGTTAGTGAATTTTCTGTCACAGGACAAGAGCATTTCTTACATA
GGCTGTGCTGCCCAGCTGTACTTCCTGATTTTTCTAGCAGCTGCTGAGTGCTATCTTCTT
GTTGCCATGGCCTATGACCGTTACGTGGCCATTTGCAACCCCCTGAGATACACAATTAT
GATGAACAGAAGGGTTTGTTTTTCCCTAGTCCTGCTGTCTTTCCTCACTGGTAATGTTG
TGTCAGTGGTGCAAACTGCTTGGGTGTTCACATTGCCATTTTGTGGGCCTAACAAGATT
AACTATTTCTTCTGTGATATCTCACCACTTATTATACTTTTGTGCACTGAAACATCTCTGT
ATGAAATTCAGGCCACAACAACCACAGTATTGGTCATTTTCACCCCATTTTCTCTCATCC
TTCTGTCCTACATCTTCATCATCTCCAGGATTTTGACCATGCCCTCTGCAAAGGGAAGAT
ACAAGACCTTCTGCACCTGTTCTTCCCATCTCCTAGTAGTGATGCTGTACTACGGGAGT
GGCAGCCTGATTTACCTAAGACCCAAGTTCAGCTATCCACAAGATACTAAAAAAGTGC
TGGCTTTAGTTTACACAACCATAACTCCCATGTTGAATCCCATTATTTACAGCTTGAGAA
ATAAGGATGTGAAAAGGATCTTAAGAACAATGATAAGGAAGGTGAGGAAAAGGTAA
>Fulmar_OR8U9 (Fulmar22)
ATGGCAGAAAGCAACTGCACCCAAGTGACTGAGTTCAGCTTAATGGGGTTCACAGAG
GACCCAGTGACTCAGGCCACCCTCTTTCTGATATTTCTGCTCACCTATCTTGTCACCATC
CTGGGGAACTTTGGGGTGATCGTGTTAATCAGGGCCAGCCCTCAGCTCCGCTCCCCCA
TGTATTATTTCTTGGGTAACCTGGCCTTTGTAGACCTCTGTTCTTCCACCATTATCACCCC
CAAGATGTTGGTTGACTTCATATCAGAGAAGAAGGGCATTGCTTACGCTGGGTGCGTG

GCTCAGGTGTTCATTTTCGATCTTTTTGGGATGACCGAATGCTTCCTGCTGGCTATGATG
GCGTACGACCGTTACGTGGCCATTTGCCATCCCCTGGTGTATCCCCTTGTCCTGTCCCC
AAAATGCTGTTTCCAGCTGGTGACTGGGTCATACCTCGTGGGGTTGGCAAATGGTGTG
GGGCAGACTATTGGCATGTCCCGTTTATCCTTCTGTGGCTCCAGCGTCATCCACCTGTT
CTTCTGTGACATTTCCCCCCTGATATCTCTCTCAACCTCTGACACCACCCTCAGCCACAT
TATCCTAAGAACTTCAGCATCTTTATTCGGTGTGTCCAGCAGCCTGGTTGTCCTGGTTTC
CTACGTGGCCATCATCTCTACCATCCTGAGCATCAGTTCAGCCGAGGGCAAGCGCAAA
GCCTTCTCCACCTGCGCCTCCCACCTCACCACTGTGAGCATCTTCTACGGGACATCGTG
TTTTATGTACCTAAAGCCCAGCTCAGACAGCTCAAAGGAAGAAGACAAATGGGCTGCG
GTGCTCTACACTGTGGTGACTCCCATGCTGAACCCCTTGATCTACAGCCTGAGGAATAA
GGAGGTGAAGGAGGCTTTGAGGAGATTCACAAAAATAAAATGA
>Fulmar_OR4S2 (Fulmar23)
ATGGAGAATGCAAGCAGTGTGAAGAAATTCATTCTTCTGGGCCTCTCAGAGAATCAAG
GGGTGCAGAAAATATATTTGTGATGTTTCTGTTCTTCTATATGATTATTGTGGCAGGAA
ATCTGCTCATTATAATCACTGTAATTAGCAGTCAACGTCTGAACTCCCCCATGTATTTCTT
CCTCTGCTACCTGTCCTTTGTAGATATCTGTTACTCTTCTGTCACAGCTCCCAAAATGAT
TGCAGACTTCCTTGTTGAAAATAAAACCATCTCCTTTGTGGGTTGCATAGCACAGCTGT
TTGGGGTGCATTTCTTTGGCTGCACAGAGATCTTCATCCTCACAGTGATGGCCTATGAT
CGCTGCATTGCCATCTGCAGACCTCTCCACTACACCACCCTCATGACCAGGCGTGTATG
TAGCCGGATGGTGATCGGCTCGTGGGTAGGAGCCTTTGTGCACTCCATAGTGCAGACT
CTTCTAACTACTCAGCTCCCCTTCTGTGGCCCTAACAAAATTGACCACTACTTCTGTGA
TGTCCACCCCCTACTACAACTGGCCTGTACCGACACCTACGCTGTGGGCATCATTGTCG
TTGCCAACAGCGGAATGATAACTCTGATCTGTTTCGTCATCCTGGTCACATCCTACGTT
GTCATCCTGATTTCCTTGAAAAGTCAAACATCTGAAGGGCGGTACAAGGCCCTTTCCA
CCTGTGGGTCCCACATCACTGTGGTGATTCTGTTCTTCGGGCCATGCACGTTCATCTAC
ATACGTCCATCCAGCAATCTGTCGGAGGACAAGAGCGTGGCAGTGTTTTACACTGTCA
TCACACCCATGCTGAACCCACTCATCTACACGCTAAGAAATGAGGAGGTGAAGAGTGC
CATGAGAAAACTGTGGAGAAGAAAAGTGGGAAGTGAAAATGGAAAGGTGTAG
>Fulmar_OR51E2 (Fulmar24)
ATGCCCTTCCCCAACAGCTCTGACCTCAGCCCATCCTCCTTCATCTTGGCCAGCATCCC
AGGGCTGGAGGCTGCCCATTTCTGGATGGCGATGCTTCTGTGCTCCGTGTACATCTTGG
CGGTCACAGGCAATTGCGCGGTGCTGTTCATCGTAAAGACAGAGCCCAGCCTGCATGC
TCCCATGTACTTCTTTCTCTGCATGCTGGCTGCCATCGACTTGGCCTTGTCCACGTCCAC
GGTGCCACGTGTCCTCTCCTTCTACTGGTTCAACACCAAGGAAATCAGCTTTGGCGCTT
GTCTTGTCCAGATGTTCCTCATCCACACCCTCTCAGCCATCGAGTCCACTGTCCTCCTG
GCCATGGCCATGGACCGGTACGTGGCCATCTGCCACCCGCTCAGACATGCTGCCATCCT
CACCAATGCTGCGACAGCGAAAATAGGGCTGGTAGCCATGGCCAGGGGAGTTCTCTTC
TTCCTGCCTTTGCCTTTGCTCCTCCTGCCCCTTCCCTTCTGCAGCTCCCGGGTGCTGTC
ACACTCCTTCTGCCTGCACCAGGACGTGATGAACCTGGCCTGTGCCAACACCCCCCCC
AGCGGGGTGTACGGCCTCACCGCCATCCTGCTGGTCATGGGGGTGGACGCCATCCTCA
TCTGCCTCTCCTACGTCCTGATCCTCAAGGCTGTCTTGCGGCTGGCGTCGTGGAAGGA
GAGGCTCAAGGTGTTCAGCACTTGCGTTGCCCATATCTGCGTGGTCCTGGCCTTCTACG
TGCCCCTGATTGGGCTGTCCGTGGTGCACAGGTTTGGGAAGGATCTGGCTCCACTGGT
CCATATCACCATGGGGAACATCTACATCCTGGCGCCCGCTGTGCTCAACCCCATCATCT

ACGGGGTGAGGACCAAGCAGATACAGAGGAGGATCCTGAATTTAATTCATATATACAAT
GACAGAACTGCCCAGTGA
>Fulmar_OR52R1 (Fulmar25)
ATGTCTCTGAACTCTACTGCCTTCTCCCACCCTCCCTATTTCCTACTCATTGGCATCCCT
GGGCTGGAGAAGGAGCAGTTCTGGATTGCCTTCCCCTTCTGCATCATGTATGTCATTGC
TGTGCTGGGGAATGTGACCCTTCTCCTCATTATAAAGGCAGAGCCGAGCCTGCATGAG
CCCATGTACCTCTTCCTGGCCATGCTGGCCTTTACTGACCTGGTCCTATCAACATCCACG
TTACCCAAAATGCTTGGCATCTTCTGGCTGGGCTCTGGGGAGATTGGCTTCCTCTCCTG
CCTTGCTCAGTTGTTCTTCATCCATACCTTCTCGTCGGTGGAGTCGGGCGTGCTCATGG
CTATGGCCTTGGATCGCTACATCGCTATTTGCCACCCGCTGCGGCACTCCAGCATCCTCT
CTGTGCCGGTGGTGGCGGCCCTCGGGAGCCTGGTGCTGGTGCGTGGGGTCCTCCTGGT
GAGTCCCTTCTGCTTCCTCCTCCACAGGATGCCCTTCTGCCAGCATCATGTCATCTCCC
ACTCCTACTGCGAGCACATGGCCGTGGTGAAGCTGGCATGTGGGGACACCAGAGTCAA
TGTCATTTACGGCCTCTTCGTGGCTTTCATAGTGATAGGATCTGACATGATGCTGATCAT
TGTGTCCTATACCATGATCCTGCGGGTGGTAATGAGGCTGTCATCCACAGAGGCACGAC
TTAAAGCCTTCAGCACTTGTGCATCCCATATCTCTGTCATCCTTGCCTTTTATGTCCCTG
CCCTCTTCACATTCCTCACCCACCGGTTTGGGCAGAGCATCCCTCCCCACATCCATATAA
TGGTAGCCAACCTCTACCTGCTAGTGCCCCCCATGTTAAACCCCATTGTTTATGGGGTG
AAAACCAAGAATCTCTGGGACAGGGTGGTCCTCCTCTTCCAGTGA
>Fulmar_OR10R2 (Fulmar26)
ATGGAGAGGGACAATAAGAGCACTGATGCAACCATGGAGTTCCTCCTGCTCGGCTTCT
CCGAGCTGTTCTATCTGCGGGTCCTCCTCTTCCTTATCTTTCTCATTATCCATTTGGTCAC
GTTGGCAGGGAATATGATGATCTTCATGGCAGTGGTTATGGAGCCTTCCCGTCCTCCCA
TGCTTTTCTTCCTCTGTCAGCTCTCTGTCATCGAGCTCTGCTATACCTTAGTCATTGTCC
CTAAGGCACTCCTCAGCCTGATAGTGGTGGACGGCAGCACCATTTCTTTCATAGGCTGT
GCTGCACAGATGCACTTTTTTGTGGCACTCGGTGGGGCTGAATGCTTCCTCCTGGCAG
CCATGTCGTATGACCGTTATGTTGCCATCTGTCAGCCACTTCACTATGTAGCTGTGATGA
GTGAGGAGCTCTGCCTCAGGCTGGCTGTGGCATGCTGTCTGGGAGGCTTTGCTGTTGC
CCTGGGGTTGACGGTGGCTGTTTTCCGCTTACCTTTCTGTCAGTCTCATCGCATCAACC
ACTTCTTCTGTGATGTCCCTGCTGTGCTGCATCTGGCCTGTACACAGAGTTACACTCCT
GAGCTGCCCTTGCTGGCTGCCTGTGTGGTCCTCCTGCTGCTCCCCTTACTCCTAATCCT
GACCTCATATGTTTGCATTGCTGTTGCTTTGTTACATGTCACCTCCTCCGCAGGAAGGG
GCAAGGCCTTTTCCACCTGCATTTCACACTTGGCCATCACCTTCCTACACTATGGATGT
GCCACCTTCATGTACATTCGTCCTAAGTCCAGTTACTCACCGGCTCGAGACAAGATGGT
GTCTCTGGTCTACACCAACATTACTCCATTACTGTATCCCCTCATTTATAGCCTGAAGAA
CAAGGAAATCAGAGGGGCCCTCAGGAAAATGTTCAGGAGGAAGAAAATAGCTCAGCT
GAACTGGGATACTATCAGAGCTGTGATGTGTGTGTGTGGTAAATTTTAG
>Fulmar_OR2A2 (Fulmar27)
ATGCAGAATGAAACATCTGTCACAGAGTTCATCCTCCTGGGCTTCTCCAGCAACCCAA
CCCTGCAGCTCTGCCTCTTCGGCATTTTCTCTGTCCTCTACTCTGCCACTCTGATGGGA
AACGCACTTGTCTTTGTGCTTATCTGCCTGGACTACCGCCTCCACAGCCCCATGTACTT
CTTCCTCTGCCACCTCTCCATCATGGACATCTGCTATGCCTCTAACAATGTCCCCCATAT
GCTGAGGAACCTCCTTGGACAAGGCAGAACCATCTCCTTTGCTGGGTGTGGGACACA
GATACATCTTTATTTAATCCTTGCACTTACAGAGTGCGTGCTGCTGGCCGTGATGTCTTA

TGATCGCTACGTGGCAATCTGCCATCCCCTCCGCTACGTCCTTATCATGAGCTGGAGGG
TGTGCCTCACCCTTGCCGCAGTTTCATGGGCTTTGGGGTTCCTATTTGGTACACTACAA
GCCTCTCTGGCTTTACACCTACCTTTTTGCGGCCCCTGTGAGGTTGATCACTTCTTCTGT
GAAATCCTGGCTGTCTTAAAGCTGGCCTGCGCTGACACTACCGCCAATAAAGTCCTGAT
CTTTGCTGTTTGCGTGTGCTTCCTCCTCTTCCCTTTAGCCTTAATACTAATTTCCTACCTG
CATATCCTGGCCACCATTCTGCGCATCCGTTCTGCAGCAGGATGGCACAAAACCTTCTC
CACCTGTGGCTCCCACCTGACCGTGGTGTGTCTGTTTTATGGAAACGCCATCTTCATGT
ACATGGGGCCCGGGAGCGGTAACTCATCTGGGAGGGAGAAAGTTCTTTCCCTTTTCTA
CAGTCTCATCAGCCCAAGTTTGAACCCCCTGATTTACAGTCTGAGGAACAAGCAGGTG
AAGGAAGCCTTGCTGAAGCTTCAGAGAAGGAAAAGAGTTTTTCATTCCGTTTCATTCC
GTGGGTCTGTTTTATGGAAACGCCATCTTCATGTACATGGGGCCCAGGAGCAGTAA
>Fulmar_OR6Q1 (Fulmar28)
ATGGTTTCTGACAACAGGACTTTGGTAACTGAATTCATCATCCTAGGATTTCCCAATGT
CAGGGAAGTCGAGCTGCTTCTCTTTGCTACCTTCCTTCTGATGTACGCACTGACCTTCA
CAGAGAATGTGGCCATCATCTTGGTCATTGGCATGGACTACCGCCTTCATACTCCAATGT
ATTTTTTTATCAGTGATCTCTCCCTCCTAGACATTGGCTACACTACTGTGACAGTGCCCA
AGATGCTGGCTAACATTGCTATTCAGTCACAGACCATCTCTATCACTGGCTGCTTCACA
CAGTTGTACATCTTCTTCTTCCTTGGCTCAGCTGAATGCTTCCTACTGACTAGCATGGCC
TATGATCGCTACCTGGCCATTTGCAATCCATTGCATTACACCACCATTATGGATTGCAGA
GCTTGTTTATGGCTTGCTCTGGGGTGTTGGTTGGGTGGCTTCCTTGCACCTGCCCTTCC
CACTGCCTTCATCTTCCGCCTGCATTTCTGTGGTTCCAACATCATCAACCACTTCTTCTG
TGACTCACCACCTTTGCTGGAGCTCTCGTGCCAAGACGTCTTTGAAATCAAAGTCATC
AACTTTCTGGTAGGCACAATAGTACTAATGAGCTCCTTCATCCTCACCATGATCTCCTAC
ATCTTCATTGTGGCCACCATCTTGCACATACCCACAGCTGAAGGAAGCAGCAGCAAAG
CCTTCTCTACCTGTGCCTCCCCTCTGACCACTGTCACCACCTTCTACGGGACTACCATC
TTCATGTACATTCGCACCAAGACCATCCAGACTTTTGATTTCAACAAGACTGTCTCTGT
CTTTTACTCTGTGGTTACCCCAGTTCTGAACCCTGTCATCTATAGCCTAAGGAACAATG
ACATCAAGCAAGGCATGAGGAAAGTACTTCTGAAGCAAAAGGACACTTTCTTCATGTA
A
>Fulmar_OR8D4 (Fulmar29)
ATGGTGGCCAAAGGAAATCACACCCCCAGTGCTGAATTTGTTCTCTCGGGGTTCTCAG
AGCAGGGGGATGTCCAGGCTGTCCTCTTCATGGTCTTCTTGGTGTTCTACGTGATCACT
CTGCTGGGGAATCTGGGGGTGCTCGTGTTAATCAGGCTGGATGCCCAGCTTCACACCC
CCATGTACTTCTTCCTGAGCAGCCTGTCCTTCCTAGACATCTGCTATTCCTCCTCAATCA
CCCCCAGGCTGCTCTCAGATCTCCTATCAGAAAGGAAGGTCATTTCTTACTCTGCGTGC
CTCACACAACTTTATTTCTATGCGGTCTTTGCCACCACCGAGTGCTACCTCTTGGCCGT
GATGGCGTATGACCGCTACGTGGCCATCTGCAGCCCACTGCTCTATGCCATCTCCATGT
CCAGCAGAGTTTGTGCACTGCTGGTAGCTGGCTCGTACCTCGCTGGGATCGTGAATGC
CACTATCCACACGGGGTTTGCACTTCGGCTGTCCTTCTGTGGTCCCAACATCATCAACC
ACTTTTACTGTGAGGGCCCCCCGCTTTTTGCCATCTCTTGCACAGACCCCACTGTCAAT
GAGATTATGATGTTCATTGTGGTGGGCTTCAACCTGTTTGTCACCAACCTGACCATTCTT
ATCTCCTACACCTACATCCTGGCCACCATCTTGAGGATGCGCTCGGCCGCGGGCAAAC
ACAAAGCCTTCTCCACGTGCGCGTCCCACCTGACCGCCGTGATCCTCTTCTACGCATCT
GCTGCGTCCATGTACTCACGACCCAGTTCCAGGCACTCCCAGGACCTCGACAAAGTGG

CCTCTGTGTTTTACACTGTGGTGACCCCCATGCTGAACCCCCTCATCTACAGCCTGAGG
AACAAGGAGGTGAAGAATGCGCTGGAGAGAGTGATGGAGAGGAAACGTTCATCTGAA
AAATAG
>Fulmar_OR4D5 (Fulmar30)
ATGGCACTGGGAAACTTCTCCCGAGTGACTGAATTCATCCTCCTGGGGCTTTCCGATAC
AAGGGACCTGCAAGTCCTCTTCTTCACCTTTTTCTTCCTTGCCTATGCCCTGGTTCTGCT
GGGGAACCTTCTCATCATTGTGACAGTCAGGAGCGACCCCAAGCTGTCCTCACCCATG
TACTTTCTCCTTTGCAATTTGTCCTTCATAGATATTTGCTGTACCTCTGTCACCTCTCCCA
GGATGCTGGTGGACCTGCTCTTCCAGAGGAAGGCAATTGCATTTGAAGACTGTATAGC
TCAGCTGTTTTTCCTGCATTTTGTTGGGGCATCAGAGATGTTCCTCCTGACTGTGATGG
CATATGACCGCTACACTGCCATCTGCAAGCCCTTGCACTACGCAGCCATCATGAACCGG
CAGGTGTGCTGGGTCCTGGTGTCTGCCTGCTGGGCAGGGGGCTTCCTCCACTCCATTG
TCCAGACGCTGCTCACAATCCAGCTCCCCTTCTGTGGCCCTAACACAATCGACAACTAT
TTCTGTGACGTGCCTCCTGTCATTCGGCTTGCCTGCACAGATATCTATGTCATTGAGTGG
CTCATGGTTTCCAACAGTGGCTTAATATCCCTGGTTTGCTTCCTGGTGCTGGTCACATCT
TACACATTCATCCTGGTCACAGTCAGGGTCCGCTTCACGGAGGGACACTGGAAGGCGC
TCTCCACCTGTGCCTCACATGCGATGGTCGTCACCCTCTTCTTTGTACCCTGCATCTTCA
TCTATTTCCGGCCCTTTTCTACCTTCCCCTCTGACAAGCACATCTGTGTGATCTACACTG
TCTTATCCCCGGTTCTGAATCCCCTCATCTATACCCTGCGGAATAACGAGGTGAAAGCG
TCCATGCGGAAATTGTGGAAGCGCTGCAGAGTCTTCTGA
>Fulmar_OR5R1 (Fulmar31)
ATGGGAGGAAATCACACGCAGACTAAATTCATCCTCTTGGGAATTACAGACAGTCCAC
GTGTGCAGGCCCCTCTGTTTGGCTTGTTTCTCTCGATTTACGTTGTCACTTTGGTGGGG
AACGTTGGGATTATGGTCTTGGTGTGGGCGGTTCCCAGCCTCCACACGCCCATGTACTT
CTTCCTCGCCCATTTTTCATTTGCTGACGTCTGCTATTCCACAGTCATCTCCCCCAAAAT
GCTAGCAGACCTGTTATCGGAGAATAAAACCATTTCTTTCGCTGGCTGCGTGACGCAGT
TCCATGGCTTCGCTTTCTTTGCGACTGCCGAGTGTCACCTCCTGGCTGTGATGGCCTAT
GACCGGCATGTCGCTATCTGCAACCCCCTGCTTTACGTGACGGTCATCTCCAGCCGCGT
CTGCCGGCAGCTGGTAGCGTCAGCCTACACCGTCGCCTTTCTCAGTGCCGTCATCTACA
CAGGCTGCACGTTCGGGGGTTCCTTCTGTGGACCCAACCAGATCGACCACTTCTTCTG
CGACGTCAGCCCCGTGCTAAAGCTTGTGTGCTCCGACACCCACGGCAGCGAGATGGTC
ATCTTTGCCTTTGTCGCCATAAACGCGGTGGGCACGAGCGCGATCATTTTGCTCTCCTA
CCTCTGTATCCTCCGCACGGTCCTGAGGATGCGCTCAGGGCGGAGCAGGTCCAGAGCC
TTCAACACCTGCGCCTCCCACTTGACGGCTGTTTCCTTATTCTACGGGACAATATTCTTC
ATGTACCTACAACCTGCGTCTAGCCACAGCAGCCTGGATAAGGTGGCCTCCATCTTCTA
CACCGTGGTCACCCCCATGCTCAACCCGTTCATCTACAGCCTGAGGAACAAGGAGGTG
AAGGGTGCTCTGGTCAAGTGCAGGAGAAGGATGTTAAACCGCTGTCAACATAAAAGA
GTTGTATCAGCTAGGAAGTGA
>Fulmar_OR5J2 (Fulmar32)
ATGGCAGAGCAGAATCACACCTCAGTGACAGAGTTCATTCTCGAGGGCCTGAGTGACC
AAGCGGAGATGAAGGCAGCCCTCTTTGTGCTGTTCCTGCTCATCTACACCATCACCCTT
GTGGGCAACGTGGGGATAATCGTAGTCATCCGAGGTGACCCACGGCTCCACACGTCCA
TGTACTTCTTCCTTGGCAGCCTCTCCGTTGTTGACATCTGCTTCTCCTCTGTGATTGCCC
CCAGGACCTTGGTGAACTTCCTATCGGAGACAAAGACCATTTCCTTTGTTGGCTGCATG

GGCCAAACCTTCTTCTACATCGTCTTCGTGACGACTGAGTGTTTCCTGCTGGCCGTCAT
GGCATACGACCGGTATGTGGCCATCTGTAACCCCCTGCTCTATTCCTCTGTTATGTCTCC
GAGTGTCTGCATGCGGTTGGTGGTGGGGTCCTACATTGGGGGTGTCCTGAACTCCATCA
TACAGATGACCTTCATCATTAGGCTGCCCTTCTGCAGCTCCAATGTCATCAACCACTTCT
TCTGCGATGTTCCTCCCCTCCTGGCTCTGTCCTGTGCCAGCACCTATGTCAACGAGATG
ATCCTCTTCTCCATGGCCGGTATCATTGAGCTCAGCACCATCTCCACCATCCTGGTCTCC
TACATCTTCATCTCCTTTGCCATCCTGAGGATCCGTTCAGCTGAAGGCAGGCAAAAAGC
CTTCTCCACCTGCGCGTCCCACCTGACAGCAGTGACCGTGCTGTACGGGACGACAATC
TTCATGTATTTACGCCCCAGCTCTAGTTACTCCCTGAACACTGACAAAGTAGTCTCCGT
CTTCTACACGGTGGTCATCCCGATGCTGAACCCCCTCATCTACAGCCTGAGGAACCAG
GAGGTGAAGGATGCTCTGAGGAGAACGGCAGAAAGGATCACAGTCAGGCTCTGA
>Fulmar_OR2A5 (Fulmar33)
ATGGTTCCAAACAGAAACAACAGTCAAACACTACTGACAGAATTCATCCTCTTAGGTC
TGCCTGGTGATCCAAAGTTGCAGCGATTCATACTTGGCTTCTTCACCCTTATCTACATTA
TAACATTGCTAGGAAATTTACTTATCTTCTGTCTTATCCACCTGGATCCTCATCTGCATAT
TCCAATGTACTACTTCCTCAGGCATTTATCTCTCATAGATATATGTTACCCTTCCAGCACT
TTTCTTCCATTGCTCATTAGCTTTGTAGCTCAGAGAAGAACTATCTCATTAACTGGCTGT
GCAATTCAAATGTACATGTTTCTTGTGCTGGCAACTACTGAATGCATCCTCCTGGCAGT
CATGGCCTACGACCGACGTGTGGCCATATGTCTCCCACTCCACTACACAATGACCATGA
GCAACAGGAAGTGTGTTGCATTAACTTCACTCTCATGGGCGAGTGGCCTGGTACTGCC
AATCGCGCACACTGTCCTGACGTGGAGGCTGCCTTACTGTGGTCCCAATGTGATTGATC
ACTTCTTCTGTGAGATGCCTGCTGTACTGCAGCTGGCATGTGCTGATACATATCTGATCA
AGCTGGTCACTCAAGTGGGATGTCTCTTCACCCTGTTGATGCCTACCACTTTCATTATTT
TCTCCTACATGGACATTTTGGTAGCAATTTTGAAGATAAACTCTGCCAAGGGAAGAGA
AAAGGCCTTCTCCACTTGCCTCTCACATTTGATGGTTGTAGTTCTGTTTTATGGAAGTG
CCATCTACATGTACATGAAACCAAACTCTTTCCATTCCCCTCAGAAGGACAAAATCATC
TCTATCTTATATAATATTCTTACCCCAATGTTAAATCCAATTATTTACAGCCTGAGGAACA
TGGAGATCAAGAAAGCATTGGTGAGGCTTATTAAAAGGGAAACATGGCCCTAG
>Loon_OR14J1 (Loon1)
ATGTCCAACAGCAACTCCATCACTGACTTTCTCCTCCTGACATTCACAGAAACAAGGG
AGCTGCAGCTTTTGCACTTCTGGCTCTTCCTGGGCATCTACCTGGCTGCCCTCCTGGGC
AACAGCCTCATCATCACAGCCATAGCCTGTGACCACCGCCTCCACACCCCCATGTACTT
CTTCCTCCTCAACCTCTCCATAATCGACCTGGGCTTCATCTCCACCACTGTCCCCCAGT
CCATGGCCAATTCCCTCTGGGACACCAGGGCTATCCCCTACCTGGGATGTGCTGTACAG
GTCTTTCTGTTTGTCTTTTTGATTGTAGCAGAACTGTATCTCCTCACTGTCATGGCCTAT
GACCGCTATGTTGCCATCTGCAAACCCCTGCACTATGAGACCATCCTGGGCAACAGAG
CTTGTGCTAAAATGGCAGGAGCTGCCTGGGGCAGTGGGTTTCTCACTGCTGTGTTTCA
CACTGCCAATACATTTTCACTTCCATTCTGCCAAGGCAATGCCGTCGACCAGTTCTTCT
GTGAAATCCCCCAGATCCTCAAGCTCTCCTGCTCAGATGACTACCTCAGGGAAGTTGG
GCTTATTGTATTTGGTGTCTGTTTGGCGTTTTTTTGTTTTATTTTCATTGTTATGTCCTATG
TGGAGCTCTTCAGGGCTGTGCTGAGGATCCCCTCTGAGCAGGGACGCAACAAAGCCT
TTTCCACGTGCCTCCCTCACCTGGCTGTGGTCTCCCTGTTTATCAGCACCGCCATGGTT
GCTGGCCTCAAACCCCCCTCCTTTTCCTCCCCATCCCTGGATCTGGTGATGGCTGTTCT
GTACTCGGTGGTGCCTCCAGCAGTGAACCCCCTCATTTACAGCATGAGGAACAAGGAG

CTCAAGGATGCCATTAGGAAAGTGGTTTCAGGGCTGTTTCTCAATAGTGATAAGCTCAC
CATCTCTCTTCACAAGTGA
>Loon_OR5AP2 (Loon2)
ATGGGAGGAAATCATACGCAGCCTAAATTCATCCTCTTGGGAATTACAGACAGTCCGTG
TGTGCAGGCCCCTCTTTTTGGGTTGTTTCTATTGATTTACATTGTCACTTTGGTGGGGAA
CATTGGGATTATGGTCTTGGTTTGGGTGGTTCCCAGCCTCCACACCCCCATGTACTTCTT
CCTCACCCATTTTTCATTTGCTGACATCTGCTCTTCCTCAGTCATCTCCCCCAAAATGCT
AGCAGACCTGTTAGTGGAGAATAAAACCGTTTCTTTCGCTGGCTGCGTGACGCAGTTC
TACAGCTTTGCTTTCTTTGCCACTGCCGAGTGTCACCTCCTGGCTGTGATGGCCTATGA
CCGGCACGTTGCTATCTGCAACCCCCTGCTTTACGTGACGGTCATCTCCAGCCGCGTCT
GCCGGCGGCTGGTAGCATCGTCCTACCTCATTGCCTTTCTCAGTGCCATCATCTACACA
GGCTGCACGTTTGGAGGTTCCTTCTGTGGACCCAACCAGATTGACCACTTCTTCTGCG
ACGTCAGCCCCTTGCTAAAGCTCGTGTGCTCTGACACCCGCAGCAGCGAGATGGTCAT
TTTGGCCTTTGTCGCCATCAACGCAGTGGGCACGAGCGTGATCATTTTGCTCTCCTACA
CCTGTATCCTCCGCACGGTCCTGAGGATGCGCTCAGCACAGAGCAAGTCCAGAGCCTT
CAACACCTGCGCTTCCCACTTGACAGCTGTTTCCTTATTCTACGGGACAATATTCTTTAT
GTACCTACAACCAGCATCTAGCCACAGCAGCCTGGATAAGGTGGCCTCCATCTTCTACA
CGGTGGTCACCCCCATGCTCAACCCATTCATCTACAGCCTGAGGAACAAGGAGGTGAA
GGGTGCTCTGGTGAAGTGCAGGAGAAGGGTGTTAAACCGCTGTCAACATAAAAGAAT
TGTATCAGCTAGGAAGTGA
>Loon_OR5B21 (Loon3)
ATGGCTGAGGAAAACCAAACCAAGCAAGTTACAGAGTTGACTCTTGTGGGACTGACA
GACAACCCACGACTACAAGCGCCTTTGTTTGTCCTGTTCCTCCTCATTTACTTCATCATG
CTGGTGGGGAACCTGGGCATGGTTGTGCTGATCAGGAGGAGCAGTCAACTTGAATCAC
CCATGTACTTTTTCCTTAGCAATTTATCTCTCCTAGATGCTTGCTACTCGTCGGTGGTGG
CGCCGAGGACATTGATGAACCTTCTGATGGAGAAGAAAACAATCTCTCTAGCCGGCTG
CACCACCCAGCTTTTTTTTTTCATAGCTTTTGGGACCACTGAGTGCTTCCTCCTGGCTG
CAATGGCGTACGACCGCTACGCAGCCATCTGCAACCCTTTGCTTTACCAGGCTGTCATG
TCTCACAAGGTCTGCATCCTGCTGGTGGCCGGTTCGTACGTGCTTGGCCTGCTGCACTC
TTCGGTGCACACGGACTTCACCTTCAGCCTGCCCTTCTGCCAGCCAGCCAAGGTCGAC
CATTTCTACTGTGACCTCATGCCCGTTCTAGCGCTCTCCTGCTCCGACACCCATGTCAA
CAGGCATCTGATATTCGGCCTCGCGGGACTGGTGGAGTTGGTGACCATCTTGGCCGTCC
TGGTCTCCTACGCCTTCATCCTCTCCTCCATCTCAAAGATCAGCTCTGCCAAGGGCCAG
CACAAAGCCTTCTCCACTTGCACCTCTCACCTGGCTGGAGTCACCATCTTCCACGGGT
CTATCCTCGCCCTGAACTTCCGACCAAGGTCCAGCGACACCCTGAGCACGGACAAGAT
CTTTGCTGTGTTTTACTCGCTGGTGGTGCCCCTGCTGAACCCCCTGATCTACAGCCTCA
GGAACAGGGAGGTGAAGAACGCCCTGAGGGAGTTTGTCTTGTGGAAGTAG
>Loon_OR5J2 (Loon4)
ATGAAGTACGGCAATACGGCTAAAGGCAATCGCACCATGGTGACCCAGTTTGTCCTCCT
AGGACTGACAAGTGAGCCCAAGCTGCAGGCTCCTCTCTTCATAATCTTCTTAATTATTT
CTCTCATCACCTTGATGGGCAACCTCGGGCTGATCACATTGATCAGGACAAACCCCCG
GCTGCACACTCCCATGTACTTCTTCCTCTGCAATCTGTCTGTTGTTGATCTTTGCTACTC
CTCCGTCTTTTCGCCAAAGCTGCTTATTGGCTTCTTGGTGGAAAAGAAAACCATTTCTT
ACTCCGCCTGCTTTGCCCAGCATTTCTTTTTCCTTGTGTTTGCGACCACAGAGGTGCTC

TTGCTGGCTGTGATGGCATACGACCGCTACATAGCCGTTTGCAACCCGCTGCTCTACGC
TGTGTCTATGCCCCAGAGGGTCTGCGTTCAGCTGGTGGCCGGGTCATACGTAGGGGGG
ATTTTGAACTCACTAATCCAAACGTGTTGCTTGCTGCCGTTGCCTTTTTGTGGCCCCAA
TGTCATCAACCATTACTTCTGTGACACTAACCCTCTGCTGAAACTCACCTGCTCCGATG
ACCACCTCAATGAGCTTTTGCTTGTAACCTTCAACGGGACCATCTCCATGTCTGTGCTC
TTCATCATCATCATCTCCTATGTATACATCCTCTTCTCCATCCTGAGGATTAGGTCTGCCA
AAGGAAGGCACAAAGCCTTCTCCACCTGTGCCTCCCACCTCCTGACCATTACCCTGTT
CTACGTGCCCGCGGGGCTGAGCCACATGCAACCGGGCTCCAAGTACTCACTGGAGATG
GAGAAAGTCACCGCCGTGTTTATACCCTGATCGTCCCTATGCTCAACCCTCTGATCTA
CAGCTTGAGGAACAAGGAGGTCAAGGATGCGCTTAGGAAAGCGACAGCAAATAACGT
TTTTGTGAGTTGA
>Loon_OR52K1 (Loon5)
ATGTCAACCGCCAACCGATCCAACACCAACTCTTCGCCTTTTCTCTTGATGGGCATCCC
TGGCCTGGAAGCTTTCCACGTGTGGATTTCCATCCCATTCTGCTTTACATACATCATGAC
CTTGCTGGGAAATAGTATGGTCCTTCTTGCTGTGAGGCTGGACAAAAGCCTCCACAAG
CCTATGTACTATTTCATTTCCATGTTGGCTGTCATTGACCTCATCTTCTCAACTGCTGTAG
TTCCCAAAATGCTGGGTGTATTCTGGTTGGATTCAAGGGAGATTGGTTTTGAGGCCTGC
TTCATCCAGATGTTCTTCATCCACACATTCACTGCAGTGGAGTCAGGGGTGCTCCTGGC
AATGTCCTTTGACCGGTATATAGCCATCTGCAACCCCTTGAGATACAACACCATCCTAAC
AAGCTCAAGGACCATCCAGATAGGACTGCTGTCTCTGGCCAGGGGAGCTGGTGTCATG
ACACCATTAATGTGCCTCCTCACCAGCTTACCCTACTGCAAAACCAGAGTCATCCCTCA
TTCCTACTGCGAGCACATGGCTGTGGTGGAGCTGGCCTGCGCCGACCCGTCTGTCAGT
GATCTCTACAGCCTCATTGTGGCAACACTATTGGTGGGGGCAGACTCTGTCTTCATCAC
TTTCTCCTATGGTATGATCCTCAGGTCTGTGATGAGGCTGCCATCCCAAGAGGCACGTC
TCAAGGCACTCAGGACCTGCGGGTCCCATGTTTCCATCATCCTGCTGTTTTACATAGGT
GGCCTACTCTCCATGTACCTGCAGATGTTCTCTTTCGGCTTGGCACCTCATGTCCAAGT
CCTGGTGGCTGATTTCTATTTGACGGTCCCTCCCATGCTCAACCCCCTCATTTACGGCAT
AAAGGTGAAGCAGATCCAGGAAGGGATCTTCAAACTATGGGGACAGTTGCCAGGACT
CAATATCTCACAAGCTGATAAAGACAGGTCAGATGCTGAGAGAGGGAGGTACTGCCAG
AAAATAATTTATCCCATCCTAGAATAG
>Loon_OR6A2 (Loon6)
ATGGAGGTGAGCAATCAGAGCATGGTCAAAGAATTCATTCTCCTTGGATTTCCCATTGC
TCCTCGCTTGCAGATTCTCTTCTTTGCAGTATTTCTCACTGCATATCTCTTAGTTTTAGCA
GAAAATATTATCATCATCCTGACTGTCTGGACGAACTATAACCTCCACTCCCCCATGTAT
TTCTTTCTGAGTAATTTGTCCTTCCTGGAGATCTGGTACGTGACAGTTACACTCCCCAA
GACAATGCTGAGCATTGTATCAGTGACAAAGCAAATCTCCTTCATGGGATGCATGACAC
AACTGTACTTCTTCCTCAGCCTGGGCAACACTGAATGCCTCCTCCTGGCTGTCATGGCA
TATGATCGCTATGTTGCCATTTGCAAGCCTTTCCATTACTTGACTATCATGAGACACACT
GTCTGTGTCTACCTTACTATGGGCTCTTGGTTGACTGGTTTCTTAATTTCTGGATGCAAA
GTCTTTTTCATCTCTCAGTTAACATACTGTGGACCCAATATAATCAACCATTTCTTCTGT
GATGTTTCTCCGCTCTTGAACTTAGCCTGTACAGATATGGAAAGAGCTGCTCTTATGGA
TTTTGTGGCTGCCTTATTTATTCTCCTTATGCCTCTCTCTGTAGTAATCCTATCCTATACCT
ATATTATCTTCACTGTCCTTCACATTTCATCTGTACAAGGTTGCCAAAAAGCCTTCTCCA
CCTGTGCCTCCCACCTTGTAGTGGTCATTGTCTTTTATGCAACAAGCATTTTTATCTATG

TGAGGCCCAGAACACTTCCAGTTCATGACACAAACAAAATAGTGTCTGCTCTCTACGC
TGTTGTTGTTCCTCTCTTCAATCCCATCATTTACTGCCTGAGGAACCAGGAAATCAAGG
ACGCCCTCCAAAAGATCTTGTTCAGAAAAAGAGTTTCCTTGCAGAAACATTAG
>Loon_OR2AT4 (Loon7)
ATGGAAAGCTGCAGCGGCAACGCTTCCACTAAAGTCTTTTTCTTGGTGGGGTTTCCAG
CACTCCAGGATTTCCAGACGCCCCTCTTCGTTGTGTTCTTGCTATTCTACCTGCTGATCC
TGGTTGGTAATGCTGTTATCATCACCGTGGTTGCGGTCAACCGTACACTCCACAAACCC
ATGTACTTTTTCCTGATTAACCTCTCTGTGTTAGATGTGCTATTCACAACCACCACCATC
CCCAAAATGCTGGCGATGTTCCTGGCCAATGCTAAAACCATCTCATTTCGGGGCTGTTT
TCTGCAGATGTACAGTTTTCATGGGTTGACGGTAACCGAGGCGCTTCTCCTGGTGGTCA
TGGCTTACGACCGCTATGAAGCCATCTGCAACCCCCTTCATTACGCGGCCAAGATGACA
AGAAGAGTGAACATCCAGCTGGCAGCAAGCGCCTGGATCACTGCGCTGCTAATACCCG
TGCCTGTCATCACACAGACCTCTCGGCTAGCTTACGGAGACACAACCAGGGTTCACCA
CTGCTTTTGTGACCACCTGGCAGTGGTACAAGCTGCGTGCTCGGACTTCAGTGCCGAT
TTCCAGACCTTCTTGGGGTTCTCCATTGCTATGAGTGTGTCGGTCATCCCTCTGTTGCTC
GTCACCATCTCGTACATCCACATCATCCTCTCCGTACTAAAGATCAACTCCAAAGAAGG
ACGCATGAAAGCTTTTTCAACATGTACTTTCCATCTGCTTGTGGTGGGCACTTACTACT
CCTCCATCGCTGTGGCGTACATGTCCTACAGAGCGGACATCCCTGTTGATGTCCATGTC
ATGAGCAACGTTGTCTTCTCTATTCTAACGCCTTTGTTAAACCCCATCATTTACACTTTA
CGGAACAAGGAAGTAAAATCTGCTGTTAAAAAGTCTATTTTTCTGAAAATCTTTCCCCT
TTCTAAAAAAATAAATTTATTTGGGTAA
>Loon_OR5B21 (Loon8)
ATGTCTGATGACAACTGCACTGAGGTGACCCAGTTCACCTTCTCGGGACTCACAGAGG
ACCCACAACTGAAGCCCATCCTCTGCACCCGCTTCCTGGGCACCTATGTGCTGACGTTA
GCGGGAAACCTCGGCCTGATCGCCCTGATCAGGGTCAGCCCCCAGCTTCACACCCCCA
TGTACTTTTTCCTCGGTAACTTGTCCATCTTAGATGTTTTCTACACCTCCACCATCAGCC
CCAAAATGCTGCTGGACCTTCCAACAAAAACCAAAGCCATTTCTTTTGCTGGATGTCT
CACGCAGTTTTATTTCTATGCTAGTTTTGCCACAGCTGAGCTTTACCTGCTGGCTGCCAT
GGCCTACGACCGGTATATGGCCATCTCCAAGCCCCTGCTCTATGAGGTTGTCATGTCTC
CTGGCGTCTGCGTGAGCCTGGTCGCTGTGTCCTACCTTGTGGGGTTGCTGAATGCTATC
GTGCACACAAGCGCCTTGCTCTGGCTGTCCTTCTGCGGCCCCCCAGTCATCAACAACT
TCTGCTGCGACGGGCCACCGCTGTTTGTTGTCGCCTCGACTGACACGCGCCTCAATGA
GGGTTTAATGTTTGTGCTCGTGGGTTTCAACATGATGGCCACCAACCTGCTCATCCTCA
CCTCCTACGCCTGCATCGTGGTGGCCGTGGGCAGGATGGGCTCTGCAGCAGGCAGGCG
CAAAGCCTGCTCCACCTGCGCCTCCCACCTGGCAGCCGTCATCATTTTCGATGTGTCTG
CTACTTTTAATTACATGCAACCCAGTTCATCAAACTCACCGGAGAGCAAGAAAATCGCA
TCCATCTTTTACACCATTATAGTCCCCATGCTGAACCCCATGATTTACAGCCTCAGAAAC
AAGGAGGTGAAGCATGCCCTAACCGATTTTATCAGAAGGAAAATGTACTTCTGA
>Loon_OR52K1 (Loon9)
ATGCCCCACTCACGTCCCACCAACACCTCGCTGTCGGAGCTGCACCTGATGGGCATCC
CGGGGCTGCAGCACCTGCATCGCTGGATCTCCATCCCCTTCTGCATCATGTACCTCATC
ACGCTGGCTGGGAACATCACCCTCCTGTGCGTGATAAAGGCTGATCCCAGCCTGCACC
TGCCCATGTTCCTCTTCTTGTCCATGCTGGCTGTCATTGACTTAGTGATGTCCACCTCCA
TCACCCCCAAAATGCTAGGCATCTTCTGGTTTCACTCCACTGCCATCAGCCTGGATGCT

TGTCTCATCCAGATGTACTTTGTTCACGCTTTCTCTGTGATGGAGTCGGGGGTGCTGGT
GGCGATGGCCTTTGACCGCTACATGGCCATTTGCAATCCACTGCGGTACTTGTCCATCC
TGACCAGCCCCGTTGTGGCTGCCATTGGTTTGGCTACCTTGCTCAGGGCTGTGGTTTTC
ATGAGCCCCCTCACCTTCCAGATTCGCCGCCTGCTTCTCTGCAGCCCGGCAGTCGTGG
ACCACTCGTACTGTGAGCACATGGCCGTGCTCAAACTGGCTTGCGGGGACGCTGCCTT
CAGCAACACCTACAGCCTCTCTGTCTCCACCTATGTGGGCACCTTTGACTCGCTGCTCA
TCGCCCTCTCGTACGCGCTCATCCTCCAAGCTGTGCTCAACCTCTCCTCCCCTCAAGCC
TGCAAAAAAGCCTTCAGCACCTGCGGCTCACACCTCTGTGTAATGGCCCTCTTCTACAT
CCCTGGGCTGCTCTCCATGTACATGGAGAGGTACCACCAGGAGCTCCCACCCCATGTT
CAAGTCCTGTTGGCTGATCTCTACCTCCTCATCCCACCGGCATTCAACCCCCTGATCTA
CGGCATCAGGATGAAGCAGATTCGTGATGGAGCACGCAGGGCCATCTCCCGGAGGAG
ACCCATGGCAGGAGCGGTTGGGCCTGGCCTTCAGGGCAGAGGGCTGGGACTCATGAA
GACAAAGTCCATTCCCTAG
>Loon_OR4S2 (Loon10)
ATGGAGAATGCAAGCAGTGTGAAGGAATTCATTCTTCTGGGCCTCTCAGAGAATCAAA
GGGTGCAGAAAATATGTTTTGTGATGTTTCTGTTGTTCTATATGATTATTGTGGCAGGAA
ATCTGCTCATTGTTATCACTGTAATTAGCAGTCAACATCTGAACTCCCCCATGTATTTCTT
CCTTTCTTACCTGTCCTTTGTAGACATCTGTTACTCTTCCATCACAGCTCCTAAAATGAT
TGCAGACTTCCTTGTTGAAAATAAAACCATCTCCTTTGTGGGTTGCATAGCACAGCTGT
TTGGGGTACATTTCTTTGGCTGCACAGAGATCTTCATCCTCACAGTGATGGCCTACGAT
CGCTACATTGCCATCTGCAGACCTCTCCACTACACCAACCTCATGACCAGGCGTACGTG
TGGCCGGATGGTGATGGGCTCGTGGGTAGGAGGCTTTGTGCACTCCATAGTGCAGACT
CTTCTGACCACTCAGCTCCCATTCTGTGGCCCAAACAAGATTGACCACTACTTCTGTGA
TGTCCACCCCCTACTACAACTGGCCTGTACCGACACCTACGCTGTGGGCATCATTGTCG
TTGCCAACAGTGGAATGATAACTCTGAGCTGTTTCTTCATCCTTGTCGTGTCCTATGTTG
TCATCCTGGGTTCCTTGAAAAGTCAAACATCCGAAGGGCGGCACAAGGCCCTCTCCAC
CTGTGGGTCCCACATCACTGTGGTGATTCTGTTCTTTGGGCCATGCACGTTCATCTACAT
ACGTCCATCCAGCAATCTGTCGGAGGACAAGAGCGTGGCAGTGTTTTACACGCTCATC
ACACCCATGCTGAACCCACTCATCTACACGCTAAGAAATGAGGAGGTGAAGAGTGCCA
TGAGAAAACTGTGGAGTAGGAAAGTGGGAAATGAAAACGGAAAGGTGTAG
>Loon_OR6B1 (Loon11)
ATGGTTCTCAGCATGCCCTCAGATAACCTGACACATGTGGTTGAATTCATTCTGGTTGG
TTTTCCAGGTAAACAGGAAATCAAGCTTCTGTTCTTTTTTATGTTCTTCCTGGCTTATGT
GCTGACAGTGACAGAAAATGCAATGATTGTTGCACTTGTTTGGACAAATCTCCAGCTT
CACAAGCCAATGTATGTTTTCCTGGGCAATCTTTCCTTTCTGGAGATTTGGTACATCTCT
GTTACAATGCCGAAAGTGCTTGTGAGCTTGGTTACAAAGAAACAAGGCATCTCTTTCA
CAAGCTGCATGGCTAAGCTATTCTTCTTCCTGGCATTGGCCTGCAGTGAGTGCACTCTC
TTGGCTGTCATGGCCTATGATCGCTATGTGGCCATCTGCAACCCATTGCATTACCCAGTC
ATCATGGATCACACTCCTTGTGCCTGTCTGCCCATTGGCTCCTGGATGAGTGGCTTCCT
GATTTCCACAGGGAAGGTTTACTTCATTTCACGTCAGACCTACTGTGGGCCCAACATCA
TCGACCACTTCTTTTGTGATGTCTCCCCCTTACTGAAGCTAGCCTGTACCAACATGTCA
GTAGCTGAGCTTATGGATTTCTTACTGGCCGTACTCATCCTTGTTGTACCACTCATTGTG
ATTATGGCCTCCTATGTATGCACCATCTCTACTGTCTTGGGCATCCCCTCAGCCCAGGGG
CGTCACAAGGCCTTCTCTACCTCTGCCTCTCACCTTTTAGTGGTCACAGAGTTCTATAC

AGCCTCCCTGTTTATCTATGCCAGGCCCCAGCCTATTGATTCCTTCAGTTCCTACAAACT
GGTTTCTGTGGTACACACTGTCCTGACACCCCTCATCAACCCGGTCATCTATTGCCTAA
GGAACCAGGAATTCAAAATTGCTTTTAGGAAAACAATATACTGGAGAGACATCTTGTC
CTAG
>Loon_OR5AP2 (Loon12)
ATGGCAGAAGGGAATCATACTGTGGTGACTGAGTTTATCTTGTTGGGATTCACAGACAA
TCTGAAGTTGCAGGTCCTTCTCTTCATGGTGTTTCTACTGATCTACCTGTTGACCCTAGT
GGGAAATCTGGGGATGATTGTGCTCATCCGGATTGACTGCAAGCTCCACACCCCCATGT
ACTTCTTCATTAGCAGCCTTTCCTTCTTAGATGTCAGCTACTCCACCATCATTATACCTAG
CACACTCATGACCTTTGTGGCTGAGACAAAAGTCATATCCTACACAGCATGCACAGCT
CAGCTCTTCCTATTCTGCATCGCAGTGACAGCAGAGTGCTACCTCCTGGCCGTGATGGC
ATATGACCGTTTTATAGCCATCTGCAACCCCTTGCTCTACACTGTCATTATGTCCAAGAG
GTTCTGCGTGCTCCTTGTGTGTGGCTCCTACTTCATGAGCTGCATGAATGCAACTATTC
AGACATTATTTATTTTCCACCTGTCCTTCTGCAATTCCAACATCATCAATCATTTCTTCTG
CGATGTGCCTCCCATCCTGAAGCTCTCCTGCTCTGACACTTATATCGCTGACCTGGTCCA
TTTCACCTGTGCTACTGTGCTCGTCATCTCAACCATGCTCCTCATTCTTATCTCTTATGTC
TGCATTGGGTTTACCATCCACAAGATTAAATCCGCCAAAGGCAGATGCAAAGCCTTTTC
CACCTGTGCTTCCCACATGAGTGCTGTCACCATGTTCTATGGGACAGGCTCCTTCATGT
ACCTACGGCCCAGTTCAAAATACTCTGTGGAGCACGACAAGATCATCTCTGTGTTTTAT
ACTCTGGCAATTCCCATGCTGAACCCTATGATTTATAGTCTGAGGAACAAGGAAGTCAA
AGAAGCCCTTCGAAGGATAGTAGCAAGGCTCTATTACTCTCCATGTGTACCACAGAGAT
TCAGGAGTCCAAGCAGGACTGAGAGAACCTGA
>Loon_OR10A7 (Loon13)
ATGAAGCCAACAGAGGGACCAGAACCAGGAAACCACACTCTGCTGACCAAATTCGTC
CTCTCTGGATTGTCCAGCCACCCAGAACTGCAGCACTTGCTGTTCTTCACGTTCTGCTT
GACTTACACCATTACCATCATTGGGAACCTTCTCATCTTCATGGCCACAGTGCACCCCA
CCCTCCGCATGCCCATGTACTTCTTCCTCCGGGTCCTGTCCTTCCTGGATATTTCCACAG
CATCGGTTGTTGTTCCCAAGATGCTGGTAAACTTCCTGTCAGAGGACAGGAGCATTTCC
TACATGGGCTGTGCCACACAGCTCTACTGTGTGATTTTTTTAGGAGCCACTGAATGCTA
CCTTTTGGCAGCCATGGCCTATGACCGTTACGTGGCCATATGCAACCCCCTTAGATATGC
AATCATCATGAACAGCAGAGTTTGTCTTTCCTTGGTCCTGCTGTCATGCTGCAGTGGTA
ATATTGTGTCTGTGGTGCAGACAGCTTGGGTGTTCACATTGCTGTTTTGTGGGCCCAAG
AAGATTAACTACTACTTCTGTGATATTCCCCCACTCATTACGCTCTCCTGCACTGACACC
TCCTTGTATGAAAAGCAGATCATTACAGCCACAGTGCTGGTCATCTTTACACCATTTTGT
CTCATCCTAGTATCCTATGCCTGCATCATCTCCAGCATCCTGAAGATTTCCTCTGCAGAG
GGCAGACACAAGACCTTCTCCACCTGTTCCTCACACCTTATTGTTGTAACACTGTATTA
TGCAAGTGGGACCTTGATTTACTTACGGCCAAAATCCAGTAATTCACAAGACACTAAG
AAAGTCCTAGCTCTCATATACACAACCATAATTCCTACATTAAACCCCCTGATTTACAGC
CTTAGAAATAAAGAAGTGAAAGGAGTACTAATCAAAATTATAGCTGAGCTAAGGAATAT
GTAA
>Loon_OR5AR1 (Loon14)
ATGGTCAGAAGAAACAAGACAACTGTTGATGAGTTCATTCTCTTGGGAATCACAGATA
TTTGGGAGCTGCAGGTCATTCTCTTTGTGCTGTTCCTTCTGATATGTGTCACCTCGTTGG
TGGGGAATCTCAGCATGATTGCATTAATCAGGCTTGACTCTCGACTCCATACCCCCATGT

ACTTCTTTCTCTGCCACCTCTCCCTGGTAGACCTGGGTAATTCCTCAGCAGTTGCTCCC
AAAATGCTAGTGAGCTTCTTTGAAGAAAGGAAAGCCATCTCTCTGCCAGGGTGTGCAG
CCCAGATGTACTTTTGTGGAGTCTGCATAATCAGCGAGTGTTACCTGCTGGCTGCGATG
GCCTATGACCGGTACGTGGCCATCTGTAACCCTCTGCGCTACGTGGCCACCATGTCTCA
AAAGGTTTGTGTCCAACTGGTGGTGGGATCCTACATAATAGCTACTGTGAGTGAAATAG
TGCTTGTCAGCTCAGTGTTCAGTTTACACTTCTGTGGTCCTAATGTCATCAATCACTTCT
TCTGTGACATTCCTCCGCTCCTGAAACTTTCCTGCTCCAGTACCACTATCAATGAACAC
ATGCTTTTCACCATTGCTACTTTCATTGCACTCAGCACTTTAGCATTCATTGTTGTCTCTT
ACGGTTATATCCTTACCACTGTCCTGAGGATCCGCTCCTCAGAGGGCAGGCACAAAGC
TTTCTCCACCTGTGCCTCACATTTGACATCAGTCTCAGTTTTTATGGGACTATGATCTT
CATGTACCTCCGCCCCAGTTCTAGCTACTCTCTGGACCAGGACAAAGTGGTGTCTGTG
GTCTACACCTTGGTGATTCCCATGCTGAACCCCCTAATCTACAGCCTGAGGAACATGGA
GGTGAAGGATGCTCTCAAGAGACTCCTAGAAAAAGTTCTTGTTTCCTTTAGAAATCAA
ACTGGTAAAGAGGTGTCAAACTAA
>Loon_OR10K2 (Loon15)
ATGGAGAGAGACAATAAGAGCACTGATACAACCATGGAGTTCCTCCTGCTCGGCTTCT
CTGAGCTGCTCTGTCTACGCGTCTTCCTCTTCCTTATCTTTCTCATTGTCCATTTGGTCA
CGTTGGCAGGGAATGTGATGATCTTCATGGCAGTGGTTATGGAGCCTTCCCGTCCTCCC
ATGCTTTTCTTCCTCTGTCATCTCTCTGTCATCGAGCTCTGCTATACCTTAGTCATTGTCC
CTAAGGCACTCCTCAGCCTGATTGTGGTGGAGGGCAGCACCATTTCTTTCATAGGCTGT
GCTGCGCAGATGCACCTTTTTGTGGCACTCGGTGGGGCCGAATGCTTCCTCCTGGTAG
CCATGTCGTACGACCGTTACGTTGCCATCTGTCAGCCACTTCACTACGTAGCTGTGATG
AGTGAGGGGCTCTGCCTCAGGCTGGCTGCGGCATGCTGTCTGGGAGGCTTTGCTGTTG
CCCTGGGGTTGACGGTGGCCGTTTTCCGCTTACCGTTCTGTCAGTCGCATCACATCAAC
CACTTCTTCTGTGACGTCCCTGCTGTGCTGCACTTGGCCTGCACACAGAGTTACGCCCC
TGAGCTGCCCTTGCTGGCTGCCTGCGTGCTCCTCCTGCTGCTCCCCTTCCTCCTCATCC
TGACCTCCTATGTCTGCATCGCTGCTGCTTTGTTACATGTCACCTCCTCCGTGGGAAGG
GGCAAGGCCTTTTCCACCTGCATTTCACACTTGGCCATCACCTTGCTACACTATGGATG
TGCCACCTTCATGTACATTCGTCCTCAGTCCAGTTACTCACCAGCTCGAGACAAGATGG
TGTCTCTGGTCTACACCAACATTACTCCGTTACTGTATCCCCTCATTTATAGCCTGAGGA
ACAAGGAAATCAGAGGGGTCCTCAGGAAAATGTTGAGGAGGAAGAAAATAGCTCAGC
TGAACTGGGGTACTACCAGAGCTGTGATGTGCGTGTGTGGTAAATTTTAG
>Loon_OR6Q1 (Loon16)
ATGGTTTCTGACAACAGGACTTTGGTAACTGAATTCATCATCCTAGGATTTCCCAATGT
CAGGGAAGTCGAGCTGGTTGTCTTTGCTACCTTCCTTCTACTGTACTCACTGACCTTCA
CAGAGAATGTGGCCATCATCTTGGTCATTGGCATGGACTACCGCCTTCATACTCCAATGT
ACTTTTTTATCAGCAATCTCTCCTTTCTAGACATTGGCTACACTACTGTGACGGTGCCCA
AGATGCTGGCTAACATTGCTATTCAGTCACAGACCATCTCTATCACTGGCTGCTTCACA
CAGTTGTACATCTTCTTCTTCCTTGGCTCAGCTGAATGCTTCCTACTGACTAGCATGGCC
TATGATCGTTACCTGGCCATTTGCAATCCATTGCATTACACCACCATTATGAATCACAGA
GCTTGTTTATGGCTTGCTCTGGGGTCTTGGTTGGGTGGCTTCCTTGCACCTGCCCTTCC
CACTGCCTTCATCTTCCGACTGCCTTTCTGTGGTTCCAACATCATCAACCACTTCTTCTG
TGACTCACCACCTTTGATGGAGCTCTCGTGCCAAGACATCTTTGAAATCGAAGTCATCA
ACTTTGTGGTGGGCACAATAGTACTAATGAGCTCCTTCATCCTCACCATGATCTCCTACA

TCTTCATTGTGGCCACCATCTTGCACATACCCACAGCTGAAGGACGCAGCAAAGTCTT
CTCTACCTGTGCCTCCCATCTGACCATTGTCACCATTTTCTACGGGACCACCATCTTTAT
GTACATTCGCACCAAGACCATCCAGACTTTTGATTTCAACAAGACTGTCTCTGTCTTTT
ACTCTGTGGTTACCCCAGTTCTGAACCCTGTCATCTATAGCCTAAGGAACAATGACATC
AAGCAAGGCATGAGGAAAGTACTTCTGAAGCAAAAGGACACTTTCTTCATGTAA
>Loon_OR2A2 (Loon17)
ATGCAGAATGAAACATCTGTCACAGAATTCATCCTCCTGGGCTTCTCCAGCAACCCAA
CCCTGCGGCTCTGCCTCTTCGGTGTTTTCTCTCTCCTCTACTCTGCCACTCTGATGGGA
AACGCACTTGTCTTTGTGCTTATCTGCCTGGACTGCCGCCTCCACAGCCCCATGTACTT
CTTCCTCTGCCACCTCTCCATCGTGGACATCTGCTACGCCTCCAACAATGTCCCCCATAT
GCTGAGGAACCTCCTTGGACAAGGCAGAACTATCTCCTTTGCTGGGTGCGGTACACAG
ATACATCTTTATTTAATCTTTGCGCTTACAGAGTGCGTGCTGCTGGCCGTGATGTCTTAC
GATCGCTATGTGGCAATCTGCCATCCCCTCCGCTATGCCCTTATCATGAACCGGAGGGT
GTGCCTCACCCTTGCAGCAGTTTCATGGGCTTTTGGGTTCCTATTTGGTACACTACAAG
CCTCTCTGGCTTTACACCTGCCTTTCTGCGGCCCTTGCGAGGTTGACCACTTCTTTTGT
GAAATCCTTGCTGTCTTAAAGCTGGCCTGCACTGACACTACTGCCAATAAAGTCCTGAT
CTTTGCTGTTTGCGTGTGCTTCCTCCTCTTCCCTTTAGCCTTAATCCTAATTTCCTACCTG
CACATTCTGGCCACCATTCTGCGCATCCGCTCTGCGGCAGGATGGCACAAAACCTTCTC
CACCTGTGGCTCTCACCTGACCGTGGTGGGTCTGTTTTATGGAAACGCCATCTTCATGT
ACATGGGGCCCGGGAGTGGTAACTCATCTGGGAGGGAGAAAGTTCTTTCCCTTTTTTA
CAGTCTTGTCAGCCCAAGTTTGAACCCCCTGATTTACAGTCTGAGGAACAAGCAGGTG
AAGGAAGCCTTGCTGAAGCTTCAAAGAAGGAAAAGAGTTTTTCAGTCTGTGTAG
>Loon_OR52R1 (Loon18)
ATGTTTCTGAACTCTACTGCCTTCTCCCACCCTCCCTATTTCCTACTCGTTGGCATCCCT
GGGCTGGAGAAGGAGCAGTTCTGGATTGCCTTCCCCTTCTGCATCATGTATGCCATTGT
TGTGCTGGGGAACATCACCCTTCTCCTCATTATAAAGACAGAGCCGAGCCTGCACGAG
CCCATGTACCTCTTCCTGGCCATGCTGGCCTTCACTGACCTGGTCCTATCAACATCCATG
TTACCCAAAATGCTTGGCATCTTCTGGCTGGGCTCTGGGGAGATTGGGTTTCTCTCCTG
TCTTGCTCAGTTGTTCTTCATCCATACCTTCTCGTCGGTGGAGTCGGGCGTGCTCATGG
CCATGGCCTTGGATCGCTACATCGCTATTTGCTGCCCTCTGCGGCACACCAGCATCCTC
TCTGCACCGGTGGTGGTGGCCCTCGGCAGCCTGGTGCTGGCGCGTGGGGTCCTCCTGG
TGAGTCCCTTCTGCTTCCTTCTCCGCAGGATGCCCTTCTGCCAGCATCACATTATCTCCC
ACTCCTACTGCGAGCACATGGCCGTGGTGAAGCTGGCATGTGGGGACACCAGAGCCA
ATGTCATTTATGGCCTCTTTGTGGCTTTCATAGTGACAGGATCGGACATGATCCTGATCT
CTGTGTCCTACACCATGATCCTGCGGGTGGTAATGAGGCTGTCATCCACAGAGGCACG
ACGTAAAGCCTTCAGCACTTGTGCATCCCATGTCTGTGTCATCCTTGCCTTTTATGTCCC
TGCCCTCTTCACGTTCCTCACCCACCGGTTTGGGCAGAACATCCCTCCCCACATCCATA
TAATGGTGGCCAATCTCTACCTGCTGGTACCCCCCATGTTAAACCCCATTGTTTATGGGG
TGAGAACCAAGAAGCTCTGGGACAGGGTGGTCCTCCTCTTCCAGTGA
>Loon_OR52B2 (Loon19)
ATGCCACTATTCAACCTTACCAGCTTGAGGCCAGCAACATTCAACCTGGCTGGCATCCC
AGGCATGGAGAAGTTGCACATCTGGATCTCGATCCCGTTCTGCTCCATGTATCTGGCGT
CCCTGCTAGGAAATGGTGTCCTGTTGTTTGTCATAAGGACAGAACGTAGCCTCCACCA
GCCCATGTACCTCTTCCTGTCCATGCTGGCCATCGCTGACCTGATGCTGTCAACCACGA

CTCTGCCCAAGATGCTGGCTCTGTTCTGGTTCAGCGCTGGGGAGATTTCCTTCAGTGGC
TGCCTGACCCAGATGTTCTTCCTGCATTTCAGCTTCTCGGCAGAGTCGGTGATCCTGCT
GGCCATGGCATTTGATCGGTTTGTGGCCATCTGTTATCCCCTGCAGTATGCGGCAATGCT
GACCCAGTCAGCCGTTGTCAAAACCGGGATGGTGGCTTTGCTGAGAAGTTTCTGCATA
ATTTTTCCGTGTATATTTCTTTTGAAAAGGCTGCCGTTCTGCGGGCACAACGTCATCCC
GCACACCTACTGTGAGCACATGGGCATCGCCCGGCTGGCCTGTGCCGACATCTCCATC
AATATCTTGTATGGTCTCGCAGTGCCTTTTGCAGCAATAGTGGTAGATGTTGTACTCATT
GCTGTCTCCTATGTCTTAATTCTTCTGGCATTATTCAGACTCCCTTCCAGGACTGCCCGT
CACAAGGCTTTCAACACCTGTGGCTCTCATATCTGTGTTATATTACTTTTCTATATTCCTG
CTTTTTTCACCATTTTAACACACCGCTTTGGTCGGGAAATCCCCCACCACGTCCACATT
CTGCTGGCCAACCTGTACGTGCTCTTCCCCCCACTGCTAAACCCCATCGTCTATGGTGT
GCGAACACAAGAGATAAGAGAGAAGGTTGTGAAAGTGTTCATCTGCCCCAAGAGTCA
TTTGCTGCAAGAATAA
>Loon_OR14J1 (Loon20)
ATGACCAACAGCAGCTCCATTACTGAATTCCTCCTCCTGGCATTTGCAGACACGCAGG
AGCTGCAGCTCTTGACCTTCTGGATCTTCCTGGGCATCTACCTGGCTGCTCTCTTGGGC
AACGGCCTTATCATTACTGCCATAGCCTGCGCCACCACTCCCATGTACTTCTTCCTCCTC
AACCTCTCTTTTCTTGACCTTGGCTGCATCTCCACCACTCTCCCCAAATCCATGGCCAA
TTCCCTCTGGGACAACAGGGCCATCTCCTACTCCAGATGTGTTACCCAGGTCTTTTTCT
TCTCCCTTTTGATGCCAGCAGAATTTTTTCTTCTCACCATCATGGCCTATGACCGCTATG
TTGCCATCTGCAAACCCCTGCACTACGGGACCCTCCTGGGCAGCAGAGCTTGTGCCAA
CATGGCAGCAGCTGCCTGGGGCAGTGGGTTTCTCACTGCTGTGCTGCACACGGCCAAT
ACATTTTCACTACCGCTCTGCCAAGGCAATGCCCTGGACCAGTTCTTCTGTGAAATCCC
CCAGATCCTCAAGCTCTCCTGCTCAGACTCAGACTACCTCAGCGAGGTTGGGCTTCTT
GTGGTTAGTGCCTTCGTAGCATTTGTGTGTTTTGTCTTCATCGTGCTGTCCTATGTGCAG
ATCTTCAGGGCCGTGCTGAGGTTCCCCTCTGAGCAGGGATGGCACAAAGCCTTTTCCA
CGTGCCTCCCTCACCTGTTCGTGGTCTCCCTGTATGTCAGCACCGGCATGTTTGCCTAC
TTGAAGCACCCCTCCAGCTCCTCCCCATCCCTGGACCTGGTGGTGGCAGTTCTGTACTC
GGTGGTGCCTCCAGCAGTGAACCCCCTCATCTACAGCATGAGGAACCAGGAGCTCAA
GGATGGCATTAGGAAAGTGATTACATGGATGTTTCTCAATAGGGATAAACTTCCCATCT
CTTTCCACAAATGA
>Loon_OR2B2 (Loon21)
ATGGCCAGGGAAAATCAAAGCATAGTGACAGAATTCATCTTTCAAGGCCTTTCCTCCC
AGCCAAGGACACAGACTGTTCTTTTCATAGTGTTCCTGGTTTTTATCTGTTCACAATG
GCTGGGAACATCGTGATCATTACAGTGATCAGAGCTGATTGCCTGTTGCACTCACCCAT
GTACTTTTTCCTTGCCAACCTGTCCTTCTTAGACATCTGCTACATCTCCAGCAACATCCC
CCAGATGCTGGTGAACCTCTTGACCAAGAAGAAGACCATCTCCTTCTCTGGATGTGCT
GCTCAGATGTATTTCTCTCTGGCTTTTGGTGTGACAGAGTGTATTCTGCTTGGGGTCATG
GCTTATGATCGCTATATGGCAATATGTCACCCCTTGCTCTACACCACTGTCATGAACAGG
AAGCTTTGCACTCACATGGTGGTGGCTTCCTGGACCAGCAGCCTGCTGAGCTCTGCGG
TCATCAACAGCCTCACCTTGCGGCTGCCCTTCTGCGGGCCTGACATCTTGAACCATTAC
TTCTGCGAAGTGCCAGCAGTGCTGGCCTTGGCCTGTGCCGACACTGCCCTCGTGGAGT
TAGTCATCTTCATCTTCAGCATCCTCACAGTCTTCCTCCCCTTTCTTCTGATCATCACCTC
CTACGCCCATATCCTTTCTGCCATCTTAAAGATTCAATCTGCACATGTGCAATCCAAGGT

CTTCTCCACCTGTGGATCCCACCTGATGGTGGTAACCATATTCTATGGGACAGCCATCTG
CATGTACATGAATCCTAAGTCAAGGCCTTCACAGGACAGGGACAAAGTGGTTGCAGTG
TTTTACACCATTGTAGCCCCAATGCTGAACCCCCTCATCTACAGCCTCAGGAACAAGGA
CATGAAGCGTGCCCTGAGGAGGGCAATGAATAGACCCAAATCCCTGTTTATTTAA
>Loon_OR10C1 (Loon22)
ATGAAATCGTCTGGAGAAGGGACCACTGAAAATAAAACTTCTGCAATCGTGTTTGTTAT
CCTGGGTTTTTCTCATCGTCCAGCTATGAAAGTCATCTTCTTCATCCTATTTCTATGTATT
TACATCATCACAGTGCTGGGAAACCTAATTATTCTTCTTCTAATTAATATGGACCCTGTC
CTCCACACCCCCATGTACTTCTTCCTCAGGAACTTGTCATTTCTGGAGATCTGCTACAC
CTCTGTCACCCTGCCCAGAGTGCTGGTCAATCTGATCTCAAGTGATACATCCATCTCTTT
TGCAGGCTGTGCTGCACAGATGTATTTCTTTCTGTTCTTTGGGGCAACTGAATGCTGCC
TCTTAGCTGCTATGGCATATGACCGCTACCGAGCGATATGCAACCCTCTGCATTACATGG
ATATCATGAATAAGAAGGTATGCATGCAGCTGGCTGCTTCCTCATGGATATGTGGCAAC
CTTGTGGCCCTTGGGCACACTACATTTATCTTCTCTTTGCCCTTCTGTGGCTCCAATGTG
ATCAACCATTTCTTCTGTGAGATCCAGCCAGTGCTGATGCTGGTGTGTGGGGACACTTA
CTGGAATGAGTTACAGATCATTCTGGCTGCTGTCTTTATAATCCTGATGCCTTTTCTGCT
CATTTTGGTATCCTACAGCCTCATCATTTCCTCCATCCTCAAAATCAGGTCTGCCAAAGG
AAGGTATAAAGCATTCTCCACTTGCTTCTCACATCTCACTGTAGTGACATTATTCTATGG
GACAGCTGTGTTCATCTATATACGTCCCAAATCCAGCTATTCCCTGGATGTGGACAAGG
TGCTCTCTCTGTTCTATTCTGTGGTGACCCCAATATTGAACCCTGTTATCTACAGCCTTA
GGAATAGGGAGGTGAAAGGGGCTCTCTTTAAAATGAGAATGAAGCTATTTCACTCCAA
CTCCTAG
>Loon_OR52B2 (Loon23)
ATGGCGACTCTCAATCAAACCAGCTTGCAGCCTGCCTCCTTCCTTCTGCTGGGCATGGC
AGGCCTGGAGGACCTGCACACCTGGCTCTCCATCCCGTTCTGCCTGTTGTACATCGCA
GCACTCCTTGGCAACTTCATCCTCTTATTTGTCATTGTGACGGAACGAAGCCTGCATGA
GCCAATGTACCTCTTCCTGGCCATGTTAGCAGTGGCAGATCTCGTATTATCCTCCTCCAC
AGTGCCCAAAGCCCTGAGCATATTCTGGCGCCTTTCCAAGGAGACGTCTTTCCACGCC
TGCCTTACCCAGATGTTCTTTACACACCTGAGCTTCATTGCAGAGTCGACCATTCTGCT
GGCCATGGCATTCGACCGGTACGTGGCCATCTGCAACCCTCTGCGATATGCCGCAGTGT
TCACGCACTCGGTGATAGCCAAGGTAGGGCTGGCTGCAATAGCCAGGAGCTTTTGTGT
AATGTTCCCAACAATATTCCTCCTTCAGAGGCTGCCATACTGCAGACACAGCATTATGC
CACACACCTACTGTGAGCACATGGGCATCGCCCGGTTGGCCTGTGCCGACATCTCCGTT
AACATCTGGTACGGCTTTGCCACCACCCTTCTGTCCCCAGGCGTGGACATTGTGCTCAT
CGGGATACCATACATCCTCATTCTCCGAGCTGTCTTCAGGCTCTCGTCCAAGGATGCCC
AGCTCAAGGCAGTTGGCACCTGCAGCTCTCATGCCAGCGTTATATTAATGTTCTACACA
CCAGCATTTTTCTCATTTTTCACTCATCGATTTGGCCACAATGTCCCCCACCATGTTCAC
ATCCTGTTGGCCAATCTCTATGTGCTCCTGCCACCCATGCTAAACCCCATCATCTACACT
ATGAAAAACAAACTGATTCGAGAAAAGGTGTCCCAAGTACTCTTCAGGACTGGGCAA
ATGCGGTGA
>Loon_OR5AP2 (Loon24)
ATGGCTTGGGAAAACTGGACTACGGTGACAGAATTTGTTTTCAAGGAGTTCACGGATT
GTGTCGACCTCCAAGTTACCCTCTTTGTGGTTTTCCTGCTCATCTACGTCATCACCGTG
GTGGGAAACCTTGGCATCATTGCTGCAGTCTGGCTCGATTCCCAGCTTCAAACCCCCAT

GTACTACTTTCTCAGCAACTTGTCCTTCTTGGATCTGTGCTACTCCTCAGCTGTGACAC
CCAAAATGCTGCTGAACTTCTCATCAGAAAGGAAGACTATCTCTTTTGCTGGCTGTTTC
GCACAGCTCTATTTCTACGCTGCTTTTGTAACCGTGGAGTGTTACCTCCTGGCCGTGAT
GGCGTACGATCGCTACGTTGCCATCTGCAACCCGCTGCGTTACCCCGTCGTCATGTCCC
AGAAGGTCTGCATTTCCCTCCTGGCCGGGTCCTATGCGGTGGGGTTTTTTCATTCGGCG
GTGTTCACGGGGTTTGCGCTGAGAGTGTCCTTCTGCGGCCCCAATGTCATTGACCACTT
CTTCTGTGATGGACCACCGCTGTCAAAACTGGCCTGCTCTGATACTCGCCTCAACCAA
GTGTTGCTACTTGCCTTCGGGGGCTTCAACGAGGTCACCACCATATCAGTCATCCTCAT
GTCCTATGGCTGCATCCTGTTCACCGTCCTGAGGATGGGCTCTGTGCCGGGCAAGCGC
AAAGCTTTTGGTACCTGTGCGTCCCACCTGGGGGTCGTCACCATCTTTTACGGGACCCT
TCTCTTCATGTACCTGCGGCCCAGCTCCAGCTACTCCCTGGGCAGGGACAAAATCGTTT
CTGTCTTTTACGCGGTGGTGACCCCCATGCTGAACCCCTTCATCTACAGCCTGAGGAAC
CAGGAGGTGAAGAGCGCTCTGAAGAGAGCAGTGGGGAGAAGAATTATTTCCCTGCTG
AAGTGGTAA
>Loon_OR14A16 (Loon25)
ATGGCCAACAGCAGCTCCGTCACAGAGTTCCTCCTCTTGGCATTTGCAGACACACAGG
AGCTGCAGCTCTTGCACTTCTGGCTCTTCCTGGGCATCTACCCGGCTGCCCTCCTGGGC
AACATCTCCATCATCACAGCTGTAGCCTGCGAACATTGCCTCCACACCCCCATGTACTT
CTTCCTCCTCAATCTCTCCCTCTTTGACTTGGGCTCCATCTCCACCACCGTCCCCCAATC
CATGGCCAATTCCCTGTGGAACACCAAGGCACTCTCCCACTTGGGATGTGTTTCCCCA
GTCTTCTTTCTAGTCTTCATTTTTGGTGCAGAGTATTCCCTTCTCATTGTCATGGCCTTCG
ACCACTATGTTGCCATCTGCAAAACCCTGCACTATGAGACCATCCTGGGCAGCAGAGA
TTGTGTCCACGCTGCCTGGGGCAGGGTGTTTCCCCGTTGTCTGCTGCACACTGCTAATA
CATTTTCACTTCCATTCTGCCAAGGCAATGCCGTGGACCAGTTCTTCTGTGAAATCCCC
CAGATTCTCAAGCTCTCCTGCTCAGACAACTATCTCAGGGGAATTGGGCTTTTTGTATTT
AGTCTTGCTTTGGCTTCGGGGTGTTTTGTTTTCATCATGTTGTCCTATACGCAGATCTTC
AGGGCTGTGCTGAGGATCCCCTCTGAGCAGGGATGGCACAGTGCCTGTTCCATGTGCC
TCCCTCACCTGGCTGTGGTCTCACTCTTTATTAGCAGTTGCTTGGTTGCCTACCTGAAG
CCCTCCTCCATCTCTTCCCCATCCCTGGACCTGGTGGTGGCTCTTCTGTACTTGGTGGT
GCCTCCAGCAGCAAACCCCCTCATTTACAGCATGAGGAACCAGGAGCTCAAGGATGCC
CCGAGGAAACTTATTCAATGGGTTCACCTTCAGCAGCAGTAA
>Loon_OR52I1 (Loon26)
ATGGCTTCTGATCCCTTCAACCGCCCCAACAGCAGCTCCTCCTCTTTCATCCTTGTGGG
TGTCCCCAGCCTGGAAGCTTTCCCCACTTGTCTGGGCATCCTTTTCTGCTCAGGCTATAT
CATCGCCTTGGTAGGAAATGGTGTGGTTTTGCTTGTCATTGGGCTGGACAGCTCCCTGC
GTGACCCCATCCATTGCTTCCTGGGCATGCTGGCGGTCATTGATGTGGTGATGGTGACG
TCCATCGTCCCCAAGATGCTGGGCATATTCTGGCTGAACTCTACTGAGATTGGTTACAT
GACCTGTTTTGTTCAGATGTTCCTTGTCCACTCCACAACATCAGAGGAGTCAGGAGTG
CTCCTGGCCATGGCCGTTGACCGCTACGTTGCAATTTGTCACCCCCTCCGGTATCAAGC
CATCTTGAATCGCCAAACAATTGCCCAAATAGGCCTGGCCATTGTGGTGAGAGCTCTCC
TTTTCATGGTCCCCTTGACATGGATGGTGATGAACCTCCCCTATTGCCGTTCCCGTGTGG
TTCCCCATTCGTACTGCGAGCACATGGCCGTAGCGAAGCTGGCGTGTGCAGACCCCAG
ACCCAGTGGGCTTTACAGTGTGGCTGGGTCCTCTCTTACAGTGGGGATGGATTTGGCTT
TCATTGCTGTGTCCTATGGGATGATCCTTAAAACTGTCCTGGAGAAGAAATCGTGCTGG

AAGGCCTTCAGCACCTGCGGGTGTCATATCTGCGTGATGCTGCTGTATTACATCCCTGG
GATAGTCTCCATTTATGCACAGCAGTTCAGCAGCGGCATATCCACGCATGCACGGGTTC
TGCTAGCTGATCTCTACCTGACTCTCCCCACCATGATGAACCCCATCGTTTACAGCATG
AGGACCAAGCAGATCCGTCAGGCAGTGCTCAAAATGCTGTTTTCCAGGAAGGTTCATG
CCTGA
>Loon_OR14J1 (Loon27)
ATGTCCAACAGCAGCTCCATCACCCAGTTCCTCCTCCTGGCATTCGCAGACACGCGGG
AGCTGCAGCTCTTGCACTTCTGCCTCTTCCTGGGCATCTACCTGGCTGCCCTCCTGGGC
AATGGCCTCATCATCACCACCATAGTCTGCGACCACCACCTCCACAGCCCCATGTACTT
CTTCCTCCTCAATCTCTCCCTCCTCGACCTGGGCTCCATCTCCACCACTGTCCCCAAAG
CCATGGCCAATTCCCTCTGGGACACCAGGGCCATTTCCTATGCAGGATGTGCTGCTCAA
GTATTTCTGTTTCTCTTTTTCACGTCAGCAGAGTTTTACCTTCTCACTTTCATGTCCTAC
GACCGCTACATTGCCATCTGCAAACCCCTGCACTACGGGACCCTCCTGGGCAGCAGAG
CTTGTGTCCACATGGCAGCAGCTGCCTGGGGCAGTGGGTTGCTCACTTCTCTGCTGCAT
ACGGCCAATACATTTTCACTACCACTCTGCCAAGGCAATGCTGTGGACCAGTTCTTCTG
TGAAATCCCACAGATTGTAAATCTCTCCTGCTCAGATGCTTACCTCAGGGAAGTTGGGC
TTCTTGTTTTTACTTTATTTTTTTCTTTTCTTTGTTTTCTTTTCATTGTGCTGTCCTACGTG
GAGATCTTGAGGGCTGTGCTGAGGATCCCCTCTGAGCAGGGACCACATAAAGCCTTTT
CCACGTGCCTCCCTCACCTGGCTGTGGTCTCCCTCTTAATTTGCACGGGCACGTTTGCC
TACCTGAAGCCCCCCTCCATCTCCTCCCCATCCCTGAACCTGCTGGTGTCGTTTCTGTA
CTCGGTGGTGCCTCCAGCAGTGAACCCCCTCATCTACAGCATGAGGAACCAGGAGCTC
AAGGATGCAGTGTGGAAACTGATGACTGGATGA
>Loon_OR4D9 (Loon28)
ATGGAGCAGGAGAACTCTACCACCACTGTGAAAAAATTTTTCCTTGTTGGTTTTACTGA
AAGTGCCATGCTGCAGTATATTCTCTTTACAACCTTCCTCGTAATCTACTCAATGACTTG
GCTTGGAAATGTCACCATCATCACCACAGTAATCACAGATCAACAGCTCCACAAGCCT
TTGTACTTTTTGTTGGGAAATTTAGCCTTCATAGACCTCAGTGAATCCTCAGTGATTCTG
CCCAAGATGCTATGGGACCTCTTGTCTGAGGTTAAGACCATTAGTTTTTCGGGGTGCAT
TACACAGATGTTTTTCTTCCATTTCACAGGAGGTGCTGTGGCCATCCTCCTCATAGTGAT
GGCTCTGGATCGGTATGTGGCTCTCCATAAACCTTTGCAGTATCTAAGCATTATTAATTA
CAACGTTCTCCTCGGGCTGGTAATAGGAGCATGGGTAGGGGGGTTTGTTCATTCTATTG
TGCAGGTAGCACTGGTCATTCAGTTGCCATTCTGTGGACCAAATTTTCTAGACAATTTC
TACTGTGATGTCCCACAGGTAATCAAACTGGCCTGTACAGACATCTATGTGGTTGAGCT
ACTCATGGTGTCCAATAGTGGCCTGCTTACAGTCCTCATCTTCATTGCCCTGATTGTGTC
ATATGCTGTCATCCTGGTCAAAATCAGGACACATATCACAAAGGGGAAGCACAAAGCC
TTTTCCACTTGTGCAGCTCAGGTTGCAGGGGTGATCATCCATTTTGTACCCTGCATCTTC
ATCTATGCACGTCCGTTCCAAAAGCTTGTGGTGGACAAAGCCATGTCATCTCTTTATAC
AGTCATCACTCCAATGCTTAATCCCATGATCTACACACTGAGGAACACGGAGATGAAG
AATGCCATCAACAGATTACTCAACAACATCTTTTTCAGAATGAGAAATTCACAGCTGTC
CTTCCTTTCTTAG
>Loon_OR5B21 (Loon29)
ATGTTGGCTACAGAAAAGCAAAACCAAACCTACATTACAGAATTCATCCTCCTGGGATT
TGGCGATTTACCTGACCTTCAGGTTCCTCTCTTCCTGCTCTTCCTGGTGATCTACATCGT
GACAATGATGGGGAACATTCTCATCCTCGTGGTGGTGGTGGCTAATCAGCATCTCCACA

CCCCAATGTACTTCTTCCTCAGTAACTTGTCCTGCTTGGAGACGTGCTACAGCTCCACC
ATCCTGCCCAAGCTGCTGGCCAGCTTCCTGACAGGCGACAGAAGTATTTCCTACCGCA
GCTGCATGACACAGTTTTTCATATTTGCTTTTCTAGTTGGGGTAGAGTGCTATCTCCTGG
CAGTGATGTCTTACGATCGATACTTAGCAGTATGCAAACCTTTACATTACGTCACGCTGA
TGAATCGTAAACTTTGCATAGCACTAGCTGCAGGGGCCTGGGTAAGTGGTTTTATGATC
AGCTTTGTTATCACACTGTTGATGTCACAAATGGTGTACTGTGATCACCGTGAGATTGA
CCATTTCTTCTGTGATTTCACCCCTGTGATAAAATGCTCCTGCAGTGACACATTTCTGAT
AGAAGTTGTAACAGCTTTGATGTCTGCTACATGTACTATGCTGCCCTTTCTTTTCACTGT
TGCATCCTATATTTTAATCATCATCACCATCCTGAGAATCCCTTCAGCTCCAGGAAGGCA
AAAGGTATTTTCCACATGCTCCTCTCACCTCATTGTGGTCACTATTTTCTATTCAACTCT
CATCCTTGTCTATATGTTACCAAAAACTTACAGCCTCACGGCTTTTAACAAGTTGTTCTC
TGTCTTCTACACTGTGGTGACACCCATGGTGAATCCACTTATCTACACCTTAAGAAACA
AAGAAGTCAGAGGAGCCCTGGGGAAAGAAGTCCAGGCATTTATGGGTTTATATAAATA
A
>Loon_OR9G1 (Loon30)
ATGGAGGAGAGAAACCACAGTTCGGTCACCAGCTTCATCCTCCTGGGGTTCACCACCG
ACCCGAAGCTGCAGCTTCTCTTGTTCATGGTGTTCACCTTAGCCTACGTCACCACCCTC
GTGAGCAACATCACCCTCATCGTGGTTATACTCCATAGCTCACGGCTCCATACCCCAAT
GTACTTCTTCATTGGGAACTTGTCCTTCCTGGATCTCTGGTATTCCTCCGTCTACACCCC
CAAGATCGTGCTGAACTGCATCTCCGAGGACAAGAGCATCTCCTTTGCTGGGTGTGCT
GCTCAGTTCTTCGTCGCCAGCGGCTTGGCCTACACCGAGTGCTACCTGTTGGCGGTGA
TGGCCTATGACCGGTATGTGGCCATCTCCAACCCACTGCTCTACGCTGCTGCCATGTCC
AAGAAACTGTGCACGGGGCTGGTGGCTGCCTCCTACCTCAGCGGCTTTGCCAGCTCCA
CCCTCATTATCAGCTGCATGTTCGCCCTCCGCTTTTGTGACGCCAAAGTCATCGATGAC
TTCTTCTGTGACCTTCCCCCGCTGGTGAAGCTCTCCTGCAACGTGACAGACAGCTACC
AGGCACTCCTGTATTTCATCCTGACCTCCAATATCATCCTCCCCTCCGCGCTCATCCTCA
CGTCCTACGCCTCCATCCTGGCTGCCGTCCTGAAGATGCACTTGGCCGAGGGGCAGCG
CAAAGCGTTCTCCACCTGTGCCACCCACCTCATCACCATCACCCTCTACTACGGCTCCA
TCCTCTTCATTTACGCCCGGCCCAGCTCCACCTATGTCCTGGGAAGGGACAAGGTGGT
CTCTGTGTTTTACACAGTGATGATCCCCATGCTGAACCCCTTCATCTACAGCCTGAGGA
ACCAGGAGGTGAAGGAAGCACTGAAGAGACTGGTGAAGAGACAGACAGCTTCTTAA
>Loon_OR14A16 (Loon31)
ATGTCCAACGGCAGCTCCATCACGCAGTTCCTTCTCCTGGCATTTGCAGACACACGGG
AGCTGCAGCTGTTGCACTTCTGGCTTTTCCTGGGCATCTACCTGGCTGCTCTCCTGGGC
AACGGCCTCATCATCACAGCCATAGCCTGGGACAACCACCTTCATACCCCCATGTACTT
CTTCCTACTCAACCTCTCCCTCCTCGACCTGGGCTCCATCTCCAGCATTGTCCCCAAAT
CCATGGCCAATTCCCTCTGGGAAAAAAGAGCTATCTCTTATCCAGGATGTGCTGCCCAG
GTCTTTTGCTTTCTCTTCTTCATTGTAGGGGAGTATTGTCTTCTCACTGTCATGGCCTATG
ACCGCTACGTTGCAATTTGCAACCCCCTGCACTATGGGACCCTCCTGGGCAGCAGTGC
TTGTGCCAAAATGGCAGCAGCTGCCTGGGGCAGTGGTTTTATCAATGCTGTGCTTCAC
ACTGCCAATACATTTTCAATACCACTCTGTCAAGGCAATGCTGTGGACCAGTTCTTCTG
TGAAATCCCCCAGATTCTCAAGCTCTCCTGTTCAGACTCTTACCTCAGGGAAGTTGGGC
TTCTTGTGGTTAGTCTTTCTGTAGCTTTAGGGTGTTTTGTTTTCATTGTGCTGTCCTACTT
GCAGATCTTCAGGGCTGTGCTGAGGATCCCATCTGAGCAAGGAAGGCACAAAGCCTTT

TCCATGTGCCTCCCTCACCTGGCTGTGGTCTCCCTGTTTGCTAGCACTGCCATGGTTGC
CTACCTAAATCCCCCTTCCATCTCCTCCCCATCCCTGGATCTGGTGGTGACAGTCCTGTA
CTCAGTGGTGCCTCCAGCAGTGAACCCCCTCATCTACAGCATGAGGAACCAGGAGCTC
AAAGACACACTGAAGAAGCTCATTCAATCAGCTGCCCGTGTCTCTTGA
>Loon_OR10A7 (Loon32)
ATGGAAAATGCAGTCGGGGGCAACCACTCATCCATCACAGAAGTCACCCTCCTGGGAT
TCAGCCATCGTCCTGGGCTGCAGGTTCCTCTCTTCCTGCTTTTCCTGATGATCTACCTCA
TGACCATGGCTGCAAACATCTCCATCGTTGTGCTGGTGGTGGCTGACCGGCACTTTCAT
ACCCCCATGTACTTCTTCCTGGGCAACTTGTCCTGCCTAGAGGTCTGCTACAGCTCCAC
CATCCTGCCCAGGCTGCTGGCCAGCTTCCTGACGGGGGACAGGGCCATCTCCATCCGC
TGCTGCACCCTGCAATTCTACTTTTTTGGTTCTCTGACAGCAGTAGAGTGCTGTCTCCT
GGCTGTGATGTCGCTGGACCGCTACATGGCCATCTGCAGACCGCTGCACTACGCAGCC
TTGATGAGGGTCAGGTTCTGCCTGCCCCTGGCAGCTGGGTCGTGGATCAGTGGGTTCC
TGTCCACCATCGCCAACACCTACTGGATGTCAGCCATGGACTTATGTGGTCCCACTGAC
CTCGACCATTTCTTCTGTGATTTCTCCTCACTGTTAAAGCTTTCCTGCTCTGACACCAGC
CTGATGGCTCTGGTGACATTGCTGCTTTCTGCTTTGTTCACTCTGCCCCCCTTCCTCCTC
ACCCTCAGCTCCTACATTTGCATCATTGCGGCCATCCTGAGAATCCCATCCACCGTTGG
CCGGCACAAAGCCTTCTCCACCTGCTCCTCCCACCTCATTGTGGTTACCCTCTACTACG
GAACCATCATCTTTGTGTATGTCCTCCCAGATACTGACACGCTGAGGCCCCTGAACAAA
CTCTTCTCTCTCTTTTACACCGTCATCACTCCTTTTGCTAATCCCTTGATCTACAGCCTG
AGAAACCATGAGGTCAGGGAGACCCTGACGAGAGCTTTCACTGCTAAGCTCTGCACT
GCCTAG
>Loon_OR51G2 (Loon33)
ATGGAGCACGACTCACATACCACATGGGAATTCAATGGCTCCTTCTATCAGCCTTCAGC
TTTCCTCCTGATGGGCATCCCAGGCCTGGAAGCCCTTCACCTCTGGATCTCCATCCCTT
TCTGTGCGCTGTACCTTATTGCTCTCTTGGGAAACTGCATGATCCTCTTCATCATAAAGA
AGACCCAAAGTCTTCACGAACCAATGTACTACTTCCTCTCCATGTTGGCACTCACTGAC
CTGGGCTTGGTTCTATGTACGCTGCCTACTACTCTGGGCATTTTTTGGTTTAATATGCGA
AGGATTGGGTTTGATGCTTGCCTCACTCAGATGTACTTCATCCACATACTGTCCTTCATC
GAATCCTCTGTGCTCCTGGCAATGGCATTTGACCGCTATATTGCCATCTCCCATCCATTG
AGACACCCATCCATACTGACCAAGATGACTGTCATAAAAATAGGTCTGGCAATCATATT
GAGAGGTATGGTCTCCCTCCTTCCCATACCCTTCTTGCTCAAGAGACTAACCTATTGTG
GGAAGACCGAGCTTTCTCATTCTTTTTGCTTCCATCCTGATATCATGAACCTAGCATGTG
CAGATATAAAAGTCAATGTCTTCTACGGTATGATTATTCTCTTATCAACTGTGGGGATGG
ACTTCATCTTCATTGTGCTGTCTTACATCCTGATCATTAAAACTGTTATTGGCCTTGCAA
CCAAGGAGGAGTGTCTCAAGGCTTTGAATACATGTGTCTCCCACATTTGTGCTGTTCTA
GTGTTCTTCATCCCAATGATCGGACTGTCCATGATCCATCGCTTTGGAAAGAATGTTCCT
CCTCTGGTTAACACTTTGGTGGCTTACACCTACCTTATAATTCCCCCCGCTCTGAACCCC
ATTATCTACAGCATAAAATCCAGCCACATCCGTGAGGCTTTGCTCAGGGCACTGCGGAG
GAAGACTGAATCTGACTGGTAG
>Loon_OR5AR1 (Loon34)
ATGGCAGAGCAGAATCACACCTCGGTGGCAGAGTTCATTCTCGAGGGCTTGAGTGACC
AAGCGGAGATGGAGGCAGTCCTCTTTGTCCTGTTGCTGCTCATCTACACCGTCACCCTT
TTGGGCAACGCGGGGATTATCGTAGTCATCCGAGGTGACCCACGGCTCCACACATCCA

TGTACTTCTTCCTTGGCAGCCTCTCTGTTGTTGATATCTGCTTCTCCTCTGTGATTGCCC
CCAGGACCTTGGTGAACTTCCTATCAGAGAGGAAGATCATTTCCTTCGCTGGCTGCATG
GGCCAAGCCGCCTTCTACATCGTCTTCGTGACGACTGAGTGTTTCCTGCTGGCCGTCAT
GGCATACGACCGGTACGTGGCCATCTGTAACCCCCTGCTCTATTCCTCTGTTATGACTCG
GAGGTTGTGCATGTGGCTGGTGGTGGGGTCCTACATCGGGGGTGTCCTGAACTCCATC
ATACAGATGATCTTCATCATTAGGCTGCCCTTCTGCAGCTCCAATGTCATCAACCACTTC
TTCTGCGACGTTCCTCCCCTCCTGGCTCTGTCCTGTGCCAGCACCTACGTCAACGAGAT
GATCCTCTTCTCCTTGGCTGGCGTCACTGAGCTCAGCACCATCTCCACCATCCTGGTCT
CCTACATCTTCATCTCCTCTGCCATCCTGAGGATCCGTTCAGCTGAAGGCAGGCAAAAA
GCCTTCTCCACCTGCGCGTCCCACCTGACAGTGGTGACCATGTTGTATGGACGACAA
TCTTCATGTATTTACGCCCCAGCTCTAGTTACTCCCTGAACACTGACAAAGTGGTCTCC
GTCTTCTACACAGTGGTGATTCCCATGCTGAACCCCCTCATCTACAGCCTGAGGAACAA
GGAGGTGAAGGATGCTCTGAGGAGAACAGCAGAGAGAATCACAGTCAGGCTCTGA
>Loon_OR5V1 (Loon35)
ATGGATAACCAAACAAGTGGAACTGAGTTTGCTCTCTTGGGATTTACAAGAGATCCCC
TCCTTCAGAGCCTCCTATTCACTGTGTTTTTAATCATTTACCTAATCATTTTATTAGGAAA
TATGGTAATCATGATGGTCATAAAGACTGATCTGCATCTTCACTCTCCTATGTACTTCTTC
CTCTTTCATTTAGCCCTTGTTGACATCTGTTATGCCACTGCTGTTGTTCCAAAGTTGCTG
GTGAATTTTCTTGTGAAAGGAACCATAACTATTGCTGTCAGTGCTTGTGTGACTCAAAT
GTTCTTCATTTTCCTCTCAGCTGGGTGTGAAGTTTTTATGCTTTCAGTAATGGCATATGA
CCGATATGTGGCGATCTGTAATCCACTGAAGTATGAAGAGGCTATGAGCAAACATTTCT
GTTATCAGCTGGTGGGAAGTGCATGGACAATGGGTCTCATATATTCAATTCTAAACACG
ATTCCTGTGTTAAATGTTCGATTTTGTGTGCACGCAGAAATTAAACATTTCAGCTGTGA
GCTGCCCCCTCTCCTATCTGCTGCTTGCAGTGGGATATTTCTCAGTAAACTCATTCTTCT
TTTTTCTGCTGTGATCTTTGGGTCCAGCTCCTTTTTGCTCACCCTCATCTCCTACATCTAT
ATCATCGCAACTGTCTTGAAGATACAGTCTGCAGAAGGGAGGCACAAAGCTTTCTCCA
CTTGCAGCTCCCACTTCATTGTAGTGGGGTTACTCTACATAACTGCTCTCTTCCAATACA
CAAAACCCAAATCAGTGTCATCAATCATTCTAGACCAAGTGTTGTCTACCCAGTACAGC
ATCTTAACCCCCATGCTGAATCCTATTATCTACAGCTTGAAGAATAAAGATGTGAAAAG
AGCCTTAGGCAGAACTGTAAAGAAACTTCAATTTGTGTAA
>Loon_OR4S2 (Loon36)
ATGGTGTCTGTGGAGAACAAATCCAATGTGACGGAGTTCATCCTGCGTGGACTGACAC
AAGATAAGACAGTAGCAAAAGTGTGCTTCTCGTTATTCTTAGTCTTCTATGCCACTACC
ATTCTTGGAAACCTGCTTGTCATCGTTACTATAAAGACAAGTGAAGAGCTGAACTCTCC
CATGTACTTCTTCCTGAGCTACTTGTCTTTTGTAGACATCAGTTACTCTACTGTCACAGC
TCCCAAACTCATTTATGACCTTCTTGTTGAGAAGAAGATAATCTCTTTTGTGGGCTGCAT
AACTCAACTGTTTGCGGGCCATTTCTTCGGGTGCACTGAGATCTTCCTTCTCACAGTGA
TGGCTTATGATCGTTACATTGCTATATGCAAACCGCTTCATTACACAAATATTGTGAATAG
GCGTGTCTGTGGCTGGCTGGTGGCAGCTTCTTGGGTGGGAGCCTTTGTACACTCAGCG
GTGCAGACCCTCCTGGCCATTCAGCTACCGTTTTGTGGGCCCAATGAGATTGACCACTA
CTTCTGTGATGTTCACCCTTTACTGAAGCTGGCCTGCACTGACGCCTACATCATTGGTG
TCAGCGTGGCTGCCAACAGTGGTGTGATTTCCCTGAGCTGTTTTGTTGTTCTTGTTGTG
TCCTATGCTGCCATCTCGGTTTCTTTGAGGACACGCTCTTCTGAAGGGCGTCTCAAAGC
ACTCTATACCTGCACTTCCCACATCACTGTTGTAGTTTTGTTCTTTGGGCCGTGTGTTTT

CATCTATATGCGCCCTTCCACCACCTTCTCAGCAGACAAGATGGTGTCTGTGTTCTACA
CCATCATCACGCCTATGCTCAACCCCTTGATCTACACCCTCCGAAATGAAGAGGTGAAA
AATGCCATGAAAAGGTTGTGGAGCAGAAAAGTGAAGAGGAGTGAGAAATGA
>Loon_OR10R2 (Loon37)
ATGATGAATGAAACCAAAGTGACAGGGTTCGTCTTGATCAGATTTTCAGATTTTCCAGT
TCTTCAGATTCCGTTATTTGTCTTTTTTTTCCTGACTTACCTGGTCACCCTCACTGGAAA
TGTCCTGCTAATGACAATTATCAGGCTTCATCATCATCTTCACATCCCCATCCTTTCCATT
CTCTCTTTTTCAGAACCTTGCTATACATTTGCTGTCATTCCCAAGATGCTGGTAAATCTA
ATAACAGAAGAAAAGTCCTTTTCTTTCACTGGATGTGCTGTTCAGATGTTTTTCTTCATT
GGCTTTGGAGGCACTAACTGTATGCTTCTAACAGCAATGGGGTATGACCGATGTGTGGT
TATATATAAATCTTTGCATTACAAAGTCCTCATGAACGACAGAGTCTGTAGACAGCTAGT
GGCCTTTGCCTCAGTGACTGGCTTTACCTTGTTCCTGATAGGTACCTACTTTATATTCAC
ATTGCCTTTCTGTGGAGAGAAGGAAATTAATCACTTCTTCTGTGACATGGCTCCTGCCA
TTCAGGCAGCTTGCACAGAAAGTAACGGAATTGAGATAGTAATTTTTATTTTTTGTATTG
TGGTTGTGTTTGGCTCATTCTTGTGGATTCTTCTCTCATATGTTTTGATCGTCAACGCCAT
CCTCAAGATCCCCTCTACTGAGGGGAAAAGTAAAGCCTTTTCCACTTGTGCCTCCCATC
TCACTGTGGTTGTTGTGCACTTTGGGTGCGCATCCATCATCCATTTGAGGCCCAAATCC
ACCTATTCCCTGGAGGCGGACACTCTGATTTCTGTCATTTATGCTGTGGTGACTCCCTTG
CTGAACCCTGTGGTTTACAGCCTGAGGAACAAGGATGTGCACTTGGCCCTTCGGAAAG
GTGTGCACTTGGCCCTTCGGAAAGGCCTGGGAAGAAAATTGTGCACAAGAATGAGGT
GA
>Loon_OR11L1 (Loon38)
ATGACAAATGTTACAGCAATACTGGAATTCAGGCTACTGGGCTTCAGTAGCAACCCAC
ACTGCCAGATCCTGCTATTCACAGTGTTTTTGGTTATTTATATTCTCACCATCGTAGGAA
ACATCATTATTATTTTAGTGGTGACACTGGAGCCACGACTTCATTCACCCATGTACAAAT
TTCTCAAGAACCTCTCTTTCCTAGAGGTCTGTTACACCACCACAATTGTACCCAAGATG
CTGGCCAATCTACTGGCAAAGAGGAAGAGCATCTCATTCTCAGGATGCATGGCACAAC
TTTACTACTTCATTTCCTTGGGAGCCACTGAGTGCTACCTCTTGGCAGCGATGGCGTAT
GACAGATACCTTGCGGTCTGTGAACCCCTGCACTATGGTGTGGCCATGACTGCTGAGT
CTTATACCCGTCTGGCTGTGGGTTCGTGGGTCACTGGTGTTTTCACTGGTTTTCTGCCC
TGTCTGATGGTCTCCAGATTGCATTTCTGCAGTTACAACCTTATTGATCACTTCTTCTGC
GATATCTCTCCACTATTGAAGCTCTCATGTTCAGACACTACTGTCACAGAAACTGTCAT
CTTCATCCTCTCTCTCCTGGTCCTTTCCAGCTGTTTTCTGTTGACGCTTGTCTCATACCT
ACTTATAATTCTCAGTATCCTGAAGATACCCTCTGCTTCTGGAAAAAGAATTACCTTCTC
CACCTGCAGCTCACACCTCATGGTAGTGACGATATACTATGGTACAATGATTTCCATGTA
TGTCCGTCCCATCTATAACCTGTCCTCAGAGCTCAATAAGGCTGTATCTGTGCTCTACAC
AGTCGTCACACCCCTTCTGAACCCAGTAATCTACAGCTTGAGAAACAAGGCATTCCAG
AAGGCCTTGGGAAAAATAGTCATCGGACACCATCATCTTCATTCTTTGTAA
>Loon_OR5V1 (Loon39)
ATGCAGAGGGTCAACCTATCAACGGTATCTGAATTTGTTCTTGTAGGCCTTTCTGATGCT
CCAGAAGTCCGTTTTCTTCTCTTTGCGCTGTTTTTGATCATTTATTTGGCCACCATGGCA
GGCAACATCACAATCCTTGTTGCCATTAGCACAGAAACGCGTCTGCACAACCCCATGT
ACTTCTTCCTTGGCAACTTATCCTTACTGGATATCTTATGTCCCACTGTCACTGTGATGA
AGATGCTGGAGGCCTTGTTGCTTGAGAACAAGGTGATTTCATTCACTGGCTGCATGTTC

CAGCTGTTCTTCCTCATTGATGTTGTAGGTACAGAGATTTTTCTCTTGGCTGTTATGGCG
TATGACCGTTATGTTGCAATATGTCATCTGCTGCAGTACGTGAATATCATAAGCATGAAA
TTGTGTGCTCACCTAGCCATTGGCACCTGGGTAGTAGGATTTTTTAATTCTCTGTTGCAC
ACATCTTTGATTTTTACACTCTTTTTTTGTGATTCTAATGAAGTCGACCAATATTACTGTG
ATATTCCTCCTATGCTGGCCCTCTCCTGCTCGCCTACTTACAGTAGGGAACTGGTAATTC
TCACAGTTGCTGGGGTCCTTGGAAGCAGCGCCTTTGTGGTCACTCTGATCTCATATATC
TATATCCTCTTGGCTATCCTGGGCATGAACTCTTCTGAGAGCAGGTGCAAAGCTTTCTC
CACTTGTGGTTCTCACCTGACAGTAGTATGCCTTTTCTACGGGACCACCATAGGCACAT
ATGTATGGCCTTCGTCCACCTACTCACCTAATCAGGATAGGATCATTTCCATGCTCTATG
GAATCCTCACTCCCCTGCTAAACCCCATAATCTACAGTCTGAGGAACAAAGAAGTTAA
ATGTGCCCTAAGAAGAGTGATCACCCAAGTGAGAACTGCTTTAACAAGACAAGAACAT
CTGTCTCAGTCTCTGGCATCCTCTGGAGCCCTGGCAATTGGATAG
>Loon_OR10A4 (Loon40)
ATGATTTCTGCACTTAAAAAAAACCTGAGGGACACGACCAGCTGGAATCACACAGTGG
TGACCCATTTCCAATTTTTACCTTTCTCCAGCATTCCAGAGATCCAAGGCTCTCTCTTTT
GTCTGGTATTGCTTGTGAACCTCAGTACTTTGGTGGGAAACAGCCTCATCATTATGATC
ACGATCGTAGATGCTGCCCTTCACTCCCCCAAGTATTTTTTCCTCAAGAACTTCTTCTTG
GAGATTGGCTACACTACATCCACAATCCCCCAGATGCTGGTGAACTTCCTCACAAAAA
GGAAGGGCATATCCTTTCTGGGCTGTGCCATACAGATGTATGCCTTCTCCCTCTTAGGGA
TCACAGAATGTTGTGTGCTGGCTGCCATGGCCTATGATCGCTATGTGGCCGTATGCCATC
CCCTGCACTACACGACCATCATGAGCTGGAATACGTGCTTCCTGCTATCAGCTGTATCTT
GGCTTATTGGGGTCTTGGTGGCCTTAGGGCGGACAACTTTCATCTTTACCCTTCCCTGT
TGTGGGCCCAACAGGATCAATCACTTTTTCTGTGACCTGTCACCTCTGCTGAAGCTGG
CTTGTGTGGATACTTACAAGAATGGAATCACCACCTACATAATAGCTGTCCTCTTCATCA
TGGTACCCTTCTTACTCATAGTTGTGTCATATGTCCAGATTTTACACGCCATCTTCAAGA
TGCCATCAGCTGGGGGGAAGAGAAAAAAGTTCTCTACCTGCTCATCTCATCTGGTCGT
GTTCACTCTGTTTTATGGATCTGGCATTGTGACCTACTTGACACCCAAAGCTTTTTATTC
AAGCAGCAGTACCAAACTGCTCTCCTCTCTCTTACACACTGATGTCTCCAATGATGAAC
CCTTTGATTTACAGCTTGAGGAACAAAGAAGTGAAACAAGCCTTGAAAAGACTGATA
GCCAAAAATATAAGTGTGACATGTTTATGAATTATATTAAATTAAATCTAGCAGTAGCTT
TGATATTGTGTGCATTACTGCAAGGACAATGA
>Loon_OR8U9 (Loon41)
ATGGCAGAAAGCAATTGCACCCAAGTGACTGAGTTCAGCTTAACGGGGTTCACAGAG
GACCCAGTGACTCAGGTCACCCTCTTTCTGATGTTTCTGCTCACCTACCTTGTCACCAT
CCTGGGGAACCTTGGGATGATGGCGTTAATCAGGGCCAGCCCTCAGCTCCGCTCCCCC
ATGTATTATTTCCTGGGCAACCTGGCTTTTGTAGACCTCTGCTCTTCCACCATCATCACC
CCCAAGATGTTGGTTGACTTTATATCAGAGAAGAAGGGCATTGCTTACGCTGGGTGCGT
GGCTCAGGTGTTCATTTTTGATCTTTTTGGGATGACCGAATGCTTCCTGCTGGCTACGAT
GGCGTATGACCGTTACGTGGCCATTTGCCATCCCCTGGTGTATCCCCTTGTCATGTCCCC
AAAATGCTGTTTCCAGCTGGTGACAGGGTCATATCTCATAGGGCTGACAAATGGCATGG
GACAGACTATCGGCATGTCCAATTTATCCTTCTGTGGTCCCAGCATCATCGACCTGTTCT
TCTGTGACATTTCCCCTCTGATATCCCTCTCAACCTCCGACACCACCCTCAGCCACATTA
TCCTAAGAACTTCAGCATCTTTATTCGGTGTATCCAGCAGCCTGGTTGTCCTGGTCTCCT
ACGTGGCCATCATCTCTGCCATCCTGAGCATCAATTCAGCCGAGGGCAAGCGCAAAGC

CTTCTCCACCTGTGCCTCCCACCTCACCACTGTGAGCATCTTCTACGGGACGTCGCTTT
TCATGTACTTAAAGCCCAGCGCAGACAGCTCAAGAGAAGGAGATAAATGGGCTGCGGT
GCTCTACACCGTGGTGACTCCCATGCTGAACCCCGTGATCTACAGCTTGAGGAATAAG
GAGGTGAAGGAGGCTTTGAGGAGCCTCACAAAAATAAAGTGA
>Loon_OR51E1 (Loon42)
ATGTCATCTTCCAATAGGACTGGGTCCAGTCCCCTGACCTTCATTCTCTCTGGCATTCCC
GGTCTGCTGATGAACAGCTACTGGATGGCATTGCCTCTCTGCTGCCTGTACCTCCTCAT
GCTTCTGGGGAACTGTGCCTTACTCTGGATGATAAAGACAGACCACAGCCTCCATAGA
CCGATGTACTATTTCCTCTCCATGCTGGCCATGGCAGACCTGGGCTTGTCTCTCTCCACC
TTGCCCACCATGCTGGCCATTTTCTGGTTTGAGTCCACCTCCCTCCATTTTGAGGCGTG
CATTGTCCAGATGTACTTCATCCACTCCTTCTCTGCCATCGAGTCTGGGGTCCTGGTGG
CCATGGCATTCGACCGCTTCGTTGCTATTTGCTACCCTTTGCGCTATGCCTCTGTCCTGA
CGAGCTCCCTGATAACGAAGGCAGGGGGTGCGATCTTCATGCGGGGCATCTGTGTGGT
GCTACCCGTTGCCATCCTTATTAAGAAGATGCCTTTCTGCAGGTCGCGTGTCCTGTCTC
ACTCCTTTTGTCTACACCAGGACATGCTGAGGCTGGTTTGTGGGGATGTCAGGGTCAA
CAGCTTGTACGGGCTGACCGCAGTGATACTCACCAAGGGCCTGGACTCCCTGAGCATC
CTTCTGTCTTACATGATGATCATCAGAGCCATCTTGAACATCGTCTCCCAGGAAGCACG
AGCCAAAGCCTTCAGAACATGCATCTCCCACCTCTGTGCCATCCTGATCTTCTACATCC
CGCTCATTGGCTTGTCCATCATCCACAGGTTTGGGAAGCACCTGCCCCCCCTCACTCAC
TCGCTCCTGGCTGATACCTATCTCCTGGTGCCGCCTGTCCTGAACCCCCTCGTGTACAG
CTGGAAAACCAAGCAGATCCGCAGGCGGATCCTCATCCTGCTCTGTTGGAGAGGGACC
CAGCAGCGGGTCTAG
>Loon_OR5B21 (Loon43)
ATGGAAAACCACACCAAGGTAACTGAGTTCATTTTGATTGGGTTCAAATCTCATCCAGG
ATCACAACTCCTTCTCTCTGTTCTCTTCTCAACAATGTATGTTGTCACGGTGGTAGGAA
ATGCCTGCATGATCCTCATCATTAGAATGGACTCCAAGCTGCACACTCCCATGTATTTTT
TCCTAGAGAACTTGTCCATCTTGGACATCTGCTATTCCTCTGTCATCACTCCCAAAGCA
GCACTGATATTATTTCTGGGCAGAGGAATCATTTCCTACAATGGCTGTGCATCTCAAATG
TTCTTCTTCTCTCTCTTTGGCACAACGGAAGCCTTCTTCCTTGCTGTGATGGCTTATGAT
CGCTTCACTGCCATCTGCAATCCACTGCTGTACAAAATCATTATGAATAAAAGACTATGT
GTTTTCATGGTGGTGGGCTCTTATCTGTCAGGCTGCATCAATTGCATCATCCAGACAGG
CTTTACATTCAATTTGTCCTTTTGTGGGTCCAAAGAAATAAACCACTTCTTCTGTGATGT
TCCTGCAGTGATGCATGCCTCCTGTTCAGACACACTTGTCAATGAAATAGTAATGCTGG
CTCTATGTGGATCAATTATCATGGGCACTGCCTTGGTGGTTTTTATCTCCTATGGTTATAT
AATCATCACTATTGTCCAAATGCCTTCAGCTGAGAGCAAGCGCAAGGCCTTCTCCACTT
GCTCGTCTCACATGCTGGCCGTCAGCTTGTTCTTTGGGACTGTTTTCTTCATGTATGCTC
AGCCTGGGGCCACATCTTCACCCGACAAAAGCAACACCATCTCTATCCTCTATACTATT
GTTATTCCCATGCTGAACCCTTTCATCTACACCCTCCGAAACAAGGACGTAAAAGGGTC
TATGAAGAAACAGTTAAAAGGGAAAGGCTTTTTTTAG
>Loon_OR4D5 (Loon44)
ATGGCACTGGGCAACTTCTCACGGGTGACTGAATTCATCCTCCTGGGGCTTTCCGATAG
AAGGGAGCTGCAAGTCCTCTTCTTCACCTTCTTCTTCCTGGGCTATGTCATGGTTCTGC
TGGCGAACCTTCTCATCATTGTGACAGTCAGGACTGACCCCAAGCTGTCCTCACCCAT
GTACTTTCTCCTCTGCAATTTGTCCTTCATAGATATCTGCTGCACCTCTGTCACTTCTCCC

AGGATGCTGGTGGACCTGCTCTCCCAGAGGAAGGCCATTGCGTTTGAAGACTGTATAG
CCCAACTGTTTTTCTGCACTTTGTTGGGGCATCAGAGATGTTCCTCCTGACTGTGATG
GCATATGACCGCTACACCGCCATCTGCAAGCCCCTGCACTACATGGCCATCATGAGCAG
GCAGGTGTGCTGGCTCCTGGTGTCTGCCTGCTGGGCAGGGGGCTTCCTCCACTCCATT
GTCCAGACGCTGCTCACGATCCAGCTCCCCTTCTGTGGCCCCAACACTATCGACAACT
ACTTCTGTGATGTGCATCCTGTCATTCGGCTTGCCTGCACAGACACCAATGTCACCGAG
TGGCTCATGGCTTCCAACAGCGGCTTAATATCCCTGGTTTGCTTCCTGGTGCTGGTCAC
ATCTTACACGTTCATCCTGGTTACAATCAGGGTCCGCTTCACCGAGGGGTACTGGAAGG
CGCTCTCTACCTGTGCCTCACACGTGATGGTCGTCACCCTCTTCTTCGTACCCTGCATCT
TCATCTACCTCCGTCCCTTTTCTACCTTCCCCTCCGACAAGCACGTCTGTGTGATCTACA
CTGTCTTCTCCCCGGTGATGAACCCCCTCATCTATACCCTGAGGAATAACGAGGTGAAG
GCATCCATGTGGAAATTGTGGAAGCGCTGCAGAGTCTTCTGA
>Ibis_OR4E2 (Ibis1)
ATGGGGATCGGTGTCATCAAGCAGGAGAAAAGCAGCGCTGCTCGGCATCAATGTGTGC
CGACACAGATGCTGCCCTGTGTCTGGCTGTGCGCTGGGTGGCCAGAGAAGTCGGCCA
GGGAGCCTTTTGCCCTGCTGGAGATGGGTGTGAGGGCTGTGTCCCTGGCTGCCTCCTT
TGTCTCCTTCTGCCTGAGCACTGCACTGCCAGTTCTGGGAAACCTCAACGGTTCTAGG
ATTCTGGGCCTCGGCAATCGGCCACTCATTGGTCACGATGCTCAAGATGCCACTGCACT
TCCTACCTGTGGAGAGGACAAGCTGCTTTGGCAGCTGCGTGGCCCAGCTCTTCCTGCT
CGCAGTCGTGGCCTGCGACCGATCTGTGGCCATGTGCCAGCCCCAGGGGTCCCCGGTG
TCATGCGAGGGAAGGTCTGTGCCATTGCGGTGGCAGGCCTGAGGGCTGGGGCCACTC
TCCCCTTGTTGGCGCCAGTGGCGCTCACCCTGTGTTTGCCCAGCCGCAGCCCCAATGA
CCTGGACTGCTTCTTCTGTGAGGTACCGCCCATGCTGGCCTGTGCCGAGAGCTGCTGG
GCTGGTGTGCTCCTTGTCTCCAAAGGTGGCACCAATGCCCTGGTGTGCAGCCTGGCCT
TGCTGGGCTCCTGGGACTCAGTGGTTCAGCTCTCCACCAGGGCGGCAGACAGGAGAA
GAGAGACGCAAGGCACTTGCCACTTCACTGTGGTTGTGCTGTTCTTCGGGCCATGGTC
CCGGATCTTCACCCGCCTCACTACCAGCCTCTCCCGGGACAGAGGAGTGTCCGTCTTC
AACAGTGCTGGTGCTGCCTCTCCTCAAGCCACTGCTCTAGAGCCCGAAGAAACAGAA
GGCGAGGGAAGCGGTAAGAAAGTGGGAAACAGAGTGGTATTCTCCAGGATGTGCAAG
GATGGTGGAAGGACTGGGAGCATCCAACTGGGGCAAGTTTCTTTCTAG
>Ibis_OR5V1 (Ibis2)
ATGGAAAACCAACCAAGCAGAACTGAGTTTGTTCTCTTGGGACTTACAAAAGATCCAC
TCCTTCAAAGCCTCCTATTCACTGTGATTTTAATTATTTGCCTAATCATTTTATTAGGAAA
CACAGTAATCATGATGGTCCTAAGGACTGTCATCCACCTTCATTCTCCTATGTACATATT
CCTCTTTCCTTTAGCCCTTGTTGACATCTGTTATGCTGCTGCTGTTGTTCCAAAGATGCT
GGTGAATTTCCTTGTGAAAGGAAGCATAAGCATTGCTGTCAATGCTTGTATGACTCAAA
TGTTCTTCATCTCCCTCTCAGCTGGGTGTGAAGTTTTCATGCTTTCAGTAATGGAATATG
ACCGATATTTGGCCATCTGTAACCCACTGAAGTATCAAGAGGCTATGAGCAAACATTTC
TGTTATCAGCTGGTGGGGAGTGCAAGGGCAATGGGTCTCTCATACTCAGTTCTAAACA
CAATTCCTGTGTTAAATATTCAATTCTGTGGGCATACAGAAATCAAACATTTCAGCTGTA
AGCTGCCCCCTCTCCTGTCTGCTTCTTGCAGTGCGACCTTTCTCAGTAAACTTCTTTTTT
CTGCTGTGATCTTTGGGTCAAGCTCCTTTTTGCTCATCTTCATCTCCTACATCTATATCAT
TGCCGCTGTCTTGAAGATACAGTCTGCAGAAGGGAGGCACAAAGTTTTCTGCCCTTGC
ATCTCCCACTTCACTGTAGTGGGGTTACTCTACATAACTGCTCTGTTCCAATACACAAA

ACCCAAACCAGTCTCACCAATCATTCTAGACCAAGTCCTGTCCACCCAGTACAGCATCT
TAACTCCCATGCTGAATCCCATCATCTACAGCCTGAAAAACAAAGATGTACAAACAGC
CTTAGGCAGAATTCTAAAGAAACTGCAATTTTTGTAA
>Ibis_OR5AP2 (Ibis3)
ATGGTCGGAAGAAACAAGACAACTGTCGATGAGTTCATTCTCTTGGGAATCACAGATA
TTTGGGAGCTGCAGGTCATTCTCTTTGTGCTGTTCCTTCTGATCTGTGTCACCTCATTGG
TGGGGAATCTGGGCATGATTGCATTAATCAGGCTTGACTTTCGACTCCACACCCCCATG
TACTTCTTCCTCTGCCACCTCTCTCTGGTGGACATAGGTAATTCCTCAGCGGTTGCTCCC
AAAATACTAGTGAGCTTCTTTGAAGAAAGGAAAACCATCTCTCTGCCAGGGTGTGCAG
CCCAGATGTACTTTTGTGGAGTCTGCATCATCACCGAGTGTTACCTGCTTGCTGTGATG
GCCTATGACCGGTACATGGCCATCTGTAACCCTCTGCTCTACGTGGCCACCATGTCTCA
AAAGGTTTGTGTCCAGCTGGCTGTGGGATCCTACATAGTAGCGACTGTGAATGAAATA
GTTCTTGTCAGCTCAGTGTTCAATTTACACTTCTGTGGCCCTAATGTCATCAATCACTTC
TTCTGTGACATTCCTCCGCTCCTGAGACTGTCCTGCTCCAGTACGACTGTCAACGAACA
CGTGCTTTTTACCATTGGTACTTTTATTGCACTCAGCACTTTAGTATTCATTGTTGTCTCT
TATGGTTCTATCCTTACCACTATCCTGAGGATCCACTCCTCAGAGGGCAGGCACAAATC
TTTCTCCACCTGTGCCTCACATTTGACATCAGTCTCAGTTTTTTACGGGACTCTGATCTT
CATGTACCTCCGTCCCAGTTCTAGCTACTCCCTGGACCAGGACAAAGTGGTGTCCATTG
CCTACACCCTGGTGATTCCCATGCTGAACCCCCTGATCTACAGCCTGAGGAATGTGGAG
GTGAAGGATGCTCTCAAGAGACTCCTAGAAAAAGTTCTTGTTTCCTTTAGAAATCAAG
CTGGTAAAGAGGTGGCATAA
>Ibis_OR10R2 (Ibis4)
ATGAATGAAACCAAAGTGACGGAGTTTGTCTTGATTGGATTTTCAGATCTTCCAGCTCT
TCAGATTCCATTATTTGTGCTTTTTTTCCTGACTTACGTGGTCACCCTCACTGGAAGTGT
CCTGCTAATGACAATTATCAGGCTTCACTGTCACCTTCACATCCCCATGTATTTCTTCCT
TTCCACTCTCTCTTTTTCAGAAACTTGCTATATATTTGCTGTCATTCCCAAGATGCTGGT
AAATCTACTAACAGAAGAAAAGTCCATTTCTTGCACTGGATGTGCTGTTCAGATGCTTT
TCTTCAGTGGAGGCACTAACTGTATGCTTCTAACAGCAATGGGGTATGACCGATGTGTG
GCTATATGTAAACCTTTACATTACAACGTCCTCATGAATGACAGAGTCTGTAGCCAGCTA
GTGGCCTTTGCAACGGTCACAGGCTTTACCTTGTCCCTGATAGATACCTACTTTATATTC
ACATTGCCTTTCTGTGGAGAGAAGGAAATCAATCACTGCTTCTGTGACATGGCTCCTGT
CATTCAGGCAGCTTGCACAGAAAATAATGGAATTGAGATAGTAATTTTTATTTTTTGCAT
CGTGGTTGTGTTTGGCTCGTTCTTGCTGATTCTCCTCTCGTACGTTTTGATCTTCAACGC
CATCCTCAAGATCCCTTCTACTGAGGGGAAACAGAAAGCCTTTTCCACTTGTGCCTCCC
ATCTCACTGTGGTTGTTGTGCACTTTGGGTGCGCCTCCATCATCTATTTAAGGCCCAAAT
CCGCCTGTTCCCTAGAGGGGGACACTCTGATTTCTGTCACTTACTTACACCGTGGTGAC
TCCCTTGCTGTGGTTTATAGCCTGAGGAACAAGGATGTGCGCTTGGCCCTTCGGAAAG
GCCTGGGAAGAAAATCGTGCACATGGATGAGGTGA
>Ibis_OR6C4 (Ibis5)
ATGAACCAGACTACAGTAGTGGAATTCGCCCTCTTGGGGCTGACCTGCAGCCGCCATT
TGGAGATCATCCTCTTTGTGTTTCTTGTGATTGCCTACTTCTTGATCCTGCTTGGAAACA
TTACTGTCATCAGCATCACTCTTGTGAATCATTTCCTTCAGACCCCAATGTACTACTTCC
TCAGAAATTTTGCCCTTTTGGAAATCACGTTCACCTCCACGTTCATTCCCAGCACCCTC
TACAGCCTTCTGACAGAGAGGAAGATGATTTCCCTGCCTGGTTGTTTCCTTCAGATGCT

GCTTTTCTTTTACTTGGGTACCTGCACATTTTTCCATGTGGCAACAATGTCCTTGGATCG
TTATGTTGCCATTTGCCGCCCTTTGCATTACACAACAATTATGAACAACAGATTTTGCCT
CCAGCTGGTCCTGGCTTGCTGGGCAGTGAGTTTTCTCTTGATGTTTCCTCCCACCATTAT
GATTGTCCAGTTGCCATTCTGTGGTCCCAATGTCATGAACCACTTTTACTGTGATACTTC
CCTGTTGTTGCAACTGTCCTGCACAGACACAGGGTTCATAGAAGGACTGATGTTTACC
GTACTAATTATCATCATACCTGGTACCTTAACAGTAACTGCTGTTTCTTACGGCTGCATTA
TTATCACCATCTTGCATATACCATCTTCCACAGGTAGGAAGAAGGCATTTTCCACTTGCT
CGGCTCACCTCATGGTGGTTATGATATTTTACAGCACATGTATCTACAGGTATATCCGCC
CAGCACAGCGAGGTGGGCAGGAGTCTGACAAAGTTCTTTCTTTCTTATTCTCTGTGGT
GACTCAGATGCTTAACCCATACATCTACTCACTCAGGAACAATCAAGTCAAACAGGCT
CTAAAGGAGAGCATGCTGAAGGCATTTTCTAGCTCCCTAAGGCAGGTGTGA
>Ibis_OR5AU1 (Ibis6)
ATGGTTGAAGATAATTATACATTTGCATCCGAGTTCATTCTCCTGGGCTTCACAAACCGA
GAAGACCTGCAGGTGACAAGTTTTGTCTTATTCCTTGCCATCTATGTGGTGACTCTAAT
AGGAAATCTGGGAGTAATAATATTAATCAGACTCGATGTGTGCCTCCACACCCCCATGT
ACTTCTTCCTAAGCCACTTGGCTCTCCTGGACATCTGCTACTCCTCCACCATCATCCCTC
AAACCTTGCTGAATTTTTTAGCGGAGAAGAAGGTTATTTCCTTCATTAGGTGTGCCACT
CAGCTCTTCTCCTTTGCGACTTGTGCCACCGCCGAGTGCTACGTGCTGGCTGCCATGGC
TTACGATCGCTACGTAGCCATTTGTAAGCCCCTGCTCTACTCTGTGGTCATGTCCCAGA
GGCTTTGCGTTGGGATGTTGGCTGCTGCCTACTTAGCTGGTGTGATCAGCTCCACTATA
CACACAGTTTCCATATTTCGTCTCCCGTTCTGCCGGTCTAAGAGGATCAATCATTTCTTC
TGTGATGGACCACCGCTGCTAGCCCTCTCCTGCTCTGACACCCATGTCAACGAGGTGAT
GGTTTCTGCCGTGGTGGGGTTCAACGTGCTAAGCACCACGGTCTTCATTTTACTCTCCT
ACTTGTTGGTCCTCTCCGCCGTCTTGAAGATGCACTCCGCGGCCACTTGGCACAAAGC
CTTCTCCACTTGTGCCTCTCACTTGCTCTCCATCGCTTTGTACTACGGCAGCTCCCTCTT
CATGTACCTGCACCCCGGCTCCAGACACTCCTTGGAGCGTGACGAGGTGGTCTCCGTG
CTGTACTCCGTTGCAGTCCCCATGCTGAACCCGCTCATCTACAGCCTAAGAAACGCAG
ACATGAAGAACGCCATGAGGAAAGCAAAAGGTAGAGTCCTCTCCTCCTTGTCCATCCA
CGGTTCCTGGTTAGCTGAAAGGAGAGAGCTACCCTTCCATGGTAAGGAGGGTTAG
>Ibis_OR11A1 (Ibis7)
ATGGCTGAAGGGGAAAGGGACAACGGGACGTCATCCACGGATTTTGTGCTGCTGGGA
ATACGTGATGTCCCCATGCTCCAGAACCTGCTCTTTCTCCTCTTGTTTATGACCTACTCG
GTCACCATGGTTGGGAACATCCTCATCGTTGTACTGGTGGTGGCAGACCGGCATCTGC
ACACCCCCATGTACTTCTTCCTGGGCAATCTCTCCTCCCTGGAGATCTGCTACAGCTCC
ACCATCCTGCCTCGGCTGCTGGCCAGCTTCCTGACTGGGGACAGCAGCATCTCTGCTC
ACGGCTGCATGGCTCAGTTCTACTTCTTTGCTTCGTTTGCGACTACTGAGTGCTACCTG
CTGGCAGCCATGTCCTATGATCGGTACTTGGCCATATGCCAGCCCTTGCTCTACGCAAG
CCTCATGACCTGGAAGGTCTCTTTACACCTGGCAGCTGCATCTTGGCTATGGGGTTCAC
TGCTGCTGGTAGTTGTCACAGTCTTCTTATCCCAGTTGCAGTTCTGTGGCCCCAAGGCA
ATTGACCACTTCTTCTGTGAGTTTACTCCGTTGCTGGAGCTTGCCTGCAGTAACACCGG
GGTGTTCATGCTCATTGGGTTCCTCTTGGCCTTCCTGGACTTAATCTTTCCATTCCTGTT
CACGCTGGCCTCCTACATGTGCATCATAGCTGCCATCCTGAGGATCCCATCCGGCACGG
GCAGACAGAAGGCCTTCTCTACCTGCTCCTCTCACCTCACCCTTGTCACTGTTTTCTAT
GGCACCCTCTTTGTTGTCTACATGCTGCCCAGAACAGCCCCGCTGAGGCAGCTCAACA

AAGTGTTCTCCTTTTTCTACACTGTCCTCACCCCCCTGGTCAATCCCCTCGTCTACAGC
CTGCGGAACAGGGAGGTCAGGGAGGCCATCAGGAAAGTGCTCAGGAAAGCCCTGGC
CTGCACCCACAGCTTCCTTGGTGACACAGACACTTTTTGTTCCTTTGAATAG
>Ibis_OR52R1 (Ibis8)
ATGTCTCTGAACTCTACTGCCTTCTCCCACCCTCCCTATTTCCTACTCACTGGCATCCCT
GGGCTGGAGAAGGAGCAGTTCTGGATTGCCTTCCCCTTCTGCATCATGTATGCCATTGC
TGTGCTGGGGAACATCACCCTTCTCCTCATTATAAAGGCAGAGCCGAGCCTGCACGAG
CCCATGTACCTCTTCCTGGCCATGCTGGCCTTCACTGACCTGGTCCTATCAACATCCATG
CTACCCAAAATGCTTGGCATCTTCTGGCTGGGGTCCGGGGAGATTGGGTTTCTCTCCTG
CCTTGCTCAGTTGTTCTTCATCCATACCTTCTCATCGGTGGAGTCGGGTGTGCTCATGGC
CATGGCCTTGGATCGCTACATCGCTATTTGCCACCCGCTGCAGCACTCCAGCATCCTCT
CTGTGCCGGTGGTGGTGGCCCTCGGGAGCCTGGTGCTGGTGCGTGGAGTCCTCCTGGT
GAGTCCCTTCTGCTTCCTCCTCCACAGGATGCCCTTCTGCCAGCATCACATCATCTCCC
ACTCTTACTGCGAGCACATGGCCATGGTGAAGCTGGTGTGTGGGGACACCAGAGTCAA
TGTCATTTATGGCCTCTTTGTGGCTTTCATAGTGACAGGATCTGACATGATCTTGATTTC
TGTGTCCTACACCATGATCCTGCGGGTGGTAATGAGGCTGTCATCCACAGAGGCACGA
CTTAAAGCATTCAGCACTTGTGCATCCCATGTCTGTGTCATCCTTGCCTTTTATGTCCTT
GCCCTCTTCACGTTCCTCACCCACCGGTTTGGGCAGAGCATCCCTCCCCACATCCATAT
AATGGTGGCCAATCTCTACCTGCTAGTGCCCCCCACGTTAAACCCCATTGTTTATGGGG
TGAGAACCAAGAAGCTCTGGGACAGGGTGGTCCTCCTCTTCCAGTGA
>Ibis_OR5J2 (Ibis9)
ATGGCTAAAGGCAATCACACCGCAGTGACCCAGTTCATCCTCCTAGGGCTGACAAGTG
AGCCTAAGCTGCAGGCTCCTCTCTTTGTAATCTTCTTAATGATTTATCTCATCACCCTGC
TGGGCAATCTTGGGCTGATCACATTGATCAAGACAAACCACCGGCTGCACACCCCCAT
GTACTTCTTCCTCTGCAATCTGTCTGTTGTTGATCTTTGCTACTCCTCCGTCTTTTCTCCA
AAGCTGCTTATTGGCTTCTTGGGGGAAAAGAAAACCATTTCTTACCCTGCCTGCTTTGC
CCAGCATTTCTTTTTCCTTGCGTTCGTGACCACAGAGGTGCTCTTGCTGGCTGTGATGG
CATACGACCGCTACGTCGCCATTTGCAACCCACTGCTCTACGCTATTTCTATGCCCAAG
AGGGTCTGCGTTCAGCTGGTGGCCGGGTCGTACGTAGGGGGGATTTTGAACTCCCTCA
TCCAAACGTGTTGCTTGCTGCCGTTGCCTTTTTGTGGACCCAATGTCATCAACCATTAC
TTCTGTGACACCAACCCTCTGCTGAAACTCACCTGCTCTGATGACCGCCTCAACGAGC
TTTTGCTTGTAACCTTCAACGGGACCATCTCCATGTCCGTGCTCTTCACCATCCTCATCT
CCTACGTATACATCCTCCTCTCCATCCTGAGGATGAGGTCTGCCAAAGGAAGGCGCAA
AGCCTTCTCCACCTGCGCCTCCCACCTCCTGACTGTTACCTTGTTCTACGTCCCCGCCG
GGCTAAGCCACATGCAACCGGGCTCCAAGTACTCGCTGGAGATGGAGAAAGTCACTG
CCATGTTTTACACCCTGATCGTTCCCATGCTCAACCCTCCGATCTACAGCTTGAGGAAC
AAGGAGGTCAAGGATGCACTTAAAAAAGCAGCAGCAAATAACATTTTTCTGAGTTGCC
TGCTGACCAAACTGACCCCAATCGGTTGA
>Ibis_OR14J1 (Ibis10)
ATGTCCAACGGCAGCTCCATCACCCACTTCCTCCTCCTGGCATTCACAGACAGGTGGG
AGCTGCAGCTCTTGCACTTCTGGCTCTTCCTGGGCATCTACCTGGCTGCCCTCCTGGGC
AATGCCCTCGTCATCACCGCCATAGCCTGCGACCACCATGTCCACAGCCCCATGTACTT
CTTCCTCCTTAATCTCTCCATTCTTGATCTTGGCTCCATCTCCACCACTGTCCCCAAAGC
CATGGCCAATTCCCTCTGGGACACCAGGGCCATTTCCTTCTCGGGATGTTCTGCCCAGG

TTTTTTTCTTTTTGTTTTTGATGTCAGCAGAATTTTTTCTTCTTACACTCATGTCCTATGA
CCGCTATGTGGCCATCTGCAAACCCCTGCACTATGGGACCCTCCTGAGCAGCAGAGCT
TGTGTCCACATGGCAGCAGCTGCCTGGGGTAGTGGCTTTCTAAATGCTGTGCTGCACA
CGGCCAATACGTTTTCACTCCCCCTCTGCCAAGGCAATGCTGTGGACCAGTTCTTCTGT
GATGTCCCCCACATCCTCAAGCTCTCCTGCTCACACTCTTACCTCAGGGAATTTGGGCT
TGTCATGTTTACTGCCTTTGTATTTTGGTGGTGTTTTGTTTTCATTGTGCTGTCCTATGTG
CAGATCTTCAGGGCCGTGCTAAGGATCCCCTCCGAGCAGGGAAGGCTCAAAGCCTTTT
CCACGTGCCTCCCTCACCTGGCCGTGGCTTCCCTGTTTATCAGCACTGCCATGTTTACC
CACCTGAAGCCCCCCTCCATCTCCTCCCCATCGCTGGATCTGGTTGCTGCAGTGCTGTA
CTCAGTGGTGCCTCCAGCAGTGAACCCCCTCATCTACAGCATGAGGAACCAGGAGCTG
AAGGAGTCCATCAGGAAAGTTATTTTCAGGATGGTTCTCAATAGTGGTCAATTTTTCAT
CACTCTTCAGGAATGA
>Ibis_OR14J1 (Ibis11)
ATGGGAAATGGCTTTGATTTTGCTTGCAGAAGTCTCCCCTATCATCTCACCACCTTTTCC
TCCTTAGACAGTGCCGCATGCCCGGAAGCAGCAAATGTCCAAGGCCAGCTTCCTCACC
GAGTTCCTCCTCCTGGCATTCACGGACAGGCAGGAGCTGCAGCTCTTGCACTTCTGCG
CCTGCGACCACCGCCTCCACACCCCCATGTACTTCTTCTCCTCAACCTCTCCCTCGTCG
ACCTGGGCTCCATCTCCATCATTCTCCCCAAAGCCATGGCCAATTCCCTCTGGAACACC
AGGGACATCTCCTACCAGGGATGTGCTGCCCAAGTCATTCTGTTTGTCTTTTTGATGTC
AGCAGAGTTTTCTATTCTCATCATCATGTCCTACGACCGCTACGTTACCATCTGCAAACC
CCTGCACTACGGGACCCTCCTGGGCAGCAGAGCTTGTGTCCACATGGCAGCAGCTGCC
TGGGGCACTGGGTTCCTCTATGCTCTCCTGCACACTGCCAATACATTTTCACTCCCCCT
CTGCCACGGCAATGCTGTGGACCAGTTCTTCTGTGAAATCCCCCACATCCTCAAGCTCT
CCTGCTCAGATGCCTACCTCAGGGAAGTTGGGCTTATTGTGGTTAGTCTCTTCTCAGCA
TTTGGCTGTTTTGTTTTCATTGTGGTGTCCTGTGTGCAGATCTTGAGGGCCGTGCTGAG
GATCCCCTCTGAGCAGGGACGGCACAAGGCCTTTTCCACGTGCCTCCCTCACCTGGCC
GTGGTCTCCCTGTTTATCAGCACCGCCATGTTTGCCTACCTGAAGCCCCCCTCTGTCTC
CTCCTCATCCCTGAATCTAGTGGTTTCTGGTCTGTACTCGTTGGTGCCTCCAGCAGTGA
AGCCCCTCATCTACAGCGTGAGGAACAAGGAGCTCAAGGATGCAGTGTGGAAACTGA
TGACTGGATGTTTTTTCTGA
>Ibis_OR2D2 (Ibis12)
ATGGCCAGGGAAAACCAAAGCGTAGTGACAGAATTCATCTTTCAAGGCCTTTCCTCCC
AGCCAAGGACACGGACTGTTCTTTTCATAGTGTTCCTGGTTTTTTATCTGTTCACAGTT
GTTGGGAACATCACGATCGTTACGGTGATCAGAGCTGATTGCCAGTTGCAGTCACCCAT
GTACTTTTTCCTTGCCAACCTGTCCTTGTTGGATATCTGCTACGTCTCCAGCAACATCCC
CCAGATGCTACTGAACTTCCTGACCAAGAAGAGGACCATCTCCTTCTCTGGATGTGCT
GCTCAGATGTATTTCTCTCTGGCTTTTGGCATGACAGAGTGTGTTCTGCTTGGGGTCAT
GGCCTATGATCGATATATGGCAATATGTCAGCCCTTGCGCTACACCACTGTCATGAACAG
GAAGGTTTGCATTCACATGGTCGTGGCTTCCTGGACTAGCAGCCTGCTGTGCTCCATGG
TCATCAACAGCCTCACCTTGCGGCTGCCCTTCTGCGGGCCTGACATCTTGAACCATTAC
TTCTGCGAAGTGCCAGCAGTGCTGGCCTTGGCCTGTGCCGACACTGCCCTCATGGAGT
TGGTCATCTTCATTTTCAGCATCCTCATAGTCTTCATCCCCTTTCTTCTGATCATCACCTC
CTACGCCCATATCCTTTTCACCGTCTTGAAGATTCAGTCTGCCCATGTGCAATCCAAGG
CCTTCTCCACCTGTGGATCCCACCTGATGGTGGTAACCATATTCTATGGGACAGCCATCT

TCATGTACATGAATCCTATGTCAAGGCCTCCACAGGACAGGGACAAAGTGGTTGCAGT
GTTTTACACCATTGTAGCCCCAATGCTGAACCCCCTCATCTACAGCCTCAGGAACAAGG
ACATGAAGCGTGCCCTGAGAAGGGCAATGAATAGACCCAAAGCCCTGTTTATTCAAAG
GCTTTTCAGGGATTGGTAG
>Ibis_OR6B1 (Ibis13)
ATGAAGCAGGAAAACAGCACAAAATTCCAGGAATTTATCCTCTTGGGATTTCCAACTAT
CATGGAACTTCAGATGTTGCTCTTTGTGATATTCCTGGTGGCCTACGTGCTGACCATCTT
GGAAAATATACTCATAATTATCTTGATTAAGATGAACCATCAGCTTCACAAGCCCATGTA
TTTCTTTCTCAGCAACCTGTCCTTCCTAGAGGCTTGGTACATCTCAGTCACTGTCCCTA
AACTGCTGATGAATTTTCTTGTGGAAAGCAAGAATATATCTTTTGGAGGGTGTATGACC
CAGCTTTACTTCTTCAGTTCCTTTATCTGTACTGAGTGTGTCCTCCTTGCAGTCATGGCT
TATGACCGTTATGTGGCCATCTGCAATCCCCTGCACTATGCTGCCATCATGAACCACCAA
CTCTGCATGCAACTAGCCACATGCTCCTGGCTCATTGGCTTCTTGGCCTCCATGCTGAA
AGTATTTTTCATCTCTCAGCTGTCTTTTTGTGGCTCCAATGTCATCAACCACTTCTTCTG
TGACATCAGCCCCTTGCTGAACATATCATGTGCTGACATGACAATGGCTGAAATGGTGG
ATTTCACCCTGGCCTTGCTTATCTTGCTGGTTCCTGTCTCTGTCACGATTATCTCCTATAT
ATGCATCATCAGCACCATCCTACGTATCCCCACAGCCCAGGGCAGGAAGAAAGCCTTC
TCCACCTGTGTTTCTCACCTCACTGTAGTCATTGTCTTCTTTTCAGCCACTCTGTTCATG
TATGCCCGGCCCAAGAGAATCCACCCTTTTGACTTAAACAAGCTGGTGTCAGTTGTGTA
CACAATTGTAACTCCTATGCTCAATCCCTTCATTTACTGTCTGAGGAACCAGGAGGTTA
AAGGGGCTTTCAAAAAAACCTTCAGTGTTAGATAG
>Ibis_OR5B12 (Ibis14)
ATGCTCCATGGCAGACACAAACAAGAAAATCAGACATCCCTCACGGAATTCATCCTCT
TGGGGTTTGGGAATCTCCCTAAACTACAGATTTTTCTCTTTCTGGTTTTTTATTAATCTA
CTTTGTAACCATAATTGGGAACATACTCCTCGTTCTTCTGGTTGTGATAGATGAGCATCT
ACATACACCTATGTACTTCTTCTTGGGGAACTTGTCTTGCTTGGAGACCTGCTACACTT
CAAACATCTTACCCAGGATGCTGGTCAACTTCCTGACAAGGGAAAAGAGAATCTCATT
TAACAGCTGCCTCTTGATATTTTATTTCTTTGCTTCCTTGGCATGTGCTGAATGTTACCTT
TTATCTGCAATGTCTTATGATCGTTATCTAGCAATATGCAAACCCTTGCATTATACAACAC
TTATGAATGTCAGATTGTGCATCCTTCTAGCAGCTGGATCTTGGCTTTGTGGATTTCTGG
CTAGCATCTGTACTGCACTTTATGCATCATGGCTGACTTTCTGTGGGCCAAATGAAATTG
ACCATTTTTTTTGTGATTATACACCATTATTAGTGCTCTCCTGCAGTGAAACCCAGAAAA
CAGAATTGCTCACGTCCATTTTAGCCTCTGCATGCACCATGCCTCCATTTCTTTTAACCA
TGGCAACCTATATTTGTATCATCATTGCTATTCTGAAAATCCTTTCCAGCATAGGGAGGC
AAAAGGCTTTCTCTACTTGCTCCTCCCACCTCATTGTAGTCACAGTTTTCTATGGAACC
CTAATTGCTGTCTACACATTGCCCAAAACTAAAGCCCTTAGAGAGTTAAACAAAGTCTT
ATCTGTTTTTTATACTATCTTGACTCCTATGCTCAATCTCCTCATCTACAGCTTGAGAAAC
AAAAAGTTTAAAGAAGCTTTGAGAAAAGCTATTGGCAAATATATTCATTAA
>Ibis_OR5V1 (Ibis15)
ATGGCAGGTGAGAATCAGACTCATGTGACGGAGTTCACGCTCCTGGGCTTTTCTCACA
GCCAGCCCTTCCTCTTTGTTCTTTTCCTGGCCATTTACCTGGCCACGCTGCTGGGCAAC
TCTGCAATACTTGCCCTCGTGTCCCTGGATCCCCATCTCCACAGCCCCATGTACTTCTTC
CTCAGTCACCTCTCCTGCTTGGACATTTGCTACTCATCAGTGACAGTGCCCAAGATCCT
GGCAAATGCCCTGCGCCCGCAAGCGACCATCTCCTACCACGGGTGCCTGGCACAGATA

TTCTTCCTGATGGGGTGCGCGGGGACCGAGTGTGCGCTCCTGGCTGTCATGGCCTACG
ACCGTTATGCAGCCATATGCCATCCCCTGCGCTACACCCATGCCATGAGCCGGGGTGTC
TGTGTGGCGGCAGTCACCAGCTGCTGGCTCTGGGGGATGCTGGACTCGGCCGTGCAC
ACGCTCCTGGCCTCCAGGCTCTCCTTCTGCGGGGCTGCCCAGCTCCAGCACATCTTCT
GTGACGTCCCTCCTCTGCTGAGGGCTGCATGCAGCAACACCCACCCCAGCCAAGTGGC
ACTCCACGCTGCCAGTGTCTTTGTGGGCCTCAGCCCCTTCCTGTTTGTCATTGTCTCCT
ACCTCCGCATCCTGGCCACTGTCCTCAGGATACCTGTGGCCACTGGCCGGCACAAGGC
CTTCTCCACATGCTCTGCCCACCTGCTTGTCGTCGCCCTGTACTTTGTGACGGCCAACC
TGAACTACAACCGGCCCAGCTCCGGCTACTCCCCGGCAGCTGACACACTGGTCTCCGC
ACTGTACTGCATCATCACCCCCATGCTGAACCCCCTCATCTACAGCCTCCGCAACCAGG
AGGTGCGGGGGGCCCTGCGGAAGGCTGTGTGGGGATGGGGCACGCCAGGCTCCCCAG
GCAGCAATGCATGA
>Ibis_OR52B2 (Ibis16)
ATGGCAGCTCTCAATCAAACCAGCTTGCAGCCTGCCTCCTTCCTTCTGCTGGGCATGGC
GGGCCTGGAGGACCTGCATACATGGCTCTCCATCCCATTCTGCCTGATATACATCTCGGC
ACTCCTTGGCAACTTCATCCTCTTATTTGTCATTGTGACGGAGCGAAGCCTCCACGAGC
CAATGTACCTCTTCCTGGCCATGTTAGCGGTGGCAGATCTCGTATTATCCTCCTCCACAG
TGCCCAAAGCCCTGAGCATATTCTGGTCCCTTTCCAAGGAGATGTCTTTCCACGCCTGC
CTTACCCAGATGTTCTTCACACACCTGAGCTTCATTGCAGAGTCAACCATTCTGCTGGC
CATGGCATTTGACCGGTACGTGGCCATCTGCAACCCCCTGCGATATGCCACAGTGTTCA
CACACTCGGTGATAGCCAAGATAGGGCTGGCTGCAATAGCCAGGAGCTTTTGTGTGAT
GTTCCCAACAATATTCCTCCTTCAGAGGCTGCCGTACTGCAGACACAGCGTCATGCCGC
ACACCTACTGCGAGCACATGGGCATCGCCCGGCTGGCCTGTGCCGACATCTCCGTTAA
CATCTGGTATGGCTTTGCCACCACCCTTCTGTCCCCAGGTGTGGACATTGTGCTCATCG
GGGTATCCTATGTCCTCATTCTCCAGGCTGTCTTCAGGCTCTCATCTAAGGATGCCCAGC
TCAAGACAGTTGGCACCTGCAGCTCTCATGCCTGTGTTATATTGATTTTCTACACACCA
GCATTTTTCTCATTTTTCACTCATCGGTTTGGCCGCAACGTCCCCCACCATGTTCACAAC
CTATTGGCCAATCTCTATGTGCTCCTGCCACCCATGCTGAACCCCATCGTCTACACTATG
AAAAACAAACTCATTCGAGAAAAGGTGTCCCAAGTACTCTTCAGGACTGGGCAAGTG
CGCTGA
>Ibis_OR5B21 (Ibis17)
ATGGAGAGAGGGGAATGGGAGAACCAGACATTGCTGATGGAGTTCCGCTTGCTTGGA
CTGGGGGATGACCATGAGCTCCAGACACCTCTCTTTCTCCTCTGTCTGACCATATACAC
AGTGACCATGGTTGGGAATAGCCTCATCATTGTGCTGGTGGTCACAGACCCGCATCTCC
ACACACCCATGTACTTCTTCCTGGTGAATCTGTCCAGCCTAGAGACCTGCTACAGCTCC
ACCATCCTGCCCAGGCTGCTAGCCAGCTTCCTGACTGGAGATAGGACCATCTCTGTGC
AGGGATGTATGGCACAGTTTTTCTTCTTCGGCACTTTTGCAACTTCTGAGTGTTACCTG
CTGGCTGCTATGTCCTATGACCGGTATCTAGCCGTATGTCAACCCCTGCTTTATGCAAGC
CTCATGAACTGGAAGATATGTCTCCAACTGGTGGCTGGATCATGGGTTGCAGGACTGCT
AATTTCTACAGGAATCACATCTTTCATATCTCGCCAAAGGTTCTGTGGCCCCAGTGCAA
TTGACCATTTCTTCTGTGAAGAAGCTCCATTGCTAGAACTCTCCTGCAGTGACACTGAG
ATGATCAGAATTCTTATTATCATATTATCTTTCCCAGATGTAGTTTTCCCATTTCTGTTCAC
GCTGGCATCCTATGTCTGCATCATAGCTGCCATCCTGAGGATCCCATCCAGCATGGGGA
GGCACAAGGCCTTTTCCACCTGCTCCTCTCATCTCACTATGGTCATTGTTTTCTATGGGA

CCCTCATCATTGTCTACATGTTGCCCAGAACAGTGCCACTGAGACAGCTCAACAAAAT
GTTCTCCTTTTTTTACACAGTCCTCACACCTCTCATCAATCCACTCATCTACGCTCTGAG
GAACAGAGAGGTCAAGGGGGCACTCGGGAGGGTGCTCAGAAGGGCTGCCTTGCATTG
A
>Ibis_OR52B2 (Ibis18)
ATGTATGAGCTCAACGAAAGCAGCTTTGATCCTATTACCTTTGTCCTGACGGGCATCCC
AGGCATGGAGGAGTCCCACATCTGGATCTCTGTCCCCTTCTGTCTGATGTACATCATTG
CAGTGTTTGGCAACTCTGTCCTCCTCTTTGTCATCGTCATGGAAAGGAGCCTCCACGA
GCCCATGTACCTCTTCCTTGGTATGCTGGCTGTTTCTGACCTCATGCTTTCGACCACGAC
GGTCCCCAAAATGCTGGCGATCTTCTGGTTCAGTGCCAGGGAAATTTCCTTCGATGCCT
GCATTACACAGATGTTCTTCACCCATTTCAGCTTCATCGTGGAATCATCTGTTCTGCTGG
CAATGGCGTTCGATCGGTACGTGGCTGTCTGCGACCCGCTGCGGTACTCATCAACCCTA
ACCCCCTCGGTGATCGGGAAAATAGCTGTGACTGCCGTTGTCCGGGGGTTCTGCATCAT
GTTCCCACCCATCTTCCTCCTGAAGCGGCTGGCGGGCTCCCACACCTACTGTGAGCAC
ATGGGCATCGCCCGCCTGGCCTGTGCCGACATAAAAGCCAACGTCTGGTACGGGCTGA
CAACGGCTCTTCTCTCCTCCGGCCTGGACGTTGTGCTCATCGCTGTCTCTTACGCTCTG
ATCCTCAGGACGGTCTTTCGGCTCCCGTCCCCGGAGGCTCGTCTCAAAACCCTGAGCA
CCTGCGGCTCCCACCTCTGCGTGATCCTCATGTTCTACATGCCCGCCTTTTTCTCCTTTC
TCACGCATCGGTTTGGCCACCACATCCCAAGTCACGTTCACATCCTCCTGGCCAACCTC
TACGTCGTGGTCCCGCCGATGCTCAACCCCATCGTGTACGGGGTGAGGACAAGGCAGA
TCCGGGAGCGCGTCGTCCGCCTCTTCTGCCCCGCGGGGGAGTGCCCCTGCCCCGGCGG
GGGGAGCAGGTGCTGA
>Ibis_OR6M1 (Ibis19)
ATGGGAGCAGAAAATGAAACTGCAGTTACTGAGTTCATCCTAGAGGGTTTCTCAGGGC
TTGATCAAAGACTACAGCTATTTCTCTCTCTGCTCCTTCTGCTCATATACCTGACAACAG
TGATGGGGAAGGCAACCATCATTTTCCTCCTGGGTGTAGATCACCGCCTGCAAACCCC
CATGTACTTTTTCATCAGCAATCTGGCCTTCCTGGAAATCTGGTTCACATCCTCCACAA
GCATCAAATTGTTTGTGATCCTGAGTTCTGGTAGGAGGACAATCTCACTAAGCAGCTGC
TTTGCCCAGTCCTGTTTCTATTTTGCCCTGGGCTGTACAGAGTTGGTTCTACTTGTTGTC
ATGTCCTTTGACTGCTACGTTGCCATCTGCCAGCCTTTGCACTATGCTGCCATCATGAAG
CCTCAGCTCTGCATCCACCTGGTTGTTGCTGCTTGGGTCATAGGCATCACAGCCTTCAG
TTACTGTCTGGTCCTCCTCTACAAGCTGACTTTCTGTGGCTCAAACAAGATCCACTCCC
CCTTATTCAAATTGTCCTGCTCTGACCCCAGCCTGCTTTGGAAAACAGACTCTGTTTTA
TTATCGTTTATCATACTGGGTTCCTTATGTTTAATTCTGGCATTTTACATGGGCATCCTTTT
CTGTATTCTGCCCCTGCCAGCAGCTGCTGGGAGGAAAAAAGCTTTTACTACATGTTCTT
CCCACCTCACCACCTTGGCCATTGCATATGGGAGCTGCATTGCTCTCTATGTGCATCCTT
CCAGAGATGTTTCCTTGGAGACAAACAGAACTGTAGCTTTGCTGAACACTGTCCTGTA
CCCACTCTTAAATCCATTCATCTATAGCCTTAGAAACAAGACTGTGATACTGGCCCTGA
ACGAAACCATTGCCTGTGCAACAGCACAGCTTTTCCCCTAA
>Ibis_OR51E2 (Ibis20)
ATGCTCTCCACCAACAGCTCTGAGATCAGCCCATCCTCCTTCATCCTGGCCAGCATCCC
AGGGCTGGAGGCTGCCCATTTCTGGATGGCGATCCTTCTGTGCTCCATGTACGTCTTGG
CGGTCACAGGCAACTGCGCGGTGCTGTTCATCGTGAAGATGGAGCCCAGCCTGCATGC
TCCCATGTACTTCTTTCTCTGCATGCTGGCTGCCATCGACCTGGCCCTGTCCACGTCCA

CGGTGCCACGCACCCTCTCCTTTTACTGGTTCAACACCAGGGAGATCAGCTTTGACGC
TTGCCTTGTCCAGATGTTCCTCATCCACACCCTCTCAGCCATCGAGTCCACTGTCCTCC
TGGCCATGGCCGTGGACCGGTACGTGGCCATCTGCCACCCGCTGAGACACGCTGCCAT
CCTCACCAACGCTGTGACAGCGAAAATAGGGCTGGTGGCCATGGCCAGGGGAGTTCT
CTTCTTCCTGCCTTTGCCTTTGCTCCTCCTGCCCCTTCCCTTCTGCAGCTCCCGGCTGCT
GTCCCTCTTTCTCTGCCTGCACCAGGACGTGATGAACCTGGCCTGTGCCAACACCACC
CCCAGCGTGGTCTATGGCCTCACCGCCATCCGGCTGGTCATGGGGCCGGACGCCATCC
TCATCTGCCTCTCCTACATCCTGATCCTCAAGGCTGTCTTGCGGTTGGCATCATGGAAG
GAAAGGCTCAAGGTGTTCAGCACCTGCATTGCCCATATCTGCGTGGTCCTGGCCTTCTA
CGTGCCCCTGATTGGGCTCTCCGTGGTGCACAGGTTTGGGAAGGGCCTGGCTCCACTG
GTCCATATCATCATGGGGAACATCTACATCCTGGTGCCTGCTGTGCTCAACCCCATCATC
TACGGAGTGGGGACCAAACAGATACAGAGGAGGATCCTGAATTTAATTCATATATACAA
TGACAGCACTGCCCATTGA
>Ibis_OR10A7 (Ibis21)
ATGAAGCCAACAGAGGGACCAGAACAAGGTAACCACACTCTGCTGACTGAATTTGTC
CTCTCCGGTTTGTCCAACCACCCAGAACTGCAGCACTTGCTGTTCTTCACATTCTGCTT
AATTTACACCTTCACCGTCATTGGGAACCTTCTCATCTTCATGGCCACACTGCACCCCA
CCCTCTGCACGCCCATGTACTTCTTCCTCCGGGTCCTGTCCTTCCTGGATATTTCCACAG
CATCGATCGTTGTTCCCAAGATGCTGGTAAACTTCCTGTCAGAGGACAGGAGCATTTCC
TACATGGGCTGTGCCACACAGCTCTACTGTGTGATTTTCTTTGCAGCCACTGAATGCTA
CCTTTTGGCAGCCATGGCCTATGACCGTTACATGGCCATATGCAACCTCCTTAGATATGC
AATCATCATGAACAGCAGAGTTTGTCTTTCCTTGGTCCTGCTGTCATGCTGCAGTGGTA
ATGTTGTGTCTGTGGTGCAGACAGCTTGGGTGTTCACATTGCCGTTTCGTGGGCCCAA
GAAGATTAACTACTTCTTCTGTGATATTCCCCCACTTGTTATGCTCTCCTGCACTGATAC
GTCTTTGTATGAAAAGCAGATCATCACAGCCACAGTGCTGGTCATCTTTACACCATTTT
GTCTCATCCTGGTATCCTATGCCTGCATCGTCTCCAGCATCCTGAAGATTTCCTCTGCAG
AGGGCAGGCACAAGACTTTCTCTACCTGTTCCTCACACCTTACTGTTGTAACACTGTAT
TATGCAAGTGGGACCTTGATTTACTTACGGCCAAAATCCAGTAATTCACAATACACTAA
GAAAGTCCTAGCTCTCATATACACGACCATAATTCCCACATTAAACCCCCTGATTTACAG
CCTGAGAAATAAAGATGTGAAAGGAGTACTAATCAAAATTATAGGTGAGCTGAGGAAG
ATATAA
>Ibis_OR5V1 (Ibis22)
ATGAAAGATAAGATGCAGAGGGTCAACCTATCAACGGTATCTGAATTTGTTCTTGTAGG
CCTTTCTGATACTCCGGAAGTCCGTTTTCTTCTCTTTGTGCTGTTTTTGATCATTTATTTG
GCCACCATGGCAGGCAACATCACAATCCTTGTTGCCATTAGCACAGACACTCGTCTGC
ACAACCCCATGTACTTCTTCCTTGGCAACTTGTCCTTACTGGATATCCTATGTCCCACTA
TCACTGTGCCGAAGATGCTGGAGGCCTTGTTGCTTGAGAACAAGGTGACTTCATTCAC
TGGCTGCATGTTCCAGCTGTTCTTTCTCATTGACGTTGTAGGCACAGATATTTTTCTCTT
GGCTGTGATGGCGTATGACCGGTACGTTGCAATATGTCATCCGCTGCGCTACATGAATAT
CATGAGTATGAAACTGTGTGCTCACCTCGCCATTGGCACCTGGGTAGTAGGACTTTTTA
ATTCTCTGTTGCACACATCTTTGATTTTTACACTCTTTTTTTGTGATTCTAATGAAGTTGA
CCAGTATTACTGTGATATTCCTCCTATGCTGGCCCTCTCCTGCTCGCCTACTTACAGTAG
GGAACTGGTAATTCTCACAGTTGCTGGGGTCCTTGGAAGCGGTGCCTTTGTGGTCACT
CTGATCTCATATATCTACATCCTCTTGGCTATCCTGTGCATGAACTCTTCTGAGAGCAGG

CACAAAGCTTTCTCCACCTGTGGTTCTCACTTGACAGTAGTATGCCTTTTCTACGGGAC
CACCATTTGCACATATGTACGGCCTTCCTCCACCTACTCACCTAATCAGGATAGGATAGT
TTCTATGTTCTATGGAATCCTCACTCCCCTGCTAAACCCCATAATCTACAGTCTGAGGAA
CAAAGAGATTAAATGTGCCCTAAGAAGAGCGATCAGCCAGGTAAGAACTGCTTTAACA
AGACAAGAACATCTCTCTCGGTCTCTGGTGTCCTCTGGAGCCCTAGTGATTGGATCGGC
ACTGCAGTTTGACTGA
>Ibis_OR5AP2 (Ibis23)
ATGGGAGGAAATCACACGCAGACTAAATTCATCCTCTTGGGACTTACAGACAGTCCAT
CTGTGCAGGCCCTTCTTTTTGGGTTGTTTCTATTGATGTACATTGTCACTTTGGTGGGGA
ACATTGGGATTATGGTCCTGGTTTGGCTGGTTCCCAGCCTCCACACCCCCATGTACTTC
TTCCTCACCCATTTTTCATTTGCCGATGCCTGCTATTCCACGGTCATCTCCCCCAAAACG
CTAGCAGGCCTCTTATCAGAGAATAAAACCATTTCTTTCGCCGGCTGCGTGACGCAGTT
CCACAACGTTGCTTTCTTTGCAACTGCCGAGTGTCACCTCCTGGCTGTGATGGCCTATG
ACCGGCACGTTGCCATCTGCAACCCCCTGCTTTATGTGACGGTCATCTCCAGCCGCGTC
TGCCGGCAGCTGGTAGCATCATCCTACCTCATCGCCTTTCTCAGTGCCATCATCTACACC
GGCTGCACCTTTGGGGGTTCCTTCTGTGGACCCAACCAAATCAACCACTTCTTCTGCG
ACGTCAACCCCGTGCTAAAGCTTACGTGCTCCGACACCCGCAGCAGCGAGATGGTCAT
CTTTGCCTTTGTCGCCATAAACTTGGTGGGCACCAGCGTGATCATTTTTCTCTCCTACCT
CTGTATCCTCCGCACGGTCCTGAGGATGCGCTCAGCACAGAGCAGGTCCAGAGCCTTC
AGCACCTGCGCCTCCCACTTGACGGCTGTTTCCTTATTCTACGGAACAACAGTCTTCGT
GTACCTACAACCTGCATCCAGCCACAGCAGCCTGGATAAGGTGGCCTCCATCTTCTACA
CCGTGGTCACCCCCGTGCTTAACCCATTCATCTACAGCCTGAGGAACAAGGAGGTGAA
GGGTGCTCTGGTGAAGTGCAGGAGCAGGATATTAAACTGCTGCCAACATGAAAGAGTT
GTATCAGCTAGGAAGCAAACAGTATCGTAA
>Ibis_OR52I1 (Ibis24)
ATGGCTTCTGATCCCTTCGACCGCCCCAACAGAAACTCCTCCTCCTTCATCCTTGTGGG
TGTCCCCAGCCTGGAAACTTCCCCCATCTGCCTGGGCATCCTTTTCTGCTCAGGCTATAT
CATCACCTTGGTAGGAAATGGTGGTGTTTTGCTTGTCATTGGGCTGGACAACTCCCTGC
GTGACCCCGTCCATTGCTTCCTGGGCATGCTGGCAGTCATTGATGTGGTGATGGTGACG
ACCATCGTTCCCAAGATGCTGAGCGTGTTCTGGCTGAACTCTACACAGATTAGTTACAT
GGCCTGTTTTGTTCAGATGTTCCTTGTTCACTCCACAACATCAGAGGAGTCAGGAGTG
CTCTTGGCCATGGCTGTTGACCGCTACGTTGCAATTTGTCACCCCCTTAGGTACCAGGC
CATCTTGAATCGCCAAACAATTGCCCAGATAGGCCTGGCCATTGTGGTGAGAGCTCTCC
TTTTCATGGACCCCTTGACTGGGATGGTGACAAAGCTCCCCTATTGCCATTCCCACGTT
GTTCCCCATTCATACTGCGAGCACATGGCCATAGCGAAGCTGGCGTGTGCAAACCCCA
GACCCAGTGGGCTTTACAGTGTGGCTGGGTCCTCTCTTATAGTAGGGATGGACATGGCT
TTCATTGCTGTGTCCTATGGGATGATCCTTAAAACTGTCCTGGGGAAGAAATCGTGCTG
GAAGGCCTTCAGCACCTGTGGGTGTCATATCTGCGTGATACTGCTGTTTTACGTCCCTG
GGATAGTCTCCATATATGCACAGCAGTTCGGCAGTGGCATATCTGTGCATGCACAGGTT
CTGCTGGCTGATCTCTACCTGACCCTCCCCACTGTGCTGAACCCCATCGTTTACAGCAT
GAGGACCAAGCAGATCCGTCAGGCAGTGCTCAAAATGCTATTTTCCAGGAAGGTTCAT
GCCTGA
>Ibis_OR6B1 (Ibis25)
ATGAAGAACCAAACTGCTATCAATGAGTTCATTCTTCTGGGATTTTCCTATGGGCTGCA

GGTCCAGACCTTGCTTTACCTGCTCTTTCTGGTCACCTACACGGTAACAATCACTGAGA
ATGCAATCATCATCGTTGTGGTGAAAAGGAACCATCACCTCCAAAAGCCCATGTATTAT
TTCCTGGGGAACTTGTCCTTCCTGGAGATTTGGTATGTGTCAGTAACATTGCCTAGGCT
TTTATTTGGGTTCTGGTCACAGAGCATGACCATCTCATTCTCCAGCTGCATGACGCAGT
TATACTTCTTTATCTCCCTTATGTGTACTGAATGTGTTCTCTTGGCTGTAATGGCCTATGA
CTGCTATTTGGCTGTCTGCCATCCCCTGCGCTACCCAGCCGTCATGACCCACAAGTTGT
GCTTTCAGCTGTCAGTTCTTTCATGGGCAGGAGGCTTTTCCATTTCCTTGGTCAAGGTT
TCTTTTATATCACGACTCACATTTTGTGGCCCACAAGTCATAAACCACTTCTTTTGTGAC
ATCTCTCCAGTGCTGAACCTTTCCTGCACTGACATGTCCCTTGCAGGGACAGTGGACTT
TGCATTAGCCTTGGTGATCCTGCTTGTACCTCTCTTGATCATTGTTTTCTCCTACTGCTGC
ATCTTGTCAACTATCTTGCGTATGCCTTCAGCCCAGGGAAGGAGAAAAGCCCTTTCCAC
TTGTACATCCCATTTCACTGTAGTCATTATCTTTTTCTCAGCCACTCTCTTCATGTATGCC
AGGCCCAGGAGGATCCATCCATTCAACCTCAACAAAATAGTGTCTGTCTTTTATGCTGT
ATTCACTCCAGCATTGAACCCTCTAATCTATTGTCTGAGGAACAAGGAGGTGAAAGAG
ATTCTGAGAAAGATCATAGGCACAAGCTGCTCTGCACACCAATAA
>Ibis_OR4S2 (Ibis26)
ATGTGCCTAGGCTGTACATTGCATATCACCGCCCAACTGATTAACAAGGATAATGCAAG
CAGTGTGAAGGAATTCATTCTTCTGGGTCTCTCAGAGAATCAAAGGGTGCAAAAAATA
TATTTTGTGATGTTTCTGTTCTTCTATATGCTTATTGTAGCAGGAAATCTGCTCATTGTTAT
CACTGTAATTAGCAGTCAACGTCTGAACTCTCCCATGTATTTCTTCCTCTGCTACCTGTC
CTTTGTAGATGTCTGTTACTCTTCTGTCACAGCTCCCAAAATGATTGCTGACTTCCTTGT
TGAAGATAAAACCATCTCCTTTGTGGGTTGCATAGCACAGCTGTTTGGGGTGCATTTCT
TTGGCTGCACAGAGATCTTCATCCTCACAGTGATGGCCTACGATCGCTACATTGCCATC
TGCAGACCTCTCCACTGCACCACCCTCATGACCAGGGGTGTGTGTGGCCGGATGGTGA
TCGGCTCGTGGGTGGGAGGCTTCATCCACTCCGTAGTGCAGACTCTTCTAACCACTCA
GCTCCCCTTCTGTGGCCCTAACAAAATTGACCACTACTTCTGCGATGTCCACCCCCTAC
TACAACTGGCCTGTACCGACACCTACGCTGTGGGCATCATTGTCGTTGCCAACAGCGG
AATGATAAGTCTGAGCTGTTTCTTCATCCTGGTCATGTCCTACGTTGCCATCCTGGTTTC
CTTGAGAAGTCAAACATCCGAAGGGCGGCACAAGGCCCTCTCCACCTGTGGGTCCCA
CATCACCATGGTGATTCTGTTCTTCGGGCCATGCACATTCATCTACATACGTCCGTCCAG
CAATCTGTCGGAGGACAAGAGCGTGGCGGTGTTTACACTGTCATCACACCCATGCTG
AACCCGCTCATCTACACGCTAAGGAATGAGGAGATGAAGAATGCCATGAGAAAACTGT
GGAGTAGAAAAGTGGAAAGTTAA
>Ibis_OR10A7 (Ibis27)
ATGAGCCCCAAGTCCCTGCAGCGGGGAAATCTCAGCAGCCCCACCATATTCCTTCTCTC
GGGATTTTCCAGTGCCTACAGAGCATGGGTGACCTTCTGCCTATGCTTCTCGCTCATTTA
CCTGGTGACGGTGCTGGGGAACCTGCTCATCGTGACCCTCGTCTGGCTGGACACCCAC
CTGCACTCCCCCATGTATTTCCTCCTGGGCCACCTCTCCTTCCTGGACATCTGCTACTCC
TCCGTCACCCTCCCTAAGATCCTTGGAGACTCCTTCTCACCGCAGAAGACCATCTCCTT
TGTGGGCTGCATCACGCAAATCTACTTCTTCCTTTGCTTTGGGGGCTCCGAGTGCATGC
TCCTGGCTGCCATGGCCTACGATCGGTACCTGGCCATCTGCCACCCCCTCCACTACCCA
GCGCTCATGAGCAAGAAGATGTGCCACTGCTTAGTGGCCGTTTCGTGGCTGAGCGGCT
CCTTCTCGTCCCTGATCCAGGCTTTCCTCACAGCCCGCTTGCCCTTCTGCGGGTCCAAC
ATGATCAACCACTTGTTCTGCGAGATGCCCTTCTTGCTGAAGGCGTCCTGCAGCACTCA

GGCTCTCCTTAACAAGGCCGCCTCGTATGCTTTGGCTGGGACTATTGCGATGGGCTCTT
TCCTCCTTACTCTCGTGTCGTACGTTCACATCATCAGGGCTGTCCTCCAGAAGGGAGCA
GGGACGCAGAGGGCCTTTGCTACCTGCACCTCCCACTTGACCGTGGTGTCTCTGTTCT
TCGGCGCTGGGGCCGTTGTGTACCTGGTGCCTCAATCCAGTGGCTCCAAGGAGATGGC
TGATGTCCTCGCCCTGCTGTACGCCGTTGTGACCCCCATGCTCAACCCCATCATCTACA
GCTTGAGAAACAGCGAGGTCAAGGGGGCCATCAGGAAGGCCCTGCGCAGGGGGGTG
TTACAGATGTCCACCAAGGGTGCAGGCATCGCCGCTGAGCGACCCCCACCCTCCCCGC
GGCAGGGATGCGGCTCTCGGTGA
>Ibis_OR5J2 (Ibis28)
ATGGCAGAGCAGAATCACACCTCAGTGGCAGAGTTCGTTCTTGAGGGCCTGAGCGAC
CAAGTGGAGATGAAGGCAGCCCTCTTTGTGTTGTTCCTGCTCATCTACACCATCACCCT
TGTGGGCAACGTGGGGATAATTGTCGTTATCCGAGGTGACCCACGACTCCCCACATCCA
TGTACTTCTTCCTTGGCAGCCTCTCCGTTGTTGATATCTGCTTCTCCTCTGTGACTGCCC
CCAGGACCTTGGTGAACTTCCTATTGGAGAGGAAGACCATTTCCTTCGTTGGCTGCATG
GGCCAAGCCTTCTTCTACATAGTCTTAGTGACGACTGAGTGTTTCCTGCTGGCCATCAT
GGCGTACGACCGGTACATGGCCATCTGTAATGCCCTGCTTTATTCCTCTGTTATGACTCG
GAGGTTGTGCGTGTGGCTGGTGGTGGGGTCCTACATTGGGGGTGTCTTGAACTCCATC
ATACAGATGACCTTCATCATTAGGCTGCCCTTCTGCAACTCCAATGTCATCAACCACTTC
TGTGATGTTCCTCCCCTCCTGGCTCTGTCCTGCGCCAGCACCTACATCAGTGAGATGGT
CCTCTTCTCCTTGGCTGGCATTATTGAGCTCAGCACCATCTCCACCATCCTGGTCTCCTA
CATCTTCATCTCCTTTGCCATCCTGAGGATCCATTCAGGTGATGACAGGCAAAAAGCCT
TCTCCACCTGCGCGTCCCACCTGATAGCTGTGACCATGTTGTATGGGACAACAATTTTC
ATCTATTTACGCCCCAGCTCTATTTACTCCCTGAACACTGACAAAGTAGTCTCCGTCTTC
TGCATGGTGGTCATCCTGATGCTGAACCCCCTCATCTACAGCCTGAGGAACCAGGAGG
TGAAGGATGCTCAGAGGAGAACAGCAGAAAGAATCACAGTCAGGCTCTGA
>Ibis_OR52B2 (Ibis29)
ATGCCGCCATTCAACCTTACCAGCTTGACGCCAGCAACTTTCATCCTGTCTGGCATCCC
AGGCATGGAGAAGGCGCACATCTGGATCTCTATCCCATTCTGCTCTATGTATCTGGCTGC
CTTGCTAGGAAATGGTGTCGTGTTGTTTGTCATAAGGACAGAACGTAGCCTCCACCAG
CCTATGTACCTCTTCCTGTCCATGCTGGCCATTGCTGACCTGATGCTGTCGACCACGAC
TCTGCCCAAGATGCTGGCTGTGTTCTGGTTCAGCGCCGGGGAGATTTCCTTCGGTGCCT
GCCTGACCCAGATGTTCTTCCTGCATTTCAGCTTCTCGGCAGAGTCGATGATCTTGCTG
GCCATGGCGTTTGATCGGTTTGTTGCCATCTGTTACCCGCTGCGGTACGTGACAGTGCT
GACCCGGTCAGTCGTTGTCAAAACTGGGCTGGTGGCTTTGCTGAGAAGTTTCTGCATC
ATTTTCCCATGTATATTTCTTTTGAAAAGGCTGCTGTTCTGTGGGCACCACGTCATCCCG
CACACCTACTGCGAGCACATGGGCATCGCCCGGCTGGCCTGTGCCGACATCTCCATCA
ATATCTTGTATGGCCTCGCAGTGCCTTTTGCAACAATAGTGGTAGATGTTGTACTCATTG
CTGTCTCCTATGTCTTAATTCTTCTGGCATTATTCAGACTCCCTTCCAGGACTGCCCGTC
ACAAGGCTTTCAGCACCTGTGGCTCTCATCTCTGTGTTATATTGCTTTTCTATATTCCTGC
TTTTTTCACTGTTTTAATGCACCGCTTTGGTCGGCAAGTCCCCCAGCACATCCACATTCT
GCTGGCCAACATGTACGTGCTCTTCCCCCCGCTGCTGAACCCCGTTGTGTATGGTGTAC
GGACACAACAGATAAAAGAGAAGGTTGTGAAAGTGTTCATCTGCCCCAAGAGTCATTT
GCTGCAAGAATAA
>Ibis_OR5AU1 (Ibis30)

ATGATGGCCAAAGGAAATTGCACCCCCGGTGCCGAATTTGTTCTCTCGGGGTTCTCGG
AGCAGGGGGATGTCCAAGCTGTCCTCTTCACGGTCTTCTTGGTGATCTACGTGATCACT
CTTCTGGGGAATCTGGGGATGTTCGTGTTAATCAGGCTAGATGCCCAGCTTCACACCCC
CATGTACTTCTTCCTGAGCAGCCTGTCCTTCCTAGACATCTGCTATTCCTCCTCAATTAC
CCCCAGGCTGCTCTCAGACCTCCTAGCAGAAAGAAAGGTCATTTCTTACTCTGCGTGC
CTCACACAATTTTATTTCTATGCAGTCTTTGCCACCACTGAGTGCTACCTCTTGGCTGCG
ATGGCGTATGACCGCTACGTGGCCATCTGCAGCCCGCTGCTCTATGCCACCTCCATGTC
CGGCAAAGTCTGCGCGCTGCTGGTAGCTGGCTCATACCTCGCTGGGATTGTGAATGCC
ACCATCCACACAGGGTTTGCACTTCGTCTGTCCTTCTGTGGTCCCAACATCATCAACCA
CTTTTACTGCGAGGGGCCCCCGCTTTACGCCATCTCTTGCACGGACCCCACCGTCAATG
AGATTATGATGTTCATTGTGGTTGGCTTCAACCTGTTTGTCACCAACCTGACCATCCTCA
TCTCCTACACCTACATCCTGGCCACCATCCTGAGGATGAGCTCGGCCGCGGGCAAACA
CAAAGCCTTCTCTACCTGCGTGTCCCACCTGACCACCGTGACCCTCTTCTACGGATCTG
CTGCGTCCATGTACTCACGACCCAGCTCCAGGCACTCCCAGGACCTCGACAAAGTGGC
CTCTGTGTTTTACACTGTGGTGACCCCCATGCTGAACCCCCTCATCTACAGCCTGAGGA
ACAAGGAGGTGAAGAATGCGCTGGGGAGAGTGATGGGGAGGAAACGTTCAGCTGAA
AAATAG
>Ibis_OR14I1 (Ibis31)
ATGTCCAACGAAAGCACTGTGACAGAGTTTCTTCTCCTGGGATTCTCTGATACTCGAGA
ACTGCAGATATTACACTTTACGGTATTTCTGGGGATTTATCTGGCTGCCATGGTGGGAAA
CCTTCTCATCATTGTGGTCATAGCTGTTAGTAATCACCTTCACACCCCCATGTACTTCTT
CTTGATGAATTTGTCCATCTTAGACCTTTCAACTGTCTCTGTAACCCTCCCTAAGTCCAT
GGCCAATTTTCTGTTGAATACCACAGCCATCTCTTATTCTGGCTGTGCTGCCCAAGTCTT
TTTCTTCCACGTCTTCGGTGCTGCTGATCTTGCTTTACTCACTGTCATGGCATATGACCG
TTACATTGCCATCTGCAAACCTCTTTACTACAAGACAATCATGAAGGGGACTACTTGCA
TCCGCATGGCAGCCAGTGCATGGCTCAGCTGCGTCCCTTACTCTGCTCTCCACACTGGG
AACATCTTTTATTTATCCTTCTGCAAGTCCAACATCATTGACCAGTTCTTTTGTGAAGTT
CCACAGCTTCTCAAGCTTTCCTGCTCTGATTCATACTTCAGTGAAGTTGGGGTTCTTAC
AGTTAGTTTCCTTTTAGTCGTCATCTGCTTTGCTTTTATAAGTATGTCCTATATTCAGATTT
TCACCGTGGTCTTGAGAATTCCTGTCAAGGAAAAACAGCGTAAAGCTTTTTCAACCTG
CATCCCTCATCTCATTGTGGTCTCCATGTTTGTTAGCACTGGTTCTTTTGCCTATCTTAAG
CCTGTCTCCAACTCCCCATCAGCTCTGGATCTCACAGCTTCTATTCTCTATTCTGTGTTG
CCACCAGTGATAAATCCAGTCATATACACCATGAGGAACAACGACATCAAAGCTGCCC
TGCGAAAATTAACTGAGCACAGATTATTATGCAGAAATAAATAA
>Ibis_OR10K2 (Ibis32)
ATGCCATGTGAAGGCTGCATGGAGAGGGACAATAAGAGCACTGTTGCAACCATGGAGT
TCCTCCTGCTCGGCTTCTCCGAGCTGCTCTGTCTGCGGGTCCTCCTCTTCTTTATCTTTC
TCATTATCCATTTGGTCACGTTGGCAGGGAATGCGATGATCTTCATGGCAGTGGTTATGG
AGCCTTCCCGTCCTCCCATGCTTTTCTTCCTCTGTCAGCTCTCTGTCATCGAGCTCTGCT
ATACCTTAGTCATTGTCCCTAAGGCACTTCTCAGCCTGATAGCAGTGGATGGAAGCACC
ATTTCTTTCATAGGCTGCGTTGCACAGATGCACCTTTTTGTGGCACTCGGTGGGGCTGA
ATGTTTCCTCCTGGTAGCCATGTCGTATGACCGTTATGTTGCCATCTGTCAGCCACTTCA
CTACGTAGCTGTGATGAGTGAGGGGCTCTGCCTCAGGCTGGCTGTGGCATGCTGTCTG
GGAGGCTTTGCCGTTGCCCTGGGGTTGACGGTGGCTGTTTTCCGCTTACCTTTCTGTCA

GTCGCATCGTATCAACCACTTCTTCTGTGATGTCTCTGCTGTGCTGCACCTGGCCTGTA
CGCAGAGTTACACCCGCGAGCTGCCCTTGCTGGCTGCTTGTGTGCTTCTCCTGCTGCTC
CCCTTCCTCCTAATCCTGACCTCGTATGTTTGCATTGCTGCTGCTTTGTTACATGTCACC
TCCTCCGTGGGAAGGGGAAAGGCCCTTTCCACCTGCATTTCACACTTGGCCATCACCT
TGCTACACTATGGATGTGCCACCTTCATGTACGTTCGTCCTAAGTCCAGTTACTCGCCA
GCTCGAGACAAGATGGTGTCTCTGGTCTACACCAATATTACTCCATTGCTGTATCCCCTC
ATTTACAGCCTCAGGAACAAGGAAATCAGACGGGTCCTCAGGAAAATGTTGAGGAGG
AAGAAAATAGCTCAGCTGAACCGGCATGCTATCAGAGCTGTGACGTGTGTGTGGTAA
>Ibis_OR51G2 (Ibis33)
ATGGAGCATGACTCACACACCACGTGGGAATTCAATGGCTCTTTCTATCAGCCTTCAGC
TTTCCTCATGATGGGCATCCCAGGCCTGGAAGCCCTTCACCACTGGATCTCCATCCCTT
TCTGTGCACTGTACCTTATTGCTCTCTTGGGAAACTGCATGATCCTATTCATCATAAAGA
AGACCCCAAGTCTTCACGAACCAATGTACTACTTTCTCTCCATGCTGGCAGTCACTGAC
CTGGGCTTGGTTCTGTGTACGCTGCCTACTACTCTGGGCATTTTTTGGTTTAATATGCGA
AGGATTGGGTTTGATGCTTGCCTCACTCAGATGTATATCATCCACCTACTGTCCTTCATT
GAATCCTCTGTGCTCCTGGCAATGGCATTTGACCGCTTCATTGCCATCTCCCATCCACTG
AGACACTCATCTATACTGACCAAGACGACTGTCATAAAAATAGGTCTGGCAATTATATT
GAGAGGTATGGTCTCCCTCCTTCCCATACCCTTCTTGCTCAAGAGACTAACCTATTGCG
GGAAGACTGAGCTTTCTCATTCTTTTTGCTTCCATCCTGATATCATGAACCTAGCATGTG
CAGATATAAAAGTCAATGTCTTCTATGGTATGATTATTCTCTTATCAACGGTGGGGATGG
ACTTCATCTTCATTGTGCTGTCCTACATCCTGATCATTAAAACTGTTATCAGCCTTGCAA
CCAAGGAGGAATGTCTCAAGGCTTTGAATACGTGTGTCTCCCATATCTGTGCTGTTCTG
GTGTTCTTCATCCCAATGATTGGACTGTCCATGATCCATCGCTTTGGAAAGAACGTTCC
TCCTCTGGTTAACACTTTGGTGGCCTACACTTACCTTATAATTCCCCCTGCTCTCAACCC
CATTATCTACAGCATAAAATCCAGCCATATCCGTGAGGCTTTGCTCAGGGCACTGAGGA
GGAAGAGTGAATCTGACTGGTAG
>Ibis_OR52K1 (Ibis34)
ATGTCAACCGCCAACCGATCCAACACCAACTCTTCGCCTTTTCTCCTGATGGGCATCCC
TGGCCTGGAAGATCTCCATGTGTGGATTTCCATCCCATTCTGCTTTTCATACATCATGAC
CTTGCTGGGAAATAGCATGGTCCTTCTTGCTGTGAGGCTGGACAAAAGCCTCCATGAG
CCTATGTACTATTTCATTTCCATGTTGGCTGTCATCGACCTCATCTTCTCAACTGCTGTAG
TTCCCAAGATGCTGGGTGTATTCTGGTTGGATTCAAGGGAGATTGGTTTTGAGGCCTGC
TTCATCCAGATGTTCTTCATCCACACATTCACTGCAGTGGAGTCAGGGGTGCTCCTGGC
AATGTCCTTTGACCGGTATATAGCCATCTGCAACCCCCTGAGATACAACACCATCCTAA
CGAGCTCAAGGACCATCCAGATAGGACTGCTGTCTCTGGCCAGGGGAGCTGGTGTCAT
GACACCTTTAATGTGCCTCCTCACCAGCTTACCCTACTGCAAAACCAGAGTCATCCCTC
ATTCCTACTGCGAGCACATGGCCGTGGTGGAGCTGGCCTGCGCCGACCCATCTGTCAG
CGATCTCTACAGCCTCATCGTGGCAACACTATTGGTGGGGACAGACTCTGTCTTCATCA
CCTTCTCCTATGGTATGATCCTGAGGTCTGTGATGAGGCTGCCATCCCAAGAGGCACGT
CTCAAGGCCCTCAGGACCTGTGGGTCCCATGTTTCTATCATCCTGCTGTTTTACATAGGT
GGCCTACTCTCCATGTACCTGCAGATGTTCTCTTTTGGCTTGGCTCCTCATGTCCAAGTC
CTAGTGGCTGATTTCTATTTGACAGTCCCTCCCATGCTCAACCCCCTCATTTATGGCATA
AAGATGAAGCAGATCCAGGAAGGGATCTTCAAACTATTGGGGCAGTTGGCAAGACTC
AATATCTCACAAGCTGATAAAGACAGGTCAGATGGTGAGAGAAGGAGGCACTGCCAG

AAAATAATTTATCCCATCCTAGAATAG
>Ibis_OR2A2 (Ibis35)
ATGCAGAATGAAACGTCTGTCACAGAATTCATCCTCCTGGGGTTCTCCAGCAACCCAG
CCCTGCAGTTCTGCCTCTTTGCCATTTTCTCTGTCCTCTACTCTGCCACTCTGATGGGAA
ACGCACTTGTCTTTGTGCTTATCTGCCTGGACTACCGCCTCCACAGCCCCATGTACTTCT
TCCTCTGCCACCTCTCTATCGTGGACATCTGCTACGCCTCCAACAATGTCCCCCATATGC
TGAGGAACCTCCTTGGACAAGGCAGAACCATCTCCTTTGCTGGGTGTGGGACACAGAT
ACATCTTTATTTAATTTTTGCACTTACAGAGTGCGTGCTGCTGGCCGTGATGTCTTATGA
TCGCTATGTGGCAATCTGCCATCCACTCCGCTATGCCCTTATCATGAGCTGGAGGGTGT
GCCTCACCCTTGCTGCAGTTTCATGGGCTTTTGGGTTCTTATTTGGCACACTGCAAGCG
TCTCTGGCTTTACACCTGCCTTTCTGTGGCCCCTGCGAGGTTGACCACTTCTTCTGTGA
AATCCTTGCTGTCTTAAAGCTGGCCTGCACTGACACTACTGCCAATAAAGTCCTGATCT
TTGCTGTTTGCGTGTGCTTCCTCCTCTTCCCTTTAACCTTAATCCTAATTTCCTACCTGCA
CATCCTGGCCACCATTCTGCGTGTCCACTCTGTGGCAGGATGGCACAAAACTTTCTCCA
CCTGTGGCTCCCACCTGACCGTGGTGGGTCTGTTTTATGGAAACGCCATCTTCATGTAC
ATGGGGCCCAGGAGTAGTAACTCGTCTGGGAGGGAGAAAATTCTTTCCCTTTTCTACA
GTCTTGTCAGCCCAAGTTTGAACCCCCTGATTTACAGTCTGAGGAACAAGCAGGTGAA
AGAAGCCTTGCTGAAGCTTCAGAGAAGGAAAAGAGTTTTTCATTCCGTGTAG
>Ibis_OR5AU1 (Ibis36)
ATGGCTGACGAGAACTGCACCGAGGTGACCCAGTTCACCTTCTCGGGACTCACAGAG
CACCCACAACTGAAGCCCGTCCTCTTCACACTCTTCCTGGGCACGTACGTGCTGATGTT
AGCGGGAAACCTCGGCCTCATCGCCCTGATCAGGGTCAGCCCCCAGCTTCACACCCCC
ATGTACTTTTTCCTCGGTAACTTGTCCTTCTTAGACATTTGCTACTCCTCCACCATCAGC
CCCAAAATGCTGCTGGACCTTCCAACAAAAAACAAAACCATTTCTTTTGCTGGCTGCC
TCACGCAGTTTTATTTCTATGCTATTTTTGCCACAGCTGAGATTTACCTGCTGGCTGCCA
TGGCCTATGACCGGTATGTGGCCATCTCCAAGCCCCTGCTCTACGAGGCTATCATGTCT
CCTGGCATCTGCGTGAACCTGGTCGCTGTGTCCTACCTTGCAGGGTTGCTGAATGCCAT
CGTGCACACAAGCGCCCTGCTCCAGCTGTCCTTCTGTGGCCTCCCGGTCATCAACAAC
TTCTACTGCGACGGGCCACCGCTGTTTGTTGCCGCCTCCATCGACACGCGCCTCAATGA
GGGTTTAATGTTTGTGTTTGTGGGCTTCAACATGATAACCACCAACCTGCTCATCCTCA
CCTCCTACGCCTGCATCATGGTGGCCGTGGGCAGGATGGGCTCTGCTGCAGACAGGCG
CAAAGCCTGCTCCACCTGCGCCTCCCACCTGGCAGCCGTCATCATTTTCTATGTGTCTG
CTACTTTTAATTACATGCAACCCAGTTCATCAAACTCACTGGAGAGCAAGAAAATCGCA
TCCGTCTTTTACACCATTATAGTCCCCATGCTGAACCCTGTGATTTACAGCTTGAGAAAC
AAGGAGGTGAAGCATGCCCTGACCAGTTTTATCAGGAGGAAATTTCACTTCTGA
>Ibis_OR52K1 (Ibis37)
ATGTCCCACTCCTGTCCCACCAACACCTCGCTGTCAGAGCTGCACCTGACGGGCATCC
CGGGGCTGCAGCACCTGCACCACTGGATCTCCATCCCCTTCTGCCTCATGTACCTTGTC
ACGCTGGCTGGGAACAGCACCCTCCTGTGTGTGATAAAGGCTGATCCCAGCCTGCGCC
TGCCCGTGTTCCTCTTCCTGTCCATGCTGGCTGTCATTGACCTGGTGATGTCCACCTCCA
TCAGCCCCAAAATGCTGGGCATCTTCTGGTTTCAGTCCACTGCCATCAGCCTGGATGCT
TGTCTTACCCAGATGTACTTTGTTCATGCTTTCTCTGTGATGGAGTCAGGGGTGCTGGT
GGCGATGGCCTTTGACCGCTACGTGGCCATTTGCAATCCACTGCGGTACTTGTCCATCC
TGACCAGCCCCGTTGTGGCTGCCATTGGCTTGGCCACCTTGCTCAGGGCTGTGGTTTTC

ATGAGCCCCCTCACCTTCCAGATTCGCCGCCTGCCTCTTTGCAGCCCGGCAGTCGTGG
ACCACTCATACTGTGAGCACATGGCTGTGCTCAAACTGGCCTGCGGGGACACTGCCTT
CAGCAACACCTACAGCCTCTCTGTCTCCACCTACGTGGGCAGCTTTGATTCACTGCTCA
TCGCCCTCTCATACACGCTCATTCTCCGAGCAGTGCTCAGCCTCTCCTCCCCACAAGCC
CGCAAAAAAGCCTTCAGCACCTGCGGCTCACACCTCTGTGTCATGGCCCTCTTCTACAT
CCCCGGGCTGCTCTCCATGTACGTGGAGAGGTACCACCAGGAGCTCCCACCCCATGTC
CAGGTCATGTTGGCTGATCTCTACCTCCTTATCCCTCCGGCATTCAACCCCCTGATCTAT
GGCATCAGGATGAAGCAGATTCGTGATGGAGTACGCAGGGCGATCTCCCGGAGGAGA
CCCGTGGCAGGACGGATTGGGCATGGCCTTCAGGGCACAGGGCTGGGACTCATGAAG
ACAAAGTCCGTTCCCTAG
>Ibis_OR14A16 (Ibis38)
ATGTCCAACACCAGCTCCATCACTCAGTTCCTCCTCCTGCCATTCGCAGACACACAGG
AGCTGCAGCTCTTGCACTTCTGCCTCTTCCTGGCCATCTACCTGGCTGCCCTCCTGGGC
AATGGCCTCATCATCACCGCCATCGCCTGTGACCACCACCTCCACACCCCCATGTACTT
CTTCCTCCTCAACCTCTCCCTCACAGAACTAGGTTCCATCTCGACCACTCTCCCCAAAG
CCATGGCCAATTCCCTGTGGGACACCAGGGCCATCTCCTACTTGGGATGTGCTGCCCAA
GTCTTTTTTTTCTTTTTCTTTGTTACAGCAGAATTTTGTCTTCTCACTGTCATGGCCTATG
ACCGGTACGTAGCCATCTGCAAACCCCTGCACTATGGGACCCTCCTGGGCAGCAGAGC
TTGTGTCCACGTGGCAGCAGCTGCCTGGGGCAGTGCGTTTCTCAATGCTGTGCTGCAC
ACTGCCAATACATTCTCACTACCACTCTGCCAAGGCAATGCTGTGGACCAGTTCTTCTG
TGAAGTGCCCCAGATCCTCAAGCTCTCCTGCTCAGATGCCTACCTCAGGGAAGCTGGG
CTTCTTGTGATTAGCATCTGTTTATTCTGTGTGTGTTTTGTTTTCATTGCGGTGTCCTATG
TGCAGATCTTCAGGGCCGTGCTGAGGATCCCCTCTGAGCAGGGACGGCACAAAACCTT
TTCCACGTGCCTCCCTCACCTGGTCGTGGTCTCCCTGTTTGTCAGCACTGCCATGTTTG
CCTACCTGAAGCCCTCCTCCATCTCCTCCCCAGCCCTGGATGTGGTGATGGCAGTTCTG
TACTCAGTGTTTCCTCCAGCAGTGAACCCTCTCATCTACAGCATGAGGAACAAGGAGC
TCAAGGATTCCCTGTGGAAGCTGTTTGAATATGATCTATTTCAGATTAATAAGGTGCCCA
TTATTCCACTAGGGCTGCCACCGTATCTCAGGAAAACCCAGGACTAA
>Ibis_OR4M1 (Ibis39)
ATGGAGCATGAGAACTATACAGTAGTTACAGAGTTTATTCTGTTGGGATTGACTCAAAA
CCAAAAAGTCCAGATGATTCTCCTCTTCTTCTTCCTGCTCTTCTATATGATCATTCTGCC
AGGCAACGTCCTTATCATTCTCACAGTTTGGTGGGATTCCCAACTGGGATCACCCATGT
ATTTTTTCCTGGCCAATTTGGCATTCTTGGACATCTGCTACTGTTCTGTGACCCCGCCTA
AAATGTTGGCTGACTTTTTCTCACACCGTAAGACGATCTCCTACAGTGCCTGCATGGCC
CAGCTTTTCTTCCTCCACTTCCTGGGAGGAGCTGAAGCTTTTCTGCTCATGGTCATGGC
CTATGACCGTTATGTAGCCATTTGCAAACCTCTTCGCTACACTAAGCTTGTGAACAGGG
GGGTATGCTGTGTCCTGGTTGGAGCTACATGGGGTGCGGGCTTCATCCATGGCATCATT
CTATTTGCTCTCAGCATCCACCTCCCCTTATGTGGTCCCAACATCCTGGACAACTTTTTC
TGTGATGTCCATCAGCTGGTGAAGTTGGCCTGTGCCAACACTTACATAGTGGAGCTTTT
GATGTTCCTCAACAATGGAGTTGTTATAGTCATGTGCTTTACACTTCTCCTAATCTCCTA
CACTGTCCTGTTGCTGAAGCTCTGGACACAGTCCCCCAGGGCAAATAACAAAGTAGCT
TCTACCTGTGTTTCCCATATCATTGTCGTTTTTGTCATGTGTGGCCCAGCTATGTATCTCT
ATGGTTTACCCTTCCAAGCTGTCCCAATGGAAAAAGTTGTTGCTGTTTTCCATACAGTC
ATCTTCCCCTTGACTAATGCCATGATCTATACCTTGCGTAACAAGGAGATCAAAGACTC

GATGTGGAAGCTGATCAGCAAATACACACTTTGGTGTGGAAAATTAAAAAAAAATATT
TTGAAGTAA
>Ibis_OR6Y1 (Ibis40)
ATGGGTGGAAGGAATGAAACCAATGTCATGTATTTCATTCTCCTGGGTTTTCCCACCAC
TGCTGAACTCCAGCTGCTTCTCTTCTCTGCTTTACTTCTGGCTTATTTATTAACTGTGTT
GGAAAATTTCCTTATCATTCTCATCATCCGAAGTAATCACAGTCTGCAAAAACCCATGTA
TTTTTTCCTAGGAAATCTGTCTGTCTTAGAGATTTGGTATGTTTCTGTCATTGAGCCAAA
GATGCTCATAGATTTCCTCTCTCAAGACAAACATATCTCATTCCAGGGGTGCATGACAC
AGTTATATTTCTTTGTGACTTTTGTTTGTACTGAGTATATTCTGTTAGCTGTTATGGCCTAT
GACCGTTTCTTGGCCATATGCAAACCTCTCCGATATTCACTCATCATGAATCATCAGTTC
TGTGCTCAGCTGACAGCTGGCTGTTGGATGTGTGGTTTGATCACTTCTTCCATCAAGCT
AAGCTTTATAGCCCAGCTATCGTTCTGTGATGTAGACAAAATCAATCACTATTTCTGTGA
TATTTCACCCCTACTGAGTATCTCCTGCAGCGATTCCTCTTCGGCTGAGCTAGTGGACTT
CATCCTGGCTCTGATCGTCATCATGGTGCCTCTGTGTACTGTGGTCACCTCCTATATTTG
CATCATATTCACTGTGTTGAAGATCCCTTCTTCTCAGGGAAGGCAAAAGGCCTTTTCCA
CCTGCAGCTCCCACTTGACTGTAGTGATATTGTTCTACTCCACCACTCTTTTCACTTATG
CTCACCCTAAGGTCATGTATACCTACAGTGCTAACAAGTTGGTATCAGTCTTGTACACA
GTAGTTGTGCCACTTCTGAATCCTCTCATATATTGTCTTAGAAACAAAGAAGTCAGGTT
TGCCCTGAAGAAGACCTTTACTTGCACAAGACCTAAGAAGGGGCCTTGCTCATAA
>Ibis_OR4D5 (Ibis41)
ATGGCACTCAGAAACTTCTCCCGGGTGACTGAATTCATCCTCCTGGGGCTTTCTGATAC
AAGGGAGCTGCAAGTCGTCTTCTTCACCTTCTTCTTCCTGGCCTATGCCATGGTTCTGC
TGGGCAACCTTCTCATCATCGTGACATTCAGGACTGACCCCAAGCTGTCCTCACCCATG
TACTTTCTCCTCTGCAATTTGTCCTTCATAGATATCTGCTTCACTTGTGTCACCTCTCCCA
GGATGCTGGTGGACCTGCTCGCCCAGAGGAAGGCCATTGCGTTTGAAGACTGTATAGC
CCAGCTGTTTTTTCTGCACTTTGTTGGGGCATCAGAGATGTTCCTCCTGACTGTGATGG
CTTATGACCGCTACGCTGCCATCTGCAAGCCCCTGCACTACACAGCCATCATGAGCCGG
CCGGTGTGTTGGGTCCTGGTGTCTGCCTGTTGGGCAGGGGGCTTCCTCCACTCCATTGT
CCAGATGCTGCTCACAGTCCAGCTCCCCTTCTGTGGCCCTAACACAATCGACAGCTATT
TCTGTGACGTGCCTACTGTCATTCGGCTTGCCTGCACAGACACCTATGTCACCGAGTGG
CTCGTGGTTTCCAACAGCGGCTTAATATCCCTGGTTTGCTTCCTGATGCTGGTCACATCT
TACACGTTCATCCTGGTCACCATCAGGGTCCGCTTCACTGAGGAGCACTGGAAGGCGC
TCTCTACCTGTGCCTCCCATGTGATGGTCGTCACCCTCTTCTTTGTACCCTGCATCTTCG
TCTACCTCCGGCCCTTTTCTAACTTCCCCTTCAACAAGCACATCTGTGTGATCAACACC
GTCTTCTCCCCGGTGATGAACCCCCTCATCTATACCCTGAGGAATAACGAGGTGAAGGC
GTCCATGTGGAAATTGTGGAAGCGCTGCAGAGTCTTCTGA
>Ibis_OR12D2 (Ibis42)
ATGCTGAACCAGACAGAGGTCAGTGAGTTCATCCTTTTGGGCCTCACCGATATCCAGG
GGCTGCAGCACTTTTTCTTCATCTCCTTTCTGTTGCTCTACTTGACTGGTCTTCTGGGAA
ATGGTGCCATTGTGACCATGGTGATAGCTGAGCCCCGGCTCCACACCCCGATGTACTTC
TTCCTGGGGAACTTGTCCTGCCTGGATACTTTCTACTGCACAGTCACTGTTCCCAAGAT
GTTGACTGGCTTTCTCTTTGGGCATCAGCCCATCTCTTTTGGTGGGTGCTTGGCCCAGC
TCCACTTCTTCCACTTCCTGGGCAGTACTGAGGCTGTGCTCCTGGCCACCATGGCCTAC
GACCGCTATGTGGCCATTTGCAACCCTTTGCGCTACACCCTTGTCATGAGCCCACGGAC

TTGTCTGCTGCTGGCTGTGGCCAGCTGGTCCACTGGTTTGTACATGCCATGGTGCACT
CAGTCATGACCTCTCAACTGAGCTTCTGTGGCCACAACCACATTCATCACTTCTTCTGT
GACATCAAGCCACTGTTGAACTTGGCTTGCAGTAGTACCAGCCTCAACATGGCCCTCC
TCAATGTTGTCACCACGTCTATTGCTCTAGGCCCCTTCACTCTCATAGTCCTCTCCTATC
TCTATATTATCTCCTTCCTCTTCTATGAAGTCCGGTCCCAGGAAGAAAGATGGAAGCCC
TTCTCCACCTGCGCCTCCCACCTCACCGTTGTGGCACTGCTTTACATACCAGTGACCTT
CAACTATACACCACCTGCCTCAGGAAGCTTCCCTAAGAGGGATGTGCAAGTGTCTCTC
ATGTACAGTGCTGTCACCCCAGCTCTGAACGCCTTGAATTACACTCTTAGGAACCAGC
AGGTGAGATCTGCCCTGGAAAAAATGTTAGGGAGAAAACGCTTTCCTGGAGGAAAGT
GA
>Ibis_OR6A2 (Ibis43)
ATGGAGGTGAGCAATCACAGCATGGTCAAAGAATTCATTCTCCTTGGATTTCCCATTGC
TCCTCGCTTGCAGATTCCCTTCTTTGCAATATTTCTCATTGCATATCTCTTGGTTCTAGCA
GAAAACACTATCATCATCCTGACTGTCTGGACAAACTATAAACTCCACTCTCCCATGTA
TTTCTTTCTGAGTAATTTGTCCTTCCTGGAGATCTGGTATGTGACAGTCACACTCCCCA
AGACAATGCTGAGCTTTGTGTCAGTGAAAAAGCAAATCTCCTTCATGGGATGCATGAC
ACAGCTGTACTTCTTCCTTAGCCTGGGCAACACTGAGTGCCTCCTCCTGGCTGTCATGG
CATATGATCGCTATGTTGCCATTTGCAAGCCTTTCCATTATTCAACTATCATGAGACACA
CTGTCTGTGTCTACCTTACTATGGGATCTTGGTTGACTGGTTTCTTAATTTCTGGATGCA
AAGTATTTTTTATCTCTCAGCTAACATACTGTGGACCCAATATAATCAACCATTTCTTCTG
TGATGTTTCTCCTCTCTTGAACTTAGCCTGCACAAATATGGAAAGAGCTGCTCTTATGG
ATTTTGTCGCTGCCTTATTTATTCTCCTGATGCCTCTCTCTGTAGTAATCCTATCCTATACC
TATATTGTTTTCACTGTTGTTCACATTTCATCTGTACAAGGTTGCCAAAAAGCCTTCTCC
ACCTGTGCCACCCACCTTATAGTGGTCATTGTCTTCTATACGACAAGTATTTTCATCTAT
GTGAGGCCGAAAACACTTCCAGTTCATGACACAAACAAAATTGTGTCTGCTCTCTACG
CTGTTGTTGTTCCTCTCTTCAATCCTATCATTTACTGCCTGAGGAACCAGGAAATCAAG
GATGCTTTCCAAAAGATCTTGTTCAGAAAAAGAGTGTCCTTGCAGAAACATTAG
>Ibis_OR6B1 (Ibis44)
ATGGCTGAAGGGGAAAGGGACAACGGGACACCATCCACGGATTTTGTGCTGCTGGGA
ATAAGTTATGTCCCCACGCTCCAGAACCTGCTGTTTCTCCTCTTGCTTATGATCTACTCA
GTCACTGTGGTTGGGAACATCCTCGTCGTTGTACTGGTGGTGGCAGACCGGCATCTGC
ACACCCCCATGTACTTCTTCCTGGGCAATCTGTCCTCCTTGGAGATCTGCTACAGCTCC
ACCACCCTGCCTCAGTTGCTGGCCAGCTTCCTGACTGGGGACAGCAGCATCTCTGCTC
ACAGCTGCATGGCTCAGTTCTACTTCTTTGCTTCATTTGCAACTACTGAGTGTTACCTGC
TGGCGGCCATGTCCTATGATCGGTACTTGGCCATATGCCAGCCCTTGCTCTATGCGAGCC
TCATGACCTGGAAGGTCTCTTTACACCTGGCAGCTGCATCTTGGCTAGGGGGTTCACTA
TTGCTGGTGGCAGTCACAGTCTTCTTATCCCAGTTGAGGTTTTGTGGCCCCAAGGCAGT
TGACCACTTCTTCTGTGATTTTACCCCATTGCTGGAGCTCGCCTGCAGTGACACCAGAG
GGATCACACTTGTTTCTTTCATATTTGGCATCTTGGATGTAGTTTTTCCTTTTCTGTTCAC
ACTGGCCTCCTACATGTGCATCATGGCTGCAATCTGGAGGAAACCATCCAGCACAGGC
AGGCAGAAGGCCTTCTCCACCTGCTCCTCTCACCTCATCGTTGTTACTATTTTCTATGGC
ACCATCTTTGTTGTCTACATGCTGCCCAGGAGACCCTCGCTGAGGCAGCTCAACAAAG
TGTTCTCCTTTTTCTACACCATCGTCACACCCCTGGTCAATCCCCTCATCTACAGCCTGA
GGAACAGGGAGGTCAGGGAGGCCTTCAGGAAAGTGCTCAGGAAAGCACTGTCTGGC

ACCCAGAGCTACCTTGGTGACAGATCCAAAAGACATTTCTAG
>Ibis_OR14A16 (Ibis45)
ATGTCCAACAGCAGCTCCATCACCCAGTTCCTCCTCCTGGCATTCGCAGACACACGGG
AGCTGCAGCTCTTGCACTTCTGGCTCTTCCTGGCCATCTACCTGGCTGCCCTCCTGGGC
AATGGCCTCATCATCGCTGCCATCGCCTGTGACCACTGCCTCCACAACTCCATGTACTT
CTTCCTCCTCAACCTCTCTGTTCTTGACCTGGGCTGCATCTCTACCACTGTCCCCAAAG
CCATGGCCAATTCCCTCTGGGACACCAGGGCCATTCCGTACTATGGTTGCACTGCACAA
GTCTTTCTGTTACTCTGTTTTATTGCAGCTGAGTTGTATCTTCTCACTGTCATGGCCTATG
ACCGCTGTGTGGCCATCTGCAAACCCCTGCACTACGGGACCCTCCTGGGCAGCAGAGC
TTGTGTCCACATGGCAGCAGCTGCCTGGGGCACTGGGTTCCTCAATGCTCTCCTTCAC
ACTGCCAACACATTTGCACTACACCTCTGCAGAGGCAATGCTGTAGATAACTTCTTCTG
TGAAATCCCCCACATCCTCAAGCTCTCCTGCTCACACTCCTACCTCAAGGAAGTTGGG
CTTATTGTCATTAGTGTCTTTGTGGACTTTGGGTGTTTTATTTTCATAGTGGTGTCCTACG
TGCAGATCTTCAGGGCTGTGCTGAGGATCCCCTCACAGCAGGGACGCCACAAAGCCTT
TTCCACATGCCTCCCTCACCTGGCTGTGGTCTCCCTGTTTTTCAGCACTCTAGTGTTTGC
CTACCTGAAGTCCCCCTCCACCTTCTCCCCATCCCTGGATCTGGTGGTGGCAGTTCTGT
ACTCGGTGGTGCCTCCAGCAGTGAACCCCCTCATCTACAGCATGAGGAACCAGGAGCT
CAAGGATGCCTTATGGAAACTGGTCCAATGGATGCCCTTTCACTGA
>Ibis_OR6B1 (Ibis46)
ATGCCCTCAGATAACCTGACCCATGTGATTGACTTCATTCTGGTTAGTTTTCAAGGTAA
ACAGGAAATCAAGCTGCTGCTCTTTTTTATGTTCCTCCTGGCTTATGTGCTGACAGTGA
CAGAAAATGCAACGATTGTTGTGCTTGTTTGGACAAATCTCCAGCTTCACAAGCCCAT
GTATGTTTTCCTGGGCAATCTTTCCTTTCTGGAGATCTGGTACATCTCTGTCACAGTGCC
CAAAGTGCTTGTGAGCTTGGTAACAAAGAGACAAGGCATCTCCTTCACAGGCTGCATG
GCTCAGCTATTCTTCTTCCTGGCATTGGCCTGCAGTGAGTGCACTCTCTTGGCTGTCAT
GGCCTACGATCGCTATGTAGCCATCTGCAACCCATTGCATTACCCAATCATCATGGATCA
CACTCTTTGCACCCGTCTGGCCATTGGCTCCTGGATGAGTGGCTTCCTGATTTCCACAG
GGAAGATTTACTTCATTTCACGTCAGACCTACTGTGGGCCCAACATCATCAACCACTTC
TTTTGTGATGTCTCCCCCTTACTGAAGCTAGCCTGCACCAACATGTCAGTAGCTGAGCT
TGTGGATTTCTTACTGGCCCTACTCATCCTTCTTGTACCGCTCATTGTGATTATAGCCTCC
TATGCGTGCATCATCTCTGCTGTCTTGGGCATCCCCTCAGCCCAAGGGCGCCACAAGGC
CTTTTCCACCTGTGCCTCTCACCTTTTAGTGGTCATGGTGTTCTATACAGCCTCCCTGTT
TATCTATGCCAGGCCTCAACCTATTGATTCCTTCAGCTCCTACAAACTGGTTTCTGTGGT
ATACACTGTCCTGACACCCCTCGTCAACCCGGTCATCTATTGCCTAAGGAACCAGGAAT
TCAAAATTGCTCTTAGGAAAACAATATACTGGAGAGACATCTTATCCTAG
>Ibis_OR2AT4 (Ibis47)
ATGGAAAGCTGCAGCAGCAACGCTTCCACTAAAGTATTTTTCTTGGTGGGGTTTCCAG
CTCTCCAGGATTTCCAGACCCCCCTCTTTGTTGTGTTCTTGCTATTCTACCTGCTGATCC
TGGTTGGTAACGCTGTTATCATCACCGTGGTTGTGGTCGACCGTACGCTCCACAAACCC
ATGTACTTTTTCCTGATTAACCTCTCTGTGTTAGATGTGCTATTCACAACCACCACCATC
CCCAAAATGCTGGCAATGTTCCTGGCCAACGCTAAAACCATCTCGTTTCGGGGCTGTTT
TCTGCAGATGTACAGTTTTCACGGGTTGACGGTAACCGAGGCGCTTCTCCTGGTGGTC
ATGGCTTATGACCGCTACGAAGCCATCTGCAACCCCCTCCATTACCCGGCCAAGATGAC
AAGAAGAGTGAACATCCAGCTGGCAGCGAGCGCCTGGATCACTGCGCTGCTAATACCC

GTGCCCGTCATCATGCAGACTTCTCAGCTAGCTTACGGGGACACAACCACGGTTCACC
ACTGCTTTTGTGACCACCTGGCAGTGGTACAAGCCGCGTGCTCGGACTTCCGTGCCAA
TTTCCAGACCTTCTTGGGGTTCTGCATCGCTATGACAGTGTCGGTCATCCCTCTGTTGCT
CGTCACCCTCTCGTACGTCCACATCATCCTCTCCGTACTAAAGATCAACTCCAAAGAAG
GACGCGTGAAAGCTTTTTCTACGTGTACTTCCCATCTGCTTGTGGTAGGCACTTACTAC
TCCTCCATCGCTGTGGCTTACATGTCCTACAGAGCGGACATCCCCGTTGATGTCCATGT
CATGAGCAATGTTGTCTTCTCTATTTTAACTCCCTTGTTAAACCCCATCATTTACACTTTA
CGGAACAAGGAAGTAAAATCTGCGGTTAAAAAGTCTATTCTTCTGAAAATCTTTCCCTT
TTCTAAAAAATTTAATTTATTTGGGTAA
>Pelican_OR52B2 (Pelican1)
ATGCCACCATTCAACCTTACCAGCTTGACGCCAGCAACATTCATCCTGGCTGGCATCCC
AGGCATGGAGAAGTTCCACATCTGGATCTCGATCCCTTTCTGCTCCATGTATCTGGCTG
CCCTGCTAGGAAACGGTGTTATGTTGTTTGTCATAAGGACGGAACGGAGCCTGCACCA
GCCCATGTACCTCTTCCTGTCCATGCTGGCCATCGCTGACCTGATGCTGTCGACCACGA
CTCTGCCCAAGATGCTGGCTCTGTTCTGGTTCAGCGCTGGGGAGATTTCCTTCGGCGCC
TGCCTGACCCAGATGTTCTTCCTGCATTTCAGCTTCTCGGCAGAGTCGGTGATCCTGCT
GGCCATGGCGTTTGATCGGTTTGTTGCTATCTGTTACCCCCTGTGGTATGTGGCGGTGCT
GACCCACTCAGCCGTTGTCAAAATGGGGCTGGTGGCTTTGCTGAGAAGTTTCTGTATC
ATTTTCCCGTGTATATTTCTTTTGAAAAGGCTGCCGTTCTGCGGACACAATGTCATCCCG
CACACCTACTGCGAGCACATGAGCATCGCCCGGCTGGCCTGTGCTGACATCTCCGTCA
ATATCTTGTATGGCCTCGCAGTGCCTTTTGCAGCAATAGTGGTAGATGTTGTACTCATTG
CTGTCTCCTATGTCTTAATTCTTCTGGCATTATTCAGACTCCCTTCCAGGACTGCCCGTC
ACAAGGCTTTCAACACCTGCGGCTCTCATGTCTGTGTTATATTACTTTTCTATATTCCTGC
TTTTTTCACTGTTTTAACGCACCGCTTTGGTCAGCAAATCCCCCGCCACATCCACATTCT
GCTGGCCAACCTGTATGTGCTCTTCCCCCCGCTGCTGACCCCTGTCGTGTATGGTGTGC
GGACACAACAGATAAAAGAGAAGGTTGTGAAGGTGTTCATCTGCCCCAAGAGTCATTT
GCTGCAAGAATAA
>Pelican_OR5AR1 (Pelican2)
ATGATCAGAAGAAACAAGACAACTGTTGATGAGTTCATTCTCTTGGGAATCACAGATAT
TTGGGAGCTGCGGGTCATTCTCTTTGTGCTGTTCCTTCTGATTTGTGTCGCCTCATTGGT
GGGGAATCTTGGCATGATTGCATTAATCAGGCTTGACTCTCGACTCCACACCCCCATGT
ACTTCTTCCTCTGCCACCTCTCTCTGGTAGACCTAGGCAATTCCTCAGCAGTTGCTCCC
AAAATGCTAGTGAGCTTCTTTGAAGAAAGGAAAGCCATCTCTCTGCCAGGGTGTGCAG
CCCAGATGTACTTTTGTGGAGTCTGCATAATCACTGAGTGTTACCTGCTGGCTGCGATG
GCTTATGACCGGTACGTGGCCATCTGTAAGCCTCTGCTCTACGTAGCCACCATGTCCCA
AAAGGTTTGCATCCAACTGGCTGTGGGATCCTACATAGTAGCTGCTGTGAATGGAATAG
TGCTTGTCAGCTCAGTGTTCAGTTTACACTTCTGTGGCCCTAATGTCATCAATCACTTCT
TCTGTGACATTCTTCCGCTCCTGAAACTGTCCTGCTCCAGTACTACTGTCAATGAACAT
GTGCTTTTTATCATGGCTACTTTTATTGCGCTGGGCACTTTAGTATTCATTGTTGTTTCTT
ATGGTTATATCCTTACCGCTGTCCTGAAGATCTGCTCCTCAGAGGGCAGGCACAAAGCT
TTCTCCACCTATGCCTCACATTTGTCATCAGTCACAGTTTTTTACGGAACTATGATCTTC
ATGTACGTCCGCCCCAGTTATAGCTACTCCCTGGCCCAGGAAAAAGTGGTGTCTGTTGT
CTACATCTTGGTGATTCCCATGCTGAACCCCCTGATCTACAGCCTGAGGAACACAGAG
GTGAAGGAGGCTCTCAAGAGACACCTAGAAAAAGTTCTTGTTTCCTTTAGAAATCAAA

CTGGTAAAGAGGTGCCATGA
>Pelican_OR6B1 (Pelican3)
ATGAAGCAGAAAAACAGCACAAAATCCCAGGAATTTATCCTCTTGGGATTTCCAACTA
CCATGGAACTTGAAATGTTGCTCTTCGTGATATTCCTGGTGGCCTACATGCTGACGATCT
TTGAAAATATACTCATAATTGTCTTGATAAAGATGAACCATCAGCCTCACAAGCCCATGT
ATTTCTTTCTCAGCAACCTCTTCTTCCTGGAGGCTTGGTACATCTCAGTCACGGTCCCT
AAACTGCTCATGAATTTTCTTGTGGAAAGCAAGAATATATCTTTTGGAGGCTGTATCAC
CCAGCTTTACTTCTTCAGCTCCCTTATTTGTACTGAGTGTGTCCTCCTTGCAGTCATGGT
TTATGATCGCTATGTGGCCATTTGCAATCCCCTGTGCTATCCAGTCATCATGAACCACCG
GCTCTGCATGCAACTAGCTACGTGCTCCTGGCTCATTGGCTTCTTGGCCTCCATGCTGA
AAGTATTTTTCATCTCTCAGCATTCTTTTTGTGGCTCCTATGTCATCAACCACTTCTTCTG
TGACATCAGCCTCTTGCTGAACATATCATGTGCTGACATGACAATGGCTGAAATAGTGG
ATTTCATCCTGGCCTTGCTTATCTTGCTGGTTCCCCTCTCTGTCACAATTATCTCCTATATA
TGCATCATCAGCACCATCCTGCATATCCCCACAGCCCAGGGCAAGAAGAAAGCCTTGT
CCACTTGTGTTTCTCACCTCACTGTAGTCATTATCTTCTTTTCAGCCACTCTGTTTATGTA
TGCACGGCCCAAAAGAATCCACCCTTTTGACTTAAACAAGCTGGTGTCAATTGTGTAC
ACTATTGTAACTCCTATGCTTAATCCCTTCATTTACTGTCTGAGGAACCAGGAGGTTAAA
CAGGTTGTGAAAAAAATTTTCATTGTTAGACAGACTGCCCCCAAGGTCTCTCAATCCAT
TGCTGTCCACCTTTAA
>Pelican_OR14J1 (Pelican4)
ATGGAACAAATATCCAACGGCAGCTCCATCACTGAGTTCCTGCTCCTGGCGTTTGCAG
ACACGCGGGAGCTGCAGCTCCTGCACTTCTGGCTCTTCCTGGGCATCTACCTGGCTGC
CCTCCTGGCCAACGGCCTCACCGTCGCCGCCGAAGCCTGCGACCACCGCCTCCACACC
CCCATGTACTTCTTCCTCCTCAACCTCTCCCTCCTCGACCTGGGCTTCATCTCCACCACT
GTCCCCAAATCCATGGCCAATTCCCTCTGGGACACCAGGGCCATCTCCTATGCAGGATG
CGCTGCCCAGATCTTTCTGTTTCTCTTCTTCACTGGGGGAGAGTGTTCCCTTCTCACCA
TCATGGCCTACGACCGCTACCTTGCCATCTGCAAGCCCCTGTGCTACGGGACCCTGCTG
GGCAGCAGGGCTTGTGTCCACCTGGCAGCGGCTGCCTGGGGCAGTGGGATGCTCAAC
GCTGCCCTGCACGCGGGCAACACCTTTTCACTACCGCTCTGCCAAGGCAACGCCCTGG
ACCAGTTCTTCTGTGAAATCCCCCAGATCCTCAAGCTCTCCTGCTCAGACTCCTACCTC
AGGGAAGTTGGCCTTCTCGCGTTCAGTGCCTCTTTTGCTTTTGTGTGTTTTGTTTTCATC
CTGCTGTCCTACGTGCAGATCTTCAGGGCCGTGCTGAGGATCCCCACCAAGCAGGGGC
GGCACAAAGCCTTCTCCACGTGCCTCCCTCACCTGGCCGTGGTCTCCCTGTTCCTCAG
CACCATAATGTTTGCCAACCTGAAGCCCCCCTCCATCTCCTCTCCATCCCTGGATCTGGT
GGTGGCTGTGCTGTACACGGTGGTGCCTCCAGCAGCAAACCCCCTCATCTACAGCATG
AGGAACCAGGAGCTCAAGGAGGCCCTGAAGAAATTGATTTCATAG
>Pelican_OR52K1 (Pelican5)
ATGCCCCACTCACGTCCCACCAACACCTCGCTGTCAGAGCTGCATCTGACGGGCATCC
CAGGGCTGCAGCACCTGCACCACTGGATCTCCATCCCCTTCTGCATCATGTACCTCATC
ACGCTGGCTGGGAACAGCACCCTCCTGTGCGTGATAAAGGCTGATCCCAGCCTACACC
TGCCCATGTTCCTCTTCCTGTTCATGCTGGCTGTCATTGACCTGGTGATGTCCACCTCCA
TCACCCCCAAAATGCTGGGCATCTTCTGGTTTCACTCCACTGCCATCAGCCTGGATGCT
TGTCTTACCCAGATGTACTTTGTTCACGCTTTCTCCGTGATGGAGTCGGGGGTGCTGGT
GGCGATGGCCTTTGACCGCTACGTGGCCATTTGCAATCCACTGCGGTACTTGTCCATCC

TGACCAGCTCCGTTGTGGCTGCCATTGGCTTGGCTACCTTGCTCAGGGCTGTGGTTCTC
ATGAGCCCCCTTACCTTCCAGATTCACCACCTGGCTCTCTGCAGCCCGGCAGTCGTGG
ACCACTCGTACTGCGAGCACATGGCCGTGCTCAAACTGGCCTGTGGGGACGCTGCCTT
CAGCAACACCTACAGCCTCTCCGTCTCCACCTATGTGGGCAGCTTTGACTCGCTGCTCA
TCGCCCTCTCATACGTGCTCATCCTCCGAGCTGTGCTCAGCCTCTCCTCCCCACAAGCC
CGCAAAAAAACCTTCAGCACGTGCGGCTCACACCTCTGCATCATGGCCCTCTTCTACAT
CCCTGGGCTGCTCTCCATGTACATGGAGAGGTACCACCAGGAGCTCCCACCCCACGTC
CAGGTCCTGTTGGCTGATCTCTACCTCCTCATCCCACCGGCATTCAACCCTCTGATATAC
GGGATCAGGACAAAGCAGATTCGTGATGGAGCATGCAGGGCGATCTCCTGGAGGAGA
CCCATGGCAGGAGGGATTGGGCCTGGCCTTCAGAGCACAGGGCTGGGACTCATGAAG
ACAAAGTCTGTTCCCTAG
>Pelican_OR10A7 (Pelican6)
ATGAAGCCAACAGAGGGACCAGAACCAGGAAACCACACTCTGCTGGCCAAGTTTGTC
CTCTCTGGATTGTCCAACCACCCAGAACTGCAGCACTTGCTGTTCTTCACATTCTGTTT
AATTTACACCATCACCATCATTGGGAACCTTCTCATCTTCATGGTCACAGTGCATCCCAC
CCTCCGCATGCCCATGTACTTCTTCCTCCGGGTCCTGTCCTTCCTGGATATTTCCACAGC
ATCGATCGTTGTTCCCAAGATGCTGGTAAACTTCCTGTCGGAGGACAGGAGCATTTCCT
ACATGGGCTGTGCCACACAGCTCTACTGTGTGATTTTCTTAGCAGCCACTGAATGCTAC
ATTTTGGCAGCCATGGCCTATGACCGTTATGTGGCCATATGCAACCCCCTTAGATATGCA
ATCATCATGAACAGCAGAGTTTGTCTTTCCTTGGTTCTGCTGTCATGCTGCAGTGGTAA
TGTTGTGTCTGTGGTGCAGACAGCTTGGGTGTTCACATTGCCATTTTGTGGGCCCAAG
AAGATTAACTACTTCTTCTGTGATATTCCCCCACTCATCATGCTCTCCTGCACTGACACC
TCTTCGTATGAAAAGCAGATCATTACAGCCACAGTGCTGGTCATCTTTATACCATTTTGT
CTCATCCTGGTATCCTATGCCTGCATCATCTCCAGCATCCTGAAGATTTCCTCTGCAGAG
GGCAGACATAAGGCCTTCTCCACCTGTTCCTCACACCTTACTGTTGTAACACTCTATTAT
GCAAGTGGGACCTTGATTTACTTACGGCCAAAATCCAGCAATTCACAAGACACTAAGA
AAGTCCTAGCTCTCATGTATACAACCATAATTCCCACATTAAACCCCCTGATTTACAGCC
TGAGAAATAAAGAAGTGAAAGGAGTACTAATCAAAATTATAGCTGAGCTGAGGAAGAC
TTAA
>Pelican_OR10K2 (Pelican7)
ATGGAGAGGGACAATAAGAGCACTGATGCAACCATGGAGTTCCTCCTGCTCGGCTTCT
CCGAGCTGCTCTGTCTGCGGGTCCTCCTCTTCCTTATCTTTCTCATTGTCCATTTGGTCA
CGTTGGCAGGGAATGCGATGATCTTCATGGCAGTGGTTATGGAGCCTTCCCGTCCTCCC
ATGCTTTTCTTCCTCTGTCAGCTCTCTGTCATCGAGCTCTGCTATACCTTAGTGATTGTC
CCTAAGGCACTTCTCAGCCTGATAGTGGTGGATGGCAGCACAATTTCTTTCATAGGCTG
TGCTGCACAGATGCACCTTTTTGTGGCACTTGGTGGGGCTGAATGCTTCCTCCTGGTAG
CCATGTCATATGACCGTTACGTTGCCATCTGTCAGCCACTTCACTACGTAGCTGTGATGA
GTGAGGGGCTCTGCTTCAGGCTGGCTGTGGCATGCTGTCTGGGAGGCTTTGCTGTTGC
CCTGGGGTTGACAGTGGCTGTTTTCCGCTTACCTTTCTGTCAGTCGCATCGTATCAACC
ACTTCTTCTGTGATGTCCCTGCTGTGCTGCACCTGGCCTGTATACAGAGTTATACCCGTG
AGCTGCCCTTGCTGGCTGCCTGTGTGCTCCTTCTGCTGCTCCCCTTCCTCCTAATCCTG
ACCTCGTATATTTGCATTGCTGCTGCTTTGTTACATGTCACCTCCTCCATGGGAAGGGGA
AAGGCCTTTTCCACCTGCATTTCACACTTGGCCATCACCTTGCTACACTATGGATGTGC
CACTTTCATGTACATTCGTCCTAAATCCAGTTACTCGCCAGCTCGAGACAAGATGGTGT

CTCTGGTCTACACCAATGTTACTCCATTACTGTATCCCCTCATTTACAGCCTGAGGAACA
AGAAAATCAGAGGGGTCCTCAGGAAAATGTTGAGGAGGAAGAAAATAGCTAGCTGA
>Pelican_OR5J2 (Pelican8)
ATGGCGGAGCAGAATCACACCTCAGTGGCAGAGTTTGTTCTCGAGGGCCTGAGTGAC
CAAGCGGAGATGAAGGCAGCTCTCTTTGTGCTGCTCCTGCTCATCTACACCCTCACCCT
TTTGGGCAACGCGGGGATAATCCTCGTTATCCAAGGTGACCCACGACTCCACACATCCA
TGTACTTCTTTCTTGGCAGCCTCTCCGTTGTTGACATCTGCTTCTCTTCTGTGATTGCCC
CCAGGACCTTGGCGAGCTTCCTATCGGAGAGGAAGACCATTTCCTTCGTTGGCTGCAT
GGGCCAAGCTGCCTTCTACATCGTCTTAGTGACGACTGAGTGTTTCCTGCTGGCCGTCA
TGGCGTACGACCGGTACGTGGCCATCTGCAACCCCCTGCTCTATTCCTCTGTCATGACT
CGGAGGTTGTGCGTGTGGCTCGTGGTGGGGTCCTACATTGGGGGTGTCCTAAACTCCA
CCATACAGATGACCTTCATCGTTAGGCTGCCCTTCTGCAGCTCCAATGTCATCAACCAC
TTCTTCTGTGACGTTCCTCCCCTCCTGGCTCTGTCCTGTGCCAGCACCTACGTCAACGA
GATGATCCTCCTCTCCTTGGCCGGTGTCATTGAGCTCAGCACCATCTCCACCATCCTGG
TCTCCTACATCTTCATCTCCTTTGCCATCCTGAGGATCCGTTCAGCTGAAGGCAGGCAA
AAAGCCTTCTCCACCTGCGCATCCCACCTGACAGCAGTGACCATGTTGTATGGACAA
CAATCTTCATGTATTTACGTCCCAGCTCTAGTTACTCCCTGAACACTGACAAAGTGGTC
TCCGTCTTCTACACGGTGGTGATCCCAATGCTGAACCCCCTCATCTACAGCCTGAGGAA
CCAGGAAGTGAAGGATGCTTTGAGGAGAGCAGCAGAAAGAATCAGTCAGGGTCTGAA
CCCTCCTCAATAA
>Pelican_OR6C4 (Pelican9)
ATGAACCACACAACAGTAGTGGAATTTGTCCTCTTGGGGCTGACCAACAGCCGCCATT
TGGAGATCATCCTCTTTCTCTTTCTTGTGATCGCCTATTTCTTGATCCTGCTTGGAAACA
TTACTGTCATCAGCATCACTTTTATGAATCATTTCCTTCAGACCCCAATGTACTACTTCCT
CAGAAATTTTGCCCTTTTGGAAATCACTTTCACCTCCACATTCATTCCCAGCACCCTCTA
CAGCCTTTTGACAGAGAGGAAGATGATTTCCCTGCCTGGTTGTTTCCTTCAGATGCTGC
TTTTCTTTTACTTGGGTACCTGCACATTTTTCCATGTGGCAACAATGTCCTTGGATCGGT
ATGTTGCTATTTGCCGCCCTTTGCATTACACAACAATTATGAACAACAGATTTTGCCTCC
AGCTGGTCCTGGCTTGCTGGGCAGTGAGTTTTCTCTTAATGTTTCCTCCCACCATTATGA
TTGTCCAGTTGCCATTCTGTGGTCCCAATGTCATGAACCACTTTTACTGTGATACTTCCC
TGTTGTTGCAACTGTCCTGCACAGACACAGGGTTCATTGAAGGACTGATGCTTATCATA
CTAATTATCATCATACCTGGTACCTTAATAGTGACTGCTGTTTCTTATGGCTGTATTATTAT
CAGTATCTTGCATATACCATCTTCCACAGGTAGGAAGAAAGCATTTTCCACTTGCTCAG
CTCACCTCATGGTGGTGATGATATTTTACAGCACATGTATTTACAGGTATATCCGCCCAG
CACAGCGAGGTGGGCAGGACTCTGACAAAGTTCTTTCTTTCTTCTTCTCTGTGGTGAC
TCAGATGCTTAACCCATACATCTACTCACTCAGGAACAATCAAGTCAAACAAGCCTTAA
AGGAGGGCATGTTGAAGGCATTTTCTAGCTCCCTAAGGTAG
>Pelican_OR5AP2 (Pelican10)
ATGGCTTGGGAAAACTGGACTACGGTGACAGAATTTGTTTTCAAGGGGTTCACGGATC
GCCTTGACTTCCAAGTTACCCTCTTTGTCATTTTCCTGCTCATCTATGTCATCACTGTGG
TGGGAAACCTTGGCATCATTGCTGTAGTCTGGCTTGATTCCCACCTTCAAATGCCCATG
TACTTCTTTCTCAGCAACTTGTCCTTCTTGGATCTGTGCTACTCTTCAGTTGTGACACCC
AAAATGCTGCTTAACCTCTCATCAGAAAGGAAGACTATTTCTTTCGCTGGCTGCTTCAT
ACAGCTGTATTTCTACGCTGCTTTTGTAACTGTGGAGTGTTACCTCCTGGCCGCGATGG

CATGCAATCGCTATGTTGCCATCTGCAACCCGCTGTACTACCCCATCGTCATGTCCCAGA
AGGTCTGCGTTTCCCTCTTGGCTGGGTCCTACATAGCTGGGTTTGTGAATTCAGTGGTG
CTCACGGGGTTTGCGCTGAGAGTATCCTTCTGCGGCCCCAATGTCATTGACCACTTCTT
CTGTGATGGCCCACTGCTGTCAAAACTGGCCTGCTCTGACACCCGCCTCAACCAAGTG
CTGGTACTTGCTTTTGGGGGCTTCAATGAGGTCACCACCATATCAGTCATCCTCATATCC
TACGGCTGCATCCTATTCACCATCCTGAGGATGGGCTCTGTGCTGGACAAGTGCAAAG
CTTTTGGTACCTGTGCATCCCACCTGGTCGTCATCACCATCTTCTACGGGACCCTTCTCT
TCATGTACCTGCTGCCCAGCTCCAGCTACTCCCTGGGGAGGGACAAAATTGTTTCTGTC
TTTTATGCGGTGGTGACCCCCATGCTGAACCCCTTCATCTACAGCCTGAGGAACCAGG
AGGTGAAGAGTGCTCTGAAGAGAGCAGTGGGGAGAATAATTACTTCCCTGCTGAAGT
GGTAA
>Pelican_OR13A1 (Pelican11)
ATGCATCCACAGAATCTCACCAAGGTTTCTGAATTCATCCTTGAGGGTGCTTTTGAAAA
CCCTCACTTCCGAGGTCTCTATATTGCAATATCTTTGTGTCTCTACCTCACTGCTATCCTC
AGCAACTCATTTGTCATTATAGCTACCATTGTCCACCCACCACTTCACACCCCTATGTAC
TTTTTCATCTCCAACCTGGCTATCCTTGACTTGGTAGGTGTCTCCTCAGTTCTGCCCAAG
ATGTTGGAGAACCTGATCCTGGACAAGAACACCATCTCCTTTGAGGGCTGTGTAGCAC
AGCTCTACACCTTCACTTTCTCGGGCTCAACAGAGCTTATGCTGTTAACAGTCATGTCT
TATGACCGGTACCTGGCCATCCGCCAGCCCCTGCACTACATAACCATGATGAGCAAGGG
GACATGCATCTCTTTAATGGCTGGTGTCTGGGGAATTGGTACCATCAATTCCCTCATAAA
CACCCTTCTTGTGGCACAGCTGGACTTCTGTGGGCCAAATTTAGTCCAAAATTTCTTGT
GTGAGATACCCCCAGTCCTTGCCCTGTCCTGCAGCTCAACCTATCTCAGTGAAATCACA
GTTTACTTGGCAGACATCATCTTAGGCATGGGAAACTTCTTGCTGGTCATCCTCTCCTAC
TGCCTCATCATCATCACCATCCTGAAAATCCAGGGCTCTGCAGGGAAGTGGAAAGCCT
TCTCCACCTGCTCCTCACACCTTGCTATTGTTGGCTTGTTCTACTCCACCATCATCTACA
CCTATATCCAGCCAACCAGCACTCCATTAGAGAAGAAGAACAAAACAGTGAGTGTTGT
GTACACTCTGGTGACTCCCACTCTGAACCCTTTGATCTACAGCCTGCGCAACAAAGTG
ATCAAAGCAGTGTTCTGGAAAATTGTACCATTTCCCAAGACAAACTAA
>Pelican_OR4S2 (Pelican12)
ATGGAGAATGCAAGCAGTGTGAAGGAATTCATTCTTCTGGGCCTCTCAGAGAATCAAG
GGGTGCAGAAAATATGTTTTGTGATGTTTCTGTTCTTCTATATGATTACTGTGCCAGCAA
ATCTGCTCATTGTTATCACTGTAATTAGCAGTCAACGTCTGAACTCCCCCATGTATTTCT
TCCTCTGCTACTTGTCCTTTGTAGATATCTGTTACTCTTCTGCCACAGCTCCCAAAATGA
TTGCTGACTTTCTCGTTGAAAATAAAACCATCTCCTTTGTGGGTTGCATAGCACAGCTG
TTTGCGGTACATTTCTTTGGCTGCACAGAGATCTTCATCCTCACAGTGATGGCCTACGA
TCGCTGCATTGCCATCTGCAGACCTCTCCACTACACCACCCTCATGACCAGGCATGTGT
GTGGCTGGATGGTCATCGGCTCGTGGGTAGGAGGCTTCATGCACTCTGTAGTGCAGAC
TCTTCTAACCACACAGCTCCCCTTCTGTGGCCCTAACAAAATTGACCACTACTTCTGTG
ATGTCCACCCCCTACTACAACTGGCCTGTACCGACACGTATGCTGTGGGCATCATTGTC
GTTGTCAACAGCGGAATGATAACTCTGAGCTGTTTCTTCATCCTGGTCATGTCCTATGTT
TTCATCCTGGTTTCCTTGAAAAGTCAAACATCCAAAGGATGGCATAAGGCCCTCTCCAC
CTGTGGGTCCCACATCACTGTGGTGATTCTGTTCTTCGGGCCATGCATGTTCATCTACAT
GCGTCCGTCCAGCAATCTATTGAAGGACAAGAGCATGGCAGTGTTTTACACTGTCATC
GCACCCATGCTGAACCCACTCATCTACACGCTAAGAAATGAGGAGGTAAAGAGTGCCA

TGAGAAAACTGTGGAGTAGAAAAGTGGGAAGTGAAAATGGAAAAGTGTAG
>Pelican_OR6Y1 (Pelican13)
ATGGGTGGGAGGAATGAAACCAATACCGTGTATTTCATTCTCCTGGGTTTTCCCACCAC
TGCTGAACTGCAGCTGCTCCTCTTCTCTGCTTTACTTCTGGCTTATTTATTAACTGTTTT
GGAAAACTTCCTTATCATTTTCATCATCAGAACTAACCACAGTCTGCAAAAACCCATGT
ATTTCTTCCTAGGGAATCTGTCTGTCTTAGAGATCTGGTATGTTTCTGCCATTGAGCCAA
AGATGCTCATAGATGTCCTCTCTCAAGACAAACATATTTCATTGCAGGGGTGCATGACA
CAGTTGTATTTCTTTGTGACTTTTGTTTGTACCGAGTATATTCTGTTAGCTGTTATGGCCT
ATGACTGTTTCTTGGCCATATGCAAACCTCTCCAATATTCACTCATCATGAATCATCAAT
TCTGTGCTCAGCTGACAGCTGGCTGTTGGATGTGTGGTTTGATCACTTCTTCCATCAAG
CTGAGTTTTATAGCCCAGCTCTCATTCTGTGATGTAGACAAAATCAATCACTATTTCTGT
GATATTTCACCCCTACTGAATATATCCTGCAGTGATTCCTCTTCGGCTGAGCTAGTGGAC
TTCATCTTGGCTCTGATCGTTATCATGGTGCCTCTGTGTACTGTGGTCACTTCCTATATTT
ACATCATATTCACTGTGTTGAAGATCCCTTCTTCTCAGGGGAGGCAAAAGGCCTTTTCC
ACCTGCAGCTCCCATTTGACTGTAGTGATACTATTCTACTCCACCACTCTTTTCACTTAC
GCCCACCCTAAGGTCATGTACACCTACAGTGCTAACAAGTTGGTATCAGTCTTGTACAC
AGTAGTTGTGCCACTTCTGAATCCTCTCATATATTGTCTTAGAAACAAAGAAGTCAGGT
TTGCTCTGAGGAAGACCTTTATTTGCACAAGACACACCTAA
>Pelican_OR5AU1 (Pelican14)
ATGGCTGATGAGAACTGCACCGAGGTGACCCAGTTCACCTTCTCGGGACTCACAGAGC
ACCCACAACTGAAGCCCGTCCTCTTCGTGCTCTTCCTGGGCACCTATGTGCTGACATTA
GCGGGAAACCTCGGCCTGATCGCCCTGATCAGGGTCAGCCCCCAGCTTCGCACCCCCA
TGTACTTTTTCCTCGGTAACTTGTCCTTCTTAGACATTTGCTACTCCTCCACCATCAGTC
CCAAAATGCTGCTGGACCTTCCTGCAAAAACCAAAGCCATTTCTTTTGCTGGCTGCCT
CACACAGTTTTATTTCTACGCTGTTTTTGCCACAGCTGAGGTTTACCTGCTGGCTGCCAT
GGCCTATGACCGGTACGCGGCCATCTCCAAACCCCTGCTCTACAAGGTTGTCATGTCTC
CTGGCGTCTGCATGAGCCTGGTGGCTGTGTCCTACCTTGCAGGGTTGCTGAATGCCATC
GTGCACACAAGCGCCCTGCTCCGGCTGTCCTTCTGCGGCCCCCCAGTCATCAACAACT
TCTACTGCGACGGGCCGCCGCTGTTTGTTGTCGCCTCCACCAACACGCGCCTCAATGA
GGGTTTAATGTTTGTGTTTGTGGGTTTCAACATGATCACCACCAACCTGCTCATCCTCA
CCTCCTACGCCTGCATCGTGGTGGCTGTGGGCAGGATGGGCTCTGCTGCAGGCAGGAG
CAGAGCCTTCTCCACCTGCGCCTCCCACCTGGCAGCTGTCATCATTTTCTATGTGTCTG
CTACTTTTAATTACATGCGACCCAGTTCATCAAACTCACTGGAGAGCAAGAAAATCGCA
TCCATCTTTTACACCATTATAGTCCCCATGCTGAACCCCATGATTTACAGCCTGAGAAAC
AAGGAGGTGAAGCATGCCTTAATCATTTTTATCAGGAGGAAAATGTACATCTGA
>Pelican_OR51E2 (Pelican15)
ATGCCCTTCCCCAACAGCTCTGACCTCAGCCCATCCTCCTTCATCCTGGCCGGCATCCC
GGGGATGGAGGCTGCCCATTTCTGGATGGCAATCCTTCTGTGCTCCATGTACATCTTGG
CAGTCACAGGCAACTGCGTGGTGCTGTTCATCGTGAAGACGGAGCCCAGACTGCATAT
TCCCATGTACTTCTTTCTCTGCATGCTGGCTGCCATCGACCTGGCTTTGTCCACAGCCA
CGGTGCCACGCGTTCTCTCCTTCTACTGGTTCAACACCAGGGAGATCAGCTTTAGCGCT
TGCCTTGTCCAGATGTTCCTCATTCACACCCTCTCAGCCATCGAGTCCACTGTCCTCCT
GGCCATGGCTGTAGACCGGTACATGGCCATCTGCCACCCACTGAGACACGCTGCCATC
CTCACTAACACTGCGATAGTGAAAATAGGGCTGGTAGCCATGGCCAGGGGAGTCCTTT

TCTTCCTGCCTTTGCCTTTGCTCCTCCTGCCCCTTCCATTCTGCAGCTCCAGGGTGCTGT
CACACTCCTTCTGCCTGCACCAGGACGTGATGAACCTGGCCTGCAACCACACCACCCC
CAGTGTGGTGTATGGCCTCACCGCCATCCTGCTGGTCATGGGGCTGGACGCCATCCTCA
TCTGCCTCTCCTACGTCCTGATCCTCAAGGCTGTCTTGCGGCTGGCATTGTGGAAGGAG
AGGCTCAAGGTGTTCAGCACCTGCGTTGCCCATATCTTCGTGGTCCTGGCCTTCTACGT
GCCCCTGATTGGGCTGTCCGTGGTGCACAGGTTTGGGAAGGACCTGGCTCCACTGGTC
CATATCACCCTGGGGAATGTTTACATCCTAGTGCCTGCTGTGCTCCACCCCATCATCTAC
GGGGTGAGGACCAAAGAGATACAGAGGAGGATCCTGAATTTAATTCATATATACAACA
ACAGAACTGCCCAGTGA
>Pelican_OR14J1 (Pelican16)
ATGTCCAACAGCAGCTCCATCACTGAGTTCCTGCTCCGTGCTGAGTTCCTCCTCCTGGC
ATTTGCAGGCACACGGGAGCTGCAGCTCCTGCACTTCTGGCTCTTCCTGGGCATCTACC
TGGCTGCCCTCCGGGCAATGGCCCTCATTATCACCGCCATAGCCTGTGACCACTGCCTC
CACACCCCCATGTACTTCTTCCTCCTCAACCTCTCCCTCCTCGACCTGGGATCCATCTCC
ACCACTGTCCCTAAAGCCATGGCTCATTCTTTGTGGGACACCAGGGCCATCTCCTACTC
AGGATGTGCTGCCCAAGTCTTTCTGTTTATCGTTTTCATTTCAGCAGAGTATTATCTTCT
CACTGTCATGGCCTACGACCGCTATGTTGCCATCTGCAAACCCCTGCACTACGGGACCC
TGCTGGGCAGCAGAGCTTGTGTCCACATGGCAGCAGCTGCCTGGGGTGGTGGGTTTCT
CTATGCTTTGCTGCACACTGCTAATACATTTTCACTACCCCTCTGCCATGGCAATGCTGT
GGGCCAGTTCTTCTGTGAAATCCCACAGATCCTCAAGCTCTCCTGCTCAGATGCCTACC
TCAGGGAAGTTGGACTTATTGTGTTTAATCTCTTAGTAGTATTCAGCTGTTTTGTTTTCA
TTGTGCTGTCCTATGTGCAGATCTTCAGGGCAGTGCTGAGGATCCCCTCTGAGCAGGG
ACGCCACAAAGTCTTCTCCATGTGCCTCCCTCACATCACCGTGGTCTCCCTGTTTATCA
GCACTGCCATGTTTACCTACCTGAAGCCCCCCTCCATCTCCTCCCCATCCTTGGACCTG
GTGCTGGCAGTTCTGTACTCGGTGGTGCCTCCAGCAGTGAACCCCCTCATCTACAGTAT
GAGGAACCAGGAGCTCAAGGATGCAGTGGGGAAACTAATAACTGGATGTTTTTCTGAA
GCAAAAAACTTCTTCTGTAGAGCAGCTATAATGTACCTCAATACAGGCCCAGCCTATTG
TCTGTAA
>Pelican_OR2D2 (Pelican17)
ATGGCCAGGGAAAACCAAAGCGTAGTGACAGAATTCATCTTTCAAGGCCTTTCCTCCC
AGCCAAGGACACAGACTGTTCTTTTCATAGTGTTCCTGTTTTTTATCTGTTCACAATTG
TGGGGAACATCATGATCATTACAGTGATCAGAGCTGACTGCCAGTTGCAGTCACCCATG
TACTTTTTCCTTGCCAGTCTGTCCTTCTTAGATATTGGCTACGTCTCCAGCAACATCCCA
CTGATGCTGGTGAACCTCTTGACCAAGAAGAGGACCATCTCCTTCTCTGGATGCGCTG
CTCAGATGTATTTCTCTCTGGCTTTTGGCATGACAGAGTGTGTTCTGCTTGGGGTCATG
GCCTATGATCGATATATGGCAATATGTCACCCCTTGCTCTACACCACTGTCATGAACAGG
AAGGTTTGCATTCACATGGTCGTGGCTTCCTGGACCAGCAGCCTGCTGAGCTCCATGG
TCATCAACAGCCTCACCTTGCGGCTGCCCTTTTGTGGGCCTGACATCTTGAACCATTAC
TTCTGTGAAGTGCTAGCAGTGCTGGCCTTGGCCTGCACTGACACTGCCCTCATGGAGA
TGGTCATCTTCATCTTCAGCATCATCATAGTCTTCATCCCCTTTCTATTGATCATCACCTC
ATATGCCCATATCCTTTCCGCCATCTTGAAGATCCAATCTGCACGTGTGCAATCCAAGGC
CTTCTCCACCTGCGGATCCCACCTGATGGTGGTGAGCATATTCTATGGGACGGCCATCT
GCGTGTACATGAATCCTAAGTCAAGGCCTCCACAGGACAGGGACAAAGTGGTTGCAG
TGTTTTACACCATTGTAGCCCCAATGCTGAACCCCCTCATCTACAGCCTCAGGAACAAG

GACATGAAGCGCGCCCTGAGAAGGGCAATGAATAGGCCCAAATCCCTGTTTATTTAA
>Pelican_OR5AP2 (Pelican18)
ATGGTTGAAGATAATTATACATTCGCATCCGAGTTTATTCTCCTGGGCTTCACAAACCGA
GAAGACCTGCAGGTGACATTCTTTGTCTTATTCCTTGCCATCTATGTGGTCACTCTAGTA
GGAAATCTGGGAGTAATTATATTAATCAGAATCGATTTGTGCCTACACACCCCCATGTAC
TTCTTCCTAAGCCACTTGTCTCTCCTGGACATCTGCTACTCCTCCACCATCCTCCCTCAA
ACCTTGCTGAATTTTTTAGTCGAGAAGAAGGTTATTTCCTTCATTGGGTGTGCCACTCA
GCTCTTCTCCTTTGCGACTTGTGCCACTGCCGAGTGCTACGTGCTGGCTGCCATGGCTT
ATGATCGCTACGTGGCCATTTGTAACCCCCTGCTCTACTCCGTGGTCATGTCCCAGAGG
CTTTGTGTTGGGATGTTGGCTGCTGCCTACTTAGCTGGTGTGATCAGCTCCACCATACA
CACAGTTTCCATATTTCGTCTCCCATTCTGCCGGTCCAAGAGGATCAATCATTTCTTCTG
TGATGGACCACCGCTGCTAGCCCTCTCCTGCTCAGACACCCATGTCAATGAAGTGATG
GTTTCTGCCGTGGTGGGGTTCAATGTGCTAAGCACCATGGTCTTCATTTTAGTCTCCTAC
TTGTCGGTCCTCTCCACCGTCTTGCGGATGCGCTTCAAGGCCGGTTGGCACAAAACCT
TCTCCACTTGTGCCTCTCACTTGGTCTCCATTGCTTTGTACTACGGCAGCTCCCTCTTCA
TGTACCTGCGCCCTGGCTCCAGACACTCCTTGGAGCATGACGAGGTGGTGTCCATGCT
GTACGCCATTGCAGTCCCCATGCTGAACCCGCTTATCTACAGCCTAAGAAACATGGACA
TGAAGAACGCCGTGAGGAAAGCAAAAGGTAGAGTCCTCTCCTCCTTGTCCATCCACG
GTTCCTCGTCAGCTGAAAGGAGAGGGCTACACTTCCATGGTAAGGAGGGTTAG
>Pelican_OR51F2 (Pelican19)
ATGTCATCTTTCAATAGGACTGGGTCCAGTCCCCTGACCTTCATCCTCACTGGGATTCCT
GGTTTGTCAATGAGCAGCTACTGGACGGCATTGCCTTTCTGCTGCCTGTACCTCCTCAT
GCTCCTGGGGAACTGCACCTTACTCTGGATGATAAAGACAGACCACAGCCTCCATACA
CCGATGTACTATTTCCTCTCCATGCTGGCCATGGCAGACCTGGGCTTGTCTCTCTCCACC
CTACCCACCATGCTGGCCATTTTCTGGTTTGAGTCCACCTCCCTCCATTTTGAGGCGTG
CATTGTCCAGATGTACTTCATCCACTCCTTCTCTGTCATCGAGTCCGGGGTCCTGGTGG
CCATGGCCTTCGACCGCTTTGTTGCTATTTGCTACCCTTTGCACTATGCCTCTGTCCTGA
TGAGCTCCCTGATAATGAAGGCAGGGGGGGCAATCTTCATGCGGGGCATTTGTGTGGT
GCTACCTGCTGCCATCCTTATTAAGAAGATGACTTTCTGTGGGTCCCATGTCCTGTCTCA
CTCCTTCTGTCTGCACCAGGACGTGCTGAGGCTGGTTTGTGGGGATGTCAGGGTCAAC
AGCTTGTATGGGCTGACTGCAGTGTTATTCACCAAGGGCCTGGACTCCCTGACCATCCT
CCTGTCTTACACGATGATCATCAGAGCCATCTTGAACATTGTCTCCCAGGAAGCACGAG
CCAAAGCCTTTAGCACGTGCATCTCCCACCTCTGTGCTGTCCTGATCTTCTACATCCCC
CTCATTGGCTTGTCCATCATCCACAGGTTTGGGAAGCATCTGCCCCCTCTCACTCACAC
GCTCCTGGCTGATGCCTATCTCCTGGTGCCGCCTGTCCTGAACCCGCTCGTGTACAGCT
GGAAAACCAAGCAGATCCGCAGGCGGATCCTCATCCTGCTCTGTCGGAGAGAGACCC
AGCAGCAGGTCTAG
>Pelican_OR52K2 (Pelican20)
ATGTCAACCACCAACCAATCCAACACCAACTCTTCGCCTTTTGTCCTGACGGGCATCCC
TGGCCTGGAAGCTCTCCACGTGTGGATTTCCATCCCGTTCTGCTTTACATACATCATGAC
CTTGCTGGGAAATAGCATGGTCCTTCTCACTGTGAGGCTGAACAAAAGCCTCCATGAG
CCTCTATACTATTTCATTTCCATGTTGGCTGTCATTGACCTCATCTTCTCAACTGCTGTAA
TTCCCAAAATGCTGGGTGTATTCTGGCTGGATTCAAGGGAGATTGGTTTTGAGGCTTGC
TTCATCCAGATGTTCTTCATCCACACATTCACTGCAGTGGAGTCAGGGGTGCTCCTGGC

AATGTCCTTTGACCGATATATAGCCATCTGCAACCCCCTGAGATACAACTCCATCTTAAC
GAGCTCAAGGACCGTCCAGATAGGACTGCTGTCTCTGGCTAGGGGAGCTGGTGTCATG
ACACCTTTAATGTGCCTCCTCACCAGCTTACCCTACTGCAAAACCAGAGTCATCCCTCA
TTCCTACTGCGAGCACATGGCCGTGGTGAAGCTGGCCTGCGCCGACCCGTCTGTCAGC
GATCTCTACAGCCTCATTGTGGCAACACTATTGGTGGGGACAGACTCTGTCTTCATCAC
CTTCTCCTATGGTATGATCCTCAGGTCTGTGATGAGGATGCCATCCCAAGAGGCACGTC
TCAAGGCCCTCAGGACCTGTGGGTCCCATGTTTCCATCATCCTTCTGTTTTACGTCGGT
GGCCTACTCTCCATGTACCTGCAGATGTTCCCTTTTGGCTTGGCACCTCATGTCCAAGT
CCTGGTGGCTGATTTCTATTTGACAGTCCCTCCCATGCTCAACCCCCTCATTTATGGCAT
AAAGATGAAGCAGATCCAGAAAGGGATCTTCAAACTATTGCGGCAGTTGGCAGGACTC
TATCTCACAAGCTGA
>Pelican_OR5AR1 (Pelican21)
ATGGCCAAAGGAAATTGCAGCCCCAGTGCTGAATTTGTTCTCTCAGGGTTCTCAGAGC
AGGGGGCTGCCCAGGCTGTCCTCTTCATGGTCTTCTTGGTGATCTACCTGATCACTCTG
CTGGGGAATCTGGGGATGCTTGTGTTAATCAGGCTGGATGCCCAGCTTCACACCCCCAT
GTACTTCTTCCTGAGCAGTCTGTCCTTCCTAGACATCTGCTACTCCTCCTCAATCACCCC
CAGACTGCTCTCGGATCTCCTAGCAGAAAGAAAGGTCATTTCTTACTCTGCGTGCCTCA
CACAATTTTATTTCTATGCGGTCTTTGCCACCACCGAGTGCTACCTCCTGGCTGCGATGG
CGTATGACCGCTACGTGGCCATCTGCAGCCCACTGCTCTATGCCACCTCCATGTCCAGC
AGAGTTTGTGTGCTGCTGGTAGGTGGCTCGTACCTCACTGGGATCGTGAATGCCACTAT
CCACACAGGGTTTGCACTTCGGCTGTCCTTCTGTGGTCCCAACATCATCAACCACTTTT
ACTGTGAGGGGCCCCCGCTTTACGCCATCTCCTGCACGGACCCCACCATCAATGAGATT
GTGATGTTCCTTGTGGTTGGCTTCAACCTATTTGTCACCAACCTGACCATCCTCATCTCC
TACGCTTACATCCTGGCCACCATCCTGAGGATGCGCTCGGCCACAGGCAAACACAAAG
CCTTCTCCACGTGTGCATCGCACCTGACCACCGTGACCCTCTTCTACGGATCTGCTGCG
TCCATGTACTCACGACCCAGCTCCCGGCACTCCCAGGATCTCGACAAAGTGGCCTCTG
TGTTTTACACTGTGGTGACCCCCATGCTGAACCCCCTCATCTACAGCCTGAGGAACAA
GGAGGTGAAGAATGCTCTGGGGAGAGTGCTGGAGAGGTTCGTCTAA
>Pelican_OR6B1 (Pelican22)
ATGAAGAACCAAACTGCTATCAATGAGTTCATTCTTCTGGGATTTTCCTACGGGCTGCA
GGTCCAGACCTTACTTTACCTGGTCTTTCTGGTCACCTACATGGTAACAATCACTGAGA
ATGCAATCATCATCTTTGTGGTGAAAAGGAACCACCACCTCCAAAAACCCATGTATTAT
TTTCTGGGGAACTTGTCGTTCCTGGAGATTTGGTATGTCTCAGTAACATTGCCTAGGCT
TTTGTTTGGATTCTGGTCACAGAGCATGACCATCTCATTCTCCAGCTGCATGACCCAGT
TATACTTTTTTATTTCCCTTATGTGCACTGAATGTGTCCTTTTGGCTGTAATGGCCTATGA
CCGCTATTTGGCTGTCTGCCATCCTCTGCGCTACCAAGCCATCATGACCTATAAGTTGTG
CTTTCAGCTGTCAATTCTCTCATGGGCAGGAGGCTTTTCCATTTCCTTGGTCAAAGTGT
CTTTTATTTCACGCCTCACTTTTTGTGGTCCACAAGTCATAAACCACTTCTTTTGTGACA
TCTCTCCAGTCCTGAACCTTTCCTGCACTGACATGTCCCTTGCAGAGACAGTGGACTTT
GCATTAGCCTTGGTGATCCTGCTAGTGCCTCTCTTGATCATTGTTTTCTCCTACTGCTGTA
TCTTGTCAGCTATTTTGTGTATGCCTTCAGCCCAGGGAAGGAGAAAAGCCTTTTCCACT
TGTACCTCCCATTTCACTGTAGTCATTATCTTTTTCTCAGCTACTCTCTTCATGTATGCCA
GGCCCAGGAGGATCCATCCCTTCAACCTCAACAAAATAGTGTCTGTCTTTTATGCTGTA
TTCACTCCAGCACTGAACCCTCTAATCTATTGTCTGAGGAACAAGGAAGTGAAAGAGA

TTCTGAGAAAGATCATAGGCACAAGCTGCTCTGCACACCAATAA
>Pelican_OR4D5 (Pelican23)
ATGGCACTGGGAAACTTCTCCCGGGTGACCGAATTCATCCTCCTGGGGCTTTCTGACA
CAAGGGAGCTGCAAGTCCTCCTCTTCACCACCTTCTTCCTGGCCTATGTCATGGTTCTG
CTGGGGAACCTTCTCATCATTGTGACAGTCAGGACTGACCCCAAGCTGTCCTCACCCA
TGTACTTTCTCCTCTGCAATTTGTTCTTCATAGATATCTGCTGCACCTCTGTCACCTCTCC
AAGGATGCTAGTGGACCTGCTCTCCCAGAGGAAGGCCATTGCGTTTGAAGAATGTATA
GCCCAGCTGTTTTTTCTGCACTTTGTTGGGGCATCGGAGATGTTCCTCCTGACTGTGAT
GGCATACGACCGCTACACTGCCATCTGCAAGCCCCTGCGCTACACAACCATCATGAGC
CGGCGGGTATGCTGGGTCCTGGTGTCTGCCTGCTGGGCAGGGGGCTTCCTCCACTCCA
TTGTCCAGACGCTGCTCACAGTCCAACTCCCCTTCTGTGGCCCTAACACAATTGACAG
CTACTTCTGTGACGTGCCTCCCGTCGTTCAGCTAGCCTGCAGAGACATCTATGTCACCG
AGTGGCTCATGGCTTCCAACAGCGGCTTAATATCCCTGGTTTGCTTCCTGGTGCTGGTC
ACATCTTACACATTCATCCTGGTCACAATCAGGGCCCGCTTCACTGAAGGGCACTGGA
AGGCGCTCTCTACCTGTGCCTCACACATGATGGTTGTCTCCCTCTTCTTTGTACCCTGCA
TCTTCATCTACCTCCGGCCCTTTTCTACCTTCCCCTCCGACAAGCACATCTGTGTGATCT
GCACTGTCTTCTCCCCAGTGATGAACCCCCTCATCTATACCCTGCGGAATAACGAGGTG
AAGGCATCCATGTGGAAATTGTGGAAGCGCTGCAGAGTCTTCTGA
>Pelican_OR10A7 (Pelican24)
ATGTGCCCCAAATCCCTGCAGCAGGGAAATCTCAGCAACCCCACCATGTTCCTTCTCCT
GGGATTTTCCAGTGCCTACAGAGCACAGGTGACCCTCTGCCTGTGCTTCTCACTCATTT
ACCTGGTGACAGTGCTGGGGAACCTGTTCATCGTGACCCTCGTCTGTCTGGATGCCCA
CCTGCACTCCCCCATGTATTTCTTCCTGGGCCACCTCTCCTTCCTGGACATCTGCTACTC
CTCCGTCACGCTCCCTAAGATCCTTGGAGACTCCTTCTCACCGCAGAAGACCATCTCCT
TTGTGGGCTGCATTACACAGATCTACTTCTTCCTTTGCTTTGGGGGCTCTGAGTGCATG
CTCCTGGCTGCCATGGCCTACGATCGGTACCTGGCCATCTGCCACCCCCTCCACTACCT
GATACTCATGAGCAAGAAGATGTGCCACTGCTTAGTGGCCATTTCGTGGCTGAGTGGC
TCCTTCTCATCCCTGATCCAGGCCTTCCTCACAGCCCACTTGCCCTTCTGTGGATCCAA
CATGATTGACCACTTGTTCTGCGAGACACCCTTCTTGCTGAAGGCATCCTGCAGCCCTA
GTGCTCCCCTCAACAAGGCCGCCTTGTATGTTTTTTCTGGGACTATTGCGATGGGCTCT
TTCCTCCTTACTCTCACATCGTATGTGCACATCATCACGGCTGTCCTTCAGAAGGGAGC
AGGGATGCAGAGGGCCTTTGCCACCTGCACTTCCCACCTGACTGTGGTGTCCCTGTTC
TTCGGCGCCGGGGCTGTTGTGTACCTGGTGCCTCACTCCAGTGGCTCCAAGGAGATGG
ATGAAGTCCTTGCCCTGCTGTATGCCGTTGTGACCCCCATGCTCAACCCCATCATCTAC
AGCTTGAGAAACAGCGAGGTCAAAGGGGCCATCAGGAAAGCCCTGCACAGGGGGGTA
TTACAGACATCCACCAAAGGTGCAGGCATCACTGCTGAGTAA
>Pelican_OR52I1 (Pelican25)
ATGGCTTCTGATCCCTTCAACCACCCCAACAGCAGCTCCTCCTCTTTCACCCTTGTGGG
TGTCCCCAGCCTGGAAGCTTTTGCCACCTGCCTGGGCATCCTTTTCTGCTCAGGCTATA
TCATCGCCTTGGTAGGAAATGGTGTAGTTTTGCTTGTCATTGGGCTGGACCACTCCCTG
CGTGACCCCATCCATTGCTTCCTGGGCATGCTGGCGGTCATCGATGTGGTGATGGTGAC
ATCCATCATCCCCAAGATGCTGAGCGTGTTCTGGCTGAACTCTACGGAGATTGGTTACA
CAGCCTGTTTTGTTCAGATGTTCCTTGTTCACTCCACAACATCAGAGGAGTCGGGAGT
GCTCCTGGCCATGGCTGCTGACCGCTACGTTGCAATTTGTCACCCCCTCAGGTACCAAG

CCATCTTGAATCACCAAACAATTGCCCAAATAGGCCTGGCCATTGTGGTGAGAGCTCTC
CTTTTCATGATCCCCTTGACAGGGATGGTGACAAACCTCCCCTATTGCCGTTCCCGTGT
GATTCCCCATTCATACTGCGAGCACATGGCCGTGGCGAAGCTGGCATGTGCAGACCCC
AGACCCAGCAGGCTTTACAGTGTGGCTGGGTCCTCTCTTATAGTAGGGATGGACATGG
CTTTCATTGCTGTGTCCTATGGGATGATCCTTAAAACCGTCCTGGGGAAGAAATCATGC
TGGAAGGCCTTCAGCACCTGCGCGTGTCATATCTGCGTGATACTGCTGTATTACATCCCT
GGGATAGTCTCCATATACATACAGCAGTTCGGCAGCACAGTAGCCGTGCATGCACAGGT
CCTGCTGGCTGATCTCTACCTGACCATCCCCACCATGCTGAACCCTGTTGTTTACAGCA
TGAGGGCCAAGCAGATCCGGCAGGCAGTGCTCAAAATGCTGTTTTCCAGGAAGGTTCA
TACCTGA
>Pelican_OR14J1 (Pelican26)
ATGTCCAACAGCAGCTCCATCACTGAGTTCCTCCTCCTGGCATTTGCAGACACGCGGG
AGCTGCAGCTCCTGCACTTCTGGCTCTTCCTGGGCATCTACCTGGCTGCCCTCCTGGGC
AACGGCCTCATCATCACTGCCATAGCCTGCGACCACCGCCTCCACACCCCCATGTACTT
CTTCCTCCTCAACCTCTCCCTCCTTGACCTGGGCTCCATCTCCACCACCGTCCCCAAAT
CTATGGCCAATTCCCTCTGGGATATGAGGGATATTTCTTACTGTGGATGTGCTACCCAGG
TCTTTTGGTTTCTCTTCTTTGTTTCTTCAGAATATTATCTTCTTACTGTCATGGCCTATGA
CCGCTATGTAGCCATCTGCAAACCCCTGCACTATGTAACCCTCATGGACAGCCGAGCTT
GCGTCAAAATGGCAGGAGCTGCCTGGGGTGGTGGTTTCCTCAATGCGGTGCTGCACAC
TGGAAGCACATTTTCAATTCCATTCTGCCAAGGCAATGCTGTGGATCAGTTCTTCTGTG
AAATCCCCCAGCTCCTCAAGCTCTCCTGCTCAGACTCCTATGTCAGGAAAGCTGGGTTT
ACTGTGGTTAGTCTTTGTGTAGCTTGTGGGTGTTTTGTTTTCATTGTTCTGTCCTATGTG
CAGATCTTCATGGCTGTGCTGAAGATCCCTTCTGCGCAGGGACGGCACAAAGCCTTTT
GCACGTGCCTCCCTCACCTGGCCGTGGTCTCTCTGTTTGCCAGCTCTGCCATGTTTGCC
TACCTGAAGCCCCACTCCATCTCATCTCCAGCTCTGAATCTGGTGGTGGCTGTTCTGTA
CGCGGTGGTGCCTCCAGCGGTGAACCCCCTCATCTACAGCATGAGGAACCAGGAGCTC
AAAGATGCGCTACAGAGACTGATCCACTGTCTGCTGTTGCAGCAGGGATAA
>Pelican_OR14J1 (Pelican27)
ATGTCCAACAGCAGCTCCATCACTGAGTTCCTCCTCCTGGCATTTGCAGACACGCGGG
AGCTGCAGCTCCTGCACTTCTGGCTCTTCCTGGGCATCTACCTGGCTGCCCTCCTGGGC
AACGGCCTCATCATCACTGCCATAGCCTGCGACCACCGCCTCCACACCCCCATGTACTT
CTTCCTCCTCAACCTCTCCCTCCTCGACCTGGGCTCTGTCTCCACCACTCTCCCCAAAT
CCATGGCCAATTCCCTCTGGGACACCAGGATTATTTCCTACTATGGATGTGCTGCCCAG
GTCTCTATGTTTGTCTTGTTTGTCACAGGAGAGTTTTATCTTCTCACCATCATGGCCTAC
GACCGCTACCTTGCCATCTGCAAGCCCCTGCACTATGGGACCCTCCTGGGCAGCAGAG
CCTGTGTCAAAATGGCAGCAGCTGCCTGGGGCAGTGGGTTTATCAGTGCTGTGGGGCA
CACTGCCAATACATTTTCACTACCACTCTGCCAAGGCAGTACCATAGACCAGTTCTTCT
GTGAACTTCCCCAGCTCCTCAAACTCTCCTGCTCAGATGACTACCTCAGGGAAATTTG
GCTTCTTGTGTTTTGTTTTTCTTCAGGTATCGGGTGCTTTATTTTCATTGTTCTGTCCTAT
GTGCAGATCTTCAGGGCTGTGCTGAGGATCCCCTCTGAACAGGGACGGCACAAAGTG
TTTGCCACATGCCTCCCTCACCTGGCCGTGGTCTCCCTGTTTATTGGCACTGCCATGTTT
GCCTACCTGAAGCCCTCCTCCATCTCCTCCCAGTCCCTGAATCTGGTGGTCGCAGTTCT
GTACTCAGTGGTGCCTCCAGCTGTGAACCCCCTCATTTACAGCATGAGGAACCAGGAG
CTCAAGGATTCCCTGAGGAAACTGTTTTTATGGACATTTTTCTGTAGTCATAAAGTTCCC

ATCACTCTCTAG
>Pelican_OR2AT4 (Pelican28)
ATGGAAAGCTGCAGCAGCAACGCTTCCACTAAAGTATTTTTCTTGGTGGGGTTTCCAG
CTCTCCAGGATTTCCAGACCCCTCTCTTCGTTGTGTTCTTGCTATTCTACCTGCTGATCC
TGGTTGGTAATGCCATCATCATCACCGTGGTTGTGGTCGACCGTACGCTCCACAAACCC
ATGTACTTTTTCCTGATTAACCTCTCTGTGTTAGACGTGCTATTCACAACCACCACCATC
CCCAAAATGCTGGCAATGTTCCTGGCCAACGCTAAAACCATCTCGTTTTGGGGCTGTTT
TCTGCAGATGTACAGTTTTCACGGGTTGACGGTAACCGAGGCGCTTCTCCTGGTGGTC
ATGGCTTACGACCGCTACGAAGCCATCTGCAACCCCCTCCATTACCCGGCCAAGATGAC
AAGAAGAGTGAACATCCAGCTGGCAGCGAGCGCCTGGATCACCGCGCTGCTAATACCT
GTGCCCGTCATCACGCAGACCTCTCGGCTAGCTTACAGGGACACAACCAGGGTTCACC
ACTGCTTTTGCGACCACCTGGCCGTGGTACAAGCCGCATGCTCGGACTTCAGCGCCGA
TTTCCAGACTTTCTTGGGGTTCTTCATCGCTATGTCAGTGTCGGTCGTCCCCCTGTTGCT
TGTCACTCTCTCATACGTCCACATCATCCTCTCCATACTAAAGATCAACTCCAAAGAAG
GACGCATGAAAGCTTTTTCAACATGTACTTCCCATCTGCTTGTGGTGGGCACTTACTAC
TCCTCCATCGTTGTGGCATACATGTCGTACAGAGCAGACATCCCTGTTGATGTCCATGT
CATGAGCAACGTTGTCTTCTCTATTTTAACTCCCTTGTTAAACCCTATTATTTACACTTTA
CGGAACAAGGAAGTAAAATCTGCAGTTAAAAAGTCTATTTTTGTGAAAATCTTTCACCT
TTCTGAAAAAATTAATTTATTTGGGTAA
>Pelican_OR5V1 (Pelican29)
ATGCAGAGGCTCAACCTATCAACGGTATCTGAATTTGTTCTTGTAGGCCTTTCTGATGCT
CCACAAGTCCGTTTTCTTCTCTTTGTGCTGTTTTTGATCATTTATTTGGCCACCATGGCA
GGTAACATCGCAATCCTTGTTGCCATTAGCACAGACACTCATCTGCACAACCCCATGTA
CTTCTTCCTTGGCAACTTATCCTTACTGGATATCTTATGTCCCACTGTCACTGTGCCAAA
GATGCTGGAGGCCTTGTTGCTTGAGAACAAGGTGATTTCATTCACTGGCTGCACACTC
CAGCTGTTCTTCCTCATTGATGCCGTAGGCACAGAGATTTTTCTTTTGGCTGTGATGGC
GTATGACCGCTATGTTGCAATATGTCATCCGCTGCAGTACATGAATATCGTGAGCATGAA
ACTGTGTGCCCACCTAGCCATTGGCACCTGGGTAGTAGGATTTTTTAATTCTCTGTTGC
ACACATCTTTGATTTTTACACTCTTTTTTTGTGATTCTAATGAAGTTGACCAATATTACTG
TGATATTCCTCCTATGCTGACCCTCTCCTGCTCACCTACTTACAGTAGGGAACTGGTACT
TCTCACAGTTGCTGGGGTCCTTGGAAGCAGCGCCTTTGTGGTCACTCTGATGTCATATA
TCTGCATCCTCTTGGCTATCCTGGGCATGAACTCTTCTGACAGCAGGCACAAAGCTTTC
TCCACTTGTGGTTCTCACTTGACAGTAGTATGCCTTTTCTACGGGACCACCATTTGCAC
ATATGTACGGCCTTCCTCCACCTACTCACCTAATCAGGATAGGATAATTTCTATGCTCTAT
GGAATCCTCACTCCCCTGCTAAACCCCATAATCTACAGTCTGAGGAACAAAGAAATTA
AATGTGCCCTAGGAAGAGTGATCAGCCAGATAAGAACTGCTTTAACAAGACGAGAACA
TCTATCTCAGTCTCTGGTGTCCTCTGGAGCCCTAGCAAATGGATTGGCACTGCAGTTTG
ACTGA
>Pelican_OR4M1 (Pelican30)
ATGGAGCATGAGAACTATACAGTAGTTACAGAGTTTGTTCTGTTGGGATTGTCCCAAAA
CCATGAAGTCCAGATGATTCTTTTCTTCTTCTTCCTGCTCTTCTATATGATCATTCTGCCA
GGCAATGTCCTTATCATTCTCACAATTCAGGGGGATTCCCAACTCGGATCACCTATGTAT
TTTTTCCTGGCCAATTTGGCATTCTTGGATATCTGCTACTGTTCTGTGACCCCACCCAAA
ATGTTGGCTGACTTTTTCTCACATCATAAGACTATCTCCTACAGTGCCTGCATGGCCCAG

CTTTTCTTCCTCCACTTCCTGGGAGCAGCTGAAGCTTTCCTGCTCATGGTCATGGCCTAT
GACCGTTATGTAGCCATTTGCAAACCTCTTCATTATAACAGGCTTGTGAACAGGGGGGT
ATGCTGTGTCCTGGTTGGAGCTACATGGGGTGGGGGCTTCATCCATGGCATCATTCTATT
TGCTCTCAGCATTCACCTGCCCTTATGCGGTCCCAACGTCCTCGATAACTTCTTCTGTGA
TGTCCATCAGCTAGTCAAGTTGGCCTGTGCCAACACTTACATAGTGGAGCTTTTGATGT
TCCTCAACAATGGAGTTGTTATAGTCATGTGCTTTACACTTCTCCTAATCTCCTACACTG
TCCTGTTGCTGAAGGTCTGCAAACAGTCTTCCAAGGCAAAGAACAAAGTAGCCTTCAC
CTGTGTTTCCCACATCATTGTGGTTTTTGTCATGTGTGGCCCAGCTATGTGTATCTATGG
TTTACCCTTCCAAGCTGTCCCAATGGAAAAAGTTGTTGCTGTTTTCCATACTGTCATCTT
CCCCTTGACTAATCCCATGATCTATACCTTGTGTAACAAGGAGATCAAAGGCTCGATGG
GAAAGCTGGTCAGCAAATACATACTTTGGTGTGGAAAATTAAAAAAAATTTTTTGA
>Pelican_OR5V1 (Pelican31)
ATGGCAGGTGAGAACCAGACTCATGTGACGGAGTTCATGCTCCTGGGCTTTTCCCATG
GCCAACCCTTCCTCTTTGTTCTTTTCCTGGCCATTTACCTGGCCACACTGCTGGGAAAC
TCTGCAATACTCACCCTCGTGTCTCTGGATCCCCATCTCCACAGCCCCATGTACTTCTTC
CTCAGTCACCTGTCCTGCTTGGATATTTGCTACTCATCAGTGACGGTGCCCAAGATCCT
GGCAAATACCCTGCGCTCACAAGCAACCATCTCCTACCGTGGGTGTCTGGCACAAATG
TTCTTCCTGATGGGGTGTGCGGGGGCTGAGTGCGCACTCCTGGCTGTCATGGCCTACG
ACCGCTATGCAGCCATATGCCAGCCCCTGCGCTACACCCATGCCATGAGCCGGGGTGTT
TGTGTGGTGGCAGCCGCCAGCTGCTGGCTCTGGGGGATGCTGGACTCAGCCGTGCAC
ACCCTCCTGGCCTCCAGGCTCTCCTTCTGCGGGGCTGCCCGGCTCCAGCACATCTTCTG
TGACGTCCCTCCACTGCTGAGGGCTGCGTGCAGCAACACCCGCCCCAGCGAAGTGGC
ACTCCATGCTGCCAGTGTCTTTGTGGGCCTCAGCCCCTTCCTGCTTGTCATCGTCTCCT
ACCTCCACATCCTGGCCACTGTTCTCAGGATGCCTGTGGCCACCAGCCGGCGCAAGGC
CTTCTCCACATGCTCTGCCCACCTGCTCGTGGTCGCCCTGTACTTTGTGACAGCCAATC
TGAACTACAACTGGTCCAGCTCCAGCTACTCCCCAGCCACCGACACACTGGTCTCCGC
ACTGTACTGCATCGTCACCCCCATGCTGAACCCCCTCATCTACAGCCTCCGCAACCAGG
AAGTGCAGGGGGCCCTGCGGAAGGCTGTGTGGGGATGGGGCACGCTGGGCTGCCCAG
GCAGCAATGCATGA
>Pelican_OR51G2 (Pelican32)
ATGAGCAACAGTAGCTTCCTTAGACTTTCTACCTTCCCTCTAACAGGAATCCCAGGACT
GGAAAGTGGGAACTTGTGGCTTGCCATTCCTTTCTGCTGCATGTACGTTATTTCCATCCT
AGGAAATAGTGCAATCCTCTTTGTCATCGAAGCAGAGCGCAGCCTCCATGAGTCCATGT
ACCTCTTCCTGCGCATGCTGGCTATTGCAGAGCTTGGTGTGTCTCTGTCTACACTCCCT
ACAGTGCTAAGTGTGCTGCTTTTTGATTCATGGGAGATCAGGTTTGTTGCCTGCTTCAC
CCAGATGTTCTTCATTCACTGCTTCTCCATCCTGGACTCTGGGGTGCTGTGGGCCATGG
CCTTTGAGTGCTTCATGGCCATCTACAATCCCTTGCAGTATTCATCCATCCTGACCAACC
CCAGGATAGATGTCATCGGGCTGGGGCTCACAGTGAGGACCATTAGTGTCTTGCTCCC
CTTGCCGATACTTCTGAAGAAGCTGTCCTTCTGCAGGTCCCACATGCTGGCTCACTCCT
GTTTGCATCCCAACTTACTCCAGCTGCCCTGTGCAGATATCAAGGTGAATAGCATGTAT
GGCTTATTTGTCATCCTGGCCACCTTTGGGCTAGACTTGCTGTCTATCATCCTGTCCTAT
GTCATGATCATTAAGACTGTGCTGAGTATCAGCAAGGAAGAGTATCTCAAGGCCCTGA
ACACCTGCATCTCTCATATCTGTGCTGTTTTGATTCATTATATCCCCATGATAGGCTTGTC
CATGGTGTACAGGTTTGGGAAACCTGCCTCTCCTCTGATTCATGTCCTCATGGCCAATAT

CTACCTCCTTGCACACCCTGTGCTAAACCCCATTATTCACAGTATAAAAACTAAACAGA
TCTGCAAAGGGATACACAAACTTCTCACTCCAAGAAGGCGCTGA
>Pelican_OR8U9 (Pelican33)
ATGACACCTGTCAATCGCACTGATGTGCATGAGTTCATTCTCTTGGGGCTGGCCACCCA
CCCGGATCTCCAGCTCCCCCTTTTCTTGGCTTTCCTGGCCATGTATATGGTGACCCTCTT
GGGGAACCTTGGGATAATTATATTAATCAGGACCGATCTTCACCTTCACACCCCCATGTA
CTATTTCCTCAGCCACTTGGCTTTTGTCGATGTCTGCTATTCCTCCGTCATCCTCCCCAA
AATGCTGGTGCAGATCTTGCTGGAGGAGAAAACCATCGGCTTCTTGGGCTGCGCAGCC
CAGCTCTGCTGCTTCGTTGTTTTTGGGGTCACCGAGTGCCTCTTGCTCGCTGTGATGGC
CTACGACAGGTATGTGGCCATCTGCAAGCCCCTCCTGTACCCTGCCATCATGGGCGGAT
GGACGTGCTGGTGGCTTGTCACTGGTTCCTACGCTGCCGGCGTCTTGCACGCGGTGAC
ACACACCACTTTCATCTTTACTTTCTCCTTCTGCCGCTCCAATGTTATCAACCACTACTT
CTGCGACGTTGCCCCACTCTTAGCTCTCTCCTGCTCTGACACCCACACCTACGAGGTG
GTTGTCCTTGCTCTTGTCAGCATAAACTGTCTCAGCACCATGACCATCATCTTTGTCTCC
TACACTTATACCCTCCCGGCTGTCCTGAGGATCCGCTCTCCGGAGGGCAGGAGAAAAG
CCTTCTCCACCTGCGGCTCCCACCTGACGGTTGTCACCATGTTTTACGGGGCGATCTTG
TTCGTGTACCTGCGCCCCAGCTCCGCCTATGCATTGGATGAGAACAAGGTGGCCACGCT
ATTTTACACCATCATGACCCCCACGCTGAACCCCTTGGTCTACAGCCTGAGGAACAGT
GAGGTGAAGGCTGCCTTGAGAAGAGCGATTGGGAGACGGCAGTGA
>Pelican_OR51G2 (Pelican34)
ATGGAGCATGACTCGCATACCACGTGGGAATTCAATGGCTCCTTCTATCAGCCTTCAGC
TTTCCTCATGATGGGCATCCCAGGCCTGGAAGCCCTTCATCACTGGATCTCCATCCCTTT
CTGTGCACTGTACCTTATTGCTCTCTTGGGAAACTGCATGATCCTATTCATCATAAAGAA
GACCCAAAGTCTTCACGAACCAATGTACTACTTCCTCTCCATGCTGGCAGTCACTGACC
TGGGCTTGGTTCTATGTACACTGCCTACAACTCTGGGCATTTTTTGGTTTAATATGCGAA
GGATTGGGTTTGATGCTTGCCTCACTCAGATGTATTTCATCCACATACTGTCCTTCATTG
AATCCTCTGTGCTCCTGGCGATGGCATTTGACCGCTTCATTGCCATCTCCCATCCACTGA
GACATCCATCCATACTGACCAAGACAACTGTCATAAAAATAGGTCTGGCAATTATATTG
AGAGGTGTGGTCTCCCTCCTTCCCATACCTTTCTTGCTCAAGAGACTAACCTATTGTGG
GAAGACTGAGCTTTCTCATTCTTTTTGCTTCCATCCTGATATCATGAACCTAGCATGTGC
AGATATAAAAGTCAATGTCTTCTATGGCATGATTATTCTCTTATCAACAGTGGGGATGGA
CTTCATCTTCATAGTGCTTTCCTACATCCTGATCATTAAAACTGTTATCAGCCTTGCAAC
CAAGGAGGAGTGTCTCAAAGCTTTGAATACATGCGTCTCCCACATCTGTGCTGTTCTAG
TGTTCTTCATCCCAATGATCGGACTGTCCATGATCCATCGCTTTGGAAAGAATGTTCCTC
CTCTGATTAACACTTTGGTGGCCTACACTTACCTTATAATTCCCCCTGCTCTCAACCCCA
TTATCTACAGCATAAAATCCAGCCACATCCGTGAGGCTTTGCTCAGGGCACTGCGAAG
GAAGAGTGAATCTGACTGGTAG
>Pelican_OR52B2 (Pelican35)
ATGTATGAGCTCAACGAAAGCAGCTTTGATCCTATCACCTTCGTCCTGACAGGCATCCC
AGGCATGGAAGAGTCCCACATCTGGATCTCTGTCCCCTTCTGCCTGATGTACATCACTG
CGGTGTTTAGCAACTCTGTCCTCCTCTTTGTCATCATCATGGAAAGGAGCCTCCATGAG
CCCATGTACCTCTTCCTTGCTATGCTGGCGGTTGCTGACCTCATGCTTTCGACCACGAC
GGTGCCCAAAATGCTGGCGATCTTCTGGTTCAGTGCCAGGGAAATTTCCTTTGACGCC
TGCATTACACAGATGTTCTTTACCCATTTCAGTTTCATTGTGGAATCGTCCGTTCTGCTG

GCGATGGCGTTTGATCGGTACGTGGCTGTCTGCGACCCGCTGCGGTACTCTTCAATCTT
AACCCCCTCGGTGATCGGGAAAATAGCTGTGACTGCCGTTGTCCGGGGCTTCTGCATC
ATGTTCCCACCCATCTTCCTCCTGAAGCGGCTGCCGTACTGTGGACACAACGTCATGCC
CCACACCTACTGTGAGCACATGGGCATCGCCCGCCTGGCCTGTGCCGACATAAAAGCC
AACGTCTGGTATGGGCTGACAACGGCTCTTCTCTCCTCCGGCCTGGACGTCGTGCTCAT
CGCTGTCTCTTACGCTCTGATCCTCAGGGCGGTCTTTCGGCTCCCGTCCCCGGAGGCCC
GTCTCAAAACCCTGAGCACCTGCGGCTCCCACCTCTGCGTGATCCTCATGTTCTACGTG
CCCGCCTTTTTCTCCTTTCTCACGCACCGGTTTGGCCACCACATCCCAAGTCGCGTTCA
CATCCTCTTGGCCAACCTCTACGTCGTTGTCCCACCGATGCTCAACCCCATTGTGTACG
GGGTGAGGACAAGGCAGATCCGGGAGCGCGTCATCCGCCTCTTCTGCCCCGCGGGGG
AGTGCCCCTGCCCCGGCTGGGGGAGCAGGTGCTGA
>Pelican_OR10A7 (Pelican36)
ATGAACCATACAGAAAAATCTGGTAGAAGCAACTGCACCACAGTGACCATGTTCATTC
TCCTGGGCCTCTTCAGACATACTGAGCTGCAAATCTTGTTTTTCTTCATGTTTGTCCTGA
TTTACACTATTACTATCATCGGGAACAGACTTGTCATCATTGTCACAGTTCGACCATCCT
TTTATACACCCATGTACTTTATTCTCAGGGTCCTCGCCTTCATGGATATTTGTACTACTTC
AGTCATTGTACCCAAGATGTTAGTGAATTTTCTCTCACGGGGCAAGTGCATTTCCTACAT
AGGCTGTGCTGCCCAGCTGTACTTCCTGATTTTTCTAGCAGCTGCTGAGTACTATCTTCT
TGTTGCCATGGCCTATGACCATTACCCGGCCATTTGCAACCCCCTGAGATACAGAATTTT
GGTGAACAGAAGGGTTTGTTTTTCCCTAGTCCTGCTGTCTTTCCTCACTGATAATGTTG
TGTCAGTGGTGCAAACTGCTTTGTGTTCACATTGCCATTATGTGGGCCTAACAAGATTA
ACTATTTCTTCTGTGATATCACAACACATTATATTTCTGTGCACTGAAACATCTCTGTTTG
AAATTCAAGCCATGACAACCACAGTATTGGTCATTTTCACCCCATTTTCTCTCATCATTC
TGTCCTACACCATCATCTTCTCCAGGATTTTGACCATGCCCTCTGCAAGTGGAAGATAC
AAGACATTCTGGACCTGTTCTTCGCATCTCCTAGTGGTGATGCTTCAGTATGGGAGTGG
CAGCCTGATTTACCTAAGACCCAAGTTCAGCTATCCACAAGATGCTAAAAAAGTGCTG
GCTTTAGTGTACCCAACCATAACTCCTTTGTTAAATCCCATTATCTACAGCTTGAGAAAT
AAGGATGTGAAAAGGATTTTAAGAACAATAATAAGGAAGGTGAGGAAAAGGTAA
>Cormorant_OR14J1 (Cormorant1)
ATGCCCTCTGGGACACCAGGACTATTTCCTACTATGGATGTGCTGCCCAGGTCTCTATGT
TTGTCTTTTTTGTCACAGGAGAGTTTTATCTCCTCACTGTCATATCCTACGACCGCTACG
TGGCCATCTGCAAACCCCTGCACTACGGGATGCTCCTGGGCGGCAGAGCTTGTGCCCA
CATGGCAGCAGCTGCCTGGGCCAGTGGGTTTCTCAGTGCTGTGCTGCACACGGCCAAT
ACATTTTCACTCCCCCTCTGCCAAGGCAATGCTGTGGACCAGTTCTTCTGTGAACTTCC
CCAGAAAGCTCTCCTGTGCTGCCCAGGTCTCTATGTTTGTCTTTTTTGTCACAGGAGAG
TTTTATCTCCTCACTGTCATATCCTACGACCGCTACGTGGCCATCTGCAAACCCCTGCAC
TACGGGATGCTCCTGGGCGGCAGAGCTTGTGCCCACATGGCAGCAGCTGCCTGGGCCA
GTGGGTTTCTCAGTGCTGTGCTGCACACGGCCAATACATTTTCACTCCCCCTCTGCCAA
GGCAATGCTGTGGACCAGTTCTTCTGTGAACTTCCCCAGATCCTCAAGCTCTCCTGCTC
AGACACGTACCTCAGGGAAGTCGGGGTTATTGCCTTTTGTTTTTGTTCAGGTATTGGGT
GTTTTGTTTTCATTGTTTTCTCCTACGTGCAGATCTTCAGGGCCGTGCTGAAGATCCCCT
CTGAGCACGGACGGCACAAAGCCTTTTCCACGTGCCTCCCTCACCTGGCCGTGGTCTC
CCTGTTTATCAGCACTGCCATGGTTGCCTACCTGAAGCCTCCCTCCATCTCCTCCCGATC
CCTGAATCTGGTGGTGGCAGTTCTGTACTCAGTGGTGCCTCCAGCAGTGAATCCCCTCA

TCTACAGCATGAAGAACCAGGAGCTCAAAGATTCCCTGAGGAAGCTGATTTTATGGAC
ATTTTTCTGTAGTCATAAAATTCCCATCACTCTCCACAAGTGA
>Cormorant_OR2AT4 (Cormorant2)
ATGGAAAGCTGCAGCAGCAATGCTTCGGCTAAAGTATTTTTCTTGGTGGGGTTTCCAGC
TCTCCAGGATTTCCAGACCCTGCTCTTCGTTGTGTTCTTGCTATTCTACCTGCTGATTCT
TGTTGGTAACGCCGTTATCATCACCGTGGTTGTGGTTGACCGTACACTCCACAAACCCA
TGTACTTTTTCCTGATTAACCTCTCTGTGTTAGACCTGCTGTTCACAACCACCACCATCC
CCAAAATGCTGGCAATGTTCCTGGCCAACGCTAAAACCATCTCGTTTCGGGGCTGTTTT
CTGCAGATGTACAGTTTTCACGGGTTGACGGTAACCGAGGCGCTTCTCCTGGTGGTCA
TGGCTTACGACCGCCACGAAGCCATCTGCAACCCCCTCCGTTACCCGGCCAAGATGAC
AAGAAGGGTGACCATCCAGCTGGCAGCAAGCGCCTGGATCACTGCGCTGCTGATACCC
GTGCCCGTCATCACGCAGACCTCTCAGCTAGCTTACAGGGACACGACCAGGGTCCACC
ACTGCTTTTGCGACCACCTCGCGGTGGTACAAGCCGCGTGCTCGGACTTCAGTGCCGA
TTTCCAGACCTTCTTGGGGTTCTCCATCGCTATGACAGTGTCGGTCGTCCCTCTGTTGC
TCGTCACCCTCTCGTACGTCCACATCATCCTCTCCGTATTAAAGATCAACTCCAAAGAA
GGACGCGTGAAGGCTTTTTCAACATGTACTGCCCATCTGCTTGTGGTGGGCACTTACTA
CTCCTCCATCGTTGTGGCGTACATGTCCTACAGAGCGGACATCCCGGTTGATGTCCATG
TCATGAGCAACGTTGTCTTCTCTATTTTAACTCCCTTGTTAAACCCCATCATTTACACTTT
ACGGAACAAGGAAGTAAAATCCGCGGTTAAAAAGTCTATTTTTCAGAAAGTCTTTCTC
CTTTCTAAAAAAATTAATTTATTTGGGTAA
>Cormorant_OR5AU1 (Cormorant3)
ATGGTTGAAGATAATTATACGTTTGCATCAGAGTTTATTCTCCTGGGCTTCACAACCCGA
GAAGATCTGCAGGTAACATTCTTTGTCTTATTCCTTGCCATCTATGTCGTCACTCTAATA
GGAAATCTGGGAGTAATTATATTAATCAGAATCAATTCATGCCTACACACCCCCATGTAC
TTCTTCCTAAGCCACTTGTCGCTCCTGGATATCTGCTACTCCTCCACCATCATCCCTCAA
ACCTTGTTGAATTTTTTAGTGGAGAAGAAGGTTATTTCCTTCGTTAGGTGTGCCACTCA
GCTCTTCTCCTTTGCGACTTGTGCCACCGCCGAGTGCTACGTGCTGGCTGCCATGGCTT
ATGATCGCTACGTGGCCGTTTGTAACCCCCTGCTCTACTCTGTGGTCATGTCCCGGAGG
TTTTGCGTTGGGATGTTGGCTGGTGCCTACTTAGCTGGTGTGATCAGCTCCACCATACA
CACAGTTTCTATATTTCTTCTCCCGTTCTGTCGGTCCAAGAGGATCAATCATTTCTTCTG
CGATGGGCCACCGCTGCTAGCCCTCGCCTGCTCTGACACCCATGTCAACGAGGTGATG
GTTTCTGCCGTGGTGGGGTTCAACGTGCTAAGCACCGCGGTCTTCATTTTAGTCTCCTA
CTCGTCGGTCCTCGCCACCGCCTTGCAGATGCGCTCTGCAGCCGGTTGGCACAAAGCC
TTCTCCACTTGTGCCTCGCACTTGGCCTCCATCGCTTTGTACTACGGCAGCTCCCTCTTC
ATGTACCTGCGCCCCGGCTCCAGACACTCCTTGGAGCACGGCGAGGTGGCCTCCGTGC
TCTACTCCGTTGCAGTCCCCATGCTGAACCCGCTCATCTACAGCCTAAGAAACACGGA
CATGAAGAACGCCATGAGGAGAGCAAAAGGTAGAGTCCTCTCCTCCTTGTCTACCCAC
GGTTCCTGGTCAGCTGGAAGGAGAGGACCACCCTTCCGTGGTGAGGAGGGTTAG
>Cormorant_OR6Y1 (Cormorant4)
ATGGGTGGGAGGAATGAAACCAATGTCATGTATTTCATTTTCCTGGGTTTTCCCACCAC
TGCTGAACTGCAACTGCTCCTCTTCTCTGCTTTACTTCTGGCTTATTTATTAACTGTGTT
GGAAAACTTCCTTATCATTCTCATCATCCGAAATAACCACGCTCTGCAAAAACCCATGT
ATTTCTTCCTAGGAAATCTGTCTGTCTTAGAGATCTGGTATGTTTCTGTTATTGAGCCAA
AGATGCTCATAGATTTCCTCTCTCAAGACAAGCATATCTCATTCCAGGGGTGCATGACA

CAGTTGTATTTCTTTGTGACTTTTATTTGTACTGAGTACATTCTGTTAGCAGTTATGGCCT
ATGACCGTTTCTTGGCCATATGCAAACCTCTCCAGTATTCACTCATCATGAATCATCAGT
TCTGTGCTCAACTGACAGCTGGCTGTTGGATGTGTGGTTTGATCACTTCTTCAATCAAG
CTGACCTTTATAGCCCAGCTCTCATTCTGTGATGTAGACAAAATCAATCACTATTTCTGT
GATATATCACCCCTACTGAATATCTCCTGCAGCGATTCCTCTTCAGCTGAGCTAGTGGAC
TTCATCTTGGCTCTGATTGTCATCATGGTGCCTCTGTGTACTGTGGTCACCTCTTATATTT
GCATCATATTCACCATGCTGAAGATCCCTTCTTCTCAGGGGAGGCAAAAGGCCTTTTCC
ACCTGCAGCTCCCACTTGACTGTAGTGATATTGTTCTACGGCACCACTCTTTTCACTTAT
GCCCACCCTAAGGTCATGTATACCTACAGTGCTAACAAGTTGGTATCAGTCTTGTACAC
GGTAGTTGTGCCACTTCTGAATCCTCTTATATATTGTCTTAGAAACAAAGAAGTCAGGT
TTGCCCTGAGGAAGACCTTTACTTGCACAAGACACACCTAA
>Cormorant_OR4D5 (Cormorant5)
ATGGCACTGGGAAACTTCTCCCAGGTGACTGAATTCATCCTACTGGGGCTTTCCGATAC
AAGGGAGCTGCAAGTCCTCTTCTTCACCTTCTTCTTCCCAGCCTATGCCATGGTTCTGC
TGGGGAACCTTCTCATCATTGTAGCAGTCTGGACTGACCCCAAGCTGTCCTCACCCATG
TACCTTCTCCTCTGCAATTTGTCCTTCATAGATATCTGCTGCACCTCTGTCACCTCTCCC
AGGATGCTGGTGGACCTGCTCTCCCAGAGGAAGACCATTGCGTTTGAAGACTGCATAG
CCCAGCTGTTTTTTCTGCACTTTACTGGGGCATCAGAGATGTTCCTCCTGACTGTGATG
GCATACGATCGCTACACTGCCATCTGCAAGCCCCTGCACTACACAGCCATCATGAGCCG
GCAGGTGTGCTGGGTCCTGGTGTCTGCCTGCTGGGCAGGGGGCTTCCTCCACTCCATT
GTCCAGACGCTGCTCACAGTCCAGCTGCCCTTCTGTGGCCCTAACACAATCGACAACT
ACTTCTGTGACATGCCTCTTGTCATTCGCCTTGCCTGCACAGACATCTATGTCACTGAG
TGGCTTATGGCTTCCAACAGCGGCTTAATATCCCTGGTTTGCTTCCTGGTGCTGGTCACA
TCCTACACCTACATCCTGGTCACAATCAGGGTCCGCTTCACCGAGGGGCGCTGGAAGG
CACTCTCTACCTGTGCCTCACATGTGATGGTCGTCACCTTCTTCTTCCTACCCTGCATCT
TCATCTACTTCCGGCCCTTTTCTACCTTCTCCTTTGACAAGCACATCTGTGTGATCTCCA
CTGTCTTGTCCCCAGTGATGAACCCCCTCATCTATACCCTGAGGAATAACGAGGTGAAG
GCATCTATGTGGAAATTGTGGCAGCGCTGCAGAGCCTTCTGA
>Cormorant_OR10A7 (Cormorant6)
ATGAGCCCCAAATCCCTGCAGTGGGGGAACCTCAGCAGCCCCACCGTGTTCCTTCTTT
TGGGATTTTCAAGTGCCTACAGAGCACAGGTGACGCTCTGCCTATGCTTCTTGCTCATT
TACCTGGTGACGGTGCTGGGGAACCTGCTCATCATGACCCTTATCTGGCTGGATGCTCA
CCTGCACTCTCCCATGTATTTCTTCCTGGGCCACCTCGCCTTCCTGGACATCTGCTACTC
CTCTGTCTCTCTCCCTAAGATCCTTCGAGACTCCTTCTCACCGCAGAAGACCATCTCCT
TTGTGGGCTGCATCACACAGATCTACTTTTTCCTTTGCTTTGGGGGCTCCGAGTGCATA
CTCCTGGCTGCCATGGCCTATGATCGGTACCTGGCCATCTGCCACCCCCTCCACTACCC
GGCATTCATGAGCAAGAAGATGTGCCACTGCTTAGTGGCCATTGCGTGGCTGAGCGGC
TCCTTCTCATCCCTGGTCCAGGCCTTCCTCACAGCCCGCTTGCCCTTCTGTGGGTCCAA
CATGATTGACCACTTGTTCTGCGAGATGCCCTTCTTGCTGAAGGCATCCTGCAGCCCTA
ATACTCCCCTCAACAAGGCCACCTTGTATGCTTTGGCTGGGACTGTTGCAATGGGCTCT
TTCCTCCTTACTCTCTTGTCGTACGTTCATATCATTGGGGCTGTCCTCCAGAAGGGAGC
AGGAACACAGAGGGCCTTTGCTACCTGCACCTCCCACCTGACCGTGGTGTCCCTGTTC
TTCGGCACTGGGGCCATTGCATACCTGGTGCCTCACTCCAGCAGCTCCAAGGAGATGG
ATGAGGTCCTCACCCTGCTGTATGCCGTTGTGACCCCCATGCTCAACCCCATCATCTAC

AGCTTGAGAAACAGTGAGGTCAAAGGAGCCATCAGGAAAGCCCTGCACAGGGGGGC
GTTACAGATGTCCACCAAGGGTGCAGGCATCACTGCTGAGCAACCCCGACCCTCCCCA
CAGCAGGGATACGACTCTCAGTGA
>Cormorant_OR5AR1 (Cormorant7)
ATGGCAGAAAGCAACTGCACGCGAGTGACTGAGTTCAGATTAACAGGGTTCTCAGAG
GACCCAGGGATTCAGGTCACCCTCTTTCTGGTACTTCTCATCACCTACCTTGTTACCATC
CTGGGGAACCTTGGGATGATTGTGTTAATCAGGGCCAGCCCTCAGCTCCACTCCCCCAT
GTATTATTTCTTGGGTAACCTGGCTTTTGTAGACCTCTGTTCTTCCACCGTCATCGCCCC
CAAGATGTTGGTTGACTTTATATCAGAGAAGAAGGGCATCGCTTATGCTGGGTGCGTGG
CTCAGGTGTTCACTTTTGATCTTTTTGGGATGACCGAATGCTTCCTGCTGGCTATGATGG
CGTACGACCGTTACGTGGCCGTTTGCCATCCCCTGGTGTATCACCTTGTCATGCCCCCA
AAATGCTGTTCCCAGCTGGTGACTGGGTCATATCTTGTGGGGCTGACAAACGGCATGG
GACAGACGTTCTGCATGGCCAGTTTATCCTTCTGCGGCTCCAGCATCATCAACCTGTTC
TTCTGTGACATTTCCCCTCTGATATCACTCTCAACCTCCGACACCACCCTCAGCCGCAT
CATCCTAAGGACTTCAGCATCTTTATTCGGTGTGTCCAGCAGCCTGGTTGTGCTGGTTT
CCTACGTGGCCATCATCTCTGCCATCCTGAGCATCAGTTCAACCAAGGGCAAGCACAA
AGCCTTCTCCACCTGCGCCTCCCACCTCACCGCTGTGAGCATCTTCTATGGGACATCGC
TTTTTATGTACTTAAAGCCCAGCTCAGACAGCTCAAGAGAAGATAAATGGGCTGCGGT
GCTCTACACTGTGGTGACTCCCATGCTGAACCCTTTGATCTACAGCCTGAGGAATAAGG
AGGTGAAGGAGGCTTTGAGGAGACTCACAAAAATAAAATGA
>Cormorant_OR5V1 (Cormorant8)
ATGGCAGGTGAGAATCAGACTCATGTGATGGAGTTCATGCTCCTGGGCTTTTCCCACA
GCCAGCCCTTCCTCTTTGTTCTCTTCCTGGCCATTTACCTGGCCACACTGCTGGGAAAC
TCTGCAATACTTGCCCTCGTGTCCCTGGATCCCTGTCTCCGCAGCCCCATGTACTTCTTC
CTCAGTCACCTGTCCTGCTTGGACATTTGCTACTCATCAGTGACGGTGCCAAAGATCCT
GGCAAATGCCCTGCGCCCACAGGCGACCATCTCCTACCGTGGGTGCCTGGCACAGATG
TTCTTCTTGATGGTGTGTGCAGGGGCTGAGTGCGCACTCCTGGCTGTCATGGCCTACGA
TCGCTATGCAGCCATATGCCAGCCCCTGCGCTACACCCATGCCATGAGCCGGGGTGTCT
GTGTGGTGGCAGCTGCCAGCTGCTGGCTCTGGGGGATGCTGGACTCAGCCGTGCACA
CCCTCCTGGCCTCCAAGCTCTCCTTCTGCAGTGCTGTCCAGCTCCAGCACATCTTCTGT
GATGTCCCCCCACTGCTGAGGGCTGCTTGCAGCAACACCCGCCCCAGTGAAGTGGCA
CTCCACACTGCCAGTGTCTTTGTGGGCCTGATCCCCTTCCTGCTTGTCATCATCTCCTAC
CTCTGCATCCTGGCCACTGTCCTCAGGATGCCCATGGCCACTGGCCGGCGCAAGGCCT
TCTCCACATGCTCTGCTCACTTGCTTGTGGTGACCCTGTACTTTGTGGCGGCCAACTTG
AACTACAACCGGCCCAGCTCTGGCTACTCCCCAGCAGCTGACACACTGGTCTCCACAC
TGTACTGCATCGTCACCCCCATGCTGAACCCCGTCATCTACAGCCTCCGCAACCAGGA
GATGCGGGAGGCTCTGCGGAAGGCTGTGTGGGGACGGGGCAAGCTGGACTCCCCAGG
CAGCAATGCATGA
>Cormorant_OR5J2 (Cormorant9)
ATGGCTAAAGGCAATCACACCACGGTGACCCAGTTCCTCCTCCTGGGACTGACGAGCG
AGCCTAAGCTGCAGGCTCCTCTCTTCGTAATCTTCTTAATGATTTATCTCATCACCCTGAT
AGGCAATCTTGGGCTGATCACGCTGATCAGGACAAACCGCCGCCTGCACGCTCCCATG
TATTTCTTCCTCTGCAACCTCTCTGTTGTTGATCTTTGCTACTCCTCCGTCTTTTCTCCAA
AGCTGCTTATTGGCTTCTTGGCAGAAAAGAAAACGATTTCTTACCCCGCCTGCTTCGCC

CAGCATTTCTTTTTCCTTGCGTTTGTGACCACGGAGGTGCTCTTGCTGGCTGCCATGGC
GTACGACCGCTACGTAGCCATTTGCCACCCGCTGCACTATGCTGTTTCTATGCCCGGGA
GGGTCTGCGTTCAGCTGGTGGCCGGGTCATACGTAGGGGGGATTTTGAACTCGCTAAT
CCAAACGTGTTTCTTGTTGCCGTTGCCTTTTTGTGGGCCCAATGTCATCAACCATTACTT
CTGTGACACCAACCCTCTGCTCAAACTCACCTGCTCTGATGACCACCTGAATGAGCTG
TTGCTTGTAACCTTCAACGGGACCATTTCCATGTCTGTGCTCTTCATCATCATCGTCTCC
TATGCCTACATCCTCTTCTCCATCCTGAGGATTAGATCTGCCAAAGGAAGGCACAAAGC
CTTCTCCACCTGCGCCTCCCACCTCCTGACCGTTACCTTGTTCTACGTGCCCGCGGGGC
TGAGCCACATGCAACCGGCCTCCAAGTACTCACTGGAGATGGAGAAAGTCACCGCCG
TGTTTTACACCCTGATCGTCCCTATGCTCAACCCTCTGATCTACAGCTTGAGGAACAAG
GAGGTCAAGGATGCACTTAGGAAAGCGACAGCAAATAACACTTTTGGGAGTTGCCTG
CTCCCCAAGCCGACCCCAGTCAGTTGA
>Cormorant_OR51G2 (Cormorant10)
ATGGAGCATGACTCACATACCACGTGGGAATACAATGGCTCCTTCTATCAGCCTTCAGC
TTTCCTCATGATGGGCATCCCAGGCCTGGAAGCCCTTCACTACTGGATCTCCATCCCTTT
CTGTGCGCTGTACCTTATTGCTCTCTTGGGAAACTGCATGATCCTCTTCATCATAAAGAA
GACCCAAAGTCTTCATGAGCCAATGTACTACTTCCTCTCCATGCTGGCAGTCACTGACC
TGGGCTTGGTTCTGTGTACGCTGCCTACTACCCTGGGCATTTTTTGGTTTAATATGCGAA
GGATTGGGTTTGATGCTTGCCTCACTCAGATGTATTTCATCCACATACTGTCCTTCATTG
AATCCTCTGTGCTCCTGGCAATGGCGTTTGACCGCTTCATTGCCATCTCCCATCCACTG
AGACACCCATCCATACTGACCAAGACGACTGTCATAAAAATAGCTCTGGCAATTATATT
GAGAGGTATGGTCTCCCTCCTTCCCATACCGTTCTTGCTCAAGAGACTAACCTATTGTG
GGAAGACTGAGCTTTCTCATTCTTTTTGCTTCCATCCTGATATCATGAACCTAGCATGTA
CAGATATAAAAGTCAATGTCTTCTACGGTATGATTATTCTCTTATCAACAGTGGGGATGG
ACTTCATCTTCATTGTGCTGTCCTACATTCTGATCATTAAAACTGTTATCGGCCTTGCAA
CCAAGGAGGAGTGTCTCAAGGCTTTGAATACTTGTGTCTCCCACATCTGTGCTGTTCTA
GTGTTCTTCATCCCAATGATCGGACTATCCATGATCCATCGTTTTGGAAAGAATGTTCCT
CCCCTGGTTAACACTTTGGTGGCCTACACCTACCTTATAATCCCCCCTGCTCTCAACCCC
ATTATCTACAGCATAAAATCCAGCCACATCCGTGAAGCTTTGCTCAGGACACTGCGGAG
GAAGAGTGGATCTGACTGGTAG
>Cormorant_OR4S2 (Cormorant11)
ATGGAGAATGCAAGCAGCGTGAAGGAATTTGTTCTTCTGGGCCTCTCAGAGAACCAAG
GGGTGCAGAAAATATTTTTTGTGATGTGTCTGTTCTTCTATATGATTATTGTAGCAGGAA
ATCTGCTCATTGTTATCACTGTAATTAGCAGTCAACGCCTGAACTCTCCCATGTATTTCT
TCCTCTGCTACTTGTCCTTTGTAGATATCTGTTACTCTTCTGTCACAGCTCCCAAAATGA
TTGCCGACTCCCTTGTTGAAAATAAAACCATCTCCTTTGTGGGTTGCATAGCACAGCTG
TTTGGGGTACATTTCTTTGGCTGCACAGAGATCTTCATCCTCACAGTGATGGCCTACGA
TCGCTATATTGCCATCTGCAGACCTCTCCACTACACCACCCTCATGACCAGGCGGGTGT
GTGGCCAGATGGTGATCGGCTCGTGGGTAGGAGGCTTTGTACACTCCATAGTGCAGAC
TCTTCTAACCACTCAGCTCCCCTTCTGTGGCCCTAACAAAATTGACCACTACTTCTGTG
ATGTCCACCCCCTACTACAACTGGCCTGTACCGACACCTACGCTGTAGGTATCATTGTT
GTTGCCAACAGCGGAATGATAACGTTGAGCTGTTTCTTTATCCTGGTCATGTCCTACGT
TGTCATCCTGGTTTCCTTGAAAAGTCAAACATCCAAAGGGTGGCACAAGGCCCTCTCC
ACCTGTGGGTCCCACATCACTGTGGTGATTCTGTTCTTCGGGCCGTGCACGTTCGTCTA

CATACGTCCATCCAGCAATCTGTCGGAGGACAAGAGCGTGGCGCTGTTTTACGCTGTC
ATCACGCCCATGCTGAACCCACTCATCTACACGCTAAGAAATGAGGAGGTGAAGAGTG
CCATGAGAAATCTGTGGAGTAGAAAGGTGGGAAGTGAAAATGGAAAGGTGTATAACT
GTGGTAACATTTCCAGTGGATCAGAGAAAATCAAGCATTTTTCACTATAA
>Cormorant_OR6B1 (Cormorant12)
ATGAAGAACCAAACTGATATCAATGAGTTCATTCTTCTGGGATTTTCATATGGGCTGCA
GGTCCAGACCTTACTTTACCTGGTCTTTCTGGTCACCTACATGGTAACAATCACTGAGA
ATGCAATCATCATCTTTGTGGTAAAAAGGAACCATCTACTCCAAAAGCCCATGTATTATT
TCCTGGGGAACCTGTCCTTCCTGGAGATTTGGTATGTTACAGTAACATTGCCTAGGCTT
TTATTTGGGTTCTGGTCACAGAGCATGACCATCTCATTCTCCAGCTGCATGACACAGTT
ATACTTCTTTATCTCCCTTATGTGCACTGAATGTGTCCTCCTGGCTGTAATGGCCTATGAC
CGCTATTTGGCTGTCTGCCATCCCCTGCGCTACCCAGCCATCATGACCCACAAGTTGTG
CTTTCAGCTGTCGATTCTCTCATGGGCAGGAGGCTTTTCCATTTCCTTGGTCAAGGTGT
CTTTTATTTCACGCCTCACGTTTTGTGGTCCACAAGTCATAAACCACTTCTTTTGTGACA
TCTCTCCAGTCCTGAACCTTTCCTGCACTGACATGTCCCTAGCAGAGACAGTGGACTTT
GCATTAGCCTTGGTGATCCTGCTGGTACCTCTCTTGATCATTGTTTTCTCCTACTGCTGTA
TCTTGTCAACTATCTTGTGTATGCCTTCAGCCCAGGGAAGGAGGAAAGCTTTTTCCACT
TGTACCTCCCATTTCACTGTAGTCGTTATCTTTTTCTCAGCCACCCTCTTCATGTATGCCA
GACCCAGGAGGATCCATCCATTCAACCTCAACAAAATAGTGTCTGTCTTTTATGCTGTA
TTCACTCCAGCACTGAACCCTCTAATCTATTGTCTGAGGAACACAGAGGTGAAGGAGA
TTCTGAGAAAGATCATAGGCACAAGCTGCTTTGCACACCAATAA
>Cormorant_OR5AR1 (Cormorant13)
ATGACGGCCAAAGGAAATTGCACCCCCAGTGCCGAATTTCTCCTCTCGGGGTTCTCAG
AGCAGGGGGATGTCCAGGCTGTCCTCTTCATGGTCTTCCTGGTGATCTATGTGATCACT
CTGCTGGGGAATCTGGGGATGTTGGTGTTAATCAGGCTGGATGCCCAGCTTCACACCC
CCATGTACTTCTTCCTGAGCAACCTGGCCTTCCTAGACATCTGCTATTCCTCCTCAATCA
CCCCCAGACTGCTCTCGGACCTGCTAGCAGAAAGGAAGGTCATTTCTTACCCCGCGTG
CCTCGCACAATTTTATTTCTATGCGGTCTTTGCCACCACCGAGTGCTACCTCTTGGCTGT
GATGGCGTATGACCGCTACGTGGCTATCTGCAGCCCACTGCTCTATGCCATCTCCATGTC
CAGCAGAGTTTGTGCACTGCTGGTAGCTGGCTCGTGCCTCGCTGGGATCGTGAATGCC
ACTATCCACACGGGCTTTGCGCTTCGGCTGTCCTTCTGTGGCCCCGCCATCGTCAACCA
CTTTTACTGTGAGGGGCCCCCGCTTTACGCCATCTCGTGCACGGACCCCACCCTCAATG
AGATTATGATGTTCGTTGTGGTTGGCTTCAACCTGTTTGTCACCAACCTGACCATCCTCA
TCTCCTATACCTACATCCTGGCCACCATCCTGAGGACGCGCTCGGCCGCGGGCAAACG
CAAAGCCTTCTCCACGTGCGCGTCCCACCTCACCGCCGTGACCCTCTTCTATGGATCTG
CTGCGTCCATGTACTCCCGACCCAGCTCCAGGCACTCCCAGGACCTTGACAAAGTGGC
CTCTGTGTTTTACACTGTGATCACCCCCATGCTGAACCCCGTCATCTACAGCCTGAGGA
ACAAGGAGGTAAAGAACGCGCTGGGCAGGGTGATGGAGAGGAAATGTTCATCAGAAA
AGTAG
>Cormorant_OR51E1 (Cormorant14)
ATGTCATCTTCCAATAGGACTGGGTCCAGTGCCCTGGCCTTCGTCCTCACCGGGATTCC
TGGTCTGCCGATGAGCAGCTACTGGATGGCGTTGCCTCTCTGCTGCCTGTATCTCCTCA
TGCTCCTGGGGAACTGCACCTTAATCTGGATGATAAAGACAGAGCACAGCCTCCATAC
ACCAATGTACTATTTCCTCTCCATGCTGGCCGTGGCAGACCTGGGCTTGTCTCTCTCCA

CCCTGCCCACCATGCTGGCCGTTTTCTGGTTTGAGTCCACCTCCCTCTGTTTTGAGGCG
TGCGTCGTTCAGATGTACTTCATCCACTCCTTCTCTACCATTGAGTCTGGGGTCCTGGT
GGCCATGGCCTTTGACCGCTTTGTTGCTATTTGCTACCCTTTGCAATACGCCTCTGTCCT
GATGAGTTCCCTGATAATGAAGGTGGCGGGGGCGATCTTCATGCGGGGCATCTGTGTG
GTACTACCTGTTGCCATCCTTATTAAGAAGATGCCTTTCTGCGGGTCCCATGTCCTGTCT
CACTCCTTCTGTCTGCACCAGGACATGCTGAGGCTGGTTTGTGGGGACATCAGGGTCA
GCAGCTTGTATGGGCTCATCGCAGTGATACTCACCAAGGGCCTGGACTCCCTGACCATC
CTCCTGTCCTACGTGATGATCATCAGAGCCATCTTGAACATCGCCTCCCACGAAGCACG
AGCCAAAGCCTTTAGCACATGCATCTCCCACCTCTGTGCTGTCCTGATCTTCTACATCC
CGCTCATTGGCTTGTCCATCATCCACAGGTTTGGGAAGCACCTGCCCCCCCTCACTCAC
ATGGTCCTGGCTGACGCCTATCTCCTGGTGCCTCCTGTCCTGAACCCACTCGTGTACAG
CTGGAAAACCAAGCAGATCCGCAGGCGGATCCTCATCCTGCTCTGTCGGAGAGGGTCC
CAGCAGCAAGTTTAG
>Cormorant_OR6C4 (Cormorant15)
ATGAACCACACAACAGTAGCAGAATTTGTCCTCTTGGGGCTGACCAACAGCCGCCATT
TGGAGATCATCCTCTTTCTGTTCCTTGTGATTGCCTACTTCTTGATCCTGCTTGGAAACA
TTACTGTCATCAGCATTACTCTTATGAATCATTTCCTTCAGACCCCAATGTACTACTTCCT
CAGAAATTTTGCCCTTTTGGAAATCACTTTCACCTCCACCTTCATTCCCAGCACCCTCT
ACAGCCTTGTGACAGAGAGGAAGATGATTTCCCTGCCTGGCTGTTTCCTTCAGATGCT
GCTTTTCTTTTACTTGGGTACCTGCACATTTTTCCATGTGGCAACAATGTCCTTTGATCG
ATACGTTGCCATTTGCTGCCCTTTGCATTACACAACAGTTATGAACAACAGATTTTGCCT
CCAGCTGGTTCTGGCTTGCTGGGCAGTGAGTTTTCTCTTGATGTTTCCTCCCACCATTAT
GATTGTCCAGTTGCCATTCTGTGGTCCCAATGTCATGAACCACTTTTACTGTGATACTTC
CCTGTTGTTGCAGCTGTCCTGCACAGACACAGGGTTCATTGAAGGACTGATGCTTATTA
TACTAATTATCATCATACCTGGGACCTTAATAGTAACTGCTGTTTCTTATGGCTGCATTGT
TATCACCATCTTGCATATACCATCTTCCACAGGTAGGAAGAAGGCATTTTCCACTTGCTC
AGCTCACCTCATGGTGGTGATTATTTTTACAGCACATGTATTTACAGGTATATCCGCCC
AGCACAGCGAGGTGGGAAGGACTCTGACAAAGTTCTTTCTTTCTTCTTCTCAGTGGTG
ACTCAGATGCTTAACCCATACATCTACTCACTCAGGAACAATCAAGTCAAACAAGCCTT
AAAGGAGAGCATATTGAAGGCATTTTCTAGCTCCCTAAGGCAGTAG
>Cormorant_OR5AP2 (Cormorant16)
ATGGCTGGGGGAAACCACTTGAGCGTGACTGAGTTTGTCCTCCTGGGCTTCACCGACC
TGCAGGAGCTGCCCTTCGTGATTTCTATACTCATCTACATCGCCATGCTCGTGGGGAAC
CTGAGGATGACCATCCTCATCAGGACCAACTCTCAGCTTCACATGCCCATGCAATTTTT
CCTCAGCCACCTCTCTTTCCTGGACACCTGCTGTTCTTCATCCATCACGCCAAAGCTCC
TGTTGGGTCTCCTTGCAGAGAGAAATGTCATTTCTTTCAATGGCTGCATCACATACTTCT
TTGCAGTATTCGGCACTACGGAAGCCGTACTTCTGGCCGTCACGGTGTACGATCGCTAC
GTGGCCATCCGTGAGCCTCTGCACCTCTTGGCTGCCACGTCCCATGGGGTCTGTGTCCC
GCTGGTGGTGGGCTCCTGCGCTGCCAGGAGCCTGAACGCCCCTGTGCACACCAGTGC
TCTCCTCCAACTCTCCTTCTGGGGCCCAAAGCTTGTCAATCATTTCTACTGTGAAATCC
CACTGCTCCCGCTACTCTCGTGCTCGGACACCTGGTTCAATGAGATAGCGATGGCCGCA
TGTGGTGGCTTCATCATAACACCCTCAGTCTTGGCCATCGTTGTCTCCTGCGCCTGCAT
CCTGCTCACCAACCGGAACATTCGCTCTGCAGAAGGCAGGCACAACGCCTTCTCCACT
GGCATCTCCCACTTCGTGGCTGCTGCCCTCTCCTACAGCTTTGCAGCTTGCTTGTATTTC

CATCCCTTTTCCAGACGCGCAGAAGATCAAGGGAAAACAGCCTCTTTCTTCTACGCCG
TGGTGATGCCCATGCTCAACCCTTTCATCTACAGCCTGAGGAGCACGGAGGTGAGGAG
CACCCTCAGAAGAGCTACGAACGACCTCCCCCTGCATGTATTCCCACGGGTCCCGCT
CTACCCAAACTGAAGCAGATATCGCCGGCCGCAGTGGAGTCGGCAGAAGAAATAAGG
CTGACACAAGAGCACTGTACATTTCTGGACATCTGGTGA
>Cormorant_OR2D2 (Cormorant17)
ATGGCCAGGGAAAACCAAAGCATAGTGACAGAATTCATCTTTCAAGGCCTTTCCTCCC
AGCCAAGGACACAGACTGTTCTTTTCATAGTATTCCTGGGTTTTTATCTGTTCACAATTG
CTGGGAACATCATGATCATAACGGCAATCAGAGCTGATTGCCAGCTGCAGTCACCCATG
TACTTTTTCCTTGCCAACTTGTCCTTCTTAGATATCGCCTACGTCTCCAGCAACATCCCC
CAGATGCTGGTGAACCTCTTGACCAAGAAGAGGACCATCTCCTTCTCTGGATGTGCCG
CTCAGATGTATTTCTCTCTGGCTTTTGGCATGACAGAGTGTGTTCTGCTCGGAGTCATG
GCCTACGACCGATATATGGCAATATGTCACCCCTTGCTCTACACCACCGTCATGAACAG
GAAGGTTTGCATTCATGTGGTCGTGGCTTCCTGGACCAGCAGCCTACTGAGCTCCATG
GTCATCAACAGCCTCACCTTGCGGCTGCCCCTCTGCGGGCCTGGCATCTTGAACCATTA
CTTCTGCGAAGTGCCAGCAGTGCTGGCCTTGGCCTGCACCGACACTGCCCTCATGAAA
CTGGTCATCTTCATCTTCAGCATCCTCATAGTCTTCGTCCCCTTTCTTCTGATCATCACCT
CCTATGCCCGTATCCTTTCTGTCATCTTGAAGATTCAATCTGCACATGCACAATCCAAGG
CCTTCTCCACCTGTGGATCCCACCTGACGGTGGTAACCGTATTCTATGGGACAGCCATC
TGCATGTACATGAACCCTAAGACAAGGCCTCCACAGGATGGGGACAAGGTGATTGCAG
TGTTTTACACCGTTGTAGCCCCAATGCTGAACCCCCTCATCTACGGCCTCAGGAACAAA
GAAATGAAGCGTGCCCTGAGAAGGGCAATAAATAGACCCAAATCCCTGTTTATTTAA
>Cormorant_OR10A7 (Cormorant18)
ATGGAAGCTGTTGAAGTACCAGGGCTCAGAAACCAGACCTACTTCATCTTGCAGGGCT
TCTCCCACCTTGCTGCTCTCCAGGTCTTCCTCTTTGAGGGAATTCTTCTGATGTTTCTAA
TCATGATGACAGGGAACTTTCTCATCATCACAGTTGCAACCACTGACCTTCCCTTGCAC
ACCCCTGTGCACTATTTTCTAAAAAAACTGCCTTTAACTGAAATTTGCTTAACACTAAA
TGTAGTGCCCAGGTTGCTTGTGGATTTGTTTTTGGAGAGAAAGGTCATCTCCTTTTCTG
CCTGTGCACTACAACTTTACTTTGTCACACTCTTCATCACTTCCAAGTGTTTCCTCTTGG
GTGGCATGGCATATGACCGATACGTGGTTATATGCCATCCCTTGTACTTAGCCATAACAA
TGAACAAGAGAGTGTGTTTTCATATGGTCACAGCATGGTGCATTGCTGGTACCCCACTT
TCTGTAGGACTCACTGGTTGGTTATTTAGCTACCCCTTTTGTTGTTCAAAGGAGATTGA
ACATTTCTTTTGCGATATCACTCCCATTCTAGATCTGGTCTGTTCAGACACACGCTTATT
CAAACTTCTTGTGTTCATTGCTACTGTTGTACTTGTTTTGATCCCATTTATCTTAATAGCA
GCCTCTTATATCTGGATAATCCATACCATCCTCCAAATGCCATCTGCCGAAGAAAGATGC
AAAGCTTTTTTCACCTGCATTGCTCACCTGGTGGTGGTGACACTGTTCTACTGCACAGC
TGGCATGATACATTTAAAGCCAAAGTCCAGTCTCTTGGCAAACAGCAGGAAACTAACG
CCTCTTTTATACACCGTAGTAACTCCTATGCTGAATCCAATCATCTACACCTTATGA
>Cormorant_OR5V1 (Cormorant19)
ATGCAGAGGGCCAACCTATCAATGGTATCTGAATTTGTTCTCGTAGGCCTTTCGGATGC
TCCAGAAGTCCGTTTTCTTCTCCTTGTGCTGGTGTTGATCATTTATTTGGCCACCATGGC
AGGCAACATCGCAATCCTTGTTGTCACTAGCACAGACACTCGTCTGCACAACCCCATG
TACTTCTTCCTTAGCAACTTATCCTTACTGGATATCTTATGTCCCACTGTCACTGTGCCG
AAGATGCTGGGGGCCTTGTTGCTTGAGAACAAGTTGATTTCATTCACCGGCTGCATGCT

CCAGCTGTTCTTCCTCATTGATGTTGTAGGCACAGAGATTTTCTCTTGGCTGTGATGGC
ATATGACCGTTATGTTGCAATATGTCACCCGCTGCAATACATGAATATTATGAATATGAAG
CTGTGTGCTCACCTAGCCTTTGGCACCTGGGTAGTAGGATTTTTAATTCTCTGTTGCAC
ACATCTTTGATTTTTACGCTCTTTTTTTGCGATTCTAATGAAGTTCACCAATATTACTGTG
ATATTCCTCCTATGCTGGCCCTCTCCTGCTCGCCTCATTACAGTAGGGAACTGGTAATTC
TCACCGTTGCTGGGGTCCTCGGAAGCAGCGCCTTTGTGGTCACTCTGATCTCGTATATC
TATATCCTCTTGGCTATCCTGTACACGAGCACCTCCGAGAGCAGGCACAAAGCCTTCTC
CACCTGTGGTTCTCGCTTGACAGTAGTATGCCTTTTCTATGGGACCACCATTTGCACGTA
TGTACGGCCTTCCTCCACCTACTCACCTGATCAGGATAGGATAGTTTCTATGCTCTATGG
AATCCTCACTCCTCTGCTAAACCCCATAATCTACAGTCTGAGGAACAAAGAAATAAAAT
GTGCCCTAAGAAGAGTGATCAGCCAGGTAAGAACTGCTGTAACAAGACAAGAATATCT
CAGTTGCTGCTGCCCTCTGGAGCCTTAG
>Cormorant_OR6B1 (Cormorant20)
ATGCCCTCAGGTAACTTGACGCAAGTGGTTGAATTCATTCTGGTTGGTTTTCCAGGTAA
ACAGGAAATGAGGCTTCTGCTCTTTTTTATGTTCTTCCTGGCTTATGTGCTGACAGTGAT
AGAAAATGCAATGATTGTTGTGCTTGTTTGGACAAATCTCCAGCTTCACAAGCCAATGT
ATTTTTTCCTGGGCAATCTTTCTTTTCTGGAGATCTGGTACGTCTCTGTCACAGTGCCCA
AAGTGCTTGTGAGCTTGGTGACGAAGAGACAAGGCATCTCCTTCACAGGCTGCATGGC
TCAGCTATTCTTCTTCCTGGCACTGGCCTGCAGTGAGTGCACTCTCTTGGCTGTCATGG
CCTACGATCGCTATGTGGCCATCTGCAACCCATTGCATTACTCAGTCATCATGGATCACA
CTCTTTGCACCCACCTGGCCATTGGCTCCTGGATGAGTGGCTTCCTGATTTCCACAGGG
AAGGTTTACTTCATTTCACATCAGACCTACTGTGGGCCTAATATCATCAACCACTTCTTT
TGTGATGTCTCCCCCTTACTGAAGCTAGCCTGCACCAACATGTCAGTAGCTGAGCTTAT
GGATTTCTTACTGGCCCTGCTCATCCTTCTTGTACCACTCATTGTGATTATGGTCTCCTAT
GTGTGGATCATCTTCACTGTCTTGGGCATCCCCTCAGCCCAGGGGCGTCACAAGGCCT
TCTCTACCTGTGCCTCTCACCTCTTAGTGCTCACATTGTTCTACACAGCCTCCCTGTTTA
TCTATGCCAGGCCTCAGCCTATTGATTCCTTCAGCTCCTACAAACTGATTTCTGTGGTCT
ACACCGTACTGACACCCCTCATCAACCCGGTCATTTACTGCCTAAGGAACCAGGAATTC
AAAATTGCTTTTAGGAAAACAATATACTGGAGCAACATCTCATCATAG
>Adelie_OR51G2 (Adelie1)
ATGGAGCATGACTCACATACCACGTGGGAATTCAATGGCTCCTTCTATCAGCCTCCAGC
TTTCCTCATGATGGGCATCCCAGGCCTGGAAGCCCTTCACCACTGGATCTCCATCCCTT
TCTGTGCACTGTACCTTATTGCTCTCTTGGGAAACTGCATGATCCTATTCATCATAAAGA
AGACCCAAAGTCTTCACGAACCAATGTACTACTTCCTCTCCATGCTGGCAGTCACTGA
CCTGGGCTTGGTTCTATGTACGCTGCCTACTACTCTGGGTGTTTTTTGGTTTAATATGCG
AAGGATTGGGTTTGATGCTTGCCTCACTCAGATGTATTTCATCCACATACTGTCCTTCAT
TGAATCCTCTGTGCTCCTGGCAATGGCATTTGACCGCTTCATTGCCATCTCCCATCCACT
GAGACACCCATCCATACTGACCAAGACGACTGTCATAAAAATAGGACTGGCAATTGTAT
TGAGAGGTGTGGTCTCCCTCCTTCCCATACCCTTCTTGCTCAAGAGACTAACCTATTGC
AGGAAGACTGAGCTCTCTCATTCTTTTTGTTTCCATCCTGATATCATGAACCTAGCATGT
GCAGATATAAAAGTCAATGTCTTCTATGGTATGATTATTCTCTTAACAACAGTGGGGATG
GACTTCATCTTCATTGTGCTGTCCTACATCCTGATCATTAAAACTGTTATCAGCCTTGCA
ACCAAGGAGGAGTGTCTCAAGGCTCTGAATACATGTGTCTCCCACATCTGTGCTGTTCT
AGTGTTCTTCATCCCAATGATAGGACTGTCCATGATCCACCGCTTTGGAAAGAACGTTC

CTCCTCTGGTTAACACTTTGGTGGCCTACACCTACCTTATAATTCCCCCGCTCTCAACC
CCATTATCTACAGCATAAAATCCAGCCACATCCGTGAGGCTTTGCTCAGGGCACTGTGG
AGGAAGAGTGAATCTGACTGGTAG
>Adelie_OR2A2 (Adelie2)
ATGCAGAATGAAACGTCTGTCACAGAATTCATCCTCCTGGGGTTCTCCAGCAACCCAG
TCCTGCGGCTCTGCCTCTTTGGCATTTTCTCTGTCCTCTACTCTGCCACTCTGATGGGAA
ACGCACTTGTCTTTGTGCTTATCTGCCTGGACTACCGCCTCCACAGCCCCATGTACTTCT
TCCTCTGCCACCTGTCCATCGTGGACATCTGCTACGCCTCCAACAATGTTCCCCATATGC
TGAGGAACCTCCTTGGACAAGGCAGAACCATCTCCTTTGCTGGCTGTGGGACACAGAT
ACATCTTTATTTAATCTTTGCACTTACAGAGTGCGTGCTGCTGGCCGCGATGTCTTATGA
TCGCTACGTGGCAATCTGCCATCCCCTCCGCTATGCCCTTATCATGAACCGGAGGGTGT
GCCTCACCCTTGCTGCAGTTTCATGGGCTTTTGGGTTCCTATTTGGCACGCTACAAGCC
TCTCTGGCTTTACACCTGCCTTTCTGTGGCCCCTGCGAGGTTGACCACTTCTTCTGTGA
AATCCTTGCTGTCTTAAAGCTGGCCTGCACTGACACTACTGCCAATAAAGTCCTGATCT
TTGCTGTTTGCGTGTGCTTCCTCCTCTTCCCTTTAGCCTTAATCCTAATTTCCTACCTGCA
CATCCTGGCCACCATTCTGCGCATCCGCTCTGCGGCAGGATGGCAAAAAACCTTCTCC
ACCTGTGGCTCCCACCTGGCCGTGGTGGGTCTGTTTTATGGAAACGCCATCTTCATGTA
CATGGGGCCCGGGAGCGGTAACTCATCTGGGAGGGAGAAAGTTCTTTCCCTTTTCTAC
AGTCTCGTCAGCCCAAGTTTGAACCCCCTGATTTACAGTCTGAGGAACAAGCAGGTGA
AGGAAGCCTTGCTGAAACTTCAGAGAAGGAAAAGAGTTTTTCATTCCGTGTAG
>Adelie_OR5J2 (Adelie3)
ATGCAGGACGGTAACGTGGCTAAAGGCAATCACACCATGGTGACCCAGTTCATCCTCC
TAGGACTGACAAGCGAGCCTAAGCTGCAGACGCCTCTCTTCGTAATCTTCTTGATGATT
TATCTCATCACCCTGATGGGCAATCTTGGGCTGATCGCATTGATGAGGACAGACCGCCG
GCTGCACACTCCCATGTACTTCTTCCTCTGCAATCTGTCTGTTGTTGATCTTTGCTACTC
CTCCGTCTTTTCTCCAAAGCTGCTTATTGGCTTCTTGGTGGAAAAGAAAACCGTTTCTT
ACTCCGCCTGCTTTGCCCAGCATTTCTTTTTCCTTGTGTTTGTGACCACGGAGGTGCTC
TTGCTGGCTGTGATGGCGTACGACCGCTACGCAGCCATTTGCAACCCGCTGCTCTACAC
TATTTCTATGCCCAAGAGGGTCTGCATTCAGCTGGTGGCCGGGTCGTACGCAGGGGGG
ATCCTGAACTCACTAATCCAAACCTGTTGCTTGCTGCCGTTGCCTTTTTGTGGACCCAA
TGTCATCAACCATTACTTCTGTGACACTAACCCTCTGCTGAAACTCACCTGCTCCGATG
ACCACCTCAATGAGCTCTTGCTTGTAACCTTCAATGGGACCATTTCCATGTCCGTGCTC
CTCCTCATCATCATCTCGTACGTATACATCCTCTTCGCCATCCTGAGGATTAGGTCCGCC
GAAGGCAGGCACAAAGCCTTCTCCACCTGTGCCTCCCACCTCCTGACTGTTACCTTGT
TCTACGTGCCCGCGGGGCTGAGCCACATGCAGCCGGGCTCCAAGTACTCCCTGGAGGT
GGAGAAAGTCACCGCCGTGTTTTATACCCTGATCGTCCCTATGCTCAACCCTCTGATCT
ACAGCTTGAGGAACAAGGAGGTCAAGGATGCACTTCGAAAAGCAACAGCAAATAACA
TTTTTGCGAGTTGCCTGCTGACCAAACTGACCCTGATCAGTTGA
>Adelie_OR5V1 (Adelie4)
ATGGAAAACCAAACAAGTTCAAGTGAGTTTGTTCTCTTGGGACTTACAAGTGACCCAC
ACCTTCAAAGCCTCCTCTTCTTTGTTTTTTCAGTTATTTATTTAATCACTCTGTTTGGAAA
TATGGTGATCATGATTGTGATAAGTACTGATCCCCACCTTCACTCCCCTATGTACGTCTT
CCTTTTTCATTTAGCCCTCACAGACATCTGTTATGCTACCACCATTATTCCTTATATGCTG
GTGAAGTTCCTACTGAAGCAGAGAACCATTGACTTCAGTGCCTGTATTATCCAGATGTC

CTTAATCCTTCTCTCAGCTGGTAGCGAAATTTTCATGCTCTCAGCAATGGCATATGACCG
ATACATTGCCATCTGTAAACCACTACAATACCAAGAGGTTATGAACAAACCTGTCTGTA
GCCAGCTGGTGGGGGGTGCATGGGCAATGGGGGTCTTACACTCCATTATAAACACACT
GCCGATGCTAAATGTGCAATTCTGCAAGTACACAGAAATTAAGCATTTCAGCTGTGAGT
TGCCCCCTCTCTTAACTGCAGCCTGCGATAGGACCTTCCTCAATAAACTTGTTCTTCTGT
CTTCTGCTGTGATCTTTGAGTCAAGCTCCTTTCTGCTCATTCTCATCTCCTATATCTATAT
CATCTCTACTGTCCTGAAGATAGAGCCTGCAATGGGGATACAGAAAGCTTTCTCCACTT
GCAGCTCCCACCTCATCATAGTGGTTTTGTTATACACAACTGCTTTGTTCCAGTACACA
AAACCCAGTTCAGTCTCATCATTCATTCTAGATCAACTGTTTTCTATCCAACACAGCATT
TTAACCCCCATGCTAAATCCCATCATCTACAGCCTGAAAAATAATGATGTGAAAACAGC
TTTGGGCAGAATGTTAGGGAAAATTCAAGTTTCGCAATCAGTGTAA
>Adelie_OR11L1 (Adelie5)
ATGACAAATGTTACAGCAGTACTGGAATTCAGGCTACTGGGCTTCAGTAGCAACCCAC
ACTGCCAGATCCTACTATTCACAGTGTTTTTAGTTATTTATATTCTCACCATCCTAGGAAA
CATCATTATTATTTTAGTGGTGACACTGGAGCCACAACTTCATTCACCCATGTACAAATT
TCTCAAGAACCTCTCTTTCCTAGAGGTCTGTTACACCACCACAATTGTACCCAAGATGC
TGGCCAATCTACTGGCAAAGAGGAAGAGCATATCCTTCTCAGGATGCATGGCACAGCT
TTATTACTTCATTTCCCTGGGAGCCACTGAGTGCTACCTCTTGGCAGTGATGGCATATGA
CCGATACCTTGCCGTCTGTGAACCCCTGCACTACGGTATGGCCATGACTGCTGAGTTTT
ATACCCGTCTGGCTGTGGGCTCCTGGGTCACTGGCGTTTTCACTGGTTTTCTGCCCTGT
CTGATGGTCTCCAGATTGCATTTCTGCAGTTACAACCTCATTGATCACTTCTTCTGCGAT
ATCTCCCCACTGCTGAAGCTCTCATGCTCAGACACCACTGCCACAAAAAATGTCATCTT
CATCCTCTCTCTCCTGGTCCTTTCCAGCTGTTTTCTGTTGACTCTTGTCTCATACCTACTT
ATAATTCTCAGTATCCTGAAGATACCCTCTGCTTCTGGAAAAAGAGTTACCTTTTCCAC
CTGCAGCTCACATCTCATGGTAGTGACTATATACTATGGTACAATGATTTCCATGTATGTC
CGTCCCACCTACAACCTCTCCTCAGAGCTCAATAAGGCTGTATCTGTGCTCTACACAGT
GGTCACACCCCTTCTGAACCCAGTAATCTACAGCTTGAGAAACAAGGCATTCAAGAAG
GCCTTGGAAAAAATAGTCATCAGACACCATCGTCTTCATTCTTTCTAA
>Adelie_OR5B21 (Adelie6)
ATGGAGAGAGGGGAATGGGACAACCAGACGTTGCTGATGGAGTTCCTCTTGCTGAGA
CTAGGGGATGCCCATAAGCTCCAGACACCTCTCTTTCTCCTCTGCCTGACCATATACAC
AGTGACCATGGTTGGGAATGTCCTCATCATTGTGCTGGTGGTCATAGACCCGCATGTCC
ATACAGCCATGTACTTCTTCCTGGTGAATCTATCCAGCCTAGAGACCTGCTACAGCTCC
ACCATCCTGCCCAGGCTGCTAGCCAGCTTCCTGACTGGAGACAGGACCATCTCTGGGC
AGGGATGTATGGCACAGTTTTTCTTCTTCGGCACTTTTGCAACTTCTGAGAGTTACCTG
CTGGCTACCATGTCCTATGATCGGTATCCGGCCACGTGTCAACCTCTGCTTTATGCAAGC
CTCATGAACTGGAAAGTATGTCTCCAACTGGTGGCTGGATCATGGGTTGTGGAACTGCT
AATTTCTACAGGTATCATATCTTTCATATGTCGCCAAACGTTCTGTGGCCCCAGTGCAAT
TGACCATTTCTTCTGTGAAGAAGCTCCATTGCTAGAACTCTCCTGCACTGACATTACGA
TGATCAGAATTCTTATTATTATATTATCTTTCCTGGACGTAGTTTTCCCATTTCTGTTCACT
TTGGCATCTTATGTCTGCATGATAGCTGCCATCCTGAGGATCCCATCCAGCATGGGGAG
GCACAAGGCCTTTTCCACCTGCTTCTCTCACCTCCCTATGGTCATTGTTTTCTGTGAGA
CCCTCATCATTCTCTACATGCTGCCCAGAACAGCACCACTGAGACAGCTCAACAAAAT
GTTCTCCTTTTCTTACTCAGTCCTCAGACCTCTCACCAGTCCACTCATCTACAGTCTGA

GGAACAGAGGCATCAAGGGGGCAGTGGGGAGGGTGCTCAGGAGGGTGCTGCCTGCA
CTGGCAGCTCCAACCAGGTGTGGATTTCAGAGCACAAATCTGGTTCTCCTGCAAAGTG
ACGAAAGGAACATAGGAAGGAATGTGCTCCATCTCAAGAGTACCTCTGCAAATGTCTG
GCTTTCACCTAGGATGTGCAATGGCAAGAGGCAGAGGGAGAAGAGGGCCTAG
>Adelie_OR6B1 (Adelie7)
ATGGAGGAGAAGAATTCCACTCAGGTCCACATGTTCCTCTTGCTAGGATTCCCAGCCCT
ACCGGACTTGCATGTACTGTTCTCCATAGTATTTCTGCTGACCTACATTTTAACTGTTTT
GGAGAACGTGGTCATCATTGCCCTGATCAAGACAAACTGTGAGCTCTACAAACCCATG
TATTTTTTCCTTGGTCACCTCTCCTTCATTGAGGTCTGGTACGTCTCAGTCACTATCCCT
AAACTCTTGGCAAATTTTATTGCTGAAGACAGGAGTATTTTCTTTGCGGGATGCATGAC
CCAACTGTTTTTCTTCAGCTCCTTCATGTGCACTGAGTGTGTCCTTCTCTCTGCAATGG
CATACGACCGCTATGTGGCCATCTGTCAACCGTTGCGCTATCCGGTCATGATGACGTAC
CAAATGTGCGTTTACCTGGTAGTTGTCTCCTGGTTCAGCGGGTTCACTGTGTCTTTGAT
CAAGATTTCCTTCATCTCTCAGCTGAACTTCTGCGGCCCCCATGTTATCAACCATTTTTT
CTGTGATGTTAGCCCTGTGCTGAACCTCGCCTGCACTGATACGTCACTGGCAGAGGTG
GTGGACTTTGTGTTGGCCTTGTTCGTACTGCTTGTTCCCCTCTTCATCTCTATTGTCTCCT
ACCTATTAATTGTCATGACAATCCTGCACATCCCCAATACCCAGAGTAAGAAGAAAGCC
TTCTCCACGTGTTCTTCCCACCTAACTGTGGTGACCGTTTTCTTCTCAGCCACCCTCTTC
ATGTATGCCCGGCCCAAGAAGATCGACCCTTTTGACTTGAATAAGCTTGTGTCAGCTGT
GTATACTATTGTCACTCCCATCCTAAACCCCTTCATTTACTGTCTGAGGAACCAGGAAGT
GAAAAGGGCACTGAAAAAAGCTCTCTGTGAAAAAATGAGTGTCTCTAAGGTCTCCCTT
CTCTCCACATGCAAGGATAAGCTATGA
>Adelie_OR6B1 (Adelie8)
ATGAAGCAGAAAAACAGCACAAAATTCCAGGAATTTATCTTCTTGGGATTTCCAACTAT
CATGGAACTTCATATGTTGCTCTGTGTGATATTCCTGGTGGCTTACATGCTGACCATCTT
GGAAAATATAATTATCTTGATAAAGATGAACCATCAGCTTCACAAGCCCATGTATTTCTT
TCTCAGCAACCTCTCCTTCCTGGAGTCTTGGTACATCTCAGTCACTGTCCCTAAACTGC
TAGTGAATTTTCTTGTGGAAAGCAAGAATATATCTGTTGGAGGCTGTATGACCCAGCTT
TACTTCTTCAGCTCCCTTATTTGTACTGAGTGTGTCCTCCTTGCACTGATGGCTTATGAT
TGTTATGTGGCCAGCTGCAATCCCCTGTGCTACCCAGTCATCATGAACCACCAGCTCTG
CATGCAACTAGCCACGTGCTCCTGGCTCACTGGCTTCTTGGCCTCCATGCTGAAATTAT
TTTTCATTTCTCAGTTGTCTTTCTGTGACTCCAACGTCATCAACCACTTCTTCTGTGACA
TCAGCCCCTTGCTGAACATACGATGTGCTGATATGACAATGGCTGAAATAGTGGATTTC
ATCCTGGCCTTGCTTATCTTGCTGGTTCCCCTCTCTGTCACGATTATCTCCTATATATGCA
TCATCAAGACCATCCTGCATATCCCCACAGCCCAAAGCAGGAAGAAAGGCTGCTCCAC
CTGTGTTTCTTACCTCACTGTAGTCATTATCTTGTTTTCAGCCACTCTGTTTATGTATGCC
TGGTCCAAGAGAATCCACCCTTTTGACTTCAACAAGTTGGTGTCAATTGTATACACTAT
TGTAACTCCCATGCTCAATCCCTTCATTTACTGTTTGAGGAACTAG
>Adelie_OR51E2 (Adelie9)
ATGCCCTTCCCCAACAGGTCTGACCTCAGCCCATCCTCCTTCATCTTGGCCAGCATCCC
GGGGCTGGAGGCTGACCATTTCTGGATGGCGATCCTTCTGTGCTCCATGTACATCTTGG
CAGTCACAGGCAACTGCGCGGTGCTGTTCATTGTGAAGACAGAGCCCAGCCTGCACG
CTCCCATGTACTTCTTTCTCTGCATGCTGGCTGCCATCGACCTGGCCTTGTCCACGTCCA
CAGTGCCACGTGTTCTCTCCTTCTACTGGTTCAACACCAGAGAGATCAGCTTCGGCGC

TTGCCTTGTCCAGATGTTCCTCATCCACACCCTCTCGGCCATCGAGTCCACTGTCCTCC
TGGCCATGGCCGTGGACCGGTACGTGGCCATCTGCCACCCGCTGAGACACGCTGCCAT
CCTCACCAACGCTGTGACAGTGAAAATAGGGCTGGTAGCCATGGCCAGGGGAGTTCTT
TTCTTCCTGCCTTTGCCTTTGCTCCTCCTGCCCCTTCCATTCTGCAGCTCCCGGGTGCTG
TCACACTCCTTCTGCCTCCACCAGGATGTGATGAACCTGGCCTGCGCCAACACCACCC
CTAGTGTGGTGTATGGCCTCACCGCCATCCTGCTGGTCATGGGGCTGGATGCCATCCTC
ATCTGCCTCTCCTACATCCTGATCCTCAAGGCTGTCTTGCGGCTAGCATCGTGGAAGGA
GAGGCTTAAGGTGTTCAGCACCTGCATTGCCCATATCTGTGTGGTCCTAGCCTTCTACG
TGCCCTTGATTGGGCTGTCCGTGGTGCACAGGTTTGGGAAGGATCTGGCTCCATTGGT
CCATATCATCATGGGGAACATCTACATCCTGGTGCCTGCTGTGCTCAACCCCATCATCTA
TGGGGTGAGGACCAAACAGATACAGAGGAGGATCCTGAATTTAATTCATATATACAATG
ACAGAACTGCCCAGTGA
>Adelie_OR10K2 (Adelie10)
ATGGAGAGGGACAATAAGAGCACTGGTGAAACCATGGAGTTCCTCCTGCTTGGCTTCT
CCGAGCTGCTCTGTCTGCGGGTCCTCCTCTTCCCTATCTTTCTCATTGTCCATTTGGTCA
CATTGGCAGGGAATGTGATGATCTTCATGGCGGTGGTTATGGAGCCTTCCCGTCCTCCC
ATGCTTTTCTTCCTCTGTCAGCTCTCTGTCATCGAGCTCTGCTATACTTTAGTCATTGTCC
CTAAGGCACTCCTCGGCCTGATAGTGGTGGACGGCAGCACCATTTCTTTCATAGGCTGT
GCTGCACAGATGCACCTTTTTGTGACACTCGGTGGGGCTGAATGCTTCCTCCTGGTAGC
CATGTCGTACGACCGTTATGTTGCCATCTGTCAGCCACTTCACTATGTAGCTGTGATGAG
TGAGGGGCTCTGCCTCAGGCTGGCTGTGGCATGCTGTCTGGGAGGCTTTGCTGTTGCT
CTGGGGTTGACGGTGGCTGTTTTCCGCTTACCTTTCTGCCAGTCACATCATATCAACCA
CTTCTTCTGTGATGTCCCTGCTGTGCTGCACCTGGCCTGTACACAGAGTTACACCCCTG
AGCTGCCCTTGCTGGCTGCCTGTGTGCTCCTCCTGCTGCTCCCCTTCCTCCTAATCCTG
ACCTCATATGTTTGCATTGCTGCTGCTTTGCTACATGTCACCTCCTCTGCAGGAAGGGG
CAAGGCCTTTTTCACTTGCATTTCACACTTGTCCATCACCTTGCTACACTATGGATGTGC
CACCTTCATGTACATTCGTCCTAAGTCCAGTTACTCGCCAGCTCGGGACAAGATGGTGT
CTCTGGTCTACACCAACATTACTCCATTATTGTATCCCCTCATTTATAGCCTGAGGAACG
AGGAGATCAGAGGGGTCATCATGAAAATGTTGAGGAGGAAGAAAATAGCTCAGCTGA
ACTGGGATACTATCAGAGCTGTGATATGTGTGTGTGGTAAATTTTAG
>Adelie_OR5AR1 (Adelie11)
ATGGCAGAGCAGAATCACACCTCAGTGGCAGAGTTCATTCTCGAGGGCCTGAGTGACC
ACGCGGAGATGAAGGCACCCCTCTTTGTGCTGTTCCTGCTCATCTACACCGTCACCCTT
TTGGGCAACGTGGGGATAATCGTAGTCATCCGAGGTGACCCACGACTCCATACCTCCAT
GTACTTCTTCCTCGGCAGCCTCTCCGTTGTTGACATCTGCTTCTCCTCTGTGATCGCCCC
CAGGACCTTGGTGAGCTTCCTATCGGAGAGGAAGACTATTTCCTTCGTTGGCTGCATGG
GCCAAGCCGCCTTCTACATCGTCTTTGTGACGACTGAGTGTTTCCTGCTGGCCGTCATG
GCGTATGACCGGTACGTGGCCATCTGTAACCCCCTGCTCTATTCCTCTGTTATGACTCGG
AGGTTGTGCATGTGGCTGCTGGTGGGGTCCTACACTGGGGGTGTCCTGACCTCCCTCAT
ACAGATGACCTTCATCGTTAGGCTGCCCTTCTGCAGCTCCAATGTCATCAACCACTTCT
TCTGCGACGTTCCTCCCCTCCTGGCTCTGTCCTGCGCCAGCACCTACATCAATGAGATG
ATCCTCTTCTCCTTGGCCGGCGTCATCGAGCTCAGCACCATCTCCACCATCCTGGTCTC
CTACACCTTCATCTCCTTTGCCATCCTGAGGATCCGTTCAGCTGAAGGCAGGCAAAAA
GCCTTCTCCACCTGCGCGTCCCACCTGACAGCAGTGACCATGTTGTATGGGACGACAA

TCTTCATGTATTTACGCCCCAGCTCTAGTTACTCCCTGAACACTGACAAAGTGGTCTCC
GTCTTCTACACGGTGGTCATCCCAATGCTGAACCCCCTCATCTACAGCCTGAGGAACCA
GGAGGTGAAGGATGCTCTGAGGAGAACAGCAGAAAGAATCACAGTCAGGCTCCGAG
CCCTGCTCGATAAAACCTGA
>Adelie_OR2B2 (Adelie12)
ATGGCTAGGGAAAACCAAAGTGTAGTGACAGAATTCATCTTGCAAGGCCTTTCCTCCC
AGCCAAGGACACAGACTGTTCTTTTCACAGTGTTCCTGGTTTTTATCTGTTCACAATT
TTTGGGAACATCATGATCATTACAGTGATCAGAGCTGATTGCCAGTTGCAGGCACCCAT
GTACTTTTTCCTTGCCAACCTGGCCTTCTTAGATATCTGCTACGTCTCCAGCAACATCCC
CCAGATGCTGGTGAACCTCTTCACCAAGAAAAGGACCATCTCCTTCTCTGGATGTGCT
GCTCAGATGTATTTCTCTCTGGCTTTTGGCATGACAGAGTGTGTTCTGCTTGGGGTCAT
GGCCTATGATCGATATATGGCAATATGTCACCCTTTGCTGTACACCGCTGTCATGAACAG
GAAGGTTTGCATTCACATGGTCATGGCCTCCTGGACCAGCAGCCTGCTGAGCTCCATG
GTCATCAACAGCCTCACCTTGGGGCTGCCCTTCTGCGGGCCTGACATCTTGAACCATTA
CTTCTGCGAAGTGCCAGCAGTGCTGGCCTTGGCCTGTGCTGACACTGCCCTCATGGAG
TTGGTCATCTTCATCTTCAGCATCCTCATAGTCTTCATCCCCTTTCTTCTGATCATCACCT
CCTACGCCCATATCCTTTTCGCCATCTTGAAGATTCAGTCTGCGCATGTGCAATCCAAG
GCCTTCTCCACCTGTGGATCCCACCTGACGGTGGTAACCATATTCTATGGGACAGCCAT
CTGCATGTACATGAATCCTAAGTCAAGGCCTCCACAGGACAGGGACAAAGTGGTTGCA
GTGTTTTACACCATTGTAGCCCCAATGCTGAACCCCCTCATCTACAGCCTCAGGAACAA
GGACATGAAGCGTGCCCTGAGAAGGGCAATGAATGGACCCAAATCCCTGTTTATTTAA
>Adelie_OR52B2 (Adelie13)
ATGGCGGCTCTCAATCAAACCAGCTTACAGCCTGCCTCCTTCCTCCTGCTGGGCATGGC
AGGCCTGGAGGACCTGCACACCTGGCTCTCCATCCCATTCTGCATGATGTACATCGCGG
CGCTCCTTGGCAACTTCATCCTCTTATTTGTCATTGTGACGGAGCGAAGCCTCCATGAG
CCAATGTACCTCTTCCTGGCCATGTTAGCCATGGCAGATCTCGTATTATCCTCCTCCACA
GTGCCCAAAGCCCTGAGCGTATTCTGGTCCCTTTCCAAGGAGATTTCTTTCCATGCCTG
CCTTACCCAGATGTTCTTCACACACCTGAGCTTCATTGCAGAGTCGACCATTCTGCTGG
CCATGGCATTTGACCGGTACGTGGCCATCTGTAACCCCCTGCGATATGCCACAGTGTTC
ACGCACTCGGTGATAGCCAGGATAGGGCTGGCTGCAATAGCCAGGAGCTTTTGTGTGA
TGTTCCCAACAATATTCCTCCTTCGGAGGCTGCCATACTGCAGACACAACATCATGCCG
CACACCTACTGCGAGCACATGGGCATCGCACGGCTGGCCTGTGCCGACATATCTGTTAA
CATCTGGTACGGCTTTGCCACCACCCTTCTGTCCCCAGGAGTGGACATTGTGCTCATCG
GGGTATCGTATGTCCTCATCCTCCGGGCTGTCTTCAGGCTCTCATCCAAGGATGCGCAG
CTCAAGGCAGTTGGCACCTGCAGCTCTCATGCCTGCGTTATATTAATATTCTACACTCCA
GCATTTTTCTCATTTTTCACTCATCGGTTTGGCCGCAACGTCCCCCACCATGTTCACGTC
CTGTTGGCCAATCTCTATGTGCTCTTGCCACCTATGCTAAACCCCATCGTCTACACTATG
AAAAACAAACACATTCGAGAAAAGGTGTCCCAAGTACTCTTCAGGGCTGGGCAGGTG
CGGTGA
>Adelie_OR5R1 (Adelie14)
ATGGGAGGAAATCACGTGCAGCCTAAATTCATCCTCTTGGGAATTACAGACAGTCCGT
GTGTGCGGGCCCCTCTTTTTGGGTGGTTTCTATTGATTTACATTGTCACTGTGGTGGGG
AACGTTGGGATTATGGTCTTGGTTTGGGTGGTTCCCAGCCTCCACACCCCCATGTACTT
CTTCCTCACCCATTTTTCATTCGCTGACGTCTGCTATTCCACAGTCATTTCCCCCAAAAT

GCTAGCAGACCTCTTATCAGAGAATAAAACCATTTCTTTCGCTGGCTGCGTGACGCAGT
TCCATGGCTTTGCTTTCTTTGCGACTGCCGAGTGTCACCTCCTGGCTGTGATGGCCTAT
GACCGGCACGTTGCTATCTGCAACCCCCTGCTTTACGTGACGGTCATCTCCAGCCGCGT
CTGCCGGCAGCTGGTAGCATCGTCCTACCTCATCGCCTTTCTCAGTGCCATCATCTACA
CAGGCTGCACGTTTGGGGGTTCCTTCTGTGGACCCAACCAGATCAACCACTTCTTCTG
CGACGTCAGCCCCGTGCTAAAGCTTGCCTGCTCCGACACCCACAGCAGCGAGATGGTC
ATCTTTGCCCTTGTCGCCATAAACGCGGTGGGCACGAGCGTGATCATTTTGCTTTCCTA
CGTCTGTATCCTCCGCACAGTCCTGAGGATGCGCTCGGCACAGAGCAGGTCCAGAGCC
TTCAACACCTGCGCCTCCCACTTGACGGCTGTCTCCTTATTCTACGGGACAATATTCTTC
ACGTACCTACAACCTGCGTCTAGCCACAGCAGCCTGGATAAGGTGGCCTCCATCTTCTA
CACCGTGGTCACCCCCATGCTCAACCCATTCATCTACAGCCTGAGGAACAAGGAGGTG
AAGGGCGCTCTGGTGAAGTGCAGGAGAAGGATGTTAAACCGCTGTCAACATAGAAGA
GCTGTATCAGCTAGGAAGTGA
>Adelie_OR52B2 (Adelie15)
ATGTATGAGCTCAATGAAAGCAGCTTTGATCCTATCACCTTTGTCTTGACGGGCATCCC
GGGAATGGAGTCGTCCCACATCTGGATCTCTGTCCCCTTCTGTCTGATGTACATCACTG
CGGTGTTTGGCAACTCTGTCCTCCTCTTCGTCATCATCACGGACAGGAGCCTCCATGAG
CCCATGTACCTCTTCCTTGCTATGCTGGCTGTTGCTGACCTTATGCTTTCGACCACGACG
GTGCCCAAAATGCTGGCTATCTTCTGGTTCAGTGCCAGGGAAATTTCCTTCGATGCCTG
CATTACACAGATGTTCTTCACCCATTTCAGCTTCATTGTGGAATCATCCGTTCTGCTGGC
GATGGCATTTGACCGGTACGTGGCCGTCTGTGACCCGCTGCGGTACTCTTCAACCTTAA
CCCCCTCGGTGATTGGGAAAATAGCTGTGACTGCTGTTGTCCGGGGGTTCTGCATCATG
TTCCCACCCATCTTCCTCCTGAAGCGGCTGCCGTACTGCGGACACAATGTCATGCCCCA
CACCTACTGTGAGCACATGGGCATCGCCCGCCTGGCCTGCGCCGACATAAAAGCCAAT
GTCTGGTACGGGCTGACAACAGCTCTTCTCTCCTCCGGCCTGGACGTCGTGCTCATCA
CTGTGTCTTACGCTCTGATCCTCAGGACGGTCTTTCGACTCCCGTCCCCGGAGGCCCGT
CTCAAAACCCTGAGCACCTGTGGCTCCCACCTCTGCGTGATCCTCATGTTCTACATGCC
TGCCTTTTTCTCCTTTCTCACGCATCGGTTTGGCCACCAAATCCCCAGTCACGTTCACA
TCCTCCTGGCCAACCTCTATGTCGTGGTCCCGCCGATGCTCAACCCCATTGTGTACGGG
GTGAGGACAAGGCAAATCCGGGACCGCGTCGTCCGCCTCTTCTGCCCCACGGGGGAG
TGCCCCTGCCCCAGCTGGGGGAGCAGGTGCTGA
>Adelie_OR5B21 (Adelie16)
ATGGAGAGAGGGGAATGGGACAACCAGACGTTGCTGATGGAGTTCCTCTTGCTGAGA
CTAGGGGATGCCCATAAGCTCCAGACACCTCTCTTTCTCCTCTGCCTGACCATATACAC
AGTGACCATGGTTGGGAATGTCCTCATCATTGTGCTGGTGGTCATAGACCCGCATGTCC
ATACAGCCATGTACTTCTTCCTGGTGAATCTATCCAGCCTAGAGACCTGCTACAGCTCC
ACCATCCTGCCCAGGCTGCTAGCCAGCTTCCTGACTGGAGACAGGACCATCTCTGGGC
AGGGATGTATGGCACAGTTTTTCTTCTTCGGCACTTTTGCAACTTCTGAGAGTTACCTG
CTGGCTACCATGTCCTATGATCGGTATCCGGCCACGTGTCAACCTCTGCTTTATGCAAGC
CTCATGAACTGGAAAGTATGTCTCCAACTGGTGGCTGGATCATGGGTTGTGGAACTGCT
AATTTCTACAGGTATCATATCTTTCATATGTCGCCAAACGTTCTGTGGCCCCAGTGCAAT
TGACCATTTCTTCTGTGAAGAAGCTCCATTGCTAGAACTCTCCTGCACTGACATTACGA
TGATCAGAATTCTTATTATTATATTATCTTTCCTGGACGTAGTTTTCCCATTTCTGTTCACT
TTGGCATCTTATGTCTGCATGATAGCTGCCATCCTGAGGATCCCATCCAGCATGGGGAG

GCACAAGGCCTTTTCCACCTGCTTCTCTCACCTCCCTATGGTCATTGTTTTCTGTGAGA
CCCTCATCATTCTCTACATGCTGCCCAGAACAGCACCACTGAGACAGCTCAACAAAAT
GTTCTCCTTTTCTTACTCAGTCCTCAGACCTCTCACCAGTCCACTCATCTACAGTCTGA
GGAACAGAGGCATCAAGGGGGCACTGGGGAGGGTGCTCAGGAGGGTGCTGCCTGCA
CTGGCAGCTCCAACCAGGTGTGGATTTCAGAGCACAAATCTGGTTCTCCTGCAAAGTG
ACGAAAGGAACATAGGAAGGAATGTGCTCCATCTCAAGAGTACCTCTGCAAATGTCTG
GCTTTCACCTAGGATGTGCAATGGCAAGAGGCAGAGGGAGAAGAGGGCCTTAGGGAA
ACACATGCCCAGCTGA
>Adelie_OR11A1 (Adelie17)
ATGAGAAGAGCAGAGCAGGGCAATGAAACAGCTGTTGTGGAATTCATCATCCTCGGTT
TCCAAAGTATCCCAGAAGTACAGTTTATCCTCTTCCTGTTGTTTCTAGTGACCTACATTA
CAACTGTGTCAGGGAACCTCCTGATCATGATGCTGGTTGTGACTGATCATCACCTTCAT
AACCCCATGTTTTTTTTCCTGGGGAATCTGTCATTCTTGGAGACCTGTTACACTTCCACC
ATCCTGCCCAGAATGCTGGCAAGTTTCCTAACAAACAAATATGTTATTTCACTTAGTGG
TTGTTTTCTGCAGTACTTTTTCTTCACTGGGCTAGCAGGTGCAGAATGTTGTCTCCTGA
CTGTCATGTCTTATGACCGGTACATTGCGATATGCAAACCATTGCATTACACAGCCATAA
TGAATGGCAGGTGCTGCCTCCAGTTAGCAGGTGGATCTTGGGCAAATGGGTTGTTGGC
TAGTGCCATAGTCACCTCTCTGATGCAACAGCTAACTTTCTGTGGCCCAAATGAAATTG
ATCATTTCTTCTGTGACTTCATGCCGGTGGTAAAGCTCTCCTGCAGTGATACCCACTGG
ATTGAGCTTGTAACGTTCATCTTGGCCTCCCTTTTGACACTACCTCCATTTCTCTTGACA
CTCATATCTTATGTGTACATCAGCAACACTGTCCTCAGGATCCCATCCTCCACAGGAAG
GCATAAGGCTTTTTCTACCTGTTCTTCCCATCTCACTGTGGTGACACTTTTCTATGGGAC
CCTGATTGTTGTGTACTTGGTGCCAAAACCAAACACACTGGGAGACCTCAGCAAAGTG
TTCTCTGTCTGCTACACAGTTCTGACTCCACTGGTCAATCCCGTCATCTACAGCTTAAG
AAATAACAACATAAAGAAGGCTTTGAGAACTGCTGTGAACAAATACAGGGGGTTTGAT
AAGGCGTGA
>Adelie_OR4S2 (Adelie18)
ATGGAGAATGCAAGCAGTGTGAAGGAATTCATTCTTCTGGGCCTCTCAGAGAATCAAG
CGGTGCAGAAAATATGTTTTGTGATGTTTCTGTTCTTCTATATGGTTACTGTGGCAGGAA
ATCTGCTCATTGTTATCACTGTAATTAGCAGTCAATGTCTGAACTCTCCCATGTATTTCTT
CCTCTGCTACCTGTCCTTTGTAGATATCTGTTACTCTTCCGTCACAGCTCCCAAAATGAT
TGCCAACTTCCTTGTTGAAAATAAAACCATCTCCTTTGTGGGTTGCATAGCACAGCTGT
TTGGGGTACATTTCTTTGGCTGCACAGAGATCTTCATCCTCACAGTGATGGCCTACGAT
CGCTATGTTGCCATCTGCAGACCTCTCCACTATACCACCCTCATGACCAGGCGTGTGTG
TGGCTGGATGGTGATCGGCTCATGGGTAGGAGGTTTCGTGCACTCCACAGTGCAGACT
CTTGTAACCACTCAGCTCCCCTTCTGTGGCCCTAACAAAATTGACCACTACTTCTGTGA
TGTCCACCCCCTACTACAACTGGCCTGTACCAACACCTACGCTGTAGGCATCATTGTCG
TTGCCAACAGCAGAATGATAACTCTGAGCTGTTTCTTCATCCTGGTCATGTCCTACGTT
GTCATCCTGGTTTCCTTGAAAAGTCAAACATCCAAAGGGTGGCACAAAGCCCTCTCCA
CCTGTGGGTCCCACATCGCTGTGGTGATTCTGTTCTTCGGGCCATGCATGTTCATCTACA
TACGTCCATCCGGCAATCTGTCAGCAGACAAGAGCGTGGCAGTGTTTTACACTGTCAT
CACACCCATGCTGAACCCACTCATCTACGCGCTAAGAAATAAGGAGGTGAAGAGTGCC
ATGAGAAAACTATGGAGTAGAAAAGTGGGAAGTGAAAATGGAAAGGTGTAG
>Adelie_OR14J1 (Adelie19)

ATGTCCAACAGCAGCTCCATCACCCAGTTCCTCCTCCTGGCATTCGCAGACACGCAGG
AGCTGCAGCTGTTGCACTTCTGGCTTTTCCTGGGCTTCTACCTGGCTGCCCTCCTGGGC
AATGGCTTCATCATCACAGCCATAGCCTGTGACGACCGCCTCCATACCCCCATGTACTT
CTTCCTCCTCAACCTCTCCCTCCTCGACCTGGGCTCCATCTCCACCATTGTCCCGAAAT
CCATGGCCAATTCCCTCTGGGACACAAGGGCCATCTCTTACTCAGGATGTGCTGTCCAG
GTCTTTTGCTTTCTCTTTTTTATATCAGCAGAGTATTGTCTTCTCACCGTCATGGCCTACG
ACCGCTACGTTGCCATCTGCAAACCCCTGCACTACGGGACCCTCCTGGGCAGCAGAGC
TTGTGCCAAAATGGCAGCAGCTGCCTGGGGCAGTGGTTTCCTCAATGCTGTGCTTCAC
ACTGCCAATACGTTTTCAATACCACTCTGCCAAGGCAATGCTGTGGACCAGTTTTTCTG
TGAAATCCCCCAGATCCTCAAGCTCTCCTGCTCAGACTCCTACCTCAGGGAAGTTGGG
CTTCTTGTGGTTAGTCTTTCTGTAGCTTTAGGGTGTTTTGTTTTCATCGTGCTGTCCTAC
TTGCAGATCTTCAGGGCTGTGCTGAGGATCCCCTCTGAGCAGGGACGGCACAAAGCCT
TTTCCACGTGCCTCCCTCACCTGGCTGTGGTCTCCTTGTTTATCAGCTCTGGCCTGTTTG
CCTACCTGAAGCCCCCTTCCATCTCCTCCCCATCCATGGATCTGCTGGTCGCAGTTCTGT
ATGCAGTGATACCTCCAGCAGTGAACCCCCTCATCTACAGCATGAGGAACAAGGAGCT
CAAAGATGCACTGAAGAAGCTCTTTCAATCAGTTGTCTTTCAGCAGCAATAA
>Adelie_OR4D1 (Adelie20)
ATGGAGCCACAGAATGTAACCAATCCTGTGACGGAATTTGTGCTCCTGGGCTTCAACT
ACAGTCTTAAGATTCAGCAATTCCTCTTCACGGTCTTCTTTATTGTCTACCTGATGACCT
GCTTGGGAAACATCACCATCCTCACCACTGTTATCACTGACTACCACTTGCACATACCC
ATGTACTTCTTCTTGGCCAACTTAGCGTTCACAGATATTACCGAATCATCAGTAAATACT
CCCATTCTATTGTCAGGTCTCCTCTCCCAGCACAAAACTGTTCCATTCAAGGAGTGTAT
CTTTCACATGTTTGTCTTCCACTTCATTGGTGGTGCTCATATCTTCCTTCTTGCAGTGAT
GGCAGCTGATCGGTATGTAGCTGTCCATAAGCCCTTGCAGTACTTGACAATAATGAACC
GTGAGGTGTGTTTAAGCCTAGTGACAGGGTCATGGGCAGGTGGATTCATTCACTCTGC
AACACAGATTGCTCTGCTTCTCCCTTTACCTTACTGTGGTCCTAACACCCTGGACAATT
TCCACTGTGATGTCCCACAAGTACTGAAAGTGGCCTGCATTGACACCTACCAGACGGA
GCTGCTGATGGTCTCAAATGGTGGACTGCTCCTCATAGTAATTTTTGTCTTGCTGCTCAT
TTCATACACTGTCATTTTAGTCAAGATAAGGAGGCAAGTCACCAGAGGAACGCACAAA
GCTTTGTCTACCTGCATAGCCCAGATAATGGCAATAAGCATAACATTCATTCCAGGGATA
TTCATCTATGCTCAGCCCTTCAAGACATTTGAACTGGACAAAGTGGCCTCCATCTTTTT
CACTGTGCTTGTTCCAATGCTGAATCCCATGGTCTACACCCTGAGAAATACCGAAATGA
AAAAGTCCATCAGAAGACTTGTTTCTAGGGTTCTGTTCTCAGGAGAAAAGGGAATAGC
TTAA
>Adelie_OR5F1 (Adelie21)
ATGATTGTCTCCGAGCAGGTCCCACTGGAGACAGGGCTTTCCACCTGGGAGATACCTG
CCTGTTGCAGAAGAAACCTCCAGCAGGGAGTTCACACAGAAAGCACGCGTCCAGCTA
ATGGCAGAAAGCAATTGCTCCCAGGCGATTTGAATGAATTCAGCTTAACGGGGTTCAC
AGAGGGCCCAGTGACTCAGGTCACCCTCTTTCTGGTGTTTTTGCTCACCTACCTTGTCA
CCATCCTGGGGAACCTTGGCATGATTGCGTTAATCAGGGCCAGCCCTCAGCTTCACTCC
CCCATGTATTATTTCTTGGGTAACCTGGCTTTTGCAGACCTCTGTTCTTCCACCATCATC
ACCCCCGAGATGCCGGTTGACTTTACGTCAGAGAAGAAGGGCACTGCTTATGCTGGGT
GCGTGGCTCAGGTGGTCATTTCTGATCTTTTTGGGACGACCAAATGCTTCCTGCTGGCT
ATGATAGCGTATGACCATTACATGGCCGTCTGCCGTCCCCTGGTGTATCTGCTTGTCCCG

TCCCCAAAATGCTGTTTCCAGCTGGTGACTGGGTCATATCTCATGGGGTTGACAAATGA
CATGGGACAGACTACTGGCACAACCAATTTATCCTTCTGCAGCTCCAGCGTCATCGACC
TGTTCTTCTGTGACTCCTCTGATATCACTCTCAAGCTCCAACACCACCCTCAGACGCAT
ATAAGAACGTCAGCATCTTGGTTCGGTGTGGCCAGCAGCCTGGTTGGCCTGGTCTCCC
ATGTGGCCATCATCTCCGCCATCCTGAGCAACAGTTCAGCCGAGGGCAAGCACAAAGT
CTTCTCCGCCTGTGCCTCCCATCTCACCGTTGTGAGGATCTTCTACGGGACATCGCTTTT
TACGCACTTAAAGCCCAGCTCAGAGAGCTCGAGAGAAGAAGATAAACGGGCTGCAGT
GCTCTACACTGTGGTGACTCCCATGCTGAACCCCTTGATCTACAGCCTGAGGAATACGG
AGGTGAAGGAGGCTTTGAGGAGACTCACAAATATCAAATGA
>Adelie_OR6M1 (Adelie22)
ATGTTTAACCAGAGCTCTGTGGTGACTGAATTCATACTTCTGGGTTTTCCCGTGGTCTG
GGAACTGGTTGTCCTCTTCTTCATTGTGTTTCTCATAATCTATATACTGACAGTCTCTGA
GAAGGTGACCATCATCTCAGACACACTGGCTGATTGTCGACTCCACAGCCCCATGTATT
TCTTCCTTTGCAATCTCTCTTTCCCGGATATCATGGTGGCAACACCCATTGTTCCAAAAA
TGATGAAAGATCTTGTCTCTTTGGAAAAGACTGTCTCTGTACCTGGATGTATCTCACAA
TGCTATTTCTACTTCTTTTTTGGCACGACCAAAGTCATTCTCTTGGCTGTCATGTTACTT
GATCACTATGTGGCCATCTGCATCCCACTCAGATACTCCACCATCATGAGCACCCAGAT
GTCCTTCCAACTAGGGTTGGCTTCTTGGGCTGGGGGCTTCTTCTCTGGCCTCTCTCCTA
TGATCTCCTGGTTCCCCTTCTGTGGGCCAAATGTTATTGATCACTTTTTTTGTGACATTG
AGCCATTGATCAAGCTCTCTTGTGCAGATACCCATCTCCTGAAGCTGGTGAAGTTTGTA
TTGTCCACTGTGGTGCTCCTGTGCTCTCTGCTATTTACCATTCTCTCATATCTGCACATCA
TTGTACAGATTCTCCATATCCCCTCATCTTCAGGATGGAAAAAGGCCTTCTCCACTTGT
GCCTCCCACATCACTGTGGCTTCCATATTCTATGGCAGTGCCATCTTCATGTACATAGCG
CCAAACAAAGAGTTTTCCTTCAACCTCAAGAAGGCAGTCACCCTGCAGACGGTTGTTT
TCACTCACTTGCTTAATCCTTGCATCTACACTTTGAGGAACCAGAAGGTCAGGGATGGT
CTGAGGGACACTGCCTCCCACATCCTAAGTGGTACAAAGGCTTTCAGAGTGAACTGTA
AATTGAGTAGACAATAA
>Adelie_OR5R1 (Adelie23)
ATGGTTGAAGATAATTATACATTTCCATCTGAGTTTATTCTCCTGGGCTTCTCAAACCGA
GAAGACCTGCAGGTGACATGCTTTGTCTTATTCCTTGCCATCTATGTGATCACTCTAATA
GGAAATCTGGGAGTAATTATATTAATCAGAATCGATTCGTGCCTACGCACCCCCATGTAC
TTCTTCCTAAGCCACTTGTCTCTCCTGGACGTCTGCTACTCCTCCACCATCATCCCTCAA
ACCTTGCTGAATTTTTTAGTGGAGAAGAAGGTTATTTCCTTCGTTAGGTGTGCCACTCA
GCTCTTCTCCTTTGCGACTTGTGCCACCACCGAGTGCTACATGCTGGCTGCCATGGCTT
ATGATCGCTACATGGCCATTTGTAACCCCCTGCTCTACTCCGTGGTCATGTCCCAGAGG
TTTTGCGTTGGGATGTTGGCTGGTGCCTACTTAGCTGGTGTGATCAGCTCCATCATACA
CACAGTCTCCATATTTCATTTCCCGTTCTGCCGGTCCAAGAGGATCAATCATTTCTTCTG
TGATGGACTACCGCTGCTAGCCCTCTCCTGCTCTGACACCCATGTCAACGAGGCGATCG
TTTCTGCCGTGGTGGGGTTCAACATGCTAAGCACCATGGTCCTCATTCTAGTCTCCTAC
TCGTCCATCCTCTCCACTGTCTTGCGGATGCGCTCCACGGCCGGTTGTCACAAAGCCTT
CTCCACTTGCGCCCCTCACTTGGTCTCCATCGCTTTGTACTACGGCAGCTCCCTCTTCAT
GTACCTGCGCCCCGGCTCCAGACACTCCTTGGAGCGTGACAAGGTGGTCTCCGTGCTG
TACTCCATCGCGCTCCCCATGCTGAACCCGCTCATCTACAGCCTAAGAAACACGGACAT
GAAGAACGCCATGAGAAAAGCGAAAGGTAGAGTCCTCTCCTCCTTGTCCATCCACGGT

TCCGGGCCAGGTGAAAGGAGAGGGCTACCCTTGCATGGTAAGGAGGGGTAG
>Adelie_OR4D5 (Adelie24)
ATGGCACTGGGAAACTTCTCCCGGGTGACTGAATTCATCCTCCTGGGGCTTTCCGATAC
AAGGGAGTTGCAAGTCCTCTTCTTCACTTTCTTCTTCCTGGCCTATGCCATGGTTCTGCT
GGGAAACCTTCTCATCATTGTGACAGTCAGGACCGACCCCAAGCTGTCCTCACCCATG
TACTTTCTCCTCTGCAATTTGTCCTTCATAGATATCTGCTGCACCTCTGTCACCTCTCCC
AGGATGCTGGTGGACCTGCTCTCCCAGAGGAAGGCCATTGCTTTCGAAGACTGTATAG
CCCAGCTGTTTTTTCTGCACTTTGTTGGGGCATCAGAGATGTTCCTCCTGTCTGTGATG
GCGTATGACCGCTACACTGCCATCTGCAAGCCCCTGCACTACACAGCCATCATGAGCCG
GCAGGTATGCTGGGTCCTGGTGTCTGCCTGCTGGGCAGGGGGCTTCCTCCACTCCATT
GTCCAGACGCTGCTCACAATCCAGCTCCCCTTCTGCGGCCCTAACACAATCGACAACT
ACTTCTGTGACGTGCCTCCTGTCATTCGGCTTGCCTGCACAGACATCTACATCACTGAG
TGGCTCATGGTATCCAACAGCGGCTTGATATCCCTGGTTTGCTTCCTGGTGCTGGTCAC
ATCTTACACGTTCATCCTGGTCACAATCAGGGTCCGCTTCACTGAGGGGAACTGGAAG
GCGCTCTCCACCTGTGCCTCACATGTGATGGTCGTCACCCTCTTCTTTGTACCCTGCATC
TTCATCTACCTCCGTCCCTTTTCTACCTTCCCCTCCGACAAGCACATCTGTGTGATCTGC
ACTGTCTTCTCCCCGGTGATGAACCCCCTCATCTATACCCTGAGGAATAACGAGGTGAA
GGCATCCATGTGGAAGTTATGGAAGCGCTGCAGAATCTTCTGA
>Adelie_OR11A1 (Adelie25)
ATGACAAAGCAAGAGTGGAAAAACAAAACGGTTGTTACAGAGTTCATCCTCCTGGGA
TTTAGGAATGGCCCCAAACTGGATTCTCTTCTCTTCCTGATGTTTCTGTCAATCTACATT
GTGACCATAACTGGAAACATCTTCATCATTGTGCTGGTGGTGGCTAATCGGCACCTTCA
CACCCCAATGTACTTCTTCCTGGGCAATTTGGCCTGCTTGGAAATCTGCTACAGCTCAA
ATATCTTGCCGAGGATGTTGCTTAGCTACCTGGGTGGAGACAGAAGTATTTCAGTCAAT
GGATATTTTACACAATACTATTTCTTTGGTTGCTTGGGAGTTGCAGAATGCTATCTCCTC
ACAGTGATGTCCTATGATAGGTACCTAGCGGTGTGCAAACCCCTGCACTATCCTTCCCG
TATGAACGGCAAGCTCTGTCTCCAGCTGGGTGCTGCATCTTGGATAAGTGGCTTTCTAA
CTAATTCTATACTAACATTCCTGATCTCAAATTTAGACTTCTGTGGGCCTAATGAAATCG
ACCATTTTTTCTGTGACTCATTCCCAATGATAAAACTCTCATGTAGTGACAGTCATGTGG
TGGGACTTGTCACTTCTGTTGTGGCGGGTGTGTGCTCACTCCCTCCGTTTCTGTTGACC
TTCTCATCCTATCTCTACATCATTGTCACGGTCATGAGAATTCTTTCTGCCACTGGAAGG
AAAAAGGCCTTTTCCACTTGCTCCTCACATCTTATTGTGGTGATTCTTTTTTACTGGTCA
ATATTAACTGTCTATGTACTTCCTCATCACGATACCCAAATATCTCCCAACAAAGTCTTC
TCTGTTTTTTATACCATTCTCACTCCCTTGGTCAATCCTCTCATCTACAGTCTGAGAAAC
AAAGAGGTAAAGGAAGCTCTGCGAAAACAGGCAAGTAAATTGCTGGCTTTCAGAGGG
CTGCTTTCTGTTAAAAAAAGGGGGTAA
>Adelie_OR52K1 (Adelie26)
ATGTTTTTCTTGACTGACATCCCTGGGCTGGAAGATATGCACCCCTGGACTTCCATCCC
ATTCAGCAGAGTGTTCAGCGTAGCCCTCCTTGGGAACAGCACCCTCTTGTACGTGATG
AAGACAGAGCCATCTCTCCACAAGCCCATGTTTTATTTCCGCTCCATGCTGGCTGTCAT
TGACTTGGTGCTGTCCCTGACCACCATGCCCAAAATGCTGATCATCTTCTGGTTCAATG
CCCAGGAGATCACCTTCAATTTCTGCCTTGTGCAGATGTTTTTTCTTCACACTTTCTCAG
TCATGGAGTTGGTGGAGCTGCTGGCCGTGGCTTTCCACATGTATGCTGCCATCTGCAAC
CCCCTGAGCTACAAGCTCCATCCTGACCATCTCCCTGGTAGTCAAGAATGGGCTGCTG

GCTCTAGCCAGGGCAGCCGGGCTCATGTTGCCTCTGCCTTTCTCCTCCATTGCCTGCTG
TACTGCTGCTCCCACATCATCTCCCACTGCTACTGCAAACACATGGCAGCGGTGGAGCT
GTCCTGTGCCAACACCAGGTTCAGTAACATCATCGTGGTTCTCTTCATCATGGGGTTGG
ATCTGTTGTTCATTGGGCATTCCTACCTCAGGATCTTGAGGACCATGTTGAGCCTGGCA
TTGAAGGAGGACAGACTGAAGGTATTTGGGACCTGCCTCTCCCACATCTTCGTCATCCT
GGTCTTCTACACCCCTGTGGTTCTCTCATTAGTAATCCACAGGTTTGGTTGCCATATTGC
CTCTCACATGCACATCTTGGTGGCTAATTTCTACCTCCCATTCCCCCCCATGGTGTATGG
TGTCAAAACCAAGCATATCCATGACCAGGTGCTCATTGCATTTTTCTCAAGAGAATCTA
AATTCTCACACATCCCATCCACAGGAGCATGTACGGATATCCAAACAACTGCACATGCA
AATTGCAGCTCATGA
